# Health worker education during the COVID-19 pandemic: global disruption, responses and lessons for the future—a systematic review and meta-analysis

**DOI:** 10.1186/s12960-023-00799-4

**Published:** 2023-02-24

**Authors:** Aikaterini Dedeilia, Michail Papapanou, Andreas N. Papadopoulos, Nina-Rafailia Karela, Anastasia Androutsou, Dimitra Mitsopoulou, Melina Nikolakea, Christos Konstantinidis, Manthia Papageorgakopoulou, Michail Sideris, Elizabeth O. Johnson, Siobhan Fitzpatrick, Giorgio Cometto, Jim Campbell, Marinos G. Sotiropoulos

**Affiliations:** 1 Department of Surgery, Massachusetts General Hospital, Boston, MA USA; 2 Harvard Medical School, Boston, MA USA; 3 HEALth Workforce Education (HEAL-Edu) Study Group, Athens, Greece; 4 School of Medicine, National and Kapodistrian University of Athens, Athens, Greece; 5 Society of Junior Doctors (SJD), Athens, Greece; 6 Department of Hygiene, Epidemiology and Medical Statistics, School of Medicine, National and Kapodistrian University of Athens, Athens, Greece; 7 School of Medicine, Aristotle University of Thessaloniki, Thessaloniki, Greece; 8 School of Medicine, University of Patras, Patras, Greece; 9 Barts and the London School of Medicine and Dentistry, Queen Mary University of London, London, United Kingdom; 10 School of Medicine, European University Cyprus, Nicosia, Cyprus; 11 Health Workforce Department, World Health Organization, Geneva, Switzerland; 12 Department of Neurology, Brigham and Women’s Hospital & Massachusetts General Hospital, 55 Fruit Street, WACC721, Boston, MA 02114 USA

**Keywords:** Health worker, Healthcare worker, Education, Training, COVID-19, Pandemic, Online learning, Assessment, Disruption, Mental health, Volunteering

## Abstract

**Background:**

This systematic review and meta-analysis identified early evidence quantifying the disruption to the education of health workers by the COVID-19 pandemic, ensuing policy responses and their outcomes.

**Methods:**

Following a pre-registered protocol and PRISMA/AMSTAR-2 guidelines, we systematically screened MEDLINE, EMBASE, Web of Science, CENTRAL, clinicaltrials.gov and Google Scholar from January 2020 to July 2022. We pooled proportion estimates via random-effects meta-analyses and explored subgroup differences by gender, occupational group, training stage, WHO regions/continents, and study end-year. We assessed risk of bias (Newcastle–Ottawa scale for observational studies, RοB2 for randomized controlled trials [RCT]) and rated evidence certainty using GRADE.

**Results:**

Of the 171 489 publications screened, 2 249 were eligible, incorporating 2 212 observational studies and 37 RCTs, representing feedback from 1 109 818 learners and 22 204 faculty. The sample mostly consisted of undergraduates, medical doctors, and studies from institutions in Asia. Perceived training disruption was estimated at 71.1% (95% confidence interval 67.9–74.2) and learner redeployment at 29.2% (25.3–33.2). About one in three learners screened positive for anxiety (32.3%, 28.5–36.2), depression (32.0%, 27.9–36.2), burnout (38.8%, 33.4–44.3) or insomnia (30.9%, 20.8–41.9). Policy responses included shifting to online learning, innovations in assessment, COVID-19-specific courses, volunteerism, and measures for learner safety. For outcomes of policy responses, most of the literature related to perceptions and preferences. More than two-thirds of learners (75.9%, 74.2–77.7) were satisfied with online learning (postgraduates more than undergraduates), while faculty satisfaction rate was slightly lower (71.8%, 66.7–76.7). Learners preferred an in-person component: blended learning 56.0% (51.2–60.7), face-to-face 48.8% (45.4–52.1), and online-only 32.0% (29.3–34.8). They supported continuation of the virtual format as part of a blended system (68.1%, 64.6–71.5). Subgroup differences provided valuable insights despite not resolving the considerable heterogeneity. All outcomes were assessed as very-low-certainty evidence.

**Conclusion:**

The COVID-19 pandemic has severely disrupted health worker education, inflicting a substantial mental health burden on learners. Its impacts on career choices, volunteerism, pedagogical approaches and mental health of learners have implications for educational design, measures to protect and support learners, faculty and health workers, and workforce planning. Online learning may achieve learner satisfaction as part of a short-term solution or integrated into a blended model in the post-pandemic future.

**Supplementary Information:**

The online version contains supplementary material available at 10.1186/s12960-023-00799-4.

## Background

The Coronavirus Disease 2019 (COVID-19) pandemic has affected human health to an unprecedented degree: more than 569 million cases had been reported by July 2022 and an estimated 14.9 million excess deaths was reported in May 2022 [1]. This has been accompanied by profound disruption to health worker education, due to distancing, restrictions on access to learning facilities and clinical sites, or learner and faculty infection or illness [2, 3]. In response, many institutions rapidly embraced digital innovation and other policy responses to support continued learning [4].

Building on an earlier review by the same authors [5], this paper seeks to quantify the educational innovations and their outcomes since the start of the pandemic, as documented in published studies [6, 7], capturing different regions, levels of training, and occupations [8]. The pertinent challenge is how to translate this evidence into enduring policies, strategy and regulation on the instruction, assessment and well-being of health worker learners [9], in accordance with the WHO Global Strategy on Human Resources for Health: Workforce 2030 [10].

The aim of this systematic review and meta-analysis is to identify and quantify the impact of COVID-19 on the education of health workers worldwide, the resulting policy responses, and their outcomes, providing evidence on emerging good practices to inform policy change.

**Fig. 1:**
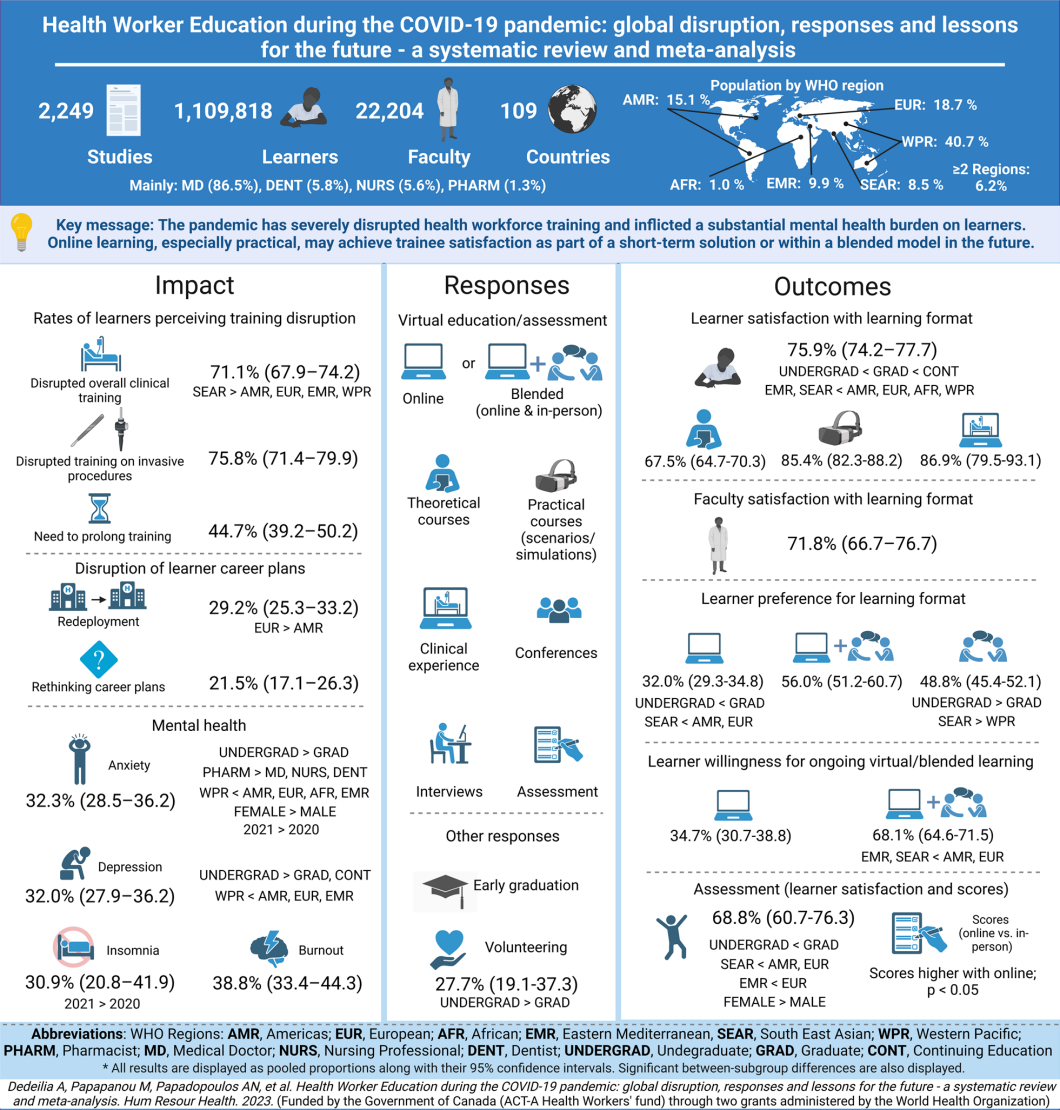
Graphical abstract of the systematic review and meta-analysis

A graphical abstract summarizing our systematic review and meta-analysis in a cohesive and legible way is presented in Fig. 1.

## Methods

### Study design

We conducted a systematic review and meta-analysis in accordance with the Measurement Tool to Assess Systematic Reviews-2 (AMSTAR-2) checklist [11] and the Preferred Reporting Items for Systematic Reviews and Meta-Analyses (PRISMA) 2020 statement [12], based on a predesigned protocol registered with PROSPERO (CRD42021256629) [13].

### Search strategy

We searched the MEDLINE (via PubMed), EMBASE, Web of Science, and CENTRAL databases, as well as ClinicalTrials.gov and Google Scholar (first 300 records) for randomized controlled trials (RCTs) or observational studies published from 1/1/2020 to 31/07/2022 in English, French or German (full search strategy available in Additional file 1). A snowball approach was also employed.

### Eligibility criteria and outcomes

Our eligible population included Health Worker (HW) learners or faculty, as defined by the International Standard Classification of Occupations (ISCO-08) [14] group of health professionals, excluding veterinarians. Health care settings per the Classification of Health Care Providers (International Classification for Health Accounts, ICHA-HP) [15] and relevant educational settings (i.e., universities, colleges) were considered eligible. The included population was divided into undergraduate learners, postgraduate (e.g., residents or fellows) and continuing education (in-service) [16]. Any change(s) and/or innovation(s) that were implemented in health worker education in response to the COVID-19 pandemic (not before the COVID-19 pandemic or amidst other pandemics) were considered eligible. Online training methods were sub-divided into predominantly theoretical courses, courses with a practical component (i.e., practical skill, simulation-based training), congresses/meetings, interviews, and clinical experience with patients (i.e., clinical rotations/electives, telehealth-based training). Comparators included conventional/traditional practices existing prior to the pandemic.

The study outcomes are organized according to (1) impact of the COVID-19 pandemic on the educational process and mental health of learners; (2) policy responses (not included in the meta-analysis); and (3) outcomes of those policy responses (Table 1). Specific meta-analysis outcomes in the categories shown in Table 1 included: regarding axis 1, clinical training, mental health (i.e., anxiety, depression, insomnia and burnout), and learner career plan disruptions (e.g., redeployment), and concerning axis 3, satisfaction, preference and performance with new training and assessment modalities and volunteerism, including any social/community/institutional work. Regarding anxiety and depression, individuals whose symptom severity was deemed moderate or higher according to validated measurement scales were considered as affected. For the Generalized Anxiety Disorder-7 (GAD-7) and Patient Health Questionnaire-9 (PHQ-9) screening tools, this corresponded to a cut-off score of 10.

**Table 1:** Outcomes framework for the systematic review

Main axis	Variables examined
1. Impacts of the pandemic on health worker education	1.1 Disruption to clinical training
	1.2 Disruption of career plans
	1.3 Mental health of learners: scaled anxiety, depression, burnout, and insomnia
2. Policy and management responses to those impacts	2.1 Transition to online or blended learning
	• Theoretical courses
	• Practical courses
	• Clinical experience
	• Conferences
	• Interviews
	2.2 Training on COVID-19 specific protocols
	2.3 Online assessment
	2.4 Volunteerism initiatives
	2.5 Early graduation, other policies and responses
3. Outcomes of policy responses	3.1 Online and blended learning
	• Satisfaction
	• Preference during the pandemic
	• Preference for the future
	3.2 Online assessment
	• Scores and performance
	• Learner and faculty perceptions (satisfaction and preference)
	3.3 Intention to participate and participation of learners in volunteering activities

### Literature search and data extraction

All retrieved records underwent semi-automatic deduplication in EndNote 20 (Clarivate Analytics) [17], and were then transferred to a Covidence library (Veritas Health Innovation, Melbourne, Australia) for title and abstract screening. Pairs of authors performed a blind scan of a random 15% sample of records. After achieving an absolute agreement rate > 95% (Fleiss’ kappa, 1st phase: 0.872, 99% confidence interval (CI) [0.846–0.898]; 2nd phase: 0.840, 99% CI [0.814–0.866]), single-reviewer screening was performed for the remainder of the studies, as per the AMSTAR-2 criteria [11]. Subsequently, pairs of independent reviewers screened the full texts of the selected studies for eligibility, and, if eligible, extracted the required data in a predetermined Excel spreadsheet. Screening and data extraction was carried out in two phases: the initial phase (1/1/2020 to 31/8/2021 by AD, ANP, M. Papapanou and MGS) and the updated living phase (1/9/2021 to 31/7/2022 by NRK, AA, DM, MN, CK, M. Papageorgakopoulou). After discussion with the WHO technical partner, we amended the extraction spreadsheet to further include descriptions of policies in the updated living phase. Satisfaction was extracted either from direct mentions of participants’ satisfaction by the authors or from questions surveying the participants’ perceptions on their satisfaction, the success, usefulness or effectiveness of the learning activity. Conflicts were resolved by team consensus. For missing data, study investigators were contacted. Studies for which the full text or missing data were unable to be retrieved were categorized as “reports not retrieved”. Studies on overlapping populations were also considered duplicates and subsequently removed if they related to the same study period and institution(s) and involved similar populations and author lines. The study with the most comprehensive report was retained.

### Risk of bias, publication bias and certainty of evidence

Pairs of all aforementioned authors performed the risk of bias assessment, and any conflicts were resolved by team consensus. The quality assessment was performed using an adapted version of the Newcastle–Ottawa Scale (NOS) for cross-sectional studies (Additional file 1), the original NOS for cohort and case–control studies, and the Cochrane risk-of-bias (RoB2) tool (Version-2) for RCTs. Publication bias was explored with funnel plots and the Egger’s test [18]. Certainty of evidence was assessed using the Grading of Recommendations, Assessment, Development and Evaluations (GRADE) approach [19].

### Data synthesis

Categorical variables were presented as frequencies (%) and continuous variables as mean (standard deviation [SD]). To dichotomize ordinal data (e.g., Likert-type scales), we used the specific author provided cut-offs for the respective scales, or, if not provided, the 60th percentile (40th if the scale was reversed). Regarding mental health outcomes, we derived scale-specific cut-offs from the literature.

Analyses were carried out on learner and faculty population subsets separately. We carried out a meta-analysis of the Freeman–Tukey (FT) double-arcsine transformed estimates using the DerSimonian and Laird (DL) random-effects model [20–22]. We used the harmonic mean in the back-transformation formula of FT estimates to proportions [23]. For each meta-analyzed outcome, we reported the raw proportion (%), pooled proportion (%) along with its 95% CI, the number of studies (*n*) and number of included individuals (*N*). When applicable, we pooled standardized mean differences (SMDs) with the method of Cohen [24]. Statistical heterogeneity was quantified by the *I*^2^ [25], and was classified as substantial (*I*^2^ = 50–90%) or considerable (*I*^2^ > 90%) [26].

### Subgroup and sensitivity analyses

We performed subgroup analyses stratified by gender, continent, WHO geographical region, ISCO-08 occupational group, stage of training, and year of undergraduate studies, and computed *p*-values for subgroup differences (*p*_subgroup_ < 0.10 indicates statistically significant intra-subgroup differences) [26]. The potential effect of time on outcomes potentially exhibiting dynamic changes during the evolution of the pandemic, such as satisfaction and preference with learning formats, as well as mental health outcomes, was explored via additional subgroup analyses by year data collection was completed (2020 vs 2021 vs 2022). Only subgroups involving 3 or more studies are presented and taken into account for the *p*_subgroup_ calculation, so no subgroup analysis is presented for the 2022 study end year.

Sensitivity analyses excluding studies with *N* > 25 000 were performed to minimize the risk for duplicate populations that may be introduced by large-scale nationwide studies. Regarding anxiety, depression and burnout, sensitivity analyses restricted to studies employing the GAD-7, PHQ-9, and Maslach Burnout Inventory (MBI, including its variants), respectively, and, even further, their low-risk-of-bias subsets were carried out.

To better account for the anticipated substantial heterogeneity, two additional meta-analytical approaches were used: (i) the Paule–Mandel estimator to calculate the between-study variance [27]; and (ii) the Hartung–Knapp method for the CI calculation [28].

Statistical significance for all analyses was set at a two-sided *p* < 0.05. All analyses were conducted using aggregate data via the STATA software, version 16.1 (Stata Corporation, College Station, TX, USA). Further explanation of adopted statistical approaches is provided in Additional file 1.

## Results

**Fig. 2:**
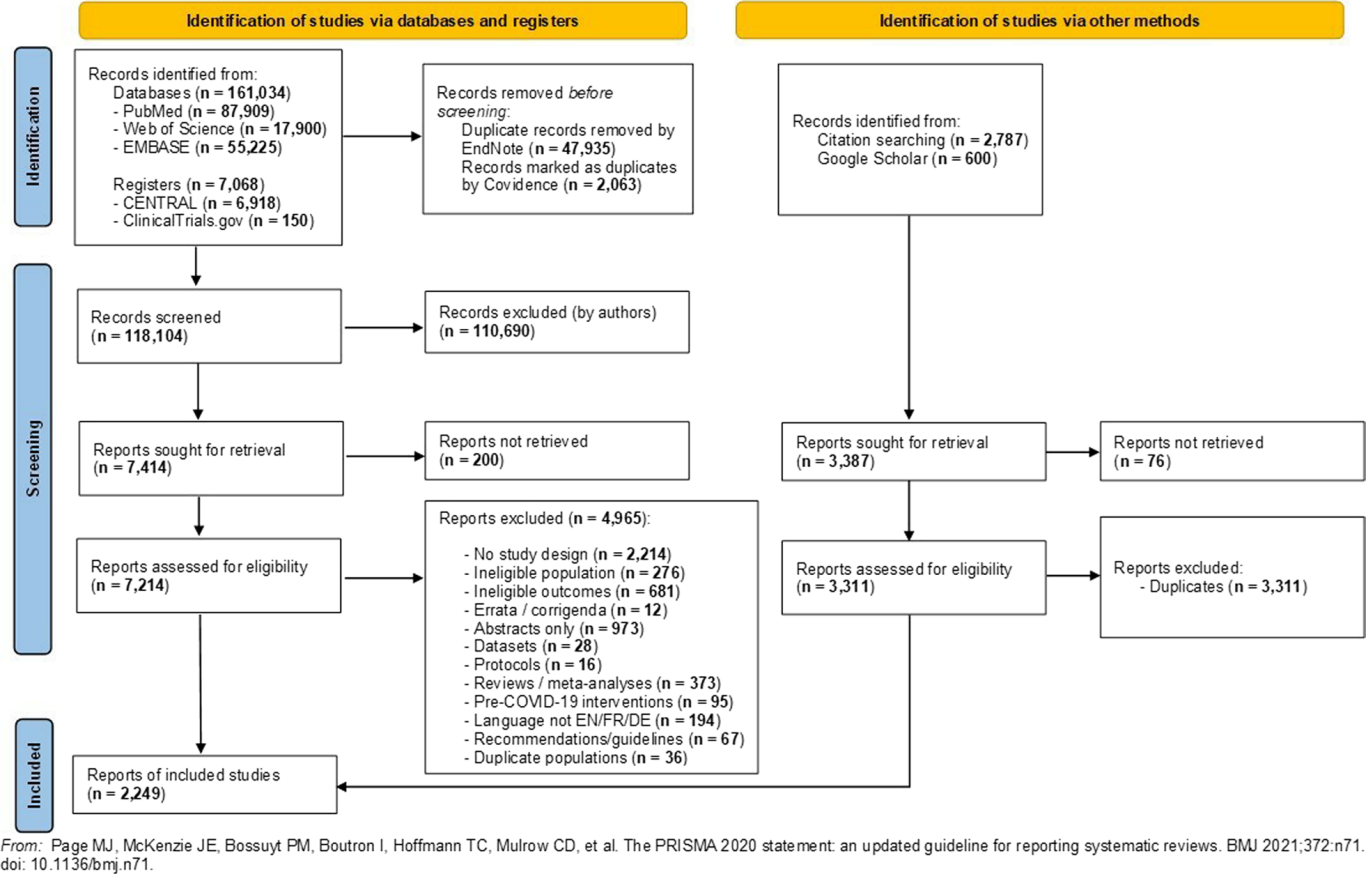
PRISMA 2020 flow diagram

The literature search yielded a total of 171 489 publications (168 102 from databases and 3 387 from snowball and Google Scholar). Following deduplication and title-abstract screening, a total of 10 525 publications (7 214 from database/register search, and 3 311 from snowball/Google Scholar) were assessed for eligibility, of which a total of 2 249 were included in the systematic review. Of these, 2 212 were observational studies (2 079 cross-sectional), and 37 RCTs. The PRISMA 2020 flow diagram is available in Fig. 2. All our included studies are cited in Additional file 2.

Overall, 1 149 073 individuals (1 109 818 learners [96.6%], 22 204 faculty [1.9%], 12 544 combined learner and faculty participants [1.1%], and 4 507 education leaders representing institutions [0.4%]) across 109 countries from 6 continents/WHO regions were included. The total number of women was 468 966 (63.4%) out of 739 127 participants whose gender was reported. Of the studies included in the meta-analysis and pertaining to the impact of the pandemic, 314 focused on training disruption, 193 on career plans disruption, and 287 on the mental health of learners; regarding the outcomes of policy responses, 1013 studies focused on innovations in learning, 121 on online assessment methods and 48 on volunteerism.

Characteristics of included individuals and settings per outcome are available in Table 2A, B, Additional file 3 and Additional file 4. The sample mostly represented undergraduate learners (81.4%), within the field of medicine (86.5%), in studies originating from institutions in Asia (59.9%) and the Western Pacific WHO Region (WPR, 40.7%).

**Table 2:** Characteristics of included individuals and settings

A: Characteristics of included individuals					
Category	Sub-category	Number of studies	Number of participants	Percentage of sub-category participants (%)	
Total	N/A	2 249	1 149 073	N/A	
Gender	Total	1 099	739 127	100.0	
	Female	1 099	468 966	63.4	
	Male	1 099	270 161	36.6	
Learner or faculty	Total	N/A	1 149 073	100.0	
	Learners	2 062	1 109 818	96.6	
	Faculty	252	22 204	1.9	
	Mixed populations of learners and faculty	49	12 544	1.1	
	Program directors (representing entire institutions)	45	4 507	0.4	
Training stage of learner	Total separate data on training stage	N/A	931 008	100.0	
	Undergraduates	1186	757 618	81.4	
	Postgraduates	645	121 475	13.0	
	CPD	176	51 915	5.6	
Year of studies (for undergraduates only)	Total	N/A	67 065	100.0	
	1st	146	23 036	34.3	
	2nd	91	8 673	12.9	
	3rd	110	10 808	16.1	
	4th	122	14 671	21.9	
	5th	48	5 003	7.5	
	6th	27	4 775	7.1	
	7th	2	99	0.1	
Training stage of faculty/teacher	Total separate data on training stage	N/A	15 855	100.0	
	Undergraduate	14	1 187	7.5	
	Postgraduate	19	2 431	15.3	
	Continuing	145	12 237	77.2	
Occupational group as per ISCO-08	Total	N/A	984 407	100.0	
	Medical doctors	1 505	851 961	86.5	
	Nursing professionals	264	54 999	5.6	
	Midwifery professionals	5	284	0.0	
	Traditional and complementary medicine professionals	1	733	0.1	
	Paramedical practitioners	8	559	0.1	
	Dentists	169	56 823	5.8	
	Pharmacists	73	12 314	1.3	
	Environmental and occupational health and hygiene professionals	2	390	0.0	
	Physiotherapists	19	3 634	0.4	
	Dieticians and nutritionists	2	581	0.1	
	Audiologists and speech therapists	4	874	0.1	
	Optometrists and ophthalmic opticians	3	1 255	0.1	
Medical doctor or different occupational group	Total	N/A	984 407	100.0	
	Medical doctors	1 505	851 961	86.5	
	Other health professionals	N/A	132 446	13.5	

B. Characteristics of included settings					
Category	Sub-category	Number of studies	Percentage of sub-category studies (%)	Number of participants	Percentage of sub-category participants (%)
Study design	Total	2 249	100.0	1 149 073	100.0
	Randomized trials	37	1.6	2 660	0.2
	Cross-sectional studies	2 079	92.4	1 118 355	97.3
	Case–control	25	1.1	3 848	0.3
	Retrospective cohorts	79	3.5	20 471	1.8
	Prospective cohorts	29	1.3	3 739	0.3
Continent	Total	2 244	100.0	1 148 118	100.0
	North America	698	31.1	142 111	12.4
	South America	59	2.6	31 015	2.7
	Europe	475	21.2	167 756	14.6
	Asia	790	35.2	687 320	59.9
	Africa	65	2.9	27 495	2.4
	Oceania	51	2.3	8 339	0.7
	2 or more continents	106	4.7	84 082	7.3
WHO region	Total	2 244	100.0	1 148 118	100.0
	Region of the Americas	756	33.7	173 061	15.1
	European Region	548	24.4	214 159	18.7
	African Region	47	2.1	11 090	1.0
	Eastern Mediterranean Region	274	12.2	113 546	9.9
	South-East Asian Region	259	11.5	97 951	8.5
	Western Pacific Region	255	11.4	467 230	40.7
	2 or more WHO regions	105	4.7	71 081	6.2
Study setting	Total	2 150	100.0	1 100 061	100.0
	University/college	977	45.5	757 315	68.8
	WHO health care provider (hospital, medical office, etc.)	1063	49.4	248 798	22.6
	University/college and WHO health care provider	110	5.1	93 948	8.5
WHO health care provider	Total	1 161	100.0	337 141	100.0
	General hospitals	1 126	96.9	331 523	98.3
	Mental health hospitals	9	0.8	1 444	0.4
	Specialized hospitals	12	1.0	1 781	0.5
	Long-term nursing care facilities	2	0.2	73	0.0
	Dental practice	9	0.8	2 199	0.7
	Other healthcare practitioners	1	0.1	17	0.0
	Pharmacies	2	0.2	104	0.0
Type of hospital	Total	741	100.0	158 556	100.0
	Academic teaching	718	96.9	154 217	97.3
	Community Teaching	17	2.3	3 466	2.2
	Non-teaching	6	0.8	873	0.6

Characteristics of included (A) participants and (B) settings. Counts and percentages of included studies and study participants according to gender, learner/faculty status, trainee level and occupation (A), as well as geographical region (continent/WHO region), study setting (university/WHO health care provider), and study design (B). The number of studies capturing the participants’ continent, gender, learner/faculty status, training stage, and year of studies does not sum to the corresponding total number of studies of each category. Additional demographics for each included outcome are available in Additional file 4

Thirty-seven RCTs were included: 20 out of them were assessed as at high risk of bias, 12 at low risk of bias, and 5 at risk of bias with some concerns. They mostly compared newly developed virtual, gamified or in-person learning for medical or nursing students during the COVID-19 pandemic to prior established teaching methods. They mostly showed better learning outcomes with the innovative modalities, with some studies showing no significant difference. More details are available in Additional file 5. Based on the NOS and adapted NOS scales, the median (Q1–Q3) quality score of all observational studies was 6 (4–7), [5 (4–7) for cross-sectional; 6 (5–7) for retrospective; 5 (4–7) for prospective cohorts; and 7 (6–7) for case-controls] (Additional file 3).

**Fig. 3:**
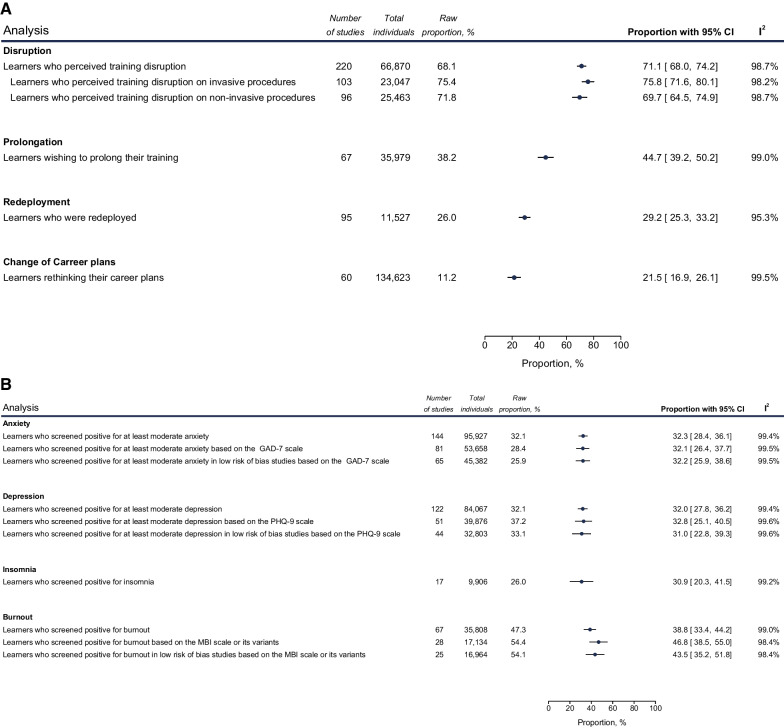
Meta-analysis of impact of COVID-19 on Health Worker Education. Random-effect meta-analyses of proportions reflecting the impact of the pandemic on health worker education. **A** Disruption of learning, redeployment, changes of career plans and potential prolongation of studies. **B** mental health effects of the pandemic on learners. Each analysis is depicted as a cyclic data marker; the horizontal lines indicate the 95% confidence intervals (CI). The “raw proportion (%)” is derived from simple weighted division. *I*^2^ quantifies heterogeneity, which is statistically significant (*p* < 0.01) in all cases (metric omitted)

**Fig. 4:**
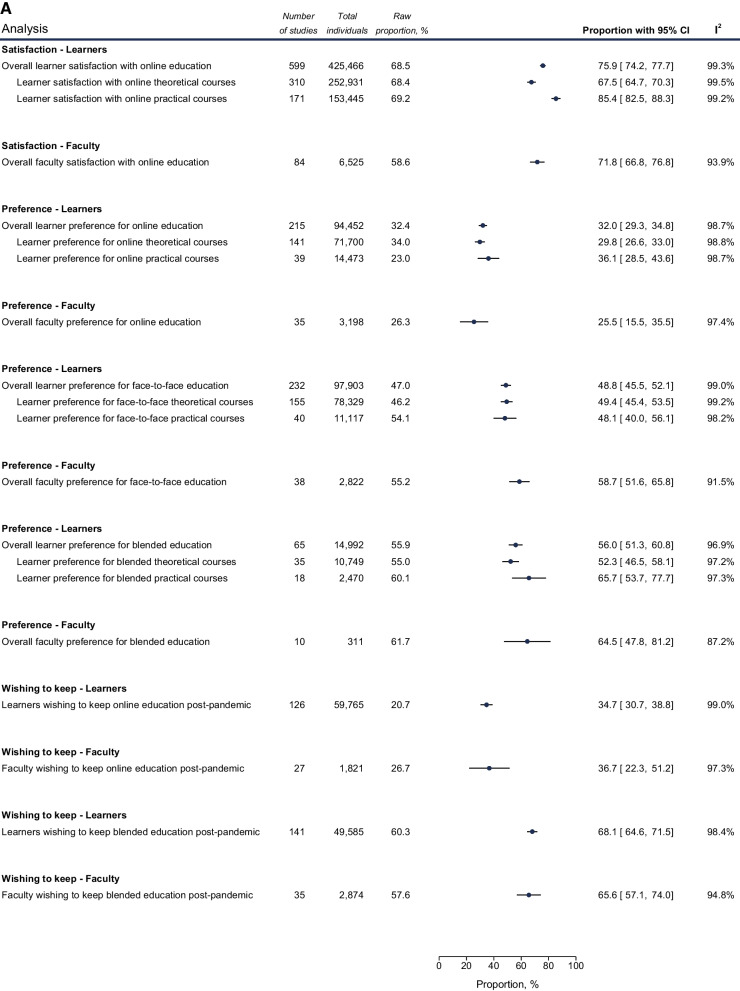

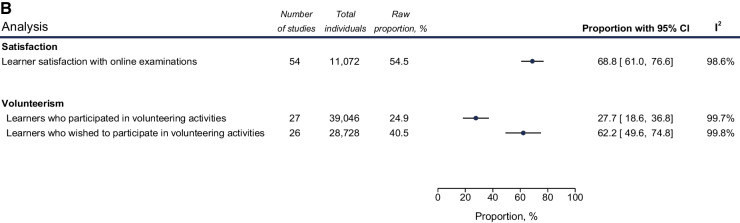
Meta-analysis of outcomes of policy responses. Random-effect meta-analyses of proportions reflecting the outcomes of policy and management responses in regard to the pandemic. **A** Learner and faculty perceptions on online and blended forms of learning. **B** Satisfaction with online assessments and volunteerism initiatives. Each analysis is depicted as a cyclic data marker; the horizontal lines indicate the 95% confidence intervals (CI). The “raw proportion (%)” is derived from simple weighted division. I^2^ quantifies heterogeneity, which is statistically significant (*p* < 0.01) in all cases (metric omitted)

The main results of our systematic review and meta-analysis are analyzed below, along with the most noteworthy subgroup results. Figures 3 and 4 also depict the main meta-analysis outcomes from Axes 1 and 3 (i.e., impact of the pandemic on health worker education and Outcomes of policy responses, Table 1). All results from subgroup analyses based on gender, ISCO-08 group, continent, WHO region, training level and undergraduate year of studies are detailed in Tables 3, 4, 5, 6, 7, 8, 9, 10, 11, 12, 13, 14, and 15. The full spectrum of analyses is also available in more detail in Additional file 6.

**Table 3:** Learners perceiving disruption of their clinical training amidst the COVID-19 pandemic by subgroups

Explanation of outcome	Subgroup	*n*	*N*	Pooled proportion (%)	Lower confidence interval (%)	Higher confidence interval (%)	*I*^2^ (%)	*P*-value for subgroup difference
Learners who perceived training disruption by ISCO group	Medical doctors	181	46 846	71.4	67.8	74.9	98.6	0.719
	Nursing professionals	8	4 190	68.3	54.6	80.6	98.7	
	Dentists	21	9 631	68.2	59.3	76.4	98.4	
Learners who perceived training disruption by training level	Undergraduate	56	36 568	71.5	65.2	77.3	99.4	0.992
	Graduate	145	23 515	70.9	67.1	74.6	97.4	
	Continuing	3	828	71.1	29.1	98.6	99.2	
Learners who perceived training disruption by undergraduate year of studies	1st	5	420	78.6	68.0	87.7	82.5	0.729
	2nd	8	668	73.8	63.3	83.1	87.4	
	3rd	7	734	68.4	46.0	87.2	97.1	
	4th	6	940	68.9	53.5	82.5	92.1	
	6th	4	769	69.3	56.6	80.8	90.8	
Learners who perceived training disruption by gender	Women	10	4 564	77.1	66.8	85.9	98.2	0.304
	Men	8	1 093	69.2	56.8	80.4	93.4	
Learners who perceived training disruption by continent	North America	63	10 743	66.9	61.0	72.5	97.4	0.103
	South America	9	2 687	69.4	48.7	86.8	98.9	
	Europe	62	14 418	70.6	65.1	75.8	97.9	
	Asia	49	18 385	76.4	71.9	80.6	97.8	
	Africa	7	4 426	80.1	65.9	91.3	98.2	
	Oceania	7	2 238	74.5	68.1	80.4	84.0	
Learners who perceived training disruption by WHO region	American	72	13 430	67.1	61.3	72.8	97.9	**< 0.001**
	European	66	15 249	71.1	65.9	76.0	97.8	
	African	3	426	73.8	63.1	83.3	81.6	
	Eastern Mediterranean	24	12 019	71.6	60.7	81.3	99.3	
	South East Asian	21	7 809	84.5	80.3	88.4	95.3	
	Western Pacific	11	3 964	69.9	60.2	78.8	97.0	
Learners who perceived disruption of non-invasive procedures (outpatient, inpatient, etc.) by training level	Undergraduate	12	7 827	68.4	52.3	82.6	99.4	0.866
	Graduate	73	13 371	69.5	63.5	75.2	98.1	
Learners who would want to prolong their training, due to the disruption caused by the COVID-19 pandemic by training level	Undergraduate	10	20 015	50.8	39.3	62.4	99.5	0.318
	Graduate	51	13 897	44.0	36.5	51.6	98.6	

Statistically significant differences (*p* < 0.05) or trends (*p* < 0.1) are noted in bold

*ISCO* International Standard Classification of Occupations, *n* number of studies, *N* number of participants

**Table 4:** Learner redeployment rates due to the COVID-19 pandemic by subgroups

Explanation of outcome	Subgroup	*n*	*N*	Pooled proportion (%)	Lower confidence interval (%)	Higher confidence interval (%)	*I*^2^ (%)	*P*-value for subgroup difference
Learners who were redeployed due to the COVID-19 pandemic by ISCO group	Medical doctors	89	10 903	27.8	23.9	31.9	95.2	0.204
	Dentists	4	390	46.4	19.3	74.6	96.5	
Learners who were redeployed due to the COVID-19 pandemic by continent	North America	37	4 596	24.8	19.4	30.6	94.6	0.146
	Europe	36	4 053	34.9	28.4	41.6	94.6	
	Asia	10	1 440	31.1	16.3	48.1	97.7	
	Africa	4	326	32.0	8.7	61.2	96.3	
Learners who were redeployed due to the COVID-19 pandemic by WHO region	American	39	4 838	24.7	19.5	30.3	94.4	**0.092**
	European	37	4 156	35.2	28.8	41.8	94.6	
	African	3	276	40.7	10.2	75.8	97.0	
	Eastern Mediterranean	5	648	25.9	9.5	46.6	96.5	
	South East Asian	3	420	13.7	0.1	43.8	97.7	

Statistically significant differences (*p* < 0.05) or trends (*p* < 0.1) are noted in bold

*ISCO* International Standard Classification of Occupations, *n* number of studies, *N* number of participants

**Table 5:** Learners’ scaled anxiety, depression, burnout and insomnia during the COVID-19 pandemic by subgroups

Explanation of outcome	Subgroup	*n*	*N*	Pooled proportion (%)	Lower confidence interval (%)	Higher confidence interval (%)	*I*^2^ (%)	*P*-value for subgroup difference
Learners who screened positive for at least moderate anxiety by ISCO group	Medical doctors	98	76 730	30.4	25.6	35.3	99.5	**< 0.001**
	Nursing professionals	11	3 196	33.0	20.1	47.4	98.5	
	Dentists	14	4 812	32.4	25.4	39.7	96.3	
	Pharmacists	4	643	50.0	45.6	54.5	19.1	
Learners who screened positive for at least moderate anxiety by training level	Undergraduate	100	63 736	34.9	30.2	39.9	99.4	**0.079**
	Graduate	37	19 343	28.4	23.2	34.0	98.4	
Undergraduate learners who screened positive for at least moderate anxiety by year of studies	1st	13	1 551	25.9	19.7	32.5	86.6	0.967
	2nd	7	700	29.0	15.2	45.0	93.8	
	3rd	7	613	27.8	13.9	44.0	94.2	
	4th	6	428	21.4	8.8	37.5	91.8	
	5th	4	516	24.9	10.6	42.7	92.7	
Learners who screened positive for at least moderate anxiety by gender	Women	37	18 384	39.7	29.5	50.4	99.5	**0.038**
	Men	24	7 913	25.4	17.6	34.2	98.4	
Learners who screened positive for at least moderate anxiety by continent	North America	16	4 769	26.0	21.4	31.0	92.6	**0.002**
	South America	8	9 523	47.2	37.2	57.2	98.8	
	Europe	25	21 102	36.0	28.7	43.7	98.9	
	Asia	82	54 434	30.8	25.6	36.2	99.4	
	Africa	6	3 185	45.1	25.9	65.2	98.8	
Learners who screened positive for at least moderate anxiety by WHO region	American	23	13 977	32.4	25.9	39.4	98.5	**< 0.001**
	European	31	28 246	38.5	30.8	46.4	99.3	
	African	3	862	33.1	15.8	53.1	94.0	
	Eastern Mediterranean	43	17 824	40.4	34.1	46.8	98.7	
	South East Asian	20	6 759	26.6	20.2	33.6	97.4	
	Western Pacific	19	26 196	15.3	9.7	21.8	99.4	
Learners who screened positive for at least moderate anxiety by year of study end (2020 vs 2021)	2020	94	55 368	28.7	24.8	32.8	99.1	**0.001**
	2021	29	22 016	41.9	35.0	48.9	98.8	
Learners who screened positive for at least moderate depression by ISCO group	Medical doctors	84	66 013	30.2	25.2	35.4	99.5	0.370
	Nursing professionals	9	4 136	38.1	23.4	54.0	98.9	
	Dentists	10	2 735	29.0	20.3	38.6	96.2	
	Pharmacists	3	543	45.8	22.0	70.6	95.8	
	Physiotherapists	3	973	57.3	20.8	89.7	98.9	
Learners who screened positive for at least moderate depression by training level	Undergraduate	79	55 559	35.0	29.9	40.3	99.4	**0.098**
	Graduate	35	18 269	25.7	17.7	34.5	99.4	
	Continuing	3	911	21.6	8.3	39.0	94.5	
Undergraduate learners who screened positive for at least moderate depression by year of studies	1st	13	1 388	34.2	21.5	48.2	96.2	0.793
	2nd	8	483	25.6	9.1	46.5	95.3	
	3rd	10	876	33.2	21.5	46.0	93.2	
	4th	8	640	23.6	11.7	38.1	91.8	
	5th	6	891	30.6	18.6	44.1	93.3	
Learners who screened positive for at least moderate depression by gender	Women	37	18 520	42.6	32.7	52.8	99.5	0.179
	Men	26	7 246	32.5	22.4	43.4	98.8	
Learners who screened positive for at least moderate depression by continent	North America	14	3 779	22.2	16.1	28.9	95.0	**< 0.001**
	South America	7	8 473	53.8	41.9	65.5	99.0	
	Europe	24	19 836	33.0	26.9	39.3	98.3	
	Asia	64	43 118	30.9	24.6	37.5	99.5	
	Africa	8	6 868	45.5	35.9	55.4	98.0	
Learners who screened positive for at least moderate depression by WHO region	American	20	11 937	32.7	23.1	43.0	99.2	**< 0.001**
	European	31	25 235	35.9	26.5	45.9	99.5	
	Eastern Mediterranean	32	17 011	43.6	36.2	51.2	99.0	
	South East Asian	15	5 885	26.4	15.6	38.9	99.1	
	Western Pacific	19	22 606	14.9	12.0	18.1	97.4	
Learners who screened positive for at least moderate depression by year of study end (2020 vs 2021)	2020	79	54 615	29.4	24.8	34.2	99.3	0.141
	2021	26	21 266	36.8	28.8	45.2	99.1	
Learners who screened positive for burnout by ISCO group	Medical Doctors	61	34 465	39.0	33.4	44.9	99.0	0.375
	Dentists	3	218	51.6	25.4	77.3	93.0	
Learners who screened positive for burnout by training level	Undergraduate	18	14 171	36.0	27.3	45.1	98.8	0.712
	Graduate	50	17 891	38.9	32.3	45.7	98.7	
	Continuing	3	911	26.5	2.9	61.7	98.5	
Learners who screened positive for burnout by gender	Women	10	2 084	25.2	15.3	36.6	96.4	0.216
	Men	8	1 110	39.8	20.5	60.8	98.0	
Learners who screened positive for burnout by continent	North America	22	5 482	41.7	32.5	51.2	97.8	0.492
	South America	4	6 648	28.5	9.4	52.8	99.7	
	Europe	21	16 584	32.8	22.2	44.4	99.2	
	Asia	13	4 140	41.8	27.3	57.0	98.9	
Learners who screened positive for burnout by WHO region	American	27	12 241	40.8	32.6	49.2	98.7	0.574
	European	23	17 859	33.8	23.4	45.0	99.3	
	Eastern Mediterranean	8	1 822	38.6	19.9	59.2	98.6	
Learners who screened positive for burnout by year of study end (2020 vs 2021)	2020	41	16 743	37.3	30.1	44.7	98.9	0.149
	2021	13	16 401	46.8	36.4	57.4	98.9	
Learners who screened positive for insomnia by year of study end (2020 vs 2021)	2020	12	7 941	24.6	14.5	36.3	99.2	**0.023**
	2021	4	1 512	50.5	31.4	69.5	98.0	

Statistically significant differences (*p* < 0.05) or trends (*p* < 0.1) are noted in bold

*ISCO* International Standard Classification of Occupations, *n* number of studies, *N* number of participants

**Table 6:** Institutions enacting the responses implemented during the pandemic to preserve the education of health workers

Organization	Number of systematic review studies (phase 2 search)	Percentage of studies (%)
Educational institution (university/college)	291	58.8
Health care institution	118	23.8
National education/health care-related body/association	40	8.1
Education/health care-related body/association at a higher level than national	9	1.8
Government	25	5.1
Intergovernmental	0	0.0
World Health Organization	2	0.4
Educational institution (university/college) and health care provider/health care institution	1	0.2
Educational institution (university/college) and national education/health care-related body/association	2	0.4
Educational institution (university/college) and education/health care-related body/association at a higher level than national	1	0.2
Educational institution (university/college) and Government	3	0.6
Health care institution and national education/health care-related body/association	3	0.6
Total	495	100

**Table 7:** Satisfaction of health worker learners with educational methods implemented during the COVID-19 pandemic by subgroups

Explanation of outcome	Subgroup	*n*	*Ν*	Pooled proportion (%)	Lower confidence interval (%)	Higher confidence interval (%)	*I*^2^ (%)	*P*-value for subgroup difference
Overall learner satisfaction with online education by ISCO-group	Medical doctors	399	334 492	76.6	74.7	78.4	99.1	0.622
	Dentists	55	30 932	71.6	62.6	79.9	99.6	
	Nursing professionals	58	8 083	76.5	68.8	83.5	98.3	
	Pharmacists	27	4 175	74.8	62.3	85.6	98.4	
	Paramedical Practitioners	3	152	82.9	70.3	92.7	65.1	
	Physiotherapists	3	667	61.9	38.8	82.6	96.6	
Overall learner satisfaction with online education by level of training	Undergraduate	375	361 819	71.9	69.8	74.0	99.4	**< 0.001**
	Graduate	134	14 611	79.1	75.5	82.6	96.0	
	Continuing	26	6 173	86.8	82.0	91.0	95.3	
Overall undergraduate learner satisfaction with online education by year of studies	1st	49	7 592	79.3	72.1	85.7	98.0	0.155
	2nd	25	2 635	70.0	60.5	78.8	96.0	
	3rd	31	3 179	80.5	72.8	87.2	95.7	
	4th	34	3 923	82.6	73.8	90.0	97.5	
	5th	13	1 247	60.4	38.2	80.7	98.3	
	6th	6	787	71.2	48.1	89.8	97.4	
Overall learner satisfaction with online education by gender	Women	33	16 371	58.3	49.5	66.9	99.0	0.644
	Men	25	8 711	61.9	52.5	71.0	98.2	
Overall learner satisfaction with online education by continent	North America	186	16 631	84.8	81.7	87.7	96.1	**< 0.001**
	South America	17	14 213	75.9	65.1	85.4	98.9	
	Europe	112	41 416	81.2	76.4	85.5	99.1	
	Asia	235	308 861	64.0	61.1	66.9	99.5	
	Africa	16	11 075	79.5	65.2	91.0	99.6	
	Oceania	5	389	87.1	59.3	100.0	97.1	
Overall learner satisfaction with online education by WHO region	American	203	31 019	84.0	80.9	87.0	97.7	**< 0.001**
	European	127	61 616	78.8	74.4	82.9	99.3	
	African	10	2 680	86.1	70.4	96.7	98.5	
	Eastern Mediterranean	87	48 152	59.6	54.0	65.1	99.3	
	South East Asian	85	23 949	60.9	53.8	67.8	99.2	
	Western Pacific	60	238 209	78.5	74.2	82.5	99.7	
Overall learner satisfaction with online education by year of study end (2020 vs 2021)	2020	237	324 466	75.2	72.6	77.8	99.5	0.144
	2021	96	50 784	70.1	63.8	76.2	99.5	

Statistically significant differences (*p* < 0.05) or trends (*p* < 0.1) are noted in bold

*ISCO* International Standard Classification of Occupations, *n* number of studies, *N* number of participants

**Table 8:** Preference of health worker learners for the virtual-only educational format by subgroups

Explanation of outcome	Subgroup	*n*	*N*	Pooled proportion (%)	Lower confidence interval (%)	Higher confidence interval (%)	*I*^2^ (%)	*P*-value for subgroup difference
Learner preference for online education by ISCO group	Medical doctors	137	71 195	33.9	30.4	37.6	98.9	0.406
	Nursing professionals	11	2 461	30.2	21.1	40.3	95.7	
	Dentists	31	8 864	30.3	23.8	37.2	97.7	
	Pharmacists	9	1 858	20.6	6.3	40.1	98.8	
Learner preference for online education by level of training	Undergraduate	146	62 459	29.5	26.5	32.6	98.5	**0.007**
	Graduate	49	16 911	39.7	33.2	46.4	98.2	
	Continuing	8	3 369	39.9	27.7	52.7	97.4	
Undergraduate learner preference for online education by year of studies	1st	16	2 994	25.1	16.4	34.9	96.8	0.105
	2nd	13	1 233	32.6	20.7	45.7	95.4	
	3rd	8	499	13.6	6.9	22.1	82.7	
	4th	6	271	21.0	5.1	43.2	93.4	
	5th	4	300	16.7	3.5	36.4	93.1	
Learner preference for online education by gender	Women	10	2 095	36.1	20.7	53.1	98.3	0.550
	Men	7	1 177	43.9	25.0	63.8	97.7	
Learner preference for online education by continent	North America	44	14 744	40.1	34.1	46.3	97.2	**< 0.001**
	South America	3	1 402	12.3	5.7	20.8	87.7	
	Europe	41	15 191	38.7	32.4	45.2	98.3	
	Asia	105	40 620	28.0	24.0	32.1	98.8	
	Africa	9	1 454	31.7	16.1	49.7	98.0	
Learner preference for online education by WHO region	American	47	16 146	38.3	31.5	45.2	98.2	**< 0.001**
	European	49	30 492	37.3	32.7	42.1	98.2	
	African	7	1 102	29.7	11.5	51.9	98.3	
	Eastern Mediterranean	39	13 421	33.1	26.2	40.4	98.7	
	South East Asian	45	17 276	22.7	18.4	27.4	97.9	
	Western Pacific	18	8282	29.7	15.9	45.6	99.4	
Learner preference for online education by year of study end (2020 vs 2021)	2020	88	44 017	30.4	26.2	34.9	98.9	1.000
	2021	37	12 681	30.2	23.6	37.3	98.5	

Statistically significant differences (*p* < 0.05) or trends (*p* < 0.1) are noted in bold

*ISCO* International Standard Classification of Occupations, *n* number of studies, *N* number of participants

**Table 9:** Preference of health worker learners for the purely in-person educational format by subgroups

Explanation of outcome	Subgroup	*n*	*N*	Pooled proportion (%)	Lower confidence interval (%)	Higher confidence interval (%)	*I*^2^ (%)	*P*-value for subgroup difference
Learner preference for face-to-face education by ISCO group	Medical doctors	136	69 565	47.9	43.5	52.3	99.2	0.781
	Nursing professionals	9	1 470	57.4	41.4	72.6	96.7	
	Dentists	37	9 299	50.3	41.8	58.7	98.4	
	Pharmacists	12	2 229	49.0	40.2	57.8	93.6	
	Physiotherapists	3	424	30.5	0.1	90.2	99.4	
Learner preference for face-to-face education by training level	Undergraduate	159	70 146	50.9	46.9	54.9	99.1	**0.003**
	Graduate	47	8 217	47.6	39.9	55.4	97.8	
	Continuing	8	3 066	30.7	21.1	41.2	95.3	
Undergraduate learner preference for online education by year of studies	1st	22	3 750	59.6	47.3	71.2	98.1	0.616
	2nd	19	2 139	53.2	41.6	64.6	96.5	
	3rd	14	1 437	54.1	42.0	65.9	94.9	
	4th	7	698	46.3	31.5	61.3	92.7	
Learner preference for face-to-face education by gender	Women	6	2 212	37.6	23.5	52.8	98.0	0.882
	Men	3	1 075	40.4	9.9	75.8	99.2	
Learner preference for face-to-face education by continent	North America	54	7 043	49.3	42.2	56.3	96.8	**0.090**
	Europe	43	20 116	51.9	43.3	60.5	99.3	
	Asia	108	41 971	49.9	45.3	54.5	98.8	
	Africa	11	6 355	37.0	27.7	46.8	96.2	
Learner preference for face-to-face education by WHO region	American	56	8 313	50.1	43.0	57.1	97.3	**0.013**
	European	50	35 270	51.3	43.0	59.5	99.5	
	African	7	942	33.5	18.0	51.0	96.6	
	Eastern Mediterranean	43	20 353	46.5	40.2	52.9	98.7	
	South East Asian	50	17 702	56.5	49.1	63.7	99.0	
	Western Pacific	15	7 483	32.9	21.7	45.2	98.6	
Learner preference for face-to-face education by year of study end (2020 vs 2021)	2020	94	48 758	46.8	41.8	51.8	99.2	0.540
	2021	48	20 905	49.6	43.2	56.0	98.8	

Statistically significant differences (*p* < 0.05) or trends (*p* < 0.1) are noted in bold

*ISCO* International Standard Classification of Occupations, *n* number of studies, *N* number of participants

**Table 10:** Preference of health worker learners for the blended educational format by subgroups

Explanation of outcome	Subgroup	*n*	*N*	Pooled proportion (%)	Lower confidence interval (%)	Higher confidence interval (%)	*I*^2^ (%)	*P*-value for subgroup difference
Learner preference for blended education by ISCO group	Medical doctors	38	7 994	52.9	46.1	59.6	97.1	0.182
	Dentists	8	2 636	65.8	56.3	74.8	95.0	
	Nursing Professionals	4	586	62.7	29.0	90.8	97.6	
	Pharmacists	4	1 313	57.5	46.7	67.9	92.1	
Learner preference for blended education by training level	Undergraduate	48	11 505	57.3	51.8	62.6	97.0	0.690
	Graduate	10	2 131	59.8	46.2	72.8	97.3	
Learner preference for blended education by continent	North America	12	1 397	51.3	36.7	65.8	95.8	**0.073**
	Europe	9	1 496	69.3	59.2	78.5	93.0	
	Asia	36	10 694	54.0	47.8	60.2	97.6	
	Africa	5	789	58.8	36.5	79.3	97.4	
Learner preference for blended education by WHO region	American	12	1 397	51.3	36.7	65.8	95.8	0.184
	European	13	3 389	64.7	54.8	73.9	96.7	
	African	3	413	70.3	36.7	95.0	97.7	
	Eastern Mediterranean	18	4 188	50.6	41.6	59.5	97.0	
	South East Asian	12	3 640	49.8	38.7	60.9	97.7	
	Western Pacific	6	1 470	64.6	47.6	79.8	96.7	
Learner preference for blended education by year of study end (2020 vs 2021)	2020	18	5 009	50.0	42.1	57.8	96.6	0.214
	2021	14	4 492	58.0	48.3	67.5	97.7	

Statistically significant differences (*p* < 0.05) or trends (*p* < 0.1) are noted in bold

*ISCO* International Standard Classification of Occupations, *n* number of studies, *N* number of participants

**Table 11:** Learners supporting the adoption of a blended format in the post-pandemic future of health worker education by subgroups

Explanation of outcome	Subgroup	*n*	*N*	Pooled proportion (%)	Lower confidence interval (%)	Higher confidence interval (%)	*I*^2^ (%)	*P*-value for subgroup difference
Learners wanting to keep blended education post-pandemic by ISCO group	Medical doctors	103	35 649	70.9	66.7	74.8	98.4	0.107
	Dentists	14	4 090	62.5	46.4	77.3	99.0	
	Pharmacists	3	992	52.2	33.3	70.8	96.6	
Learners wanting to keep blended education post-pandemic by training level	Undergraduate	84	37 525	63.9	60.2	67.6	98.1	0.176
	Graduate	39	4 517	72.2	64.6	79.3	96.4	
	Continuing	3	147	64.9	27.6	94.1	93.8	
Undergraduate learners wanting to keep blended education post-pandemic by year of studies	1st	11	1 618	64.8	50.0	78.4	96.9	0.265
	2nd	6	556	59.6	38.8	78.9	95.4	
	3rd	8	625	69.6	50.6	85.9	94.9	
	4th	5	352	68.2	34.8	93.8	97.1	
	6th	3	311	78.9	70.6	86.3	64.2	
Learners wanting to keep blended education post-pandemic by gender	Women	7	1 231	67.4	51.5	81.5	96.7	0.741
	Men	4	343	61.1	26.3	90.7	97.3	
Learners wanting to keep blended education post-pandemic by continent	North America	38	5 055	75.7	64.4	85.6	98.6	**< 0.001**
	Europe	33	7 795	76.0	70.1	81.4	96.5	
	Asia	57	30 660	56.8	52.0	61.5	98.4	
	Africa	4	573	76.7	67.7	84.6	75.2	
Learners wanting to keep blended education post-pandemic by WHO region	American	40	5 195	75.7	64.8	85.2	98.5	**< 0.001**
	European	35	8 182	74.8	68.6	80.6	97.0	
	African	3	813	76.5	52.4	94.1	94.6	
	Eastern Mediterranean	18	9 489	55.8	46.2	65.2	98.8	
	South East Asian	27	7 037	56.7	49.0	64.2	97.6	
	Western Pacific	11	13 507	62.2	55.6	68.6	97.2	

Statistically significant differences (*p* < 0.05) or trends (*p* < 0.1) are noted in bold

*ISCO* International Standard Classification of Occupations, *n* number of studies, *N* number of participants

**Table 12:** Learners supporting the adoption of a virtual-only format in the post-pandemic future of health worker education by subgroups

Explanation of outcome	Subgroup	*n*	*N*	Pooled proportion (%)	Lower confidence interval (%)	Higher confidence interval (%)	*I*^2^ (%)	*P*-value for subgroup difference
Learners wanting to keep online education post-pandemic by continent	North America	33	4 693	40.7	30.6	51.3	98.0	**0.004**
	Europe	20	3 400	36.2	24.0	49.5	98.3	
	Asia	57	31 627	28.2	23.3	33.5	98.9	
	Africa	6	1 292	62.9	41.9	81.7	98.2	
Learners wishing to keep online education post-pandemic by WHO region	American	33	4 693	40.7	30.6	51.3	98.0	0.338
	European	25	17 118	35.7	24.9	47.3	99.0	
	African	5	1 414	49.5	25.0	74.2	98.7	
	Eastern Mediterranean	21	9 963	33.8	25.2	42.9	98.8	
	South East Asian	22	6 941	29.0	21.6	37.0	97.8	
	Western Pacific	13	14 227	28.6	18.0	40.4	99.3	

Statistically significant differences (*p* < 0.05) or trends (*p* < 0.1) are noted in bold

*n* number of studies, *N* number of participants

**Table 13:** Satisfaction of learners with virtual assessment methods during the COVID-19 pandemic by subgroups

Explanation of outcome	Subgroup	*n*	*N*	Pooled proportion (%)	Lower confidence interval (%)	Higher confidence interval (%)	*I*^2^ (%)	*P*-value for subgroup difference
Learner satisfaction with online assessment by ISCO group	Medical doctors	34	7 261	73.5	62.7	83.1	98.8	0.436
	Nursing professionals	4	1 249	65.8	30.0	93.8	99.3	
	Dentists	9	1 482	61.6	50.5	72.2	94.2	
	Pharmacists	6	550	58.9	31.9	83.4	97.2	
Learner satisfaction with online assessment by training level	Undergraduate	37	9 221	62.5	52.4	72.1	98.9	**< 0.001**
	Graduate	13	726	86.6	78.1	93.3	86.5	
Learner satisfaction with online assessment by gender	Women	4	803	38.7	32.6	45.0	66.0	**0.075**
	Men	3	305	58.1	37.7	77.3	92.1	
Learner satisfaction with online assessment by continent	North America	13	1 489	82.9	69.9	92.9	96.4	**< 0.001**
	Europe	7	632	87.3	82.1	91.8	65.9	
	Asia	29	7 930	53.1	43.4	62.7	98.5	
	Africa	3	903	82.1	46.3	100.0	98.9	
Learner satisfaction with online assessment by WHO region	American	14	1 589	82.3	70.3	91.8	96.1	**< 0.001**
	European	7	632	87.3	82.1	91.8	65.9	
	Eastern Mediterranean	12	5 355	61.4	41.1	79.9	99.5	
	South East Asian	15	2 449	52.7	37.5	67.5	98.2	
	Western Pacific	4	882	55.0	31.3	77.5	97.6	

Statistically significant differences (*p* < 0.05) or trends (*p* < 0.1) are noted in bold

*ISCO* International Standard Classification of Occupations, *n* number of studies, *N* number of participants

**Table 14:** Learners’ willingness to volunteer and actual participation in pandemic-related-volunteering activities due to the COVID-19 pandemic by subgroups

Explanation of outcome	Subgroup	*n*	*N*	Pooled proportion (%)	Lower confidence interval (%)	Higher confidence interval (%)	*I*^2^ (%)	*P*-value for subgroup difference
Learners who volunteered by training level	Undergraduate	17	32 541	32.4	20.6	45.4	99.8	**0.029**
	Postgraduate	6	2 059	9.1	0.4	26.2	99.0	
Learners who volunteered by continent	North America	4	3 270	32.8	10.3	60.6	99.6	0.206
	Europe	15	10 328	31.4	19.6	44.5	99.5	
	Asia	4	8 320	17.1	7.9	28.9	98.9	
	European	16	23 368	29.3	17.2	43.2	99.7	
	Eastern Mediterranean	3	9 393	39.5	18.0	63.3	99.7	
Learners who volunteered by WHO region	American	5	3 316	25.3	7.1	49.8	99.5	0.672
	European	16	23 368	29.3	17.2	43.2	99.7	
	Eastern Mediterranean	3	9 393	39.5	18.0	63.3	99.7	
Learners who wanted to volunteer by training level	Undergraduate	21	26 890	61.2	46.4	75.1	99.8	0.187
	Postgraduate	3	939	72.7	63.2	81.2	85.5	
Learners who wanted to volunteer by continent	North America	5	2 040	68.3	49.4	84.6	98.6	0.201
	Europe	6	3 701	43.3	17.0	71.8	99.6	
	Asia	13	11 794	71.3	61.0	80.5	99.2	
Learners who wanted to volunteer by WHO region	American	6	12 473	59.0	27.8	86.6	99.8	**0.015**
	European	7	3 941	47.4	21.6	74.0	99.6	
	Eastern Mediterranean	3	2 018	60.4	55.8	64.8	73.4	
	South East Asian	6	6 648	69.4	47.1	87.8	99.5	
	Western Pacific	3	2 888	83.7	71.0	93.3	97.9	

Statistically significant differences (*p* < 0.05) or trends (*p* < 0.1) are noted in bold

*n* number of studies, *N* number of participants

**Table 15:** Summary and interpretation of main results

Outcome	Analysis	Meta-analysis results	Conclusions–interpretations
COVID-19 impacts			
Perceived training disruption of learners	Overall	71.1% (67.9–74.2), *I*^2^ = 98.7%, *N* = 66 870	A considerable rate of learners likely perceived some extent of disruption of training amidst the pandemic
	Invasive vs non-invasive experience	Invasive: 75.8% (71.4–79.9), *I*^2^ = 98.2%, *N* = 23 047; non-invasive: 69.7% (64.4–74.8), *I*^2^ = 98.7%, *N* = 25 463	Learner perceived disruption of training was high in terms of both invasive procedures and non-invasive clinical experience, though the former was more prominent
	By WHO region	AMR: 67.1% (61.3–72.8), *I*^2^ = 97.9%, *N* = 13 430 vs EUR: 71.1% (65.9–76.0), *I*^2^ = 97.8%, *N* = 15 249 vs EMR: 71.6% (60.7–81.3), *I*^2^ = 99.3%, *N* = 12 019 vs SEAR: 84.5% (80.3–88.4), *I*^2^ = 95.3%, *N* = 7 809 vs WPR: 69.9% (60.2–78.8), *I*^2^ = 97.0%, *N* = 3 964; *p*_subgroup_ < 0.001	The highest learner rate perceiving training disruption was recorded in the SEAR. These rates may be examined in combination with the satisfaction and preference rates for online learning methods. However, the disruption should be considered multifactorial (e.g., redeployment, decrease of case numbers, etc.) and dissatisfaction with virtual delivery of education may just be one of the contributing factors
Learner redeployment	Overall	29.2% (25.3–33.2), *I*^2^ = 95.3%, *N* = 11 527	Approximately 3 out of 10 learners might have been redeployed due to the pandemic
	By WHO region	AMR: 24.7% (19.5–30.3), *I*^2^ = 94.4%, *N* = 4 838 vs EUR: 35.2% (28.8–41.8), *I*^2^ = 94.6%, *N* = 4 156 vs AFR: 40.7% (10.2–75.8), *I*^2^ = 97.0%, *N* = 276 vs EMR: 25.9% (9.5–46.6), *I*^2^ = 96.5%, *N* = 648 vs SEAR: 13.7% (0.1–43.8), *I*^2^ = 97.7%, *N* = 420; *p*_subgroup_ = 0.092	When compared with their colleagues in the AMR, learners in the EUR likely exhibited higher redeployment rates due to the pandemic
Learners rethinking career plans	Overall (and sensitivity analysis)	21.5% (17.1–26.3), *I*^2^ = 99.5%, *N* = 134 623; [21.8% (17.2–26.8), *I*^2^ = 99.1%, *N* = 35 955 after exclusion of studies with *N* > 25 000 to minimize risk of duplicate population]	A considerable rate of learners reconsidered their career plans (residency/practice/expertise) due to the COVID-19 pandemic
At least moderate scaled learner anxiety	Overall	32.3% (28.5–36.2), *I*^2^ = 99.4%, *N* = 95 927	Amidst the COVID-19 pandemic, approximately one-third of learners might have screened positive for anxiety of at least moderate severity
	GAD-7 only (and sensitivity analysis)	32.1% (26.6–37.9), *I*^2^ = 99.5%, *N* = 53 658 (low risk of bias studies only: 32.2% (26.0–38.7), *I*^2^ = 99.5%, *N* = 45 382)	Learner rates of at least moderate anxiety did not materially change when only studies that used the GAD-7 screening tool (and their low-risk of bias-subset) were analyzed
	By ISCO-08 HW group	Medical doctors: 30.4% (25.6–35.3), *I*^2^ = 99.5%, *N* = 76 730 vs nursing professionals: 33.0% (20.1–47.4), *I*^2^ = 98.5%, *N* = 3 196 vs dentists: 32.4% (25.4–39.7), *I*^2^ = 96.3%, *N* = 4 812 vs Pharmacists: 50.0% (45.6–54.5), *I*^2^ = 19.1%, *N* = 643; *p*_subgroup_ < 0.001	Pharmacy learners might have screened positive for at least moderate anxiety at significantly higher rates than the other occupational groups. Anxiety is likely multifactorial and, therefore, reasons leading to higher anxiety in this occupational group might have not been investigated in this paper
	By training level	Undergraduate: 34.9% (30.2–39.9), *I*^2^ = 99.4%, *N* = 63 736 vs postgraduate: 28.4% (23.2–34.0), *I*^2^ = 98.4%, *N* = 19 343; *p*_subgroup_ = 0.079	Although anxiety is multifactorial, higher anxiety observed in undergraduate learners could be partially attributed to their lower satisfaction with online learning compared to their postgraduate counterparts
	By WHO region	AMR: 32.4% (25.9–39.4), *I*^2^ = 98.5%, *N* = 13 977 vs EUR: 38.5% (30.8–46.4), *I*^2^ = 99.3%, *N* = 28 246 vs AFR: 33.1% (15.8–53.1), *I*^2^ = 94.0%, *N* = 862 vs EMR: 40.4% (34.1–46.8), *I*^2^ = 98.7%, *N* = 17 824 vs SEAR: 26.6% (20.2–33.6), *I*^2^ = 97.4%, *N* = 6 759 vs WPR: 15.3% (9.7–21.8), *I*^2^ = 99.4%, *N* = 26 196; *p*_subgroup_ < 0.001	Learners in the WPR might have screened positive for anxiety of at least moderate severity at significantly lower rates compared to their counterparts in the other regions. Learner anxiety rates may have also been lower in the SEAR compared to the EMR and EUR. The continent analysis further showed significantly higher anxiety rates in South compared to North America (and Asia). This difference could not have been revealed by the WHO regional analysis. Combined interpretation of these analyses is therefore essential (Table 5)
	By gender	Female: 39.8% (29.5–50.4), *I*^2^ = 99.5%, *N* = 18 384 vs male: 25.4% (17.6–34.2), *I*^2^ = 98.4%, *N* = 7 913; *p*_subgroup_ = 0.038	In line with the relevant literature, female gender may have been associated with increased anxiety rates
	By year of study end date (2020 vs 2021)	2020: 28.7% (24.8–32.8), *I*^2^ = 99.1%, *N* = 55 368 vs 2021: 41.9% (35.0–48.9), *I*^2^ = 98.8%, *N* = 22 016; *p*_subgroup_ = 0.001	Learner rates of at least moderate anxiety may have been higher in 2021 compared to 2020, reflecting potential accumulation as pandemic continued to evolve. This finding could indicate that policies for prevention of learners’ anxiety should have been implemented early during the pandemic
At least moderate scaled learner depression	Overall	32.0% (27.9–36.2), *I*^2^ = 99.4%, *N* = 84 067	Amidst the COVID-19 pandemic, approximately one-third of learners screened positive for depression of at least moderate severity
	PHQ-9 only (and sensitivity analysis)	32.8% (25.3–40.7), *I*^2^ = 99.6%, *N* = 39 876 (low risk of bias studies only: 31.0% (23.0–40.0), *I*^2^ = 99.6%, *N* = 32 803)	Learner rates of at least moderate depression did not materially change when only studies that used the PHQ-9 screening tool (and their low-risk of bias-subset) were analyzed
	By training level	Undergraduate: 35.0% (29.9–40.3), *I*^2^ = 99.4%, *N* = 55 559 vs Postgraduate: 25.7% (17.7–34.5), *I*^2^ = 99.4%, *N* = 18 269 vs continuing: 21.6% (8.3–39.0), *I*^2^ = 94.5%, *N* = 911; *p*_subgroup_ = 0.098	As with anxiety, undergraduate learners may have screened positive for depression of at least moderate severity at higher rates than their postgraduate counterparts
	By WHO region	AMR: 32.7% (23.1–43.0), *I*^2^ = 99.2%, *N* = 11 937 vs EUR: 35.9% (26.5–45.9), *I*^2^ = 99.5%, *N* = 25 235 vs EMR: 43.6% (36.2–51.2), *I*^2^ = 99.0%, *N* = 17 011 vs SEAR: 26.4% (15.6–38.9), *I*^2^ = 99.1%, *N* = 5 885 vs WPR: 14.9% (12.0–18.1), *I*^2^ = 97.4%, *N* = 22 606; *p*_subgroup_ < 0.001	Learners in the WPR might have screened positive for depression of at least moderate severity at significantly lower rates compared to their counterparts in the other regions (especially AMR, EUR, EMR). Regional differences in anxiety and depression rates of at least moderate severity might follow a similar pattern, with the highest rates being observed in the EMR, followed by the EUR, AMR, SEAR and WPR. However, some of these differences may be due to chance alone. As with anxiety, significantly higher depression rates were found by studies in South America when compared with studies conducted in the other continents (Table 5)
Learner scaled burnout	Overall	38.8% (33.4–44.3), *I*^2^ = 99.0%, *N* = 35 808	Almost 4 out of 10 learners might have screened positive for burnout syndrome amidst the pandemic
	MBI and variants only (and sensitivity analysis)	46.8% (38.6–55.1), *I*^2^ = 98.4%, *N* = 17 134 (low risk of bias studies only: 43.5% (35.3–51.9), *I*^2^ = 98.4%, *N* = 16 964)	Studies using the MBI and its variants revealed higher learner burnout rates. This may be a more accurate estimation of learner burnout rates or an overestimation due to potentially higher false-positive rates observed when using certain MBI variants
Learner scaled insomnia	Overall	30.9% (20.8–41.9), *I*^2^ = 99.2%, *N* = 9 906	Almost one-third of learners might have screened positive for insomnia amidst the pandemic. Combining the findings on anxiety, depression, burnout, and insomnia (all as per measurements with validated scales) it appears that HW learners may be considered as a vulnerable group for “mental health disruption”, as they are simultaneously faced with two distinct and equally challenging tasks, namely education and patient care
	By year of study end date (2020 vs 2021)	2020: 24.6% (14.5–36.3), *I*^2^ = 99.2%, *N* = 7 941 vs 2021: 50.5% (31.4–69.5), *I*^2^ = 98.0%, *N* = 1 512; *p*_subgroup_ = 0.023	As with anxiety, learner rates of insomnia may have been higher in 2021 compared to 2020, reflecting potential accumulation as pandemic continued to evolve. This finding could indicate that policies for prevention of learners’ insomnia should have been implemented early during the pandemic
Outcomes of policies			
Satisfaction with online	Learner (and sensitivity analysis) vs faculty	Learner: 75.9% (74.2–77.7), *I*^2^ = 99.3%, *N* = 425 466 [76.2% (74.0–78.3), *I*^2^ = 99.2%, *N* = 226 348 after exclusion of studies with *N* > 25 000 to minimize risk of duplicate population]; faculty: 71.8% (66.7–76.7), *I*^2^ = 93.9%, *N* = 6 525	HW learners and faculty might have been generally satisfied with online learning methods during the pandemic, with faculty appearing somewhat less satisfied than learners. A potential explanation could be that faculty may have encountered the extra challenge of attempting to engage their audiences
Learner satisfaction with online learning	Theoretical vs practical vs clinical experience (and sensitivity analyses)	Theoretical: 67.5% (64.7–70.3), *I*^2^ = 99.5%, *N* = 252 931 (67.6% (64.4–70.7), *I*^2^ = 99.4%, *N* = 153 372 after exclusion of studies with *N* > 25 000 to minimize risk of duplicate population); Practical: 85.4%, (82.3–88.2), *I*^2^ = 99.2%, *N* = 153 445 [85.5% (82.5–88.2), *I*^2^ = 98.6%, *N* = 53 886 after exclusion of studies with *N* > 25 000 to minimize risk of duplicate population]; clinical experience: 86.9% (79.5–93.1), *I*^2^ = 98.5%, *N* = 8 640	During the pandemic, HW learners might have been more satisfied with online practical courses and online true clinical experience involving patients than with predominantly theoretical online courses. When lack of interaction/practice was addressed to the possible extent, satisfaction seemed to increase
	By training level	Undergraduate: 71.9% (69.8–74.0), *I*^2^ = 99.4%, *N* = 361 819 vs postgraduate: 79.1% (75.4–82.5), *I*^2^ = 96.0%, *N* = 14 611 vs continuing: 86.8% (82.0–91.0), *I*^2^ = 95.3%, *N* = 6 173; *p*_subgroup_ < 0.001	Satisfaction with online learning seemed to significantly increase as training level increased. Accessibility and flexibility of this format may have better suited the likely busier schedules of learners at higher training stage
	By WHO region	AMR: 84.0% (80.9–87.0), *I*^2^ = 97.7%, *N* = 31 019 vs EUR: 78.8% (74.4–82.9), *I*^2^ = 99.3%, *N* = 61 616 vs AFR: 86.1% (70.4–96.7), *I*^2^ = 98.5%, *N* = 2 680 vs EMR: 59.6% (53.9–65.1), *I*^2^ = 99.3%, *N* = 48 152 vs SEAR: 60.9% (53.8–67.8), *I*^2^ = 99.2%, *N* = 23 949 vs WPR: 78.5% (74.2–82.4), *I*^2^ = 99.7%, *N* = 238 209; *p*_subgroup_ < 0.001	Learner satisfaction with virtual learning methods might have been lower in the EMR and SEAR when compared to that in the AMR, EUR, AFR and WPR. Lower satisfaction might be attributed to lower availability of resources, potential connectivity issues or difficulty in accessing necessary equipment in these regions. Learners in the AFR might have experienced accessibility or other issues with the in-person format even before the onset of the pandemic. The need to bypass such issues may have reinforced their satisfaction with online options
Learner preference for learning method	Online vs face-to-face vs blended	Online: 32.0% (29.3–34.8), *I*^2^ = 98.7%, *N* = 94 452; face-to-face: 48.8% (45.4–52.1), *I*^2^ = 99.0%, *N* = 97 903; blended: 56.0% (51.2–60.7), *I*^2^ = 96.9%, *N* = 14 992	Learners seemed to prefer the existence of an in-person component in their curriculum. The virtual component was potentially preferred as part of a blended educational system rather than a purely distant format
	By training level	Undergraduate: 29.5% (26.5–32.6), *I*^2^ = 98.5%, *N* = 62 459 vs postgraduate: 39.7% (33.2–46.4), *I*^2^ = 98.2, *N* = 16 911 vs continuing: 39.9% (27.7–52.7), *I*^2^ = 97.4%, *N* = 3 369; *p*_subgroup_ = 0.007	Postgraduate learners likely preferred the virtual format significantly more than their undergraduate counterparts. This is in accordance with findings on satisfaction. Accessibility and flexibility of this format may have better suited their likely busier schedules
	By WHO region	AMR: 38.3% (31.5–45.2), *I*^2^ = 98.2%, *N* = 16 146 vs EUR: 37.3% (32.7–42.1), *I*^2^ = 98.2%, *N* = 30 492 vs AFR: 29.7% (11.5–51.9), *I*^2^ = 98.3%, *N* = 1 102 vs EMR: 33.1% (26.2–40.4), *I*^2^ = 98.7%, *N* = 13 421 vs SEAR: 22.7% (18.4–27.3), *I*^2^ = 97.9%, *N* = 17 276 vs WPR: 29.7% (15.9–45.6), *I*^2^ = 99.4%, *N* = 8 282; *p*_subgroup_ < 0.001	Preference for the purely virtual format appeared to be lower for learners in the SEAR when compared to their counterparts in the AMR, EUR and EMR. Focusing on the comparison of the SEAR and the EMR, and combining the results with those of satisfaction per WHO region, it is likely that the lower satisfaction with the virtual courses in the EMR region may have resulted more from issues emerging during their delivery rather than the virtual format itself. The same might not apply for countries of the SEAR, in which learners may have perceived the virtual-only format as less feasible, regardless of how well the courses were actually delivered
Learner preference for face-to-face learning	By training level	Undergraduate: 50.9% (46.9–54.9), *I*^2^ = 99.1%, *N* = 70 146 vs Postgraduate: 47.6% (39.9–55.4), *I*^2^ = 97.8%, *N* = 8 217 vs continuing: 30.7% (21.1–41.2), *I*^2^ = 95.3%, *N* = 3 066; *p*_subgroup_ = 0.003	In accordance with preference for the virtual format, preference for the in-person educational format might have been significantly higher for undergraduate learners than their counterparts at senior training stage. However, preference rates for in-person learning were likely higher than those for virtual training for learners of all levels
Learners wanting to keep learning method post-pandemic	Online-only vs blended	Online: 34.7% (30.7–38.8), *I*^2^ = 99.0%, *N* = 59 765; blended: 68.1% (64.6–71.5), *I*^2^ = 98.4%, *N* = 49 585	Learners were likely in favor of maintaining the virtual format post-pandemic along with their in-person curricular activities rather than maintaining it on its own
Learners wanting to keep blended learning post-pandemic	By WHO region	AMR: 75.7% (64.8–85.2), *I*^2^ = 98.5%, *N* = 5 195 vs EUR: 74.8% (68.6–80.6), *I*^2^ = 97.0%, *N* = 8 182 vs AFR: 76.5% (52.4–94.1), *I*^2^ = 94.6%, *N* = 813 vs EMR: 55.8% (46.2–65.2), *I*^2^ = 98.8%, *N* = 9 489 vs SEAR: 56.7% (48.9–64.2), *I*^2^ = 97.6%, *N* = 7 037 vs WPR: 62.2% (55.6–68.6), *I*^2^ = 97.2%, *N* = 13 507; *p*_subgroup_ < 0.001	As more learners have likely expressed the desire to maintain the virtual format as part of a blended system, rates of learners in favor of a future blended system were generally in accordance with the rates of satisfaction with online methods, except for the WPR. The lower-than-expected rates in the WPR might be attributed to saturation with the virtual format (even as part of a blended system and despite the potentially high quality of its delivery), considering that the pandemic struck this region first and transition to the virtual format might have occurred there first
Effectiveness of learning methods	Comparator vs intervention	SMD = − 1.09 (− 1.21 to -0.96), *I*^2^ = 98.2%, *N* = 49 911 [SMD = − 1.11 (− 1.25 to − 0.96), *I*^2^ = 97.9%, *N* = 24 432 after exclusion of studies with *N* > 25 000 to minimize risk of duplicate population] Pre vs Post-intervention (phase 2): SMD = − 1.31 (− 1.46 to − 1.16), *I*^2^ = 98.1%, *N* = 42 060 Comparator (previous method) vs intervention SMD = − 0.28 (− 0.48 to − 0.09), *I*^2^ = 94.3%, *N* = 4 489	Learning methods applied during the pandemic seemed overall effective as they likely managed to significantly improve learners’ mean knowledge or acquired overall skills’ scores compared to pre-training status or the respective pre-pandemic methods. A main limitation of these studies is that they are based on evaluations right after the intervention without long-term follow-up. That often leads to overvalued effectiveness of the interventions. That is more evident in the studies comparing knowledge/skills’ scores before and after the intervention
Learner satisfaction with pandemic face-to-face learning	Overall	93.0% (89.1–96.2), *I*^2^ = 95.4%, *N* = 6 263	Learner satisfaction with the in-person learning activities that were employed during the pandemic, was likely high (probably even higher than that with online activities). Learners might have been that satisfied either due to the in-person format inside a curriculum full of virtual activities or because of the COVID-19-related character of many of these activities, with the latter potentially providing them with essential knowledge/skills to deal with this pandemic
Learner satisfaction with online assessment	Overall	68.8% (60.7–76.3), *I*^2^ = 98.6%, *N* = 11 072	Learner satisfaction with virtual evaluation methods was likely moderate to high, probably reflecting a balance between convenience or better scores and potential cheating or perception of unfairness
	By training level	Undergraduate: 62.5% (52.4–72.1), *I*^2^ = 98.9%, *N* = 9 221 vs postgraduate: 86.6% (78.1–93.3), *I*^2^ = 86.5%, *N* = 726; *p*_subgroup_ < 0.001	Satisfaction with online evaluation might have been significantly higher for postgraduate learners compared to undergraduates. Postgraduate learners may have perceived the distant format as more flexible or even easier to prepare for, which are essential advantages, especially in the context of a likely busier schedule
	By WHO region	AMR: 82.3% (70.3–91.8), *I*^2^ = 96.1%, *N* = 1 589 vs EUR: 87.3% (82.1–91.8), *I*^2^ = 65.9%, *N* = 632 vs EMR: 61.4% (41.0–79.9), *I*^2^ = 99.5%, *N* = 5 355 vs SEAR: 52.7% (37.5–67.5), *I*^2^ = 98.2%, *N* = 2 449 vs WPR: 55.0% (31.3–77.5), *N* = 882; *p*_subgroup_ < 0.001	Exactly as with training methods, learner satisfaction rates with virtual assessment might have been lower in the EMR and SEAR when compared to those in the AMR and EUR (data on WPR are limited and less credible). This reinforces the robustness of this review’s findings on regional differences in satisfaction rates and indicates that satisfaction may represent more the learners’ views on the distant format of the innovations rather than their primary aim (i.e., training or assessment). However, data on virtual innovations for assessment are far more limited than that focusing on virtual responses for education
Learner online vs face-to-face assessment scores	Previous/in-person vs virtual/new method	SMD = − 0.68 (− 0.96 to − 0.40), *I*^2^ = 98.1%, *N* = 12 513	Learners likely achieved significantly higher scores when undertaking online assessment compared to pre-pandemic in-person evaluation methods. This finding may be attributed to easier examination formats, lower examination demands, given the circumstances, or inadequate supervision of participants
Learners’ actual participation in volunteering activities	Overall	27.7% (19.1–37.3), *I*^2^ = 99.7%, *N* = 39 046	An encouraging rate of learners might have volunteered during the pandemic
	By training level	Undergraduate: 32.4% (20.6–45.4), *I*^2^ = 99.8%, *N* = 32 541 vs postgraduate: 9.1% (0.4–26.2), *I*^2^ = 99.0%, *N* = 2 059; *p*_subgroup_ = 0.029	Undergraduate learners might have volunteered at higher rates than their graduate counterparts. This finding may be attributed more to the availability of time of undergraduates rather than differences in willingness to volunteer
Learners’ intention to volunteer	Overall	62.2% (49.2–74.4), *I*^2^ = 99.8%, *N* = 28 728	A considerable rate of learners might have intended to volunteer during the pandemic, consisting of a valuable pool of available volunteers willing to assist, if needed

WHO regions: AMR, Region of the Americas; EUR, Region of Europe; AFR, Region of Africa; EMR, Eastern Mediterranean Region; SEAR, South East Asian Region; WPR, Western Pacific Region). All numbers in parentheses refer to the 95% confidence interval of the respective metric

*ISCO* International Standard Classification of Occupations, *n* number of studies, *N* number of participants, *SMD* standardized mean difference

### Impact of the pandemic on health worker education

The widespread disruption in undergraduate, graduate and continuing education of health workers due to closures and physical distancing has been clearly reported since the start of the pandemic [5]. There were references to complete or temporary cessation of in-person educational activities including classes and patient contact [29, 30]; and in many cases the temporary cessation of face-to-face learning, both pre-clinical and clinical. Especially for undergraduate learners, bedside education was initially halted to protect learners [31]. During residency training, the main disruptions identified were the reduction in case volumes [32, 33] especially in surgical training [34, 35], less time available for learners to spend in the hospital [36], or, conversely, increased workload, especially in COVID-related specialties. Other activities including in-person scientific conferences were discontinued [37]. Timely graduation was jeopardized [38], required examinations were canceled [39] and graduates were unable to apply for their next steps [40].

#### Disruption to clinical training

Most studies surveying training disruption focused on learners in a clinical setting. Overall, self-perceived disruption of training during the pandemic was estimated at 71.1% (95% confidence interval: 67.9–74.2) and varied according to WHO region, with the highest disruption having been observed in the Southeast Asia Region (SEAR) (Table 3). When surveyed, 75.8% (71.4–79.9) of learners noted decreased exposure to invasive procedures, such as surgeries or endoscopies, whereas a somewhat lower disruption was observed for the outpatient or inpatient clinical activity and performance in non-invasive procedures (69.7%, 64.4–74.9). Due to the disruption, 44.7% (39.2–50.2) of learners would want to prolong their training to presumably cover their educational gaps.

#### Disruption of career plans

Learners were sometimes redeployed from their training programs to support the COVID-19 response [41–43]. An estimated 29.2% (25.3–33.2) of clinical learners had to be redeployed during the pandemic to fulfill new roles, either caring for COVID-19 patients or accommodating other clinical needs associated with the response to the pandemic (e.g., covering a non-COVID-19 unit because of health worker shortage). This was more evident for learners in the WHO European region (EUR) (35.2%, 28.8–41.8), compared to those in Regions of the Americas (AMR, 24.7%, 19.5–30.3) (Table 4). Also, 21.5% (16.9–26.1) of learners admitted that they were reevaluating their future career plans due to the pandemic.

#### Mental health of learners: anxiety, depression, burnout, and insomnia

At least moderate anxiety, measured by validated scales, was estimated at 32.3% (28.4–36.1%). Notably, pharmacy learners reported higher anxiety than any other occupation, undergraduate learners scored higher than graduate ones, female learners scored higher than males, and learners in the WPR scored lower than any other WHO region. Also, learners surveyed in 2021 showed higher anxiety rates than learners in 2020 (Table 5).

Based on validated instruments, at least moderate depression was prevalent in 32.0% of learners (27.8–36.2), with undergraduates showing higher rates than graduate learners, learners in South America and Africa showing higher rates than other continents, and learners in the WPR showing lower rates than any other WHO region (Table 5). Further sensitivity analyses on studies using GAD-7 or PHQ-9 revealed similar findings (32.1% for anxiety, 32.8% for depression). Pooled mean GAD-7 and PHQ-9 learner scores were 7.00 (6.22–7.79), and 6.83 (5.72–7.95), respectively.

Burnout was prevalent in 38.8% of learners (33.4–44.2), with sensitivity analysis restricted to MBI scale showing 46.8% (28.5–55.0). Finally, insomnia was estimated at 30.9% (20.3–41.5), with significantly higher scores being reported in 2021 than in 2020 (Table 5).

### Policy and management responses to those impacts

Several policy and management responses by governments, regulatory and accreditation bodies, schools, hospitals, clinical departments, health systems and student organizations were identified. A commonly cited response was the transition of face-to-face learning to online formats [44], including online videos [45], game-based learning [46], virtual clinical placements [34, 47–49], virtual simulations [50], remote teaching of practical skills as well as augmented reality [51, 52]. Interviews also transitioned to virtual format after guidance by accreditation bodies [53, 54], and face-to-face conferences were replaced with large-scale virtual conferences [55]. There were also responses relating to online assessment [56].

COVID-19-specific learning was introduced in particular for in-service and postgraduate learners [57], such as workshops on the use of personal protective equipment (PPE) [58–60] and simulations for COVID-specific protocols [61, 62]. Institutions published regulations and recommendations safeguarding learners’ health and continued learning [57, 63, 64], while there were interventions to specifically support learners’ mental health [65, 66]. Undergraduate learners were also involved in volunteering towards supporting the COVID-19 response [67, 68]. Another policy response was early graduation of final-year students who could work in a clinical capacity [69]. An overview of the institutions enacting these responses and policies as identified in the second phase of our systematic review is summarized in Table 6.

### Outcomes of policy responses

#### Online and blended learning approaches

Overall 75.9% (74.2–77.7) of learners were satisfied with online learning. Learners appeared more satisfied with online clinical exposure, such as fully virtual clinical rotations and real patient encounters (86.9%, 79.5–93.1) or online practical courses (85.4%, 82.3–88.2) compared to predominantly theoretical courses (67.5%, 64.7–70.3). Satisfaction with virtual congresses was also high (84.1%, 71.0–94.0). Learner satisfaction rates with virtual methods were lower in the EMR and SEAR (Table 7).

Overall, 32.0% (29.3–34.8) of learners preferred fully online learning, which was lower than preferences for fully in-person learning (48.8%, 45.4–52.1) or for blended learning (56.0%, 51.2–60.7). Lastly, when examined about their willingness to maintain an online-only format or not, and a blended online and in-person training or not, 34.7% (30.7–38.8), and 68.1% (64.6–71.5) of them, respectively, replied positively.

As training level was increasing (undergraduates vs graduates vs continuing education), a gradually higher preference for online learning (29.5% vs 39.7% vs 39.9%) and lower preference for learning in-person (50.9%, 47.6%, 30.7%) were observed. Also, learners in the AMR and EUR expressed higher willingness to keep blended learning after the pandemic (Tables 8, 9, 10, 11, and 12).

Assessing the same outcomes for faculty, 71.8% (66.8–76.8) expressed satisfaction with online methods. Preference for online-only, in-person or blended training methods were, respectively, 25.5% (15.5–35.5), 58.7% (51.6–65.8), and 64.5% (47.8–81.2). Their willingness to maintain an online-only or a blended online and in-person teaching post-pandemic were 36.7% (22.3–51.2) and 65.6% (57.1–74.0), respectively**.**

Responses were overall effective, significantly increasing learner skills scores when compared to scores before the response or scores achieved with pre-pandemic comparators (Table 15).

#### Assessment

The satisfaction of learners with online assessments was 68.8% (60.7–76.3). Postgraduate learners were significantly more inclined towards the use of online assessments compared to undergraduate ones (86.6% vs 62.5%), and with female learners being less satisfied than males (38.7% vs 58.1%). Learners in EMR and SEAR were less satisfied with online assessment than their colleagues in EUR and AMR (Table 13). Candidates also achieved significantly higher mean scores at online assessments compared to previous, in-person assessments or with innovations in assessment compared to traditional [pre vs post: SMD = − 0.68 (95% CI − 0.96 to − 0.40)].

#### Volunteerism

Studies investigating willingness of learners to volunteer in the COVID-19 response were also included. Despite 62.2% (49.6–74.8) of learners expressing an intention to volunteer, 27.7% (18.6–36.8) of learners reported engaging in volunteer activity, with undergraduate learners volunteering much more (pooled estimate of 32.4%) than their graduate colleagues (pooled estimate of 9.1%) (Table 14, Fig. 4).

A full list of all outcomes, Forest plots (in which the extent of the variation in the pooled estimates is more visible) and funnel plots are available at Additional file 6 and Additional file 7. Publication bias was evident in about one-fourth of the analyses. The GRADE certainty of evidence was assessed as “very low” for all outcomes of the meta-analysis. Finally, alternative meta-analytical approaches additionally undertaken for our main analyses did not materially change our findings (Additional file 8).

A summary of our main findings can be found in Table 15, with additional interpretation in “Discussion”.

## Discussion

A summary and interpretation of our main findings can be found in Table 15.

### Impacts of the pandemic on health worker education

Our meta-analysis showed that 71% of learners reported their clinical training was adversely impacted by the pandemic. In a large study surveying medical students from South America, Japan and Europe, 93% of students reported a suspension of bedside teaching [70]. Trainees in surgical and procedural fields were severely affected, with 96% of surgery residents and early-career surgeons in the US reporting a disruption in their clinical experience, with an overall 84% reduction in their operative volume in the early phases of the pandemic [71]. Most included studies did not provide separate data on the type of surgery. In similar large-scale disruptions, achieving the difficult but crucial balance between patient and trainee safety with the necessary clinical training of health workers should be a priority for policymaking.

The extent of the impact on the mental health of learners is concerning and highlights the need for sufficient resources to support learners and faculty. Our meta-analysis revealed that about one in three learners suffered from at least moderate anxiety, depression, insomnia, or burnout. These appear to be higher than reported anxiety and depression in health workers, similar to the general population during the pandemic and similar to pre-pandemic studies. In an umbrella review of depression and anxiety among health workers (not learners) during the pandemic, anxiety and depression were estimated at 24.9% and 24.8%, respectively [72], although most meta-analyses also included mild forms of anxiety and depression. A different subgroup analysis estimated moderate or higher anxiety and depression in health workers at 6.88% (4.4–9.9) and 16.2% (12.8–19.9) [73], results much lower than ours. In the general population, anxiety and depression were estimated in one meta-analysis at 31.9% (27.5–36.7) and 33.7% (27.5–40.6) [74], similar to our estimate for health worker learners. Lastly, comparing our results with a 2018 meta-analysis, the prevalence of anxiety (33.7%, 10.1–58.9) and depression (39.2%, 29.0–49.5) might be similar among health learners before and during the COVID-19 pandemic [75], and warrants further study and policy interventions. Anxiety was significantly higher in 2021 studies compared to 2020, indicating a notable effect of persisting stressors on mental health and emphasizing the need for early intervention to prevent anxiety. Pharmacy learners were significantly more anxious, which may be associated with different backgrounds and levels of familiarity with the intense clinical environment at times of capacity, in comparison to medical and nursing colleagues.

Multiple studies showed female gender was a risk factor for increased anxiety and depression among health learners [71, 76–79]. In studies that investigated underlying stressors, learners showed a high level of anxiety about their relatives’ health [41, 80–83], getting infected with COVID-19 themselves [41, 80, 84, 85], lack of PPE [86, 87], failing their clinical obligations [88], the disruption of educational activities [89, 90], or financial reasons [88, 91, 92]. A UK study on the psychological well-being of health worker learners during the pandemic associated the educational disruption with a negative impact on mental health, estimating low well-being at 61.9%, moderate to high perceived stressfulness of training at 83.3% and high presenteeism at 50% despite high satisfaction with training (90%) [93]. Learners felt a lack of mental health resources and supports in some disciplines [93]. A US study found that lack of wellness framework and lack of personal protective equipment were predictors of increased depression and burnout in surgery residents and early-career surgeons, highlighting the importance of well-designed wellness initiatives and appropriate protection for learners [71]. A summary of protective and exacerbating factors identified from included studies is available in Table 16. An international study of medical students identified high rates of insomnia (57%), depressed mood (40%) as well as multiple physical symptoms including headache (36%), eye fatigue (57%) and back pain (49%) [70]. These important physical complaints were not included in our systematic review. Interestingly, time spent in front of a screen daily correlated positively with depression, insomnia and headache. Alcohol consumption declined during the pandemic, whereas cigarette and marijuana use was unchanged. Putting together these findings, trainees’ mental- and physical-health appears to be associated with multiple factors that should be targeted by policy interventions: gender disparities, lack of well-designed wellness frameworks, stressful training, lack of protective equipment and potential implications of increased screen time. It should be noted that variants of the MBI scale also tend to overestimate burnout rates [94], so these may be actually lower than reported by our study.

**Table 16:** Risks and protective factors for anxiety and depression among health worker learners

Risk factors for anxiety	Risk factors for depression	Protective factors for anxiety and depression
Female gender [76–79]		Programs placing emphasis on their learners’ wellness [95]
History of other physical [96] or mental health disease [97, 98], use of medications [99]		Increased physical activity [95]
Having relatives or acquaintances infected with COVID-19 [100]	Personal or financial concerns [76]	
Working in a region with high COVID-19 prevalence [101]	Postponement of final examinations [102]	
Working in COVID-19 isolation units [77, 96]	Reduced sleep [102]	
Rare communication with friends and family [103]	Increased duration of internet use [102]	
Lower family income [99]		
Living alone or living with a relative at high risk for COVID-19 infection [104]		

### Outcomes of policy responses

Learners’ satisfaction with the rapidly implemented policy of online learning was relatively high (76%), especially if it included patient contact or practical training, rather than a purely theoretical approach. However, although learners were relatively satisfied when the alternative was no education, their opinions seemed to change when presented with options for the future. Learners preferred face-to-face (49%) and blended (56%) over fully online education (32%). In addition, only a small percentage of students were willing to pursue an exclusively online learning format (35%) in the post-pandemic era, with their preference trending towards a blended model (68%). The “Best Evidence in Medical Education” series and other systematic reviews, including only studies published in 2020, showed that the rapid shift to online learning proved to be an easily accessible tool that was able to minimize the impact of early lockdowns, both in undergraduate and graduate education [105–107]. Adaptations included telesimulations, live-streaming of surgical procedures and the integration of students to support clinical services remotely. Challenges included the lack of personal interaction and standardized curricula. All studies showed high risk of bias and poor reporting of the educational setting and theory [105]. Out meta-analysis of all relevant studies spanning from 2020 to mid-2022 showed that the integration of practical skill training into online courses led to higher satisfaction rates, solidifying a well-known preference for active learning among health workers. Satisfaction and preference for online learning was significantly increased in postgraduate and continuing learners compared to undergraduates, indicating it may be better suited for advanced learners with busy schedules. Higher convenience and ability to manage one’s time more flexibly and efficiently were frequently reported reasons for satisfaction and preference for online education [108–111]. Ιn synchronous learning, interaction through interactive lectures or courses, quizzes, case-based discussions, social media, breakout rooms or journal clubs were associated with increased satisfaction [112–116]. Conversely, in asynchronous learning, the opportunity for self-paced study and more detailed review of study material increased satisfaction [117–119]. Limitations of online education included challenges in comprehending material in courses such as anatomy [120, 121], as well as lack of motivation among learners [122–125]. A different systematic review found medical students appreciated the ability to interact with patients from home, easier remote access to experts and peer mentoring, whereas they viewed technical issues, reduced engagement and worldwide inequality were viewed as negative attributes of online learning [126]. Interestingly, one study comparing medical and nursing student satisfaction across India found high dissatisfaction (42%, compared to 37% satisfaction) which was not significantly different between the two fields, and higher in first-year students. Supportive faculty was important in increasing satisfaction [121].

We found that learners performed better in online assessments compared to prior in-person ones. It is unknown whether this represents lower demands, inadequate supervision, or changes in the constructive alignment between learning outcomes (e.g., theoretical knowledge) and assessment modality (e.g., multiple choice questions) [127]. However, online assessment has significant limitations in evaluating hands-on skills. Learners perceived online assessments as less fair, as cheating can be easier [128–130], or felt unable to showcase their skills online [131]. Open-book assessments focusing on thinking instead of memorization were preferred by learners [132] and may be more appropriate for online assessment. A different systematic review including studies up to October 2021 reviewed adaptations in in-person and online clinical examinations of medical students. Overall, online or modified in-person clinical assessment was deemed feasible, with similar scores to prior in-person iterations, and well received by trainees [133].

Although 62% of learners reported a willingness to volunteer, one in three actually did. This could be due to health risks, lockdowns, lack of opportunity or time, or other factors. As expected, undergraduates had more time to actually volunteer than other groups, however willingness to volunteer was comparable between the different training levels. These activities made heavy use of technology and frequently involved telephone outreach and counseling of patients and the public [134–137]. Students were also employed clinically in hospitals or other settings [138] and assisted with food and PPE donation and other nonclinical activities such as babysitting [139]. Some accrediting institutions responded by recommending that volunteering activities be rewarded with academic credit and supervised adequately [140].

### Strengths of our study

To our knowledge, this is the largest systematic review and meta-analysis exploring the impact of the pandemic on the education and mental health of health worker learners. The vast amount of data allowed us to perform multiple subgroup analyses and explore the potential differences in training disruption, mental health and perceptions on educational innovations. We included health worker learners from all regions of the world, all occupations, and all levels of training. We also undertook sensitivity analyses by restricting our analyses to a homogenous sample of higher quality studies (e.g., by only pooling GAD-7/PHQ-9/MBI low risk of bias studies for anxiety/depression/burnout). These approaches demonstrated the robustness of our findings. Finally, we attempted to explore the effect of time on outcomes, given the dynamic character of the pandemic.

### Limitations of our study

Although we excluded duplicate publications, there is still a risk for overlap, as learners may have participated anonymously in multiple cross-sectional studies. We attempted to minimize this with sensitivity analyses excluding very large datasets. Second, satisfaction was extracted from a variety of definitions among different studies leading to considerable heterogeneity. While prior experience with virtual learning might have affected learners’ or faculty perceptions, its inconsistent reporting did not allow us to account for it. For similar reasons, we did not manage to quantify mild mental health disruption for anxiety and depression. Although multiple significant subgroup differences emerged, heterogeneity remained largely unresolved. Heterogeneity is inherently high in meta-analyses of proportions, and the large sample of studies along with the subjective nature of many outcomes are in part responsible. The precision in point estimates (i.e., the observed narrow CIs) is therefore mainly a consequence of the large sample rather than true low variation. Therefore, we advise cautious interpretation and assess all our outcomes as very-low-certainty of evidence. Our sample mainly represented undergraduate students, learners in medicine and Asia, with reduced representation from Africa, South America and Oceania. Therefore, our results should be generalized with caution. However, subgroup analyses provide some insight into intra-group differences. Last, the authors were unable to include studies published in Spanish, which may in part reflect the scarcity of included studies from South America. We did, however, include studies in German and French.

Quality assessment revealed mostly observational studies and self-reported outcomes. RCTs were scarce and a considerable subset of them at high risk of bias. Publication bias was also evident in one-fourth of our analyses, leading to potential overestimation of proportions (e.g., higher satisfaction may be reported more eagerly). The above are consistent with the challenge in the education literature, which tends to capture mostly Kirkpatrick Level 1 data [141] (learner reaction), instead of objective learning assessments or behavioral changes. However, at the early stages of the pandemic, the literature is more likely to include lower-level immediate outcomes. Future studies will likely capture more objective outcomes and similar reviews should be repeated. Educational experiences are difficult to standardize and measure, making strict evidence-informed practice difficult [142]. However, quantitative evidence of any form can be a significant contributor to policy change.

## Conclusion

Our systematic review and meta-analysis quantified the widespread disruption of health worker education during the early phases of the COVID-19 pandemic. Clinical training was severely disrupted, with many learners being redeployed and some expressing a need to prolong their training. About one in three learners screened positive for anxiety, depression, burnout or insomnia. Although learners from all occupations and countries were overall satisfied with new educational experiences including online learning, indicating a cultural shift towards the acceptability of online learning, they ultimately preferred in-person or blended formats. Learners in regions with lower satisfaction with online learning (e.g., Asian countries—especially EMR or SEAR), would need further support with resources to maximize learning opportunities. Our evidence supports acceptability for a shift to blended learning, especially for postgraduate learners. This can combine the adaptability and personalized online learning with in-person consolidation of interpersonal and practical skills, which both learners and educators agree is necessary. Policies should also prioritize prevention, screening, and interventions for anxiety, depression, insomnia, and burnout among not only health workers, but also undergraduate and graduate learners, who are significantly affected. A repeated large-scale review in a few years will be able to capture a more representative sample of countries, occupations and experiences. Our review aspires to inform future studies that will objectively evaluate the effectiveness of ensuing policy and management responses.

## Supplementary Information

**Additional file 1.** Additional information on the methods of the study. Full search strategy, extracted variables on predetermined Excel table, Modified Newcastle–Ottawa Scale (NOS) for Cross-Sectional studies, further explanations for statistical analyses.

**Additional file 2.** Citations of all studies included in the systematic review.

**Additional file 3.** Descriptive Graphs and Figures for the demographics and descriptive characteristics of included individuals.

**Additional file 4.** Demographics for each outcome quantitatively synthesized.

**Additional file 5.** Characteristics of eligible randomized controlled trials. PICO, findings and Risk of Bias-2 quality assessment of the 37 included randomized controlled trials.

**Additional file 6.** Summarized results of all meta-analyses.

**Additional file 7.** Forest plots and funnel plots of all meta-analyses.

**Additional file 8.** Sensitivity analyses. Alternative data synthesis methods/alternative meta-analytical approaches for the main analyses.

## Data Availability

The datasets generated and analyzed during the current study are available from the corresponding author upon reasonable request.

## Abbreviations

HW: Health worker
COVID-19: Coronavirus disease 2019
PRISMA: Preferred Reporting Items for Systematic Reviews and Meta-Analyses
AMSTAR-2: Measurement Tool to Assess Systematic Reviews-2
AMR: Region of the Americas
CI: Confidence interval
EMR: Eastern Mediterranean Region
EUR: European Region
FT: Freeman–Tukey
GAD-7: General Anxiety Disorder-7
GRADE: Grading of Recommendations Assessment, Development and Evaluation
ICHA: International Classification for Health Accounts
ISCO: International Standard Classification of Occupations
NOS: Newcastle–Ottawa Scale
PHQ-9: Patient Health Questionnaire-9
PPE: Personal Protective Equipment
RoB-2: Risk of Bias 2
SEAR: South East Asian Region
WHO: World Health Organization
WPR: Western Pacific Region
SD: Standard deviation
SMD: Standardized mean difference
IQR: Interquartile range
*N*: Number of individuals
*n*: Number of studies
DL: DerSimonian and Laird
RCT: Randomized controlled trial
