# Supplementary material for: Health worker education during the COVID-19 pandemic: global disruption, responses and lessons for the future—a systematic review and meta-analysis
Source: Hum Resour Health. 2023 Feb 24;21:13. doi: 10.1186/s12960-023-00799-4 (PMC9951171; doi:10.1186/s12960-023-00799-4)
Supplement: Supplementary file 1 — Additional file 1. Additional information on the methods of the study. Full search strategy, extracted variables on predetermined Excel table, Modified Newcastle–Ottawa Scale (NOS) for Cross-Sectional studies, further explanations for statistical analyses. [file 12960_2023_799_MOESM1_ESM.docx]

**Additional file 1: Methods – Additional Information on the Methods of the study.**

Full search strategy, extracted variables on predetermined Excel table, Modified Newcastle-Ottawa Scale (NOS) for Cross-Sectional studies, further explanations for statistical analyses.

1a: Details on statistical analysis

- Transformations

When continuous data was provided as median (interquartile range [IQR]) or median (range), we used validated formulae to transform it into mean (SD) [1, 2]. In case data was only reported for subgroups, we additionally calculated overall metrics [1].

- Main meta-analysis

We sought to stabilize the variance and achieve approximate normality of the meta-analyzed proportions by utilizing the Freeman-Tukey (FT) double arcsine transformation [3]. Added benefits of this method include admissibility of all studies to the meta-analysis (for example the logit transformation may exclude studies with proportions near 0% or 100%), while the pooled CIs always lie within the desired range of 0-100% [3, 4]. We carried out a meta-analysis of the FT transformed estimates using the DerSimonian and Laird (DL) random-effects model, with the estimate of tau^2^ being taken from the inverse variance fixed-effect model [5]. We used the originally suggested harmonic mean in the back-transformation formula of FT estimates to proportions [6]. When applicable, we pooled standardized mean differences (SMDs) with the method of Cohen, as sample sizes were relatively large and the Hedge’s correction factor seemed unnecessary [7].

- Additional meta-analytical approaches

To statistically better account for the anticipated considerable heterogeneity, we sought to investigate the robustness of our findings by performing additional meta-analytical approaches. Specifically, for our main analyses, we used: (i) the Paule-Mandel estimator to calculate the between-study variance. It has been demonstrated that the former outperforms the DL estimator when heterogeneity increases; [8] and (ii) the Hartung-Knapp method for the CI calculation, which has been shown to be preferable in several instances and is by definition more conservative than the standard method (i.e., guarantees a CI coverage equal to or wider than the standard) [9].

1b: Detailed Search Strategy for different databases

**Algorithm for Pubmed**

("covid" OR "covid-19" OR "coronavirus" OR "corona virus" OR "2019- nCoV" OR "Coronavirus"[Mesh] OR "SARS-CoV-2" OR "SARS-CoV-2"[Mesh] OR "severe acute respiratory syndrome coronavirus 2"[Supplementary Concept] OR "COVID-19"[Supplementary Concept] OR "Coronavirus Infections"[Mesh]) AND ("health care professional*" OR "health care worker*" OR "health worker*" OR "health profession*" OR "practitioner" OR "health personnel" OR "Health Personnel"[Mesh] OR "nurs*" OR "midwi*" OR "clinic*" OR "paramed*" OR "dent*" OR "medic*"[tiab] OR "physician*" OR "pharmac*" OR "surg*" OR "anesthesiol*" OR "anaesthesiol*" OR "Oncolog*" OR "neurolog*" OR "nephrolog*" OR "cardiolog*" OR "neonatolog*" OR "endocrinolog*" OR "gastroenterolog*" OR "pediatr*" OR "pulmonolog*" OR "radiolog*" OR "urolog*" OR "gynecolog*" OR "gynaecolog*" OR "rheumatolog*" OR "patholog*" OR "optometr*" OR "allied health" OR "therap*" OR "public health student*" OR "global health" OR "Students, Health Occupations"[Mesh] OR "residen*" OR "intern*" OR "Internship and Residency"[Mesh] OR "student") AND ("teach*" OR "education*" OR "train*" OR "learn*" OR "instruct*" OR "supervis*" OR "assessment*" OR "curricul*" OR "examination" OR "OSCE" OR "evaluation" OR "preceptor*" OR "interview" OR "selection" OR "recruitment" OR "clinical skills" OR "undergraduate" OR "pre-service" OR "postgraduate" OR "in-service" OR faculty[tiab] OR "staff" OR school[tiab] OR "institution" OR "Education"[Mesh]) AND (2020/01/01:2021/02/23[pdat])

**Algorithm for Embase**

("covid" OR "covid-19" OR "coronavirus" OR "corona virus" OR "2019- nCoV" OR "SARS-CoV-2" OR "severe acute respiratory syndrome coronavirus 2") AND ("health care professional*" OR "health care worker*" OR "health worker*" OR "health profession*" OR "practitioner" OR "health personnel" OR "nurs*" OR "midwi*" OR "clinic*" OR "paramed*" OR "dent*" OR "medic*":ti,ab OR "physician*" OR "pharmac*" OR "surg*" OR "anesthesiol*" OR "anaesthesiol*" OR "Oncolog*" OR "neurolog*" OR "nephrolog*" OR "cardiolog*" OR "neonatolog*" OR "endocrinolog*" OR "gastroenterolog*" OR "pediatr*" OR "pulmonolog*" OR "radiolog*" OR "urolog*" OR "gynecolog*" OR "gynaecolog*" OR "rheumatolog*" OR "patholog*" OR "optometr*" OR "allied health" OR "therap*" OR "public health student*" OR "global health" OR "residen*" OR "intern*" OR "student") AND ("teach*" OR "education*" OR "train*" OR "learn*" OR "instruct*" OR "supervis*" OR "assessment*" OR "curricul*" OR "examination" OR "OSCE" OR "evaluation" OR "preceptor*" OR "interview" OR "selection" OR "recruitment" OR "clinical skills" OR "undergraduate" OR "pre-service" OR "postgraduate" OR "in-service" OR faculty:ti,ab OR "staff" OR school:ti,ab OR "institution")

**Algorithm for Web of Science**

("covid" OR "covid-19" OR "coronavirus" OR "corona virus" OR "2019- nCoV" OR "SARS-CoV-2" OR "severe acute respiratory syndrome coronavirus 2") AND ("health care professional*" OR "health care worker*" OR "health worker*" OR "health profession*" OR "practitioner" OR "health personnel" OR "nurs*" OR "midwi*" OR "clinic*" OR "paramed*" OR "dent*" OR "medic*" OR "physician*" OR "pharmac*" OR "surg*" OR "anesthesiol*" OR "anaesthesiol*" OR "Oncolog*" OR "neurolog*" OR "nephrolog*" OR "cardiolog*" OR "neonatolog*" OR "endocrinolog*" OR "gastroenterolog*" OR "pediatr*" OR "pulmonolog*" OR "radiolog*" OR "urolog*" OR "gynecolog*" OR "gynaecolog*" OR "rheumatolog*" OR "patholog*" OR "optometr*" OR "allied health" OR "therap*" OR "public health student*" OR "global health" OR "residen*" OR "intern*" OR "student") AND ("teach*" OR "education*" OR "train*" OR "learn*" OR "instruct*" OR "supervis*" OR "assessment*" OR "curricul*" OR "examination" OR "OSCE" OR "evaluation" OR "preceptor*" OR "interview" OR "selection" OR "recruitment" OR "clinical skills" OR "undergraduate" OR "pre-service" OR "postgraduate" OR "in-service" OR “faculty” OR "staff" OR “school” OR "institution")

**Algorithm for CENTRAL**

(covid-19) AND (health care professional OR health worker) AND (teach OR education OR train OR learn)

**Algorithm for Google Scholar**

("covid" OR "covid-19" OR "coronavirus" OR "corona virus" OR "2019- nCoV" OR "SARS-CoV-2" OR "severe acute respiratory syndrome coronavirus 2") AND ("health care professional*" OR "health care worker*" OR "health worker*" OR "health profession*" OR "practitioner" OR "health personnel" OR "nurs*" OR "midwi*" OR "clinic*" OR "paramed*" OR "dent*" OR "medic*" OR "physician*" OR "pharmac*" OR "surg*" OR "anesthesiol*" OR "anaesthesiol*" OR "Oncolog*" OR "neurolog*" OR "nephrolog*" OR "cardiolog*" OR "neonatolog*" OR "endocrinolog*" OR "gastroenterolog*" OR "pediatr*" OR "pulmonolog*" OR "radiolog*" OR "urolog*" OR "gynecolog*" OR "gynaecolog*" OR "rheumatolog*" OR "patholog*" OR "optometr*" OR "allied health" OR "therap*" OR "public health student*" OR "global health" OR "residen*" OR "intern*" OR "student") AND ("teach*" OR "education*" OR "train*" OR "learn*" OR "instruct*" OR "supervis*" OR "assessment*" OR "curricul*" OR "examination" OR "OSCE" OR "evaluation" OR "preceptor*" OR "interview" OR "selection" OR "recruitment" OR "clinical skills" OR "undergraduate" OR "pre-service" OR "postgraduate" OR "in-service" OR “faculty” OR "staff" OR “school” OR "institution")

1c: Extracted Variables in Predesigned Excel Spreadsheet

**Study Characteristics**

- Title
- Journal
- Doi
- Publication date (Jan 2020-Dec 2021)
- Study start (Jan 2020-Dec 2021)
- Study end (Jan 2020-Dec 2021)
- Institution or Organization of Intevention
- Country
- Continent
- WHO Region
- Setting-1 (university/college/school/preclinical, WHO health care provider, not classified)
- WHO health care provider (academic teaching, community teaching, non-teaching, not specified)
- Setting-2 (urban, rural, not-specified)
- Study type (observational, RCT)
- Type of observational study (cross-sectional, case-control, cohort, including retrospective, prospective observational)

**Participant Demographics**

- Age (mean)
- Age (SD)
- Women (N)
- Women (%)
- Total N of study participants
- N of specific sub-population of participants
- Learner or Faculty
- HCW population according the 4-digit ISCO population
- Level of training of Learner (undergraduate, graduate trainee, continuing education, not specified)
- Level of training of Faculty (undergraduate, graduate trainee, continuing education, not specified)
- Specialty (e.g., medical specialty/area of practice)
- If student, year of studies

**Outcome 1: Impact of the pandemic on Health Worker Education**

**1.1: Training Disruption**

- Type of disruption (what changed, stopped, etc.)
- Participants (%) that perceived training disruption
- Participants (%) that perceived reduction in cases/patients/clinical activity
- Participants (%) that perceived reduction in surgeries/ invasive medical procedures
- Participants (%) that perceived reduction in non-invasive medical procedures/rounds/etc
- Trainees (%) believing their training (e.g. residency) should be prolonged due to the disruption

**1.2: Disruption of Career Plans**

- redeployment of participants (%)
- Trainees (%) re-thinking specialty (e.g., residency) selection or future plans

**1.3: Mental Health of learners**

*Anxiety*

- anxiety (%) (mild, moderate, severe)
- anxiety/ stress scale score (numeric)
- scale used
- range, cut-off

*Depression*

- depression (%) (mild, moderate, moderately severe, severe)
- depression scale score (numeric)
- scale used
- range, cut-off

*Insomnia*

- insomnia (%)
- insomnia scale score (numeric)
- scale used
- range, cut-off

*Burnout*

- burnout (%)
- burnout scale score (numeric)
- scale used
- range, cut-off

**Outcome 2: Policies**

- Type of policy
- Organization instituting or implementing the policy

**Outcome 3: Outcomes of policy responses**

**3.1: Innovations in training**

- Type of innovation (online vs face- to-face, other innovation)
- Brief description of comparator
- Prior existence of innovation (transform existing course into a remote format, did not exist prior to covid, transform existing course, but deliver face to face, not specified)
- training on covid-19 specific protocols (1=yes, 2=no)
- X skill % (before intervention, with old method of training)
- X skill % (after intervention)
- improvement of skill (1= yes, 2=no, 3=not specified)
- satisfaction % online (or new method)
- satisfaction % face-to-face (or old method)
- preference % online (or new method)
- preference % face-to-face (or old method)
- preference % blended learning/ combination of methods
- % of attendees wanting to keep innovation in the future; (online-only, blended)
- % of attendees NOT wanting to keep innovation in the future
- % of attendees who cannot afford online (or new method) or their environment is not adequate for remote studying
- Scale used, cut-off (by author, by us)

**3.2: Innovations in exam assessment/evaluation**

- description-type of innovation
- brief description of comparator
- satisfaction (%) online
- satisfaction (%) face-to-face
- preference (%) face-to-face
- preference (%) online
- mean score face-to-face / comparator
- SD face-to-face
- mean score online
- SD online

**3.3: Volunteerism**

- Participants who volunteered (%)
- Participants who wanted to volunteer

**Quality Assessment**

- Quality assessment Scale (NOS, modified NOS, ROB2)
- (Categories about each Scale in separate columns)

1d: Modified Newcastle-Ottawa Scale (mNOS) for Cross-Sectional Studies

1. Selection of participants

- Representativeness of the sample

*(1= random sampling or non-random, 0=selected group or no explanation)*

- Sample Size

*(1=justified and satisfactory [>60], 0=not justified)*

- Response Rate/ Non-responders

*(1=response rate >80%, 0=not)*

- Ascertainment of exposure (measurement tool)

*(2=validated measurement tool, 1=not verified but explained, 0=not explained)*

1. Comparability (Confounding factors are checked, and there is comparability between subject groups)

*(2=more than one factors checked, 1=one major factor checked, 0=no factors checked)*

1. Outcome

- Assessment of outcome

*(2=independent blind ass/ment or record linkage, 1=self report, 0=no description)*

- Statistical Analysis

*(1=statistical analysis adequate, tools described, 0=not adequate, no description)*

*Total maximum of 10 points*

References (for 1a section)

1. Higgins JP, Thomas J, Chandler J, Cumpston M, Li T, Page MJ, VA W: **Cochrane handbook for systematic reviews of interventions.** (Sons JW ed.; 2019.

2. Hozo SP, Djulbegovic B, Hozo I: **Estimating the mean and variance from the median, range, and the size of a sample.** *BMC Medical Research Methodology* 2005, **5:**13.

3. Nyaga VN, Arbyn M, Aerts M: **Metaprop: a Stata command to perform meta-analysis of binomial data.** *Archives of Public Health* 2014, **72:**39.

4. Barendregt JJ, Doi SA, Lee YY, Norman RE, Vos T: **Meta-analysis of prevalence.** *J Epidemiol Community Health* 2013, **67:**974-978.

5. DerSimonian R, Laird N: **Meta-analysis in clinical trials.** *Control Clin Trials* 1986, **7:**177-188.

6. Miller JJ: **The Inverse of the Freeman – Tukey Double Arcsine Transformation.** *The American Statistician* 1978, **32:**138-138.

7. LLC S: **STATA META-ANALYSIS REFERENCE MANUAL RELEASE 17.** 4905 Lakeway Drive, College Station, Texas 77845 Stata Press; 2021.

8. Veroniki AA, Jackson D, Viechtbauer W, Bender R, Bowden J, Knapp G, Kuss O, Higgins JP, Langan D, Salanti G: **Methods to estimate the between-study variance and its uncertainty in meta-analysis.** *Res Synth Methods* 2016, **7:**55-79.

9. IntHout J, Ioannidis JPA, Borm GF: **The Hartung-Knapp-Sidik-Jonkman method for random effects meta-analysis is straightforward and considerably outperforms the standard DerSimonian-Laird method.** *BMC Medical Research Methodology* 2014, **14:**25.
