# Supplementary material for: Health worker education during the COVID-19 pandemic: global disruption, responses and lessons for the future—a systematic review and meta-analysis
Source: Hum Resour Health. 2023 Feb 24;21:13. doi: 10.1186/s12960-023-00799-4 (PMC9951171; doi:10.1186/s12960-023-00799-4)
Supplement: Supplementary file 2 — Additional file 2. Citations of all studies included in the systematic review. [file 12960_2023_799_MOESM2_ESM.docx]

**Additional file 2 – Citations of all studies included in the systematic review.**

1. Bradford N: **Understanding COVID-19's impact on college students.** *Teach Learn Nurs* 2021, **16:**I-II.

2. Abati E, Costamagna G: **Education Research: Effect of the COVID-19 pandemic on neurology trainees in Italy: A resident-driven survey.** *Neurology* 2020, **95:**1061-1066.

3. Abbas M, Dhane M, Beniey M, Meloche-Dumas L, Eissa M, Guerard-Poirier N, El-Raheb M, Lebel-Guay F, Dubrowski A, Patocskai E: **Repercussions of the COVID-19 pandemic on the well-being and training of medical clerks: a pan-Canadian survey.** *BMC Med Educ* 2020, **20:**385.

4. Abbasi MS, Ahmed N, Sajjad B, Alshahrani A, Saeed S, Sarfaraz S, Alhamdan RS, Vohra F, Abduljabbar T: **E-Learning perception and satisfaction among health sciences students amid the COVID-19 pandemic.** *Work* 2020, **67:**549-556.

5. Abbasi S ea: **Perceptions of students regarding e-learning during covid-19 at a private medical college.** *Pakistan Journal of Medical Sciences*.

6. Abdelfattah D: **Stressors Occurring in Psychiatry Residents Working in Psychiatric Hospitals During the COVID-19 Pandemic.** *Prim Care Companion CNS Disord* 2020, **22**.

7. Abdessater M, Roupret M, Misrai V, Pinar U, Matillon X, Gondran-Tellier B, Freton L, Vallee M, Dominique I, Felber M, et al: **COVID-19 outbreak situation and its psychological impact among surgeons in training in France.** *World J Urol* 2021, **39:**971-972.

8. Abdollahi A, Labbaf A, Khabaz Mafinejad M, Sotoudeh-Anvari M, Azmoudeh-Ardalan F: **Online Assessment for Pathology Residents during the COVID-19 Pandemic: Report of an Experience.** *Iran J Pathol* 2021, **16:**75-78.

9. Abdulghani HM, Sattar K, Ahmad T, Akram A: **Association of COVID-19 Pandemic with undergraduate Medical Students' Perceived Stress and Coping.** *Psychol Res Behav Manag* 2020, **13:**871-881.

10. Abdulrazzaq MM ea: **Psychological Stress among Dental Students at Al-Iraqia University after Corona Virus Pandemic.** *Indian Journal of Forensic Medicine & Toxicology* 2020.

11. Abraham HN, Opara IN, Dwaihy RL, Acuff C, Brauer B, Nabaty R, Levine DL: **Engaging Third-Year Medical Students on Their Internal Medicine Clerkship in Telehealth During COVID-19.** *Cureus* 2020, **12:**e8791.

12. Abud BT, Hajnas NM, Redleaf M, Kerolus JL, Lee V: **Assessing the Impact of a Training Initiative for Nasopharyngeal and Oropharyngeal Swabbing for COVID-19 Testing.** *OTO Open* 2020, **4:**2473974X20953094.

13. Adams CC, Shih R, Peterson PG, Lee MH, Heltzel DA, Lattin GE: **The Impact of a Virtual Radiology Medical Student Rotation: Maintaining Engagement During COVID-19 Mitigation.** *Mil Med* 2020.

14. Adesunkanmi AO, Ubom AE, Olasehinde O, Wuraola FO, Ijarotimi OA, Okon NE, Ikimalo JI, Fasubaa OB, Adesunkanmi ARK: **Impact of the COVID-19 Pandemic on Surgical Residency Training: Perspective from a Low-Middle Income Country.** *World J Surg* 2021, **45:**10-17.

15. Adusumilli NC, Eleryan M, Tanner S, Friedman AJ: **Third-year dermatology resident anxiety in the era of COVID-19.** *J Am Acad Dermatol* 2020, **83:**969-971.

16. Aebischer O, Weilenmann S, Gachoud D, Mean M, Spiller TR: **Physical and psychological health of medical students involved in the coronavirus disease 2019 response in Switzerland.** *Swiss Med Wkly* 2020, **150:**w20418.

17. Afonso N, Kelekar A, Alangaden A: **"I Have a Cough": An Interactive Virtual Respiratory Case-Based Module.** *MedEdPORTAL* 2020, **16:**11058.

18. Agarwal S, Kaushik JS: **Student's Perception of Online Learning during COVID Pandemic.** *Indian J Pediatr* 2020, **87:**554.

19. Agius AM, Gatt G, Vento Zahra E, Busuttil A, Gainza-Cirauqui ML, Cortes ARG, Attard NJ: **Self-reported dental student stressors and experiences during the COVID-19 pandemic.** *J Dent Educ* 2021, **85:**208-215.

20. Aguilar-Galvez D, Noal FC, de Araujo FB, Arriola-Guillen LE: **Virtual learning object: An asynchronous solution for virtual learning in dentistry post COVID-19.** *J Dent Educ* 2020.

21. Ahmed SA, Hegazy NN, Abdel Malak HW, Cliff Kayser W, 3rd, Elrafie NM, Hassanien M, Al-Hayani AA, El Saadany SA, Ai-Youbi AO, Shehata MH: **Model for utilizing distance learning post COVID-19 using (PACT) a cross sectional qualitative study.** *BMC Med Educ* 2020, **20:**400.

22. Kalok A, Sharip S, Abdul Hafizz AM, Zainuddin ZM, Shafiee MN: **The Psychological Impact of Movement Restriction during the COVID-19 Outbreak on Clinical Undergraduates: A Cross-Sectional Study.** *Int J Environ Res Public Health* 2020, **17**.

23. Aker S, Midik O: **The Views of Medical Faculty Students in Turkey Concerning the COVID-19 Pandemic.** *J Community Health* 2020, **45:**684-688.

24. Akinkugbe AA, Garcia DT, Smith CS, Brickhouse TH, Mosavel M: **A descriptive pilot study of the immediate impacts of COVID-19 on dental and dental hygiene students' readiness and wellness.** *J Dent Educ* 2021, **85:**401-410.

25. Al-Ahmari AN, Ajlan AM, Bajunaid K, Alotaibi NM, Al-Habib H, Sabbagh AJ, Al-Habib AF, Baeesa SS: **Perception of Neurosurgery Residents and Attendings on Online Webinars During COVID-19 Pandemic and Implications on Future Education.** *World Neurosurg* 2021, **146:**e811-e816.

26. Al-Azzam N, Elsalem L, Gombedza F: **A cross-sectional study to determine factors affecting dental and medical students' preference for virtual learning during the COVID-19 outbreak.** *Heliyon* 2020, **6:**e05704.

27. Al-Balas M, Al-Balas HI, Jaber HM, Obeidat K, Al-Balas H, Aborajooh EA, Al-Taher R, Al-Balas B: **Distance learning in clinical medical education amid COVID-19 pandemic in Jordan: current situation, challenges, and perspectives.** *BMC Med Educ* 2020, **20:**341.

28. Al-Obaidi ZMJ ea: **The impact of E-learning on pharmacy education: Pharmacy students' perspective during COVID-19 pandemic.** *European Journal of Molecular and Clinical Medicine*.

29. Biermann M, Kanoun S, Davidsen T, Gray R: **An Open Source Solution for "Hands-on" teaching of PET/CT to Medical Students under the COVID-19 Pandemic.** *Nuklearmedizin* 2021, **60:**10-15.

30. Bitonti G, Palumbo AR, Gallo C, Rania E, Saccone G, De Vivo V, Zullo F, Di Carlo C, Venturella R: **Being an obstetrics and gynaecology resident during the COVID-19: Impact of the pandemic on the residency training program.** *Eur J Obstet Gynecol Reprod Biol* 2020, **253:**48-51.

31. Bolatov AK, Seisembekov TZ, Askarova AZ, Baikanova RK, Smailova DS, Fabbro E: **Online-Learning due to COVID-19 Improved Mental Health Among Medical Students.** *Med Sci Educ* 2021, **31:**183-192.

32. Brondani M, Donnelly L: **COVID-19 pandemic: Students' perspectives on dental geriatric care and education.** *J Dent Educ* 2020, **84:**1237-1244.

33. Buckstein M, Skubish S, Smith K, Braccia I, Green S, Rosenzweig K: **Experiencing the Surge: Report From a Large New York Radiation Oncology Department During the COVID-19 Pandemic.** *Adv Radiat Oncol* 2020, **5:**610-616.

34. Busetto GM, Del Giudice F, Mari A, Sperduti I, Longo N, Antonelli A, Cerruto MA, Costantini E, Carini M, Minervini A, et al: **How Can the COVID-19 Pandemic Lead to Positive Changes in Urology Residency?** *Front Surg* 2020, **7:**563006.

35. Byrnes YM, Civantos AM, Go BC, McWilliams TL, Rajasekaran K: **Effect of the COVID-19 pandemic on medical student career perceptions: a national survey study.** *Med Educ Online* 2020, **25:**1798088.

36. Cai Y, Gulati A, Jiam NT, Wai KC, Shuman EA, Pletcher SD, Durr ML, Chang JL: **Evolving Otolaryngology Resident Roles and Concerns at the Peak of the US COVID-19 Pandemic.** *Head Neck* 2020, **42:**3712-3719.

37. Camilleri M, Zhang X, Norris M, Monkhouse A, Harvey A, Wiseman A, Sinha P, Hemsley A, Tang S, Menon A, et al: **Covid-19 ICU remote-learning course (CIRLC): Rapid ICU remote training for frontline health professionals during the COVID-19 pandemic in the UK.** *J Intensive Care Soc* 2022, **23:**183-190.

38. Cao W, Fang Z, Hou G, Han M, Xu X, Dong J, Zheng J: **The psychological impact of the COVID-19 epidemic on college students in China.** *Psychiatry Res* 2020, **287:**112934.

39. Dabbagh A, Ahmadizadeh SN, Asgari S, Fani K, Massoudi N, Moshari M, Sezari P, Shokrollahi S, Tabashi S, Tajbakhsh A, Vosoughian M: **Attitudes of the Third-Year Clinical Anesthesiology Residents Toward an Independent Clinical Practice Rotation in COVID-19 Pandemic in Iran.** *Anesth Pain Med* 2020, **10:**e110755.

40. Daccache J, Khoury M, Habibi C, Bennett S: **More than Just Soup: Use of a Student-Led COVID-19 Social Pediatrics Initiative to Propose the Integration of Social Medicine Electives in Undergraduate Medical Education.** *J Med Educ Curric Dev* 2020, **7:**2382120520973210.

41. Darnton R, Lopez T, Anil M, Ferdinand J, Jenkins M: **Medical students consulting from home: A qualitative evaluation of a tool for maintaining student exposure to patients during lockdown.** *Med Teach* 2021, **43:**160-167.

42. Dasgupta S, Shakeel T, Gupta P, Kakkar A, Vats V, Jain M, Rathi V, Panwar J, Kaur K, Gupta H: **Impact of ophthalmic webinars on the resident's learning experience during COVID-19 pandemic: An insight into its present and future prospects.** *Indian J Ophthalmol* 2021, **69:**145-150.

43. Dash C, Venkataram T, Goyal N, Chaturvedi J, Raheja A, Singla R, Sardhara J, Gupta R: **Neurosurgery training in India during the COVID-19 pandemic: straight from the horse's mouth.** *Neurosurg Focus* 2020, **49:**E16.

44. De Ponti R, Marazzato J, Maresca AM, Rovera F, Carcano G, Ferrario MM: **Pre-graduation medical training including virtual reality during COVID-19 pandemic: a report on students' perception.** *BMC Med Educ* 2020, **20:**332.

45. Degraeve A, Lejeune S, Muilwijk T, Poelaert F, Piraprez M, Svistakov I, Roumeguere T, European Society of Residents in Urology B: **When residents work less, they feel better: Lessons learned from an unprecedent context of lockdown.** *Prog Urol* 2020, **30:**1060-1066.

46. DePietro DM, Santucci SE, Harrison NE, Kiefer RM, Trerotola SO, Sudheendra D, Shamimi-Noori S: **Medical Student Education During the COVID-19 Pandemic: Initial Experiences Implementing a Virtual Interventional Radiology Elective Course.** *Acad Radiol* 2021, **28:**128-135.

47. Detterline S, Hartman-Hall H, Garbow K, Rawal H, Blackwood D, Nizialek G, Nashaat Z: **An internal medicine residency's response to the COVID-19 crisis: caring for our residents while caring for our patients.** *J Community Hosp Intern Med Perspect* 2020, **10:**504-507.

48. DeVaro SN, Uner OE, Khalifa YM, Graubart EB: **Ophthalmology Education in COVID-19: A Remote Elective for Medical Students.** *J Acad Ophthalmol (2017)* 2020, **12:**e165-e170.

49. Dhahri AA, Arain SY, Memon AM, Rao A, Medical Education Pakistan collaborator g, Mian MA: **"The psychological impact of COVID-19 on medical education of final year students in Pakistan: A cross-sectional study".** *Ann Med Surg (Lond)* 2020, **60:**445-450.

50. Di Lorenzo F, Ercoli T, Cuffaro L, Barbato F, Iodice F, Tedeschi G, Bombaci A, SigN: **COVID-19 impact on neurology training program in Italy.** *Neurol Sci* 2021, **42:**817-823.

51. Díaz-Guio DA ea: **Cognitive load and performance of health care professionals in donning and doffing PPE before and after a simulation-based educational intervention and its implications during the COVID-19 pandemic for biosafety.** *Infez Med*.

52. Dimitriu MCT, Pantea-Stoian A, Smaranda AC, Nica AA, Carap AC, Constantin VD, Davitoiu AM, Cirstoveanu C, Bacalbasa N, Bratu OG, et al: **Burnout syndrome in Romanian medical residents in time of the COVID-19 pandemic.** *Med Hypotheses* 2020, **144:**109972.

53. Divatia H, Friedland AR: **Virtual Med-Peds: Description of the First Virtual Med-Peds Student Elective During COVID-19.** *Cureus* 2020, **12:**e11971.

54. Donohue KE, Farber DL, Goel N, Parrino CR, Retener NF, Rizvi S, Dittmar PC: **Quality Improvement Amid a Global Pandemic: A Virtual Curriculum for Medical Students in the Time of COVID-19.** *MedEdPORTAL* 2021, **17:**11090.

55. Dost S, Hossain A, Shehab M, Abdelwahed A, Al-Nusair L: **Perceptions of medical students towards online teaching during the COVID-19 pandemic: a national cross-sectional survey of 2721 UK medical students.** *BMJ Open* 2020, **10:**e042378.

56. Drexler R, Hambrecht JM, Oldhafer KJ: **Involvement of Medical Students During the Coronavirus Disease 2019 Pandemic: A Cross-Sectional Survey Study.** *Cureus* 2020, **12:**e10147.

57. Drozdowicz L, Gordon E, Shapiro D, Jacobson S, Zalpuri I, Stewart C, Lewis AL, Robinson L, Myint MT, Daniolos P, et al: **Sexual Health in Child and Adolescent Psychiatry: Multi-Site Implementation Through Synchronized Videoconferencing of an Educational Resource Using Standardized Patients.** *Front Psychiatry* 2020, **11:**593101.

58. Duggan MC, Goroncy A, Christmas C, Chippendale R: **Staying Afloat in the COVID-19 Storm: GERIAtrics Fellows Learning Online And Together (GERI-A-FLOAT).** *J Am Geriatr Soc* 2020, **68:**E54-E56.

59. Dunn K ea: **The impact of COVID-19 on RMO medical education and training at CDHB.** *N Z Med J*.

60. Durfee SM, Goldenson RP, Gill RR, Rincon SP, Flower E, Avery LL: **Medical Student Education Roadblock Due to COVID-19: Virtual Radiology Core Clerkship to the Rescue.** *Acad Radiol* 2020, **27:**1461-1466.

61. Dutta S, Ambwani S, Lal H, Ram K, Mishra G, Kumar T, Varthya SB: **The Satisfaction Level of Undergraduate Medical and Nursing Students Regarding Distant Preclinical and Clinical Teaching Amidst COVID-19 Across India.** *Adv Med Educ Pract* 2021, **12:**113-122.

62. Dwivedi D, Kaur N, Shukla S, Gandhi A, Tripathi S: **Perception of stress among medical undergraduate during coronavirus disease-19 pandemic on exposure to online teaching.** *National Journal of Physiology, Pharmacy and Pharmacology* 2020, **10**.

63. Ekert JO, Luchesa Smith A, Ramsey CL, Robinson N, Love J, Gothard P, Armitage AJ: **Medical student-led simulation in COVID-19 crisis.** *Clin Teach* 2021, **18:**252-257.

64. El Bahloul M ea: **ASSESSMENT OF MEDICAL EDUCATION IN THE TIME OF COVID 19: EXPERIENCE OF TANGIER MEDICAL SCHOOL IN MOROCCO.** *Journal of Medical and Surgical Research*.

65. El-Ghandour NMF, Ezzat AAM, Zaazoue MA, Gonzalez-Lopez P, Jhawar BS, Soliman MAR: **Virtual learning during the COVID-19 pandemic: a turning point in neurosurgical education.** *Neurosurg Focus* 2020, **49:**E18.

66. Elgzar WT, Al-Qahtani AM, Elfeki NK, Ibrahim HA: **COVID-19 Outbreak: Effect of an Educational Intervention Based on Health Belief Model on Nursing Students' Awareness and Health Beliefs at Najran University, Kingdom of Saudi Arabia.** *Afr J Reprod Health* 2020, **24:**78-86.

67. Elledge R, Williams R, Fowell C, Green J: **Maxillofacial education in the time of COVID-19: the West Midlands experience.** *Br J Oral Maxillofac Surg* 2022, **60:**52-57.

68. Elsalem L, Al-Azzam N, Jum'ah AA, Obeidat N: **Remote E-exams during Covid-19 pandemic: A cross-sectional study of students' preferences and academic dishonesty in faculties of medical sciences.** *Ann Med Surg (Lond)* 2021, **62:**326-333.

69. Elsayes KM, Marks RM, Kamel S, Towbin AJ, Kielar AZ, Patel P, Chernyak V, Fowler KJ, Nassar S, Soliman MA, et al: **Online Liver Imaging Course; Pivoting to Transform Radiology Education During the SARS-CoV-2 Pandemic.** *Acad Radiol* 2021, **28:**119-127.

70. Elzainy A, El Sadik A, Al Abdulmonem W: **Experience of e-learning and online assessment during the COVID-19 pandemic at the College of Medicine, Qassim University.** *J Taibah Univ Med Sci* 2020, **15:**456-462.

71. Engberg M, Bonde J, Sigurdsson ST, Moller K, Nayahangan LJ, Berntsen M, Eschen CT, Haase N, Bache S, Konge L, Russell L: **Training non-intensivist doctors to work with COVID-19 patients in intensive care units.** *Acta Anaesthesiol Scand* 2021, **65:**664-673.

72. Engler J ea: **How do family physicians use e-learning? Results from an exploratory process evaluation of a web-based training for family physcians on communication with cancer patients about complementary medicine.** *Zeitschrift fur Allgemeinmedizin*.

73. Escalon MX, Raum G, Tieppo Francio V, Eubanks JE, Verduzco-Gutierrez M: **The Immediate Impact of the Coronavirus Pandemic and Resulting Adaptations in Physical Medicine and Rehabilitation Medical Education and Practice.** *PM R* 2020, **12:**1015-1023.

74. Essilfie AA, Hurley ET, Strauss EJ, Alaia MJ: **Resident, Fellow, and Attending Perception of E-Learning During the COVID-19 Pandemic and Implications on Future Orthopaedic Education.** *J Am Acad Orthop Surg* 2020, **28:**e860-e864.

75. Eurboonyanun C, Wittayapairoch J, Aphinives P, Petrusa E, Gee DW, Phitayakorn R: **Adaptation to Open-Book Online Examination During the COVID-19 Pandemic.** *J Surg Educ* 2021, **78:**737-739.

76. Eweida RS, Rashwan ZI, Desoky GM, Khonji LM: **Mental strain and changes in psychological health hub among intern-nursing students at pediatric and medical-surgical units amid ambience of COVID-19 pandemic: A comprehensive survey.** *Nurse Educ Pract* 2020, **49:**102915.

77. Fabiani MA, Gonzalez-Urquijo M, Cassagne G, Dominguez A, Hinojosa-Gonzalez DE, Lozano-Balderas G, Cisneros Tinoco MA, Escotto Sanchez I, Esperon Percovich A, Vegas DH, et al: **Thirty-three vascular residency programs among 13 countries joining forces to improve surgical education in times of COVID-19: A survey-based assessment.** *Vascular* 2022, **30:**146-150.

78. Faria G, Tadros BJ, Holmes N, Virani S, Reddy GK, Dhinsa BS, Relwani J: **Redeployment of the trainee orthopaedic surgeon during COVID-19: a fish out of water?** *Acta Orthop* 2020, **91:**650-653.

79. Fatani TH: **Student satisfaction with videoconferencing teaching quality during the COVID-19 pandemic.** *BMC Med Educ* 2020, **20:**396.

80. Fekih-Romdhane F, Snene H, Jebri A, Ben Rhouma M, Cheour M: **Psychological impact of the Pandemic COVID-19 Outbreak Among Medical Residents in Tunisia.** *Asian J Psychiatr* 2020, **53:**102349.

81. Fero KE, Weinberger JM, Lerman S, Bergman J: **Perceived Impact of Urologic Surgery Training Program Modifications due to COVID-19 in the United States.** *Urology* 2020, **143:**62-67.

82. Ferrara M, Romano V, Steel DH, Gupta R, Iovino C, van Dijk EHC, OphthaTraining G, Romano MR: **Reshaping ophthalmology training after COVID-19 pandemic.** *Eye (Lond)* 2020, **34:**2089-2097.

83. Figueroa F, Figueroa D, Calvo-Mena R, Narvaez F, Medina N, Prieto J: **Orthopedic surgery residents' perception of online education in their programs during the COVID-19 pandemic: should it be maintained after the crisis?** *Acta Orthop* 2020, **91:**543-546.

84. Findyartini A, Anggraeni D, Husin JM, Greviana N: **Exploring medical students' professional identity formation through written reflections during the COVID-19 pandemic.** *J Public Health Res* 2020, **9:**1918.

85. Fischbeck S, Hardt J, Malkewitz C, Petrowski K: **Evaluation of a digitized physician-patient-communication course evaluated by preclinical medical students: a replacement for classroom education?** *GMS J Med Educ* 2020, **37:**Doc85.

86. Fitzgerald A, Konrad S: **Transition in learning during COVID-19: Student nurse anxiety, stress, and resource support.** *Nurs Forum* 2021, **56:**298-304.

87. Folkard SS, Sturch P, Mahesan T, Garnett S: **Effect of coronavirus disease 2019 on urological surgery services and training up to the peak of the pandemic in South East England.** *Journal of Clinical Urology* 2020, **14:**47-54.

88. Forster C, Eismann-Schweimler J, Stengel S, Bischoff M, Fuchs M, Graf von Luckner A, Ledig T, Barzel A, Maun A, Joos S, et al: **Opportunities and challenges of e-learning in vocational training in General Practice - a project report about implementing digital formats in the KWBW-Verbundweiterbildung(plus).** *GMS J Med Educ* 2020, **37:**Doc97.

89. Gallardo FC, Martin C, Targa Garcia AA, Bustamante JL, Nunez M, Feldman SE: **Home Program for Acquisition and Maintenance of Microsurgical Skills During the Coronavirus Disease 2019 Outbreak.** *World Neurosurg* 2020, **143:**557-563 e551.

90. Gallego-Gomez JI, Campillo-Cano M, Carrion-Martinez A, Balanza S, Rodriguez-Gonzalez-Moro MT, Simonelli-Munoz AJ, Rivera-Caravaca JM: **The COVID-19 Pandemic and Its Impact on Homebound Nursing Students.** *Int J Environ Res Public Health* 2020, **17**.

91. Garcia DT, Akinkugbe AA, Mosavel M, Smith CS, Brickhouse TH: **COVID-19 and Dental and Dental Hygiene Students' Career Plans.** *JDR Clin Trans Res* 2021, **6:**153-160.

92. Garcia MN, Whitener SJ, Ghassemi A, Bitter R, Miley D, Naylor J, Drukteinis S, Hildebolt CF: **The periodontal senior case clinical challenge: Students' opinions of a formative virtual assessment during the COVID-19 emergency.** *Eur J Dent Educ* 2021, **25:**778-784.

93. Generali L, Iani C, Macaluso GM, Montebugnoli L, Siciliani G, Consolo U: **The perceived impact of the COVID-19 pandemic on dental undergraduate students in the Italian region of Emilia-Romagna.** *Eur J Dent Educ* 2021, **25:**621-633.

94. Georgiou I, Hounat A, Park JJ, Gillespie C, Bandyopadhyay S, Saunders KEA: **The Factors That Influenced Medical Students' Decision to Work Within the NHS During the COVID-19 Pandemic-A National, Cross-sectional Study.** *J Occup Environ Med* 2021, **63:**296-301.

95. Givi B, Moore MG, Bewley AF, Coffey CS, Cohen MA, Hessel AC, Jalisi S, Kang S, Newman JG, Puscas L, et al: **Advanced head and neck surgery training during the COVID-19 pandemic.** *Head Neck* 2020, **42:**1411-1417.

96. Gol I, Erkin O: **Mental status of nursing students assessed using the general health questionnaire during the COVID-19 pandemic in Turkey.** *Perspect Psychiatr Care* 2021, **57:**1712-1718.

97. Gomez-Ibanez R, Watson C, Leyva-Moral JM, Aguayo-Gonzalez M, Granel N: **Final-year nursing students called to work: Experiences of a rushed labour insertion during the COVID-19 pandemic.** *Nurse Educ Pract* 2020, **49:**102920.

98. Gonzi G, Gwyn R, Rooney K, Boktor J, Roy K, Sciberras NC, Pullen H, Mohanty K: **The role of orthopaedic trainees during the COVID-19 pandemic and impact on post-graduate orthopaedic education: a four-nation survey of over 100 orthopaedic trainees.** *Bone Jt Open* 2020, **1:**676-682.

99. Gravas S, Ahmad M, Hernandez-Porras A, Furriel F, Alvarez-Maestro M, Kumar A, Lee KS, Azodoh E, Mburugu P, Sanchez-Salas R, et al: **Impact of COVID-19 on medical education: introducing homo digitalis.** *World J Urol* 2021, **39:**1997-2003.

100. Grova MM, Donohue SJ, Meyers MO, Kim HJ, Ollila DW: **Direct Comparison of In-Person Versus Virtual Interviews for Complex General Surgical Oncology Fellowship in the COVID-19 Era.** *Ann Surg Oncol* 2021, **28:**1908-1915.

101. Grzych G, Schraen-Maschke S: **Interactive pedagogical tools could be helpful for medical education continuity during COVID-19 outbreak.** *Ann Biol Clin (Paris)* 2020, **78:**446-448.

102. Guadix SW, Winston GM, Chae JK, Haghdel A, Chen J, Younus I, Radwanski R, Greenfield JP, Pannullo SC: **Medical Student Concerns Relating to Neurosurgery Education During COVID-19.** *World Neurosurg* 2020, **139:**e836-e847.

103. Soriano GP: **Psychometric Properties of ‘Attitude towards e-Learning Scale’ among Nursing Students.** *International Journal of Educational Sciences* 2020, **30**.

104. Guiter GE, Sapia S, Wright AI, Hutchins GGA, Arayssi T: **Development of a Remote Online Collaborative Medical School Pathology Curriculum with Clinical Correlations, across Several International Sites, through the Covid-19 Pandemic.** *Med Sci Educ* 2021, **31:**549-556.

105. Guo T, Kiong KL, Yao C, Windon M, Zebda D, Jozaghi Y, Zhao X, Hessel AC, Hanna EY: **Impact of the COVID-19 pandemic on Otolaryngology trainee education.** *Head Neck* 2020, **42:**2782-2790.

106. Anshu A, Pandey S, Gupta N: **“Innovative Anatomy assessment methods in COVID-19 Pandemic: statistical observations and students viewpoints”.** *Bangladesh Journal of Medical Science* 2020.

107. Gupta T, Nazif TM, Vahl TP, Ahmad H, Bortnick AE, Feit F, Jauhar R, Kandov R, Kim M, Kini A, et al: **Impact of the COVID-19 pandemic on interventional cardiology fellowship training in the New York metropolitan area: A perspective from the United States epicenter.** *Catheter Cardiovasc Interv* 2021, **97:**201-205.

108. Gupta B, Jain G, Mishra P, Pathak S: **Preparedness to combat COVID-19 via structured online training program regarding specific airway management: A prospective observational study.** *Indian J Anaesth* 2020, **64:**796-799.

109. Gupta S, Dabas A, Swarnim S, Mishra D: **Medical education during COVID-19 associated lockdown: Faculty and students' perspective.** *Med J Armed Forces India* 2021, **77:**S79-S84.

110. Guse J, Heinen I, Kurre J, Mohr S, Bergelt C: **Perception of the study situation and mental burden during the COVID-19 pandemic among undergraduate medical students with and without mentoring.** *GMS J Med Educ* 2020, **37:**Doc72.

111. Gutkin PM, Prionas ND, Minneci MO, Allen E, 3rd, Balazy KE, Rahimy E, Chang DT, Horst KC: **Telemedicine in Radiation Oncology: Is It Here to Stay? Impacts on Patient Care and Resident Education.** *Int J Radiat Oncol Biol Phys* 2020, **108:**416-420.

112. Guven Ozdemir N, Sonmez M: **The relationship between nursing students' technology addiction levels and attitudes toward e-learning during the COVID-19 pandemic: A cross-sectional study.** *Perspect Psychiatr Care* 2021, **57:**1442-1448.

113. Hahn TW: **Virtual Noon Conferences: Providing Resident Education and Wellness During the COVID-19 Pandemic.** *PRiMER* 2020, **4:**17.

114. Haldar R, Kannaujia AK, Shamim R, Dongare P, Mondal H, Agarwal A: **A national survey evaluating the effect of COVID-19 pandemic on the teaching and training of anaesthesiology postgraduate students in India.** *Indian J Anaesth* 2020, **64:**S227-S234.

115. Kurvatteppa H, Merlin C, Shabana K, Yadidya, Bhumika S, Vinod Kumar G, Auchitya S: **An effectiveness of training program on COVID-19 among healthcare students: A cross section study.** *International Journal of Research in Pharmaceutical Sciences* 2020, **11:**1250-1254.

116. Hameed T, Husain M, Jain SK, Singh CB, Khan S: **Online Medical Teaching in COVID-19 Era: Experience and Perception of Undergraduate Students.** *Maedica (Bucur)* 2020, **15:**440-444.

117. Hamilton WG, Loper NR, Abdel MP, Springer BD, Chen AF: **Adult Hip and Knee Reconstruction Education during the COVID-19 Pandemic.** *J Arthroplasty* 2021, **36:**S395-S399.

118. Hanson KA, Borofsky MS, Hampson LA, Breyer BN, Kern NG, Conti SL, Kielb SJ, Sorensen MD: **Capturing the Perspective of Prospective Urology Applicants: Impacts of COVID-19 on Medical Education.** *Urology* 2020, **146:**36-42.

119. Harendza S, Gartner J, Zelesniack E, Prediger S: **Evaluation of a telemedicine-based training for final-year medical students including simulated patient consultations, documentation, and case presentation.** *GMS J Med Educ* 2020, **37:**Doc94.

120. Haridy R, Abdalla MA, Kaisarly D, Gezawi ME: **A cross-sectional multicenter survey on the future of dental education in the era of COVID-19: Alternatives and implications.** *J Dent Educ* 2021, **85:**483-493.

121. Harky A, Karimaghaei D, Katmeh H, Hewage S: **The Impact of COVID-19 on Medical Examinations.** *Acta Biomed* 2020, **91:**e2020135.

122. Harmon DJ, Attardi SM, Barremkala M, Bentley DC, Brown KM, Dennis JF, Goldman HM, Harrell KM, Klein BA, Ramnanan CJ, et al: **An Analysis of Anatomy Education Before and During Covid‐19: May–August 2020.** *Anatomical Sciences Education* 2021, **14:**132-147.

123. Harries AJ, Lee C, Jones L, Rodriguez RM, Davis JA, Boysen-Osborn M, Kashima KJ, Krane NK, Rae G, Kman N, et al: **Effects of the COVID-19 pandemic on medical students: a multicenter quantitative study.** *BMC Med Educ* 2021, **21:**14.

124. Hashim A ea: **Student's satisfaction in online education programs among undergraduate physiotherapy students of lahore during covid-19.** *Rawal Medical Journal*.

125. He K, Stolarski A, Whang E, Kristo G: **Addressing General Surgery Residents' Concerns in the Early Phase of the COVID-19 Pandemic.** *J Surg Educ* 2020, **77:**735-738.

126. Heldt JP, Agrawal A, Loeb R, Richards MC, Castillo EG, DeBonis K: **We're Not Sure We Like It but We Still Want More: Trainee and Faculty Perceptions of Remote Learning During the COVID-19 Pandemic.** *Acad Psychiatry* 2021, **45:**598-602.

127. Hennessy O, Fowler AL, Hennessy C, Hogan A, Nugent E, Joyce M: **Covid 19 and Surgical training: Carpe Diem.** *Br J Surg* 2020, **107:**e591.

128. Herman A, Joseph JP, Ghosh S, Lloyd SK, Lakhani R, Cartwright S, Orban N: **National, Virtual Otolaryngology Training Day in the United Kingdom During the COVID-19 Pandemic: Results of a Pilot Survey.** *J Surg Educ* 2021, **78:**1376-1385.

129. Hernandez-Martinez A, Rodriguez-Almagro J, Martinez-Arce A, Romero-Blanco C, Garcia-Iglesias JJ, Gomez-Salgado J: **Nursing students' experience and training in healthcare aid during the COVID-19 pandemic in Spain.** *J Clin Nurs* 2021.

130. Hettle D, Sutherland K, Miles E, Allanby L, Bakewell Z, Davies D, Dhonye Y, Handford V, Upton R, Vilenchik V, Wood R: **Cross-skilling training to support medical redeployment in the COVID-19 pandemic.** *Future Healthc J* 2020, **7:**e41-e44.

131. Hilbert B ea: **The impact of the corona pandemic on working life of young family physicians an online survey among members of jade (Junge allgemeinmedizin deutschland).** *Zeitschrift fur Allgemeinmedizin*.

132. Hill MV, Ross EA, Crawford D, Lai L, Turaga K, Grubbs EG, Mullen J, Dineen S, D'Angelica M, Reddy S, Farma JM: **Program and candidate experience with virtual interviews for the 2020 Complex General Surgical Oncology interview season during the COVID pandemic.** *Am J Surg* 2021, **222:**99-103.

133. Ho J, Susser P, Christian C, DeLisser H, Scott MJ, Pauls LA, Huffenberger AM, Hanson CW, 3rd, Chandler JM, Fleisher LA, Laudanski K: **Developing the eMedical Student (eMS)-A Pilot Project Integrating Medical Students into the Tele-ICU during the COVID-19 Pandemic and beyond.** *Healthcare (Basel)* 2021, **9**.

134. Al-Taweel FB, Abdulkareem AA, Gul SS, Alshami ML: **Evaluation of technology-based learning by dental students during the pandemic outbreak of coronavirus disease 2019.** *Eur J Dent Educ* 2021, **25:**183-190.

135. Alawia R, Riad A, Kateeb E: **Risk perception and readiness of dental students to treat patients amid COVID-19: Implication for dental education.** *Oral Dis* 2022, **28 Suppl 1:**975-976.

136. Albitres-Flores L, Pisfil-Farronay YA, Guillen-Macedo K, Nino-Garcia R, Alarcon-Ruiz CA: **Interns' perceptions about the medical internship suspension during the COVID-19 quarantine.** *Rev Peru Med Exp Salud Publica* 2020, **37:**504-509.

137. Alhaj AK, Al-Saadi T, Mohammad F, Alabri S: **Neurosurgery Residents' Perspective on COVID-19: Knowledge, Readiness, and Impact of this Pandemic.** *World Neurosurg* 2020, **139:**e848-e858.

138. Ali A ea: **COVID-19 Online Teaching and its Impact on Psychological Health in Higher Education: A Cross Sectional Study on Medical Students of 1st 2nd and 3rd Year MBBS.** *Journal of Research in Medical and Dental Science*.

139. Aljehani YM, Othman SA, Telmesani NK, Alghamdi RA, AlBuainain HM, Alghamdi ZM, Zakaria HM, Alreshaid FT, Busbait SA, Alqarzaie AA, et al: **Stress and psychological resilience among general surgery residents during COVID-19 pandemic.** *Saudi Med J* 2020, **41:**1344-1349.

140. Alkhowailed MS, Rasheed Z, Shariq A, Elzainy A, El Sadik A, Alkhamiss A, Alsolai AM, Alduraibi SK, Alduraibi A, Alamro A, et al: **Digitalization plan in medical education during COVID-19 lockdown.** *Inform Med Unlocked* 2020, **20:**100432.

141. Almaghaslah D, Alsayari A: **The Effects of the 2019 Novel Coronavirus Disease (COVID-19) Outbreak on Academic Staff Members: A Case Study of a Pharmacy School in Saudi Arabia.** *Risk Manag Healthc Policy* 2020, **13:**795-802.

142. Almulhim AY, Almulhim SA, Almulhim AA, Khan AS: **The impact of e-learning modalities on medical students in KSA during the COVID-19 pandemic.** *J Taibah Univ Med Sci* 2020, **15:**437-438.

143. Alpert JB, Young MG, Lala SV, McGuinness G: **Medical Student Engagement and Educational Value of a Remote Clinical Radiology Learning Environment: Creation of Virtual Read-Out Sessions in Response to the COVID-19 Pandemic.** *Acad Radiol* 2021, **28:**112-118.

144. Alqudah NM, Jammal HM, Saleh O, Khader Y, Obeidat N, Alqudah J: **Perception and experience of academic Jordanian ophthalmologists with E-Learning for undergraduate course during the COVID-19 pandemic.** *Ann Med Surg (Lond)* 2020, **59:**44-47.

145. Alqurshi A: **Investigating the impact of COVID-19 lockdown on pharmaceutical education in Saudi Arabia - A call for a remote teaching contingency strategy.** *Saudi Pharm J* 2020, **28:**1075-1083.

146. Alsaywid B, Housawi A, Lytras M, Halabi H, Abuzenada M, Alhaidar SA, Abuznadah W: **Residents’ Training in COVID-19 Pandemic Times: An Integrated Survey of Educational Process, Institutional Support, Anxiety and Depression by the Saudi Commission for Health Specialties (SCFHS).** *Sustainability* 2020, **12**.

147. Alvarez ML, Waissbluth S, Gonzalez C, Napolitano C, Torrente M, Delano PH, Alarcon R, Fernandez F, Bitran R: **How the COVID-19 pandemic affects specialty training: An analysis of a nationwide survey among otolaryngology residents in Chile.** *Medwave* 2021, **21:**e8098.

148. Amir LR, Tanti I, Maharani DA, Wimardhani YS, Julia V, Sulijaya B, Puspitawati R: **Student perspective of classroom and distance learning during COVID-19 pandemic in the undergraduate dental study program Universitas Indonesia.** *BMC Med Educ* 2020, **20:**392.

149. Amparore D, Claps F, Cacciamani GE, Esperto F, Fiori C, Liguori G, Serni S, Trombetta C, Carini M, Porpiglia F, et al: **Impact of the COVID-19 pandemic on urology residency training in Italy.** *Minerva Urol Nefrol* 2020, **72:**505-509.

150. An TW, Henry JK, Igboechi O, Wang P, Yerrapragada A, Lin CA, Paiement GD: **How Are Orthopaedic Surgery Residencies Responding to the COVID-19 Pandemic? An Assessment of Resident Experiences in Cities of Major Virus Outbreak.** *J Am Acad Orthop Surg* 2020, **28:**e679-e685.

151. Anderi E, Sherman L, Saymuah S, Ayers E, Kromrei HT: **Learning Communities Engage Medical Students: A COVID-19 Virtual Conversation Series.** *Cureus* 2020, **12:**e9593.

152. Anthony JW ea: **Effect of lockdown on Students Studying in Various Colleges of Nursing.** *Indian Journal of Forensic Medicine & Toxicology* 2020.

153. Anwar A, Mansoor H, Faisal D, Khan HS: **E-Learning amid the COVID-19 Lockdown: Standpoint of Medical and Dental Undergraduates.** *Pak J Med Sci* 2021, **37:**217-222.

154. Arima M, Takamiya Y, Furuta A, Siriratsivawong K, Tsuchiya S, Izumi M: **Factors associated with the mental health status of medical students during the COVID-19 pandemic: a cross-sectional study in Japan.** *BMJ Open* 2020, **10:**e043728.

155. Armon S, Benyamini Y, Grisaru-Granovsky S, Avitan T: **Online Obstetrics and Gynecology Medical Students Clerkship During the Covid-19 Pandemic: a Pilot Study.** *Med Sci Educ* 2021, **31:**457-461.

156. Armstrong-Mensah E, Ramsey-White K, Yankey B, Self-Brown S: **COVID-19 and Distance Learning: Effects on Georgia State University School of Public Health Students.** *Front Public Health* 2020, **8:**576227.

157. Ashry AH, Soffar HM, Alsawy MF: **Neurosurgical education during COVID-19: challenges and lessons learned in Egypt.** *Egypt J Neurol Psychiatr Neurosurg* 2020, **56:**110.

158. Astorp MS, Sorensen GVB, Rasmussen S, Emmersen J, Erbs AW, Andersen S: **Support for mobilising medical students to join the COVID-19 pandemic emergency healthcare workforce: a cross-sectional questionnaire survey.** *BMJ Open* 2020, **10:**e039082.

159. Marina C, Gheoca-Mutu D, Răducu L, Avino A, Brîndușe L, Stefan C, Scaunasu R, Jecan C: **COVID-19 outbreak impact on plastic surgery residents from Romania.** *Journal of Mind and Medical Sciences* 2020**:**212-216.

160. Mark ME, LoSavio P, Husain I, Papagiannopoulos P, Batra PS, Tajudeen BA: **Effect of Implementing Simulation Education on Health Care Worker Comfort With Nasopharyngeal Swabbing for COVID-19.** *Otolaryngol Head Neck Surg* 2020, **163:**271-274.

161. Martin-Gorgojo A, Bernabeu-Wittel J, Linares-Barrios M, Russo-de la Torre F, Garcia-Doval I, Del Rio-de la Torre E: **Attendee Survey and Practical Appraisal of a Telegram(R)-Based Dermatology Congress During the COVID-19 Confinement.** *Actas Dermosifiliogr (Engl Ed)* 2020, **111:**852-860.

162. Martinez L, Holley A, Brown S, Abid A: **Addressing the Rapidly Increasing Need for Telemedicine Education for Future Physicians.** *PRiMER* 2020, **4:**16.

163. Mattila A, Martin RM, DeIuliis ED: **Simulated Fieldwork: A Virtual Approach to Clinical Education.** *Education Sciences* 2020, **10**.

164. McRoy C, Patel L, Gaddam DS, Rothenberg S, Herring A, Hamm J, Chelala L, Weinstein J, Smith E, Awan O: **Radiology Education in the Time of COVID-19: A Novel Distance Learning Workstation Experience for Residents.** *Acad Radiol* 2020, **27:**1467-1474.

165. Meo SA, Abukhalaf AA, Alomar AA, Sattar K, Klonoff DC: **COVID-19 Pandemic: Impact of Quarantine on Medical Students' Mental Wellbeing and Learning Behaviors.** *Pak J Med Sci* 2020, **36:**S43-S48.

166. Meybodi KT, Habibi Z, Nejat F: **The effects of COVID-19 pandemic on pediatric neurosurgery practice and training in a developing country.** *Childs Nerv Syst* 2021, **37:**1313-1317.

167. Michener A, Fessler E, Gonzalez M, Miller RK: **The 5 M's and More: A New Geriatric Medical Student Virtual Curriculum During the COVID-19 Pandemic.** *J Am Geriatr Soc* 2020, **68:**E61-E63.

168. Michno DA, Tan J, Adelekan A, Konczalik W, Woollard ACS: **How can we help? Medical students' views on their role in the COVID-19 pandemic.** *J Public Health (Oxf)* 2021, **43:**479-489.

169. Mileder LP, Schuttengruber G, Prattes J, Wegscheider T: **Simulation-based training and assessment of mobile pre-hospital SARS-CoV-2 diagnostic teams in Styria, Austria.** *Medicine (Baltimore)* 2020, **99:**e21081.

170. Milgrom Y, Richter V: **Stress assessment among internal medicine residents in a level-3 hospital versus a level-2 hospital with only emergency room service for COVID-19.** *J Community Hosp Intern Med Perspect* 2020, **10:**301-305.

171. Mishra D, Nair AG, Gandhi RA, Gogate PJ, Mathur S, Bhushan P, Srivastav T, Singh H, Sinha BP, Singh MK: **The impact of COVID-19 related lockdown on ophthalmology training programs in India - Outcomes of a survey.** *Indian J Ophthalmol* 2020, **68:**999-1004.

172. Misztal-Okonska P, Goniewicz K, Hertelendy AJ, Khorram-Manesh A, Al-Wathinani A, Alhazmi RA, Goniewicz M: **How Medical Studies in Poland Prepare Future Healthcare Managers for Crises and Disasters: Results of a Pilot Study.** *Healthcare (Basel)* 2020, **8**.

173. Montepara CA, Schoen RR, Guarascio AJ, McConaha JL, Horn PJ: **Health-system implementation of a collaborative core curriculum for advanced pharmacy experiential education during the COVID-19 pandemic.** *Am J Health Syst Pharm* 2021, **78:**890-895.

174. Moreira MRC, Aquino RF, Barros LL, Parente NC, Machado M, Oliveira AMF, Candido EL: **Do medical interns feel prepared to work in the COVID-19 pandemic?** *Rev Assoc Med Bras (1992)* 2020, **66:**973-978.

175. Moreno-Fernandez J, Ochoa JJ, Lopez-Aliaga I, Alferez MJM, Gomez-Guzman M, Lopez-Ortega S, Diaz-Castro J: **Lockdown, Emotional Intelligence, Academic Engagement and Burnout in Pharmacy Students during the Quarantine.** *Pharmacy (Basel)* 2020, **8**.

176. Mouli TC, Davuluri A, Vijaya S, Priyanka ADY, Mishra SK: **Effectiveness of simulation based teaching of ventilatory management among non-anaesthesiology residents to manage COVID 19 pandemic - A Quasi experimental cross sectional pilot study.** *Indian J Anaesth* 2020, **64:**S136-S140.

177. Moussa R ea: **Does it Rain after the Storm. Family medicine resident's reflection at Qatar: Results From a cross sectional study.** *World Family Medicine*.

178. Muflih S, Abuhammad S, Karasneh R, Al-Azzam S, Alzoubi KH, Muflih M: **Online Education for Undergraduate Health Professional Education during the COVID-19 Pandemic: Attitudes, Barriers, and Ethical Issues.** *Res Sq* 2020.

179. Muhammad Alfareed Zafar S, Junaid Tahir M, Malik M, Irfan Malik M, Kamal Akhtar F, Ghazala R: **Awareness, anxiety, and depression in healthcare professionals, medical students, and general population of Pakistan during COVID-19 Pandemic: A cross sectional online survey.** *Med J Islam Repub Iran* 2020, **34:**131.

180. Munjal T, Kavanagh KR, Ezzibdeh RM, Valdez TA: **The impact of COVID-19 on global disparities in surgical training in pediatric otolaryngology.** *Int J Pediatr Otorhinolaryngol* 2020, **138:**110267.

181. Mustafa RM, Alrabadi NN, Alshali RZ, Khader YS, Ahmad DM: **Knowledge, Attitude, Behavior, and Stress Related to COVID-19 among Undergraduate Health Care Students in Jordan.** *Eur J Dent* 2020, **14:**S50-S55.

182. Nanjundaswamy MH, Pathak H, Chaturvedi SK: **Perceived stress and anxiety during COVID-19 among psychiatry trainees.** *Asian J Psychiatr* 2020, **54:**102282.

183. Naroo SA, Morgan PB, Shinde L, Lee C, Ewbank A: **Contact lens education for the practitioners of the future.** *Ophthalmic Physiol Opt* 2021, **41:**603-609.

184. Naser Moghadasi A: **Evaluation of the Level of Anxiety among Iranian Multiple Sclerosis Fellowships During the Outbreak of COVID-19.** *Arch Iran Med* 2020, **23:**283.

185. Nearing KA, Lum HD, Dang S, Powers B, McLaren J, Gately M, Hung W, Moo L: **National Geriatric Network Rapidly Addresses Trainee Telehealth Needs in Response to COVID-19.** *J Am Geriatr Soc* 2020, **68:**1907-1912.

186. Nepal S, Atreya A, Menezes RG, Joshi RR: **Students' Perspective on Online Medical Education Amidst the COVID-19 Pandemic in Nepal.** *J Nepal Health Res Counc* 2020, **18:**551-555.

187. Neupane HC, Sharma K, Joshi A: **Readiness for the Online Classes during COVID-19 Pandemic among Students of Chitwan Medical College.** *J Nepal Health Res Counc* 2020, **18:**316-319.

188. Newcomb AB, Duval M, Bachman SL, Mohess D, Dort J, Kapadia MR: **Building Rapport and Earning the Surgical Patient's Trust in the Era of Social Distancing: Teaching Patient-Centered Communication During Video Conference Encounters to Medical Students.** *J Surg Educ* 2021, **78:**336-341.

189. Ng CKC: **Evaluation of academic integrity of online open book assessments implemented in an undergraduate medical radiation science course during COVID-19 pandemic.** *J Med Imaging Radiat Sci* 2020, **51:**610-616.

190. Ni J, Wang F, Liu Y, Wu M, Jiang Y, Zhou Y, Sha D: **Psychological Impact of the COVID-19 Pandemic on Chinese Health Care Workers: Cross-Sectional Survey Study.** *JMIR Ment Health* 2021, **8:**e23125.

191. Nickinson ATO, Carey F, Tan K, Ali T, Al-Jundi W: **Has the COVID-19 Pandemic Opened Our Eyes to the Potential of Digital Teaching? A Survey of UK Vascular Surgery and Interventional Radiology Trainees.** *Eur J Vasc Endovasc Surg* 2020, **60:**952-953.

192. Nihmath Nisha S, Yuvaraj Maria F, Balaji K, Gunapriya R, Kumaresan M: **A survey on anxiety and depression level among South Indian medical students during the COVID 19 pandemic.** *International Journal of Research in Pharmaceutical Sciences* 2020, **11:**779-786.

193. Nishimura Y, Ochi K, Tokumasu K, Obika M, Hagiya H, Kataoka H, Otsuka F: **Impact of the COVID-19 Pandemic on the Psychological Distress of Medical Students in Japan: Cross-sectional Survey Study.** *J Med Internet Res* 2021, **23:**e25232.

194. O'Connell A, Tomaselli PJ, Stobart-Gallagher M: **Effective Use of Virtual Gamification During COVID-19 to Deliver the OB-GYN Core Curriculum in an Emergency Medicine Resident Conference.** *Cureus* 2020, **12:**e8397.

195. Odedra D, Chahal BS, Patlas MN: **Impact of COVID-19 on Canadian Radiology Residency Training Programs.** *Can Assoc Radiol J* 2020, **71:**482-489.

196. Ogundele IO, Alakaloko FM, Nwokoro CC, Ameh EA: **Early impact of COVID-19 pandemic on paediatric surgical practice in Nigeria: a national survey of paediatric surgeons.** *BMJ Paediatr Open* 2020, **4:**e000732.

197. Olum R, Atulinda L, Kigozi E, Nassozi DR, Mulekwa A, Bongomin F, Kiguli S: **Medical Education and E-Learning During COVID-19 Pandemic: Awareness, Attitudes, Preferences, and Barriers Among Undergraduate Medicine and Nursing Students at Makerere University, Uganda.** *J Med Educ Curric Dev* 2020, **7:**2382120520973212.

198. Önöral Ö, Kurtulmus-Yilmaz S: **Influence of Covid-19 Pandemic on Dental Education in Cyprus: Preclinical and Clinical Implications with E-Learning Strategies.** *Advanced Education* 2020, **7:**69-77.

199. Osama M, Zaheer F, Saeed H, Anees K, Jawed Q, Syed SH, Sheikh BA: **Impact of COVID-19 on surgical residency programs in Pakistan; A residents' perspective. Do programs need formal restructuring to adjust with the "new normal"? A cross-sectional survey study.** *Int J Surg* 2020, **79:**252-256.

200. Ostapenko A, McPeck S, Liechty S, Kleiner D: **Impacts on Surgery Resident Education at a First Wave COVID-19 Epicenter.** *J Med Educ Curric Dev* 2020, **7:**2382120520975022.

201. Ostapenko A, McPeck S, Liechty S, Kleiner D: **Has COVID-19 Hurt Resident Education? A Network-Wide Resident Survey on Education and Experience During the Pandemic.** *J Med Educ Curric Dev* 2020, **7:**2382120520959695.

202. Paesano N, Santomil F, Tobia I: **Impact of COVID-19 Pandemic on Ibero-American Urology Residents: Perspective of American Confederation of Urology (CAU).** *Int Braz J Urol* 2020, **46:**165-169.

203. Palacios Huatuco RM, Liano JE, Moreno LB, Ponce Beti MS: **Analysis of the impact of the pandemic on surgical residency programs during the first wave in Argentina: A cross - sectional study.** *Ann Med Surg (Lond)* 2021, **62:**455-459.

204. Pandey U, Corbett G, Mohan S, Reagu S, Kumar S, Farrell T, Lindow S: **Anxiety, Depression and Behavioural Changes in Junior Doctors and Medical Students Associated with the Coronavirus Pandemic: A Cross-Sectional Survey.** *J Obstet Gynaecol India* 2021, **71:**33-37.

205. Park J, Park H, Lim JE, Rhim HC, Lee YM: **Medical students' perspectives on recommencing clinical rotations during coronavirus disease 2019 at one institution in South Korea.** *Korean J Med Educ* 2020, **32:**223-229.

206. Park JJ: **Medical student perceptions of working in clinical environments during the COVID-19 pandemic.** *J Glob Health* 2020, **10:**020380.

207. Pasricha ND, Haq Z, Ahmad TR, Chan L, Redd TK, Seitzman GD, Parikh N, Kim TN, Schallhorn JM, Ramanathan S: **Remote corneal suturing wet lab: microsurgical education during the COVID-19 pandemic.** *J Cataract Refract Surg* 2020, **46:**1667-1673.

208. Patel J, Robbins T, Randeva H, de Boer R, Sankar S, Brake S, Patel K: **Rising to the challenge: Qualitative assessment of medical student perceptions responding to the COVID-19 pandemic.** *Clin Med (Lond)* 2020, **20:**e244-e247.

209. Patel M, Menhadji P, Mayor S: **Redeployment of Dental Core Trainees in the United Kingdom due to Coronavirus Disease 2019.** *Eur J Dent* 2020, **14:**S44-S49.

210. Patel NM, Khajuria A, Khajuria A: **Utility of a webinar to educate trainees on UK core surgical training (CST) selection - A cross sectional study and future implications amidst the COVID-19 pandemic.** *Ann Med Surg (Lond)* 2020, **59:**35-40.

211. Patel NR, El-Karim GA, Mujoomdar A, Mafeld S, Jaberi A, Kachura JR, Tan KT, Oreopoulos GD: **Overall Impact of the COVID-19 Pandemic on Interventional Radiology Services: A Canadian Perspective.** *Can Assoc Radiol J* 2021, **72:**564-570.

212. Patel SM, Miller CR, Schiavi A, Toy S, Schwengel DA: **The sim must go on: adapting resident education to the COVID-19 pandemic using telesimulation.** *Adv Simul (Lond)* 2020, **5:**26.

213. Payne A, Rahman R, Bullingham R, Vamadeva S, Alfa-Wali M: **Redeployment of Surgical Trainees to Intensive Care During the COVID-19 Pandemic: Evaluation of the Impact on Training and Wellbeing.** *J Surg Educ* 2021, **78:**813-819.

214. Pelaccia T, Sibilia J, Fels E, Gauer L, Musanda A, Severac F, Abbiati M: **And if we had to do it all over again, would we send medical students to the emergency departments during a pandemic? Lessons learned from the COVID-19 outbreak.** *Intern Emerg Med* 2021, **16:**1967-1974.

215. Pelargos PE, Chakraborty A, Zhao YD, Smith ZA, Dunn IF, Bauer AM: **An Evaluation of Neurosurgical Resident Education and Sentiment During the Coronavirus Disease 2019 Pandemic: A North American Survey.** *World Neurosurg* 2020, **140:**e381-e386.

216. Pennington Z, Lubelski D, Khalafallah AM, Ehresman J, Sciubba DM, Witham TF, Huang J: **Letter to the Editor "Changes to Neurosurgery Resident Education Since Onset of the COVID-19 Pandemic".** *World Neurosurg* 2020, **139:**734-740.

217. Perrone MA, Youssefzadeh K, Serrano B, Limpisvasti O, Banffy M: **The Impact of COVID-19 on the Sports Medicine Fellowship Class of 2020.** *Orthop J Sports Med* 2020, **8:**2325967120939901.

218. Peterseim C, Watson KH: **Family Medicine Telehealth Clinic With Medical Students.** *PRiMER* 2020, **4:**35.

219. Pettitt-Schieber B, Kuo M, Steehler A, Dong A, Fakunle O, Manalo T, Mercury O, Simpson F, Guisse N, Studer M, et al: **Implementation and evaluation of eight virtual surgical electives for medical students during the COVID-19 pandemic.** *Am J Surg* 2021, **222:**248-253.

220. Pokrajac N, Schertzer K, Poffenberger CM, Alvarez A, Marin-Nevarez P, Winstead-Derlega C, Gisondi MA: **Mastery Learning Ensures Correct Personal Protective Equipment Use in Simulated Clinical Encounters of COVID-19.** *West J Emerg Med* 2020, **21:**1089-1094.

221. Prigoff J, Hunter M, Nowygrod R: **Medical Student Assessment in the Time of COVID-19.** *J Surg Educ* 2021, **78:**370-374.

222. Prince DS, Liu K, Pavendranathan G, Strasser SI, Bollipo S, Kanazaki R: **The impact of the COVID-19 pandemic on gastroenterology trainees in Australia.** *J Gastroenterol Hepatol* 2020, **35:**1841-1842.

223. Pritchard AB, Sloan-Heggen C, Keegan CE, Quinonez SC: **Trainee perspectives of COVID-19 impact on medical genetics education.** *Genet Med* 2021, **23:**956-962.

224. Puljak L, Civljak M, Haramina A, Malisa S, Cavic D, Klinec D, Aranza D, Mesaric J, Skitarelic N, Zoranic S, et al: **Attitudes and concerns of undergraduate university health sciences students in Croatia regarding complete switch to e-learning during COVID-19 pandemic: a survey.** *BMC Med Educ* 2020, **20:**416.

225. Qanash S, Al-Husayni F, Alemam S, Alqublan L, Alwafi E, Mufti HN, Qanash H, Shabrawishi M, Ghabashi A: **Psychological Effects on Health Science Students After Implementation of COVID-19 Quarantine and Distance Learning in Saudi Arabia.** *Cureus* 2020, **12:**e11767.

226. Rafi AM, Varghese PR, Kuttichira P: **The Pedagogical Shift During COVID 19 Pandemic: Online Medical Education, Barriers and Perceptions in Central Kerala.** *J Med Educ Curric Dev* 2020, **7:**2382120520951795.

227. Rainford LA, Zanardo M, Buissink C, Decoster R, Hennessy W, Knapp K, Kraus B, Lanca L, Lewis S, Mahlaola TB, et al: **The impact of COVID-19 upon student radiographers and clinical training.** *Radiography (Lond)* 2021, **27:**464-474.

228. Rajab MH, Gazal AM, Alkattan K: **Challenges to Online Medical Education During the COVID-19 Pandemic.** *Cureus* 2020, **12:**e8966.

229. Rezvani M, Smith GA, Majzoub JA, Durbin AD, Winn AS: **A Resident-Led Virtual Journal Club to Educate Pediatric Residents About Coronavirus Disease 2019.** *Acad Pediatr* 2021, **21:**759-761.

230. Rishi SK ea: **To Study Efficacy of Online Classes among Medical Students During Covid-19 Situation.** *Indian Journal of Public Health Research & Development* 2020.

231. Robertson B, McDermott C, Star J, Lewin LO, Spell N: **Synchronous virtual interprofessional education focused on discharge planning.** *J Interprof Educ Pract* 2021, **22:**100388.

232. Roemmele C, Manzeneder J, Messmann H, Ebigbo A: **Impact of the COVID-19 outbreak on endoscopy training in a tertiary care centre in Germany.** *Frontline Gastroenterol* 2020, **11:**454-457.

233. Rosenthal HB, Sikka N, Lieber AC, Sanky C, Cayon C, Newman D, Marquez DR, Ziff J, Blum JR, Dai JB, et al: **A Near-Peer Educational Model for Online, Interactive Learning in Emergency Medicine.** *West J Emerg Med* 2020, **22:**130-135.

234. Rosenzweig B, Bex A, Dotan ZA, Frydenberg M, Klotz L, Lotan Y, Schulman CC, Tsaur I, Ramon J: **Trends in urologic oncology clinical practice and medical education under COVID-19 pandemic: An international survey of senior clinical and academic urologists.** *Urol Oncol* 2020, **38:**929 e921-929 e910.

235. Aulakh GS, Duggal S, Sutton D: **Findings from an OMFS journal club: is COVID-19 the catalyst we have needed to embrace technology?** *Br J Oral Maxillofac Surg* 2022, **60:**46-51.

236. Aziz H, James T, Remulla D, Sher L, Genyk Y, Sullivan ME, Sheikh MR: **Effect of COVID-19 on Surgical Training Across the United States: A National Survey of General Surgery Residents.** *J Surg Educ* 2021, **78:**431-439.

237. Aziz A ea: **A Bumpy Road to Online Teaching: Impact of COVID-19 on Medical Education.** *Annals of King Edward Medical University Lahore Pakistan*.

238. Baczek M, Zaganczyk-Baczek M, Szpringer M, Jaroszynski A, Wozakowska-Kaplon B: **Students' perception of online learning during the COVID-19 pandemic: A survey study of Polish medical students.** *Medicine (Baltimore)* 2021, **100:**e24821.

239. Balhareth A, AlDuhileb MA, Aldulaijan FA, Aldossary MY: **Impact of COVID-19 pandemic on residency and fellowship training programs in Saudi Arabia: A nationwide cross-sectional study.** *Ann Med Surg (Lond)* 2020, **57:**127-132.

240. Balikai SC, Badheka A, Casey A, Endahl E, Erdahl J, Fayram L, Houston A, Levett P, Seigel H, Vijayakumar N, Cifra CL: **Simulation to Train Pediatric ICU Teams in Endotracheal Intubation of Patients with COVID-19.** *Pediatr Qual Saf* 2021, **6:**e373.

241. Bamba R, Bhagat N, Tran PC, Westrick E, Hassanein AH, Wooden WA: **Virtual Interviews for the Independent Plastic Surgery Match: A Modern Convenience or a Modern Misrepresentation?** *J Surg Educ* 2021, **78:**612-621.

242. Banava S, Jorquera J, Iyer P: **Virtual caries risk assessment workshop in COVID-19 era: Innovative game-based strategy.** *J Dent Educ* 2021, **85 Suppl 3:**1922-1924.

243. Bandi F, Karligkiotis A, Mellia J, Gallo S, Turri-Zanoni M, Battaglia P, Castelnuovo P: **Strategies to overcome limitations in Otolaryngology residency training during the COVID-19 pandemic.** *Eur Arch Otorhinolaryngol* 2020, **277:**3503-3506.

244. Barik S, Paul S, Kandwal P: **Insight into the changing patterns in clinical and academic activities of the orthopedic residents during COVID-19 pandemic: a cross-sectional survey.** *Knee Surg Sports Traumatol Arthrosc* 2020, **28:**3087-3093.

245. Barratt R, Shaban RZ, Gilbert GL: **Characteristics of personal protective equipment training programs in Australia and New Zealand hospitals: A survey.** *Infect Dis Health* 2020, **25:**253-261.

246. Bashir TF, Hassan S, Maqsood A, Khan ZA, Issrani R, Ahmed N, Bashir EF: **The Psychological Impact Analysis of Novel COVID-19 Pandemic in Health Sciences Students: A Global Survey.** *European Journal of Dentistry* 2020, **14:**S91-S96.

247. Batais MA, Temsah MH, AlGhofili H, AlRuwayshid N, Alsohime F, Almigbal TH, Al-Rabiaah A, Al-Eyadhy AA, Mujammami MH, Halwani R, et al: **The coronavirus disease of 2019 pandemic-associated stress among medical students in middle east respiratory syndrome-CoV endemic area: An observational study.** *Medicine (Baltimore)* 2021, **100:**e23690.

248. Bautista CA, Huang I, Stebbins M, Floren LC, Wamsley M, Youmans SL, Hsia SL: **Development of an interprofessional rotation for pharmacy and medical students to perform telehealth outreach to vulnerable patients in the COVID-19 pandemic.** *J Interprof Care* 2020, **34:**694-697.

249. Bazan D, Nowicki M, Rzymski P: **Medical students as the volunteer workforce during the COVID-19 pandemic: Polish experience.** *Int J Disaster Risk Reduct* 2021, **55:**102109.

250. Behrends M, Hoffmann I, Marschollek M, Hi GC: **Teamwork, communication and exchange despite Covid-19 - experiences from a digital elective in human medicine studies as part of the HiGHmed project.** *GMS J Med Educ* 2020, **37:**Doc86.

251. Belfi LM, Dean KE, Bartolotta RJ, Shih G, Min RJ: **Medical student education in the time of COVID-19: A virtual solution to the introductory radiology elective.** *Clin Imaging* 2021, **75:**67-74.

252. Bellini P, Iani C, Zucchelli G, Franchi M, Mattioli AV, Consolo U: **Impact of the COVID-19 pandemic on dental hygiene students in the Italian region of Emilia-Romagna.** *Minerva Dent Oral Sci* 2022, **71:**180-191.

253. Berger WR, Baggen V, Vorselaars VMM, van der Heijden AC, van Hout GPJ, Kapel GFL, Woudstra P, Junior Board of the Netherlands Society of C: **Dutch cardiology residents and the COVID-19 pandemic: Every little thing counts in a crisis.** *Neth Heart J* 2020, **28:**625-627.

254. Bernardi L, Germani P, Del Zotto G, Scotton G, de Manzini N: **Impact of COVID-19 pandemic on general surgery training program: An Italian experience.** *Am J Surg* 2020, **220:**1361-1363.

255. Beshyah SA, Ibrahim WH, Hajjaji IM, Ben Mami F, Arekat M, Abdelmannan DK: **Impact of the COVID-19 Pandemic on Clinical Practice, Medical Education, and Research: An International Survey.** *Tunis Med* 2020, **98:**610-618.

256. Bhandoria G, Shylasree TS, Bhandarkar P, Ahuja V, Maheshwari A, Sekhon R, Somashekhar SP: **Impact of COVID-19 Pandemic on Gynecological Oncology Care: Glimpse into Association of Gynecological Oncologists of India (AGOI) Perspective.** *Indian J Gynecol Oncol* 2020, **18:**71.

257. S. B: **Online Classes for Medical Students During COVID-19 Pandemic: Through the Eyes of the Teaching Faculty.** *Journal of Research in Medical and Dental Science*.

258. Bhat PS, Kaliavaradan S, Muruganidhi N, Sethu PL: **Model for hands-on tonsillectomy surgical training of postgraduate residents during COVID-19 pandemic.** *Eur Arch Otorhinolaryngol* 2021, **278:**2631-2636.

259. Sabahat GUL ea: **The impact of COVID-19 on medical education: Our students perception on practice of online education.**

260. Saddik B, Hussein A, Sharif-Askari FS, Kheder W, Temsah MH, Koutaich RA, Haddad ES, Al-Roub NM, Marhoon FA, Hamid Q, Halwani R: **Increased Levels of Anxiety Among Medical and Non-Medical University Students During the COVID-19 Pandemic in the United Arab Emirates.** *Risk Manag Healthc Policy* 2020, **13:**2395-2406.

261. Sadeesh T, Prabavathy G, Ganapathy A: **Evaluation of undergraduate medical students' preference to human anatomy practical assessment methodology: a comparison between online and traditional methods.** *Surg Radiol Anat* 2021, **43:**531-535.

262. Sakusic A, Markotic D, Dong Y, Festic E, Krajinovic V, Todorovic Z, Sustic A, Milivojevic N, Jandric M, Gavrilovic S, et al: **Rapid, multimodal, critical care knowledge-sharing platform for COVID-19 pandemics.** *Bosn J Basic Med Sci* 2021, **21:**93-97.

263. Samueli B, Sror N, Jotkowitz A, Taragin B: **Remote pathology education during the COVID-19 era: Crisis converted to opportunity.** *Ann Diagn Pathol* 2020, **49:**151612.

264. Sandhaus Y, Kushnir T, Ashkenazi S: **Electronic Distance Learning of Pre-clinical Studies During the COVID-19 Pandemic: A Preliminary Study of Medical Student Responses and Potential Future Impact.** *Isr Med Assoc J* 2020, **22:**489-493.

265. Sandhu N, Frank J, von Eyben R, Miller J, Obeid JP, Kastelowitz N, Panjwani N, Soltys S, Bagshaw HP, Donaldson SS, et al: **Virtual Radiation Oncology Clerkship During the COVID-19 Pandemic and Beyond.** *Int J Radiat Oncol Biol Phys* 2020, **108:**444-451.

266. Sanghavi PB, Au Yeung K, Sosa CE, Veesenmeyer AF, Limon JA, Vijayan V: **Effect of the Coronavirus Disease 2019 (COVID-19) Pandemic on Pediatric Resident Well-Being.** *J Med Educ Curric Dev* 2020, **7:**2382120520947062.

267. Santamaria E, Nahas-Combina L, Altamirano-Arcos C, Vargas-Flores E: **Master Series Microsurgery for Residents: Results from a Comprehensive Survey of a Multitudinous Online Course during COVID-19 Pandemic.** *J Reconstr Microsurg* 2021, **37:**602-607.

268. Sarani B, Shiroff A, Pieracci FM, Gasparri M, White T, Whitbeck S, Gross R: **Use of the Internet to Facilitate an Annual Scientific Meeting: A Report of the First Virtual Chest Wall Injury Society Summit.** *J Surg Educ* 2021, **78:**889-895.

269. Sarkar S, Mishra P, Nayak A: **Online open-book examination of undergraduate medical students - a pilot study of a novel assessment method used during the coronavirus disease 2019 pandemic.** *J Laryngol Otol* 2021, **135:**288-292.

270. Sarwar H, Akhtar H, Naeem MM, Khan JA, Waraich K, Shabbir S, Hasan A, Khurshid Z: **Self-Reported Effectiveness of e-Learning Classes during COVID-19 Pandemic: A Nation-Wide Survey of Pakistani Undergraduate Dentistry Students.** *Eur J Dent* 2020, **14:**S34-S43.

271. Sasitharan A: **COVID-19: The impacts on foundation training in district general hospitals in the East of England and the East Midlands.** *Clin Med (Lond)* 2020, **20:**e253-e254.

272. Savitsky B, Findling Y, Ereli A, Hendel T: **Nursing Students in Crisis Mode: Fluctuations in Anxiety During the COVID-19-Related Lockdown.** *Nurse Educ* 2021, **46:**E33-E38.

273. Sawarkar G, Sawarkar P, Kuchewar V: **Ayurveda students' perception toward online learning during the COVID-19 pandemic.** *J Educ Health Promot* 2020, **9:**342.

274. Schlenz MA, Schmidt A, Wostmann B, Kramer N, Schulz-Weidner N: **Students' and lecturers' perspective on the implementation of online learning in dental education due to SARS-CoV-2 (COVID-19): a cross-sectional study.** *BMC Med Educ* 2020, **20:**354.

275. Seow YT, Teo SC, Yap W, Foo Z, Tan KH: **Interactive videoconferencing in the redesign of a health-care quality improvement workshop for the coronavirus disease 2019 pandemic.** *Proceedings of Singapore Healthcare* 2020, **30:**177-184.

276. Serebrakian AT, Ortiz R, Christensen JM, Pickrell BB, Irwin TJ, Karinja SJ, Broyles JM, Liao EC, Eberlin KR, Helliwell LA: **Webinar during COVID-19 Improves Knowledge of Changes to the Plastic Surgery Residency Application Process.** *Plast Reconstr Surg Glob Open* 2020, **8:**e3247.

277. Shah NL, Miller JB, Bilal M, Shah B: **Smartphone Apps in Graduate Medical Education Virtual Recruitment During the COVID-19 Pandemic.** *J Med Syst* 2021, **45:**36.

278. Shah S, Castro-Dominguez Y, Gupta T, Attaran R, Byrum GV, 3rd, Taleb A, Pettyjohn A, Bartel RC, Szerlip M, Henry TD, et al: **Impact of the COVID-19 pandemic on interventional cardiology training in the United States.** *Catheter Cardiovasc Interv* 2020, **96:**997-1005.

279. Shahrvini B, Baxter SL, Coffey CS, MacDonald BV, Lander L: **Pre-clinical remote undergraduate medical education during the COVID-19 pandemic: a survey study.** *BMC Med Educ* 2021, **21:**13.

280. Sharara-Chami R, Sabouneh R, Zeineddine R, Banat R, Fayad J, Lakissian Z: **In Situ Simulation: An Essential Tool for Safe Preparedness for the COVID-19 Pandemic.** *Simul Healthc* 2020, **15:**303-309.

281. Sharma K ea: **Online learning in the face of COVID-19 pandemic: Assessment of students’ satisfaction at Chitwan medical college of Nepal.** *Kathmandu University Medical Journal*.

282. Sharma S, Ray A, Sadasivam B: **Adaptations in teaching modalities for medical undergraduates in response to coronavirus disease 2019 at a single teaching institute in India.** *Korean J Med Educ* 2020, **32:**291-295.

283. Shaw KS ea: **Exchanging Dermatoscopes for Stethoscopes: Has the COVID-19 Pandemic Highlighted Gaps in US Dermatology Residency Training?** *J Drugs Dermatol*.

284. Shawaqfeh MS, Al Bekairy AM, Al-Azayzih A, Alkatheri AA, Qandil AM, Obaidat AA, Al Harbi S, Muflih SM: **Pharmacy Students Perceptions of Their Distance Online Learning Experience During the COVID-19 Pandemic: A Cross-Sectional Survey Study.** *J Med Educ Curric Dev* 2020, **7:**2382120520963039.

285. Shebrain S, Nava K, Munene G, Shattuck C, Collins J, Sawyer R: **Virtual Surgery Oral Board Examinations in the Era of COVID-19 Pandemic. How I Do It!** *J Surg Educ* 2021, **78:**740-745.

286. Sheridan GA, Hughes AJ, Quinlan JF, Sheehan E, O'Byrne JM: **Quantifying the impact of the COVID-19 pandemic on orthopaedic trainees: a national perspective.** *Bone Jt Open* 2020, **1:**645-652.

287. Shetty S, Shilpa C, Dey D, Kavya S: **Academic Crisis During COVID 19: Online Classes, a Panacea for Imminent Doctors.** *Indian J Otolaryngol Head Neck Surg* 2022, **74:**45-49.

288. Shi D, Lu H, Wang H, Bao S, Qian L, Dong X, Tao K, Xu Z: **A simulation training course for family medicine residents in China managing COVID-19.** *Aust J Gen Pract* 2020, **49:**364-368.

289. Shih G, Deer JD, Lau J, Loveland Baptist L, Lim DJ, Lockman JL: **The impact of the COVID-19 pandemic on the education and wellness of U.S. Pediatric Anesthesiology Fellows.** *Paediatr Anaesth* 2021, **31:**268-274.

290. Shin TH, Klingler M, Han A, Mocsiran JL, Vilchez V, Naples R, French J, Lipman JM, Rosenblatt S: **Efficacy of Virtual Case-Based General Surgery Clerkship Curriculum During COVID-19 Distancing.** *Med Sci Educ* 2021, **31:**101-108.

291. Shrestha RM ea: **Online education status at dental colleges during COVID-19 pandemic in Nepal.** *Kathmandu University Medical Journal (KUMJ)*.

292. Shrestha A, Shrestha A, Sonnenberg T, Shrestha R: **COVID-19 Emergency Department Protocols: Experience of Protocol Implementation Through in-situ Simulation.** *Open Access Emerg Med* 2020, **12:**293-303.

293. Joshi SK, Sharma SC, Manandhar N, Shrestha MV: **Gaming Disorder among Medical College Students during COVID-19 Pandemic Lockdown.** *Kathmandu University Medical Journal* 2020, **18:**48-52.

294. Siau K, Iacucci M, Dunckley P, Penman I, EndoTrain Survey C: **The Impact of COVID-19 on Gastrointestinal Endoscopy Training in the United Kingdom.** *Gastroenterology* 2020, **159:**1582-1585 e1583.

295. Sielicki A, White J, Berman M, Lao B, Stobart-Gallagher M: **Homeschooling for Quarantined Residents: A Virtual Back to the Basics Curriculum.** *Cureus* 2020, **12:**e11824.

296. Silva N, Laiginhas R, Meireles A, Barbosa Breda J: **Impact of the COVID-19 Pandemic on Ophthalmology Residency Training in Portugal.** *Acta Med Port* 2020, **33:**640-648.

297. Sindiani AM, Obeidat N, Alshdaifat E, Elsalem L, Alwani MM, Rawashdeh H, Fares AS, Alalawne T, Tawalbeh LI: **Distance education during the COVID-19 outbreak: A cross-sectional study among medical students in North of Jordan.** *Ann Med Surg (Lond)* 2020, **59:**186-194.

298. Singal A, Bansal A, Chaudhary P, Singh H, Patra A: **Anatomy education of medical and dental students during COVID-19 pandemic: a reality check.** *Surg Radiol Anat* 2021, **43:**515-521.

299. Singhi EK, Dupuis MM, Ross JA, Rieber AG, Bhadkamkar NA: **Medical Hematology/Oncology Fellows' Perceptions of Online Medical Education During the COVID-19 Pandemic.** *J Cancer Educ* 2020, **35:**1034-1040.

300. Singla VK, Jain S, Ganeshan R, Rosenfeld LE, Enriquez AD: **The Impact of the COVID-19 Pandemic on Cardiac Electrophysiology Training: A Survey Study.** *J Cardiovasc Electrophysiol* 2021, **32:**9-15.

301. Slivkoff MD, Johnson C, Tackett S: **First-Year Medical Student Experiences Adjusting to the Immediate Aftermath of COVID-19.** *Med Sci Educ* 2021, **31:**557-564.

302. Smith E, Boscak A: **A virtual emergency: learning lessons from remote medical student education during the COVID-19 pandemic.** *Emerg Radiol* 2021, **28:**445-452.

303. Smith EB, Boscak A, Friedman EM, Frand S, Deitte LA, Benefield T, Jordan S: **Radiology Medical Student Education 2020: Surveys of the Alliance of Medical Student Educators in Radiology and Medical Students.** *Acad Radiol* 2022, **29:**298-311.

304. Son HK: **Effects of S-PBL in Maternity Nursing Clinical Practicum on Learning Attitude, Metacognition, and Critical Thinking in Nursing Students: A Quasi-Experimental Design.** *Int J Environ Res Public Health* 2020, **17**.

305. Sponchiado Junior EC ea: **Impact of COVID-19 on dental education in Brazil.** *Open Science Framework*.

306. Srinivasan DK: **Medical Students' Perceptions and an Anatomy Teacher's Personal Experience Using an e-Learning Platform for Tutorials During the Covid-19 Crisis.** *Anat Sci Educ* 2020, **13:**318-319.

307. Srivastava S, Jacob J, Charles AS, Daniel P, Mathew JK, Shanthi P, Devamani K, Mahasampath G, Rabi S: **Emergency remote learning in anatomy during the COVID-19 pandemic: A study evaluating academic factors contributing to anxiety among first year medical students.** *Med J Armed Forces India* 2021, **77:**S90-S98.

308. Stamelou M, Struhal W, Ten Cate O, Matczak M, Caliskan SA, Soffietti R, Marson A, Zis P, di Lorenzo F, Sander A, et al: **Evaluation of the 2020 European Academy of Neurology virtual congress: transition from a face-to-face to a virtual meeting.** *Eur J Neurol* 2021, **28:**2523-2532.

309. Steehler AJ, Pettitt-Schieber B, Studer MB, Mahendran G, Pettitt BJ, Henriquez OA: **Implementation and Evaluation of a Virtual Elective in Otolaryngology in the Time of COVID-19.** *Otolaryngol Head Neck Surg* 2021, **164:**556-561.

310. Stout RC, Roberts S, Maxwell-Scott H, Gothard P: **Necessity is the mother of invention: how the COVID-19 pandemic could change medical student placements for the better.** *Postgrad Med J* 2021, **97:**417-422.

311. Suppan L, Stuby L, Gartner B, Larribau R, Iten A, Abbas M, Harbarth S, Suppan M: **Impact of an e-learning module on personal protective equipment knowledge in student paramedics: a randomized controlled trial.** *Antimicrob Resist Infect Control* 2020, **9:**185.

312. Suppan M, Stuby L, Carrera E, Cottet P, Koka A, Assal F, Savoldelli GL, Suppan L: **Asynchronous Distance Learning of the National Institutes of Health Stroke Scale During the COVID-19 Pandemic (E-Learning vs Video): Randomized Controlled Trial.** *J Med Internet Res* 2021, **23:**e23594.

313. Swaminathan N, Govindharaj P, Jagadeesh NS, Ravichandran L: **Evaluating the effectiveness of an online faculty development programme for nurse educators about remote teaching during COVID-19.** *J Taibah Univ Med Sci* 2021, **16:**268-273.

314. Swiatek PR, Weiner JA, Butler BA, McCarthy MH, Louie PK, Wolinsky JP, Hsu WK, Patel AA: **Assessing the Early Impact of the COVID-19 Pandemic on Spine Surgery Fellowship Education.** *Clin Spine Surg* 2021, **34:**E186-E193.

315. Tahir H ea: **The reduction in clinical and surgical exposure of trainees during COVID-19 and its impact on their training.** *J Pak Med Assoc*.

316. Tang PY, New LM, Leow WQ: **Zooming for cells: Tele-education of histopathology residents during the COVID-19 pandemic.** *Proceedings of Singapore Healthcare* 2020, **30:**71-75.

317. Terhune KP, Choi JN, Green JM, Hildreth AN, Lipman JM, Aarons CB, Heyduk DA, Misra S, Anand RJ, Fise TF, et al: **Ad astra per aspera (Through Hardships to the Stars): Lessons Learned from the First National Virtual APDS Meeting, 2020.** *J Surg Educ* 2020, **77:**1465-1472.

318. Thakre SS, Jadhao AR, Dhoble MA, Dass R, Thakre SB, Somani A: **Evaluation of Effectiveness of COVID-19 Training and Assessment of Anxiety among Nurses of a Tertiary Health Care Centre during the Corona Virus Pandemic- An Experimental Study.** *Journal of Clinical and Diagnostic Research* 2020.

319. Tinelli G, Sica S, Minelli F, Tshomba Y: **Vascular surgery education during COVID-19 pandemic.** *J Vasc Surg* 2020, **72:**763-764.

320. GÜÇHan Topcu Z, Belgen Kaygisiz B, Demiralp C: **How are the physical activity and anxiety levels of the university students affected during the coronavirus (Covid-19) pandemic?** *Baltic Journal of Health and Physical Activity* 2020, **Supplement 1:**33-40.

321. Torun F, Torun SD: **The psychological impact of the COVID-19 pandemic on medical students in Turkey.** *Pak J Med Sci* 2020, **36:**1355-1359.

322. Treluyer L, Tourneux P: **Burnout among paediatric residents during the COVID-19 outbreak in France.** *Eur J Pediatr* 2021, **180:**627-633.

323. Trujillo Loli Y, D'Carlo Trejo Huaman M, Campos Medina S: **Telementoring of in-home real-time laparoscopy using whatsapp messenger: An innovative teaching tool during the COVID-19 pandemic. A cohort study.** *Ann Med Surg (Lond)* 2021, **62:**481-484.

324. Tuma F, Nassar AK, Kamel MK, Knowlton LM, Jawad NK: **Students and faculty perception of distance medical education outcomes in resource-constrained system during COVID-19 pandemic. A cross-sectional study.** *Ann Med Surg (Lond)* 2021, **62:**377-382.

325. Turner S, Pham T, Robledo K, Turner S, Brown C, Sundaresan P: **Rapid Adaptation of Cancer Education in Response to the COVID-19 Pandemic: Evaluation of a Live Virtual Statistics and Research Skills Workshop for Oncology Trainees.** *J Cancer Educ* 2022, **37:**905-910.

326. Ungureanu BS, Vladut C, Bende F, Sandru V, Tocia C, Turcu-Stiolica RA, Groza A, Balan GG, Turcu-Stiolica A: **Impact of the COVID-19 Pandemic on Health-Related Quality of Life, Anxiety, and Training Among Young Gastroenterologists in Romania.** *Front Psychol* 2020, **11:**579177.

327. Unnikrishnan VV ea: **Online Teaching in Ayurveda Medical Education during COVID 19 Pandemic - A Descriptive Survey Study.** *International Journal of Ayurvedic Medicine*.

328. Upadhyaya GK, Jain VK, Iyengar KP, Patralekh MK, Vaish A: **Impact of COVID-19 on post-graduate orthopaedic training in Delhi-NCR.** *J Clin Orthop Trauma* 2020, **11:**S687-S695.

329. Vahedian-Azimi A, Moayed MS, Rahimibashar F, Shojaei S, Ashtari S, Pourhoseingholi MA: **Comparison of the severity of psychological distress among four groups of an Iranian population regarding COVID-19 pandemic.** *BMC Psychiatry* 2020, **20:**402.

330. Vala N, Vachhani M, Sorani A: **Study of anxiety, stress, and depression level among medical students during COVID-19 pandemic phase in Jamnagar city.** *National Journal of Physiology, Pharmacy and Pharmacology* 2020.

331. Vala N, Vachhani M, Sorani A: **Study of evaluation of e-learning classes among medical students during COVID-19 pandemic phase in Jamnagar city.** *National Journal of Physiology, Pharmacy and Pharmacology* 2020.

332. van der Keylen P, Lippert N, Kunisch R, Kuhlein T, Roos M: **Asynchronous, digital teaching in times of COVID-19: a teaching example from general practice.** *GMS J Med Educ* 2020, **37:**Doc98.

333. Verma A, Verma S, Garg P, Godara R: **Online Teaching During COVID-19: Perception of Medical Undergraduate Students.** *Indian J Surg* 2020, **82:**299-300.

334. Vielsmeier V, Auerswald S, Marienhagen J, Keil S, Muller N: **Digital teaching with interactive case presentations of ENT diseases - discussion of utilisation and motivation of students.** *GMS J Med Educ* 2020, **37:**Doc100.

335. Walton R, Greenberg A, Ehlke D, Sola O: **Development of a Health Policy Elective for Medical Students During the COVID-19 Pandemic: A Pilot Study.** *PRiMER* 2020, **4:**29.

336. Wang K, Zhang L, Ye L: **A nationwide survey of online teaching strategies in dental education in China.** *J Dent Educ* 2021, **85:**128-134.

337. Wang Y, Li Y, Jiang J, Feng Y, Lu D, Zhang W, Song H: **COVID-19 outbreak-related psychological distress among healthcare trainees: a cross-sectional study in China.** *BMJ Open* 2020, **10:**e041671.

338. Warnica W, Moody A, Probyn L, Bartlett E, Singh N, Pakkal M: **Lessons Learned From the Effects of COVID-19 on the Training and Education Workflow of Radiology Residents-A Time for Reflection: Perspectives of Residency Program Directors and Residents in Canada.** *Can Assoc Radiol J* 2021, **72:**637-644.

339. Weber W, Ahn J: **COVID-19 Conferences: Resident Perceptions of Online Synchronous Learning Environments.** *West J Emerg Med* 2020, **22:**115-118.

340. Webster TL ea: **COVID-19 Planning Among Radiologic Science Programs: Response Mitigation Activities.** *Radiol Technol*.

341. Wells J, Higbee D, Doty J, Louder E: **Avoiding Fumbles: Online Patient Handoff Training.** *PRiMER* 2020, **4:**32.

342. White EM, Shaughnessy MP, Esposito AC, Slade MD, Korah M, Yoo PS: **Surgical Education in the Time of COVID: Understanding the Early Response of Surgical Training Programs to the Novel Coronavirus Pandemic.** *J Surg Educ* 2021, **78:**412-421.

343. Widiyanto A ea: **The effect of social media exposure on depression and anxiety disorders in facing Covid-19 pandemic.** *European Journal of Molecular and Clinical Medicine*.

344. Williams C, Familusi OO, Ziemba J, Lee D, Mittal S, Mucksavage P, Smith A, Kovell RC: **Adapting to the Educational Challenges of a Pandemic: Development of a Novel Virtual Urology Subinternship During the Time of COVID-19.** *Urology* 2021, **148:**70-76.

345. Wittayanakorn N, Nga VDW, Sobana M, Bahuri NFA, Baticulon RE: **Impact of COVID-19 on Neurosurgical Training in Southeast Asia.** *World Neurosurg* 2020, **144:**e164-e177.

346. Wong A: **Effectiveness of Online Teaching During COVID-19 Pandemic for West Midlands Urology Trainees.** *Journal of Endoluminal Endourology* 2020, **3:**e25-e31.

347. Wyles SP, Meyer FB, Hayden R, Scarisbrick I, Terzic A: **Digital regenerative medicine and surgery pedagogy for virtual learning in the time of COVID-19.** *Regen Med* 2020, **15:**1937-1941.

348. Xie J, Li X, Luo H, He L, Bai Y, Zheng F, Zhang L, Ma J, Niu Z, Qin Y, et al: **Depressive Symptoms, Sleep Quality and Diet During the 2019 Novel Coronavirus Epidemic in China: A Survey of Medical Students.** *Front Public Health* 2020, **8:**588578.

349. Yagci I, Sarikaya S, Ayhan FF, Bahsi A, Bilir Kaya B, Erhan B, Dundar Ahi E, Okan S, Ozkan Y, Korkmaz MD, et al: **The effects of COVID-19 on Physical Medicine and Rehabilitation in Turkey in the first month of pandemic.** *Turk J Phys Med Rehabil* 2020, **66:**244-251.

350. Yadala S, Nalleballe K, Sharma R, Lotia M, Kapoor N, Veerapaneni KD, Kovvuru S, Onteddu S: **Resident Education During COVID-19 Pandemic: Effectiveness of Virtual Electroencephalogram Learning.** *Cureus* 2020, **12:**e11094.

351. Yan M, Cantwell SR, Mason M, Mardini S, Bakri K, Harless CA: **Impact of COVID-19 on a plastic surgery residency education program: Outcomes of a survey.** *J Plast Reconstr Aesthet Surg* 2021, **74:**644-710.

352. Yang T, Buck S, Evans L, Auerbach M: **A Telesimulation Elective to Provide Medical Students With Pediatric Patient Care Experiences During the COVID Pandemic.** *Pediatr Emerg Care* 2021, **37:**119-122.

353. Ye W, Ye X, Liu Y, Liu Q, Vafaei S, Gao Y, Yu H, Zhong Y, Zhan C: **Effect of the Novel Coronavirus Pneumonia Pandemic on Medical Students' Psychological Stress and Its Influencing Factors.** *Front Psychol* 2020, **11:**548506.

354. Yu NZ, Li ZJ, Chong YM, Xu Y, Fan JP, Yang Y, Teng Y, Zhang YW, Zhang WC, Zhang MZ, et al: **Chinese medical students' interest in COVID-19 pandemic.** *World J Virol* 2020, **9:**38-46.

355. Yu Z, Moustafa D, Kwak R, O'Connor DM, Cavanaugh-Hussey MW, Huang JT, LaChance AH: **Engaging in advocacy during medical training: assessing the impact of a virtual COVID-19-focused state advocacy day.** *Postgrad Med J* 2022, **98:**365-368.

356. Yuriditsky E, Horowitz JM, Nair S, Kaufman BS: **Simulation-based uptraining improves provider comfort in the management of critically ill patients with COVID-19.** *J Crit Care* 2021, **61:**14-17.

357. Zhang Q, He YJ, Zhu YH, Dai MC, Pan MM, Wu JQ, Zhang X, Gu YE, Wang FF, Xu XR, Qu F: **The evaluation of online course of Traditional Chinese Medicine for MBBS international students during the COVID-19 epidemic period.** *Integr Med Res* 2020, **9:**100449.

358. Zheng J, Hundeyin M, He K, Sachs T, Hess DT, Whang E, Kristo G: **General surgery chief residents' perspective on surgical education during the coronavirus disease 2019 (COVID-19) pandemic.** *Surgery* 2020, **168:**222-225.

359. Zhi X ea: **Investigation and analysis of psychological stress and professional identity of nursing students during COVID-19 pandemic.** *Indian Journal of Experimental Biology*.

360. Zhou M, Yuan F, Zhao X, Xi F, Wen X, Zeng L, Zeng W, Wu H, Zeng H, Zhao Z: **Research on the individualized short‐term training model of nurses in emergency isolation wards during the outbreak of COVID‐19.** *Nursing Open* 2020, **7:**1902-1908.

361. Zis P, Artemiadis A, Bargiotas P, Nteveros A, Hadjigeorgiou GM: **Medical Studies during the COVID-19 Pandemic: The Impact of Digital Learning on Medical Students' Burnout and Mental Health.** *Int J Environ Res Public Health* 2021, **18**.

362. Zoia C, Raffa G, Somma T, Della Pepa GM, La Rocca G, Zoli M, Bongetta D, De Divitiis O, Fontanella MM: **COVID-19 and neurosurgical training and education: an Italian perspective.** *Acta Neurochir (Wien)* 2020, **162:**1789-1794.

363. Carrascosa MMC, Campos T, Sampaio JE, Souza RRF, Ribeiro VL, Maia MLN, Gama LCL, Severino MP, Semer NK, Rondon O, et al: **Medical Interns and COVID-19: results of national research.** *Rev Assoc Med Bras (1992)* 2020, **66:**812-817.

364. Carroll T, Mooney C, Horowitz R: **Re-ACT: Remote Advanced Communication Training in a Time of Crisis.** *J Pain Symptom Manage* 2021, **61:**364-368.

365. Carson S, Peraza LR, Pucci M, Huynh J: **Student Hotline Improves Remote Clinical Skills and Access to Rural Care.** *PRiMER* 2020, **4:**22.

366. Caruana EJ, Patel A, Kendall S, Rathinam S: **Impact of coronavirus 2019 (COVID-19) on training and well-being in subspecialty surgery: A national survey of cardiothoracic trainees in the United Kingdom.** *J Thorac Cardiovasc Surg* 2020, **160:**980-987.

367. Caruso MC: **Virtual Microscopy and Other Technologies for Teaching Histology During Covid-19.** *Anat Sci Educ* 2021, **14:**19-21.

368. Casafont C, Fabrellas N, Rivera P, Olive-Ferrer MC, Querol E, Venturas M, Prats J, Cuzco C, Frias CE, Perez-Ortega S, Zabalegui A: **Experiences of nursing students as healthcare aid during the COVID-19 pandemic in Spain: A phemonenological research study.** *Nurse Educ Today* 2021, **97:**104711.

369. Castro-Sanchez E, Alexander CM, Atchison C, Patel D, Leung W, Calamita ME, Meno Garcia D, Cimpeanu C, Mumbwatasai JM, Ramid D, et al: **Evaluation of a personal protective equipment support programme for staff during the COVID-19 pandemic in London.** *J Hosp Infect* 2021, **109:**68-77.

370. Cates AL, Krueger J, Simpson SE, Stobart-Gallagher M: **Comparing the Effectiveness of a Virtual Toxicology Escape Room at Two Emergency Medicine Residencies.** *Cureus* 2020, **12:**e11262.

371. Chakraborty T, Subbiah GK, Damade Y: **Psychological Distress during COVID-19 Lockdown among Dental Students and Practitioners in India: A Cross-Sectional Survey.** *Eur J Dent* 2020, **14:**S70-S78.

372. Chandrasinghe PC, Siriwardana RC, Kumarage SK, Munasinghe BNL, Weerasuriya A, Tillakaratne S, Pinto D, Gunathilake B, Fernando FR: **A novel structure for online surgical undergraduate teaching during the COVID-19 pandemic.** *BMC Med Educ* 2020, **20:**324.

373. Chang DG, Park JB, Baek GH, Kim HJ, Bosco A, Hey HWD, Lee CK: **The impact of COVID-19 pandemic on orthopaedic resident education: a nationwide survey study in South Korea.** *Int Orthop* 2020, **44:**2203-2210.

374. Chatziralli I, Ventura CV, Touhami S, Reynolds R, Nassisi M, Weinberg T, Pakzad-Vaezi K, Anaya D, Mustapha M, Plant A, et al: **Transforming ophthalmic education into virtual learning during COVID-19 pandemic: a global perspective.** *Eye (Lond)* 2021, **35:**1459-1466.

375. Chaudhuri A, Paul S, Mondal T, Goswami A: **Online teaching-learning experience among medical students in a developing country during the coronavirus disease-19 pandemic: A pilot study.** *National Journal of Physiology, Pharmacy and Pharmacology* 2020.

376. Chaurasia AR, Page BR, Walker AJ, Salerno K, Camphausen K, Kwok Y, Bajaj GK, Ambrocio D, Erickson D: **Lessons to Learn From a Successful Virtual Mock Oral Examination Pilot Experience.** *Adv Radiat Oncol* 2021, **6:**100534.

377. Chawlowska E, Staszewski R, Lipiak A, Giernas B, Karasiewicz M, Bazan D, Nowosadko M, Cofta M, Wysocki J: **Student Volunteering as a Solution for Undergraduate Health Professions Education: Lessons From the COVID-19 Pandemic.** *Front Public Health* 2020, **8:**633888.

378. Chee J, Lin X, Lim WS, Loh WS, Thong M, Ng L: **Using 3D-printed nose models in nasopharyngeal swab training.** *Oral Oncol* 2021, **113:**105033.

379. Chen E, Kaczmarek K, Ohyama H: **Student perceptions of distance learning strategies during COVID-19.** *J Dent Educ* 2020.

380. Cheriyan A, Kumar S: **Impact of COVID-19 on urology residency in India - Results of a nationwide survey.** *Indian J Urol* 2020, **36:**243-245.

381. Cheserem JB, Esene IN, Mahmud MR, Kalangu K, Sanoussi S, Musara A, El-Ghandour NMF, Fieggen G, Qureshi M: **A Continental Survey on the Impact of COVID-19 on Neurosurgical Training in Africa.** *World Neurosurg* 2021, **147:**e8-e15.

382. Cheung VK, So EH, Ng GW, So SS, Hung JL, Chia NH: **Investigating effects of healthcare simulation on personal strengths and organizational impacts for healthcare workers during COVID-19 pandemic: A cross-sectional study.** *Integr Med Res* 2020, **9:**100476.

383. Chew QH, Chia FL, Ng WK, Lee WCI, Tan PLL, Wong CS, Puah SH, Shelat VG, Seah ED, Huey CWT, et al: **Perceived Stress, Stigma, Traumatic Stress Levels and Coping Responses amongst Residents in Training across Multiple Specialties during COVID-19 Pandemic-A Longitudinal Study.** *Int J Environ Res Public Health* 2020, **17**.

384. Chhabra N, Winfield A, Dyer S, Hedayati T: **Mock Oral Board Examinations via Web-based Video Teleconferencing in the Era of COVID-19.** *AEM Educ Train* 2021, **5:**116-119.

385. Chin KE, Kwon D, Gan Q, Ramalingam PX, Wistuba, II, Prieto VG, Aung PP: **Transition From a Standard to a Hybrid On-Site and Remote Anatomic Pathology Training Model During the Coronavirus Disease 2019 (COVID-19) Pandemic.** *Arch Pathol Lab Med* 2021, **145:**22-31.

386. Chiou PZ: **Learning cytology in times of pandemic: an educational institutional experience with remote teaching.** *J Am Soc Cytopathol* 2020, **9:**579-585.

387. Chiu CY, Sarwal A, Jawed M, Chemarthi VS, Shabarek N: **Telemedicine experience of NYC Internal Medicine residents during COVID-19 pandemic.** *PLoS One* 2021, **16:**e0246762.

388. Cho MJ, Hong JP: **The emergence of virtual education during the COVID-19 pandemic: The past, present, and future of the plastic surgery education.** *J Plast Reconstr Aesthet Surg* 2021, **74:**1413-1421.

389. Choi B, Jegatheeswaran L, Minocha A, Alhilani M, Nakhoul M, Mutengesa E: **The impact of the COVID-19 pandemic on final year medical students in the United Kingdom: a national survey.** *BMC Med Educ* 2020, **20:**206.

390. Chopra A ea: **Perception of dental students towards online classes.** *European Journal of Molecular and Clinical Medicine*.

391. Chou DW, Staltari G, Mullen M, Chang J, Durr M: **Otolaryngology Resident Wellness, Training, and Education in the Early Phase of the COVID-19 Pandemic.** *Ann Otol Rhinol Laryngol* 2021, **130:**904-914.

392. Christensen L, Rasmussen CS, Benfield T, Franc JM: **A Randomized Trial of Instructor-Led Training Versus Video Lesson in Training Health Care Providers in Proper Donning and Doffing of Personal Protective Equipment.** *Disaster Med Public Health Prep* 2020, **14:**514-520.

393. Civantos AM, Byrnes Y, Chang C, Prasad A, Chorath K, Poonia SK, Jenks CM, Bur AM, Thakkar P, Graboyes EM, et al: **Mental health among otolaryngology resident and attending physicians during the COVID-19 pandemic: National study.** *Head Neck* 2020, **42:**1597-1609.

394. Claps F, Amparore D, Esperto F, Cacciamani G, Fiori C, Minervini A, Liguori G, Trombetta C, Porpiglia F, Serni S, et al: **Smart learning for urology residents during the COVID-19 pandemic and beyond: insights from a nationwide survey in Italy.** *Minerva Urol Nefrol* 2020, **72:**647-649.

395. Clarke K, Bilal M, Sanchez-Luna SA, Dalessio S, Maranki JL, Siddique SM: **Impact of COVID-19 Pandemic on Training: Global Perceptions of Gastroenterology and Hepatology Fellows in the USA.** *Dig Dis Sci* 2021, **66:**3307-3311.

396. Co M, Chung PH, Chu KM: **Online teaching of basic surgical skills to medical students during the COVID-19 pandemic: a case-control study.** *Surg Today* 2021, **51:**1404-1409.

397. Coffey CS, MacDonald BV, Shahrvini B, Baxter SL, Lander L: **Student Perspectives on Remote Medical Education in Clinical Core Clerkships During the COVID-19 Pandemic.** *Med Sci Educ* 2020, **30:**1577-1584.

398. Coleman JR, Abdelsattar JM, Glocker RJ, Force R-AC-T: **COVID-19 Pandemic and the Lived Experience of Surgical Residents, Fellows, and Early-Career Surgeons in the American College of Surgeons.** *J Am Coll Surg* 2021, **232:**119-135 e120.

399. Collado-Boira EJ, Ruiz-Palomino E, Salas-Media P, Folch-Ayora A, Muriach M, Balino P: **"The COVID-19 outbreak"-An empirical phenomenological study on perceptions and psychosocial considerations surrounding the immediate incorporation of final-year Spanish nursing and medical students into the health system.** *Nurse Educ Today* 2020, **92:**104504.

400. Collins C, Mahuron K, Bongiovanni T, Lancaster E, Sosa JA, Wick E: **Stress and the Surgical Resident in the COVID-19 Pandemic.** *J Surg Educ* 2021, **78:**422-430.

401. Conroy S, McDonald D: **Rapid multi-professional training for COVID-19 in rural hospitals.** *Aust J Rural Health* 2020, **28:**618-619.

402. Costabile M: **Using online simulations to teach biochemistry laboratory content during COVID-19.** *Biochem Mol Biol Educ* 2020, **48:**509-510.

403. Cowart K, Updike WH: **Pharmacy student perception of a remote hypertension and drug information**

**simulation‐based**

**learning experience in response to the**

**SARS‐CoV**

**‐2 pandemic.** *Jaccp: Journal of the American College of Clinical Pharmacy* 2020, **4:**53-59.

404. Coyle C, Ghazi H, Georgiou I: **The mental health and well-being benefits of exercise during the COVID-19 pandemic: a cross-sectional study of medical students and newly qualified doctors in the UK.** *Ir J Med Sci* 2021, **190:**925-926.

405. Cravero AL, Kim NJ, Feld LD, Berry K, Rabiee A, Bazarbashi N, Bassin S, Lee TH, Moon AM, Qi X, et al: **Impact of exposure to patients with COVID-19 on residents and fellows: an international survey of 1420 trainees.** *Postgrad Med J* 2021, **97:**706-715.

406. Cuffaro L, Carvalho V, Di Liberto G, Klinglehoefer L, Sauerbier A, Garcia-Azorin D, Tabuas-Pereira M, Vashchenko N, Moro E, Bassetti CLA: **Neurology training and research in the COVID-19 pandemic: a survey of the Resident and Research Fellow Section of the European Academy of Neurology.** *Eur J Neurol* 2021, **28:**3437-3442.

407. Cuschieri S, Calleja Agius J: **Spotlight on the Shift to Remote Anatomical Teaching During Covid-19 Pandemic: Perspectives and Experiences from the University of Malta.** *Anat Sci Educ* 2020, **13:**671-679.

408. Hoegger MJ, Shetty AS, Denner DR, Gould JE, Wahl RL, Raptis CA, Ballard DH: **A Snapshot of Radiology Training During the Early COVID-19 Pandemic.** *Curr Probl Diagn Radiol* 2021, **50:**607-613.

409. Homer NA, Epstein A, Somogyi M, Shore JW: **Oculoplastic fellow education during the COVID-19 crisis.** *Orbit* 2022, **41:**79-83.

410. Hu H, Xiao Y, Li H: **The Effectiveness of a Serious Game Versus Online Lectures for Improving Medical Students' Coronavirus Disease 2019 Knowledge.** *Games Health J* 2021, **10:**139-144.

411. Hu L, Wu H, Zhou W, Shen J, Qiu W, Zhang R, Wu J, Chai Y: **Positive impact of COVID-19 on career choice in pediatric medical students: a longitudinal study.** *Transl Pediatr* 2020, **9:**243-252.

412. Huamanchumo-Suyon ME, Urrunaga-Pastor D, Ruiz-Perez PJ, Rodrigo-Gallardo PK, Toro-Huamanchumo CJ: **Impact of the COVID-19 pandemic on general surgery residency program in Peru: A cross-sectional study.** *Ann Med Surg (Lond)* 2020, **60:**130-134.

413. Huang L, Lei W, Xu F, Liu H, Yu L: **Emotional responses and coping strategies in nurses and nursing students during Covid-19 outbreak: A comparative study.** *PLoS One* 2020, **15:**e0237303.

414. Huffman EM, Athanasiadis DI, Anton NE, Haskett LA, Doster DL, Stefanidis D, Lee NK: **How resilient is your team? Exploring healthcare providers' well-being during the COVID-19 pandemic.** *Am J Surg* 2021, **221:**277-284.

415. Hughes T, Beard E, Bowman A, Chan J, Gadsby K, Hughes M, Humphries M, Johnston A, King G, Knock M, et al: **Medical student support for vulnerable patients during COVID-19 - a convergent mixed-methods study.** *BMC Med Educ* 2020, **20:**377.

416. Hung M, Licari FW, Hon ES, Lauren E, Su S, Birmingham WC, Wadsworth LL, Lassetter JH, Graff TC, Harman W, et al: **In an era of uncertainty: Impact of COVID-19 on dental education.** *J Dent Educ* 2021, **85:**148-156.

417. Huntley RE, Ludwig DC, Dillon JK: **Early Effects of COVID-19 on Oral and Maxillofacial Surgery Residency Training-Results From a National Survey.** *J Oral Maxillofac Surg* 2020, **78:**1257-1267.

418. Hussain R, Singh B, Shah N, Jain S: **Impact of COVID-19 on ophthalmic specialist training in the United Kingdom-the trainees' perspective.** *Eye (Lond)* 2020, **34:**2157-2160.

419. Hytonen H, Napankangas R, Karaharju-Suvanto T, Evasoja T, Kallio A, Kokkari A, Tuononen T, Lahti S: **Modification of national OSCE due to COVID-19 - Implementation and students' feedback.** *Eur J Dent Educ* 2021, **25:**679-688.

420. Ibrahim NK, Al Raddadi R, AlDarmasi M, Al Ghamdi A, Gaddoury M, AlBar HM, Ramadan IK: **Medical students' acceptance and perceptions of e-learning during the Covid-19 closure time in King Abdulaziz University, Jeddah.** *J Infect Public Health* 2021, **14:**17-23.

421. Ilonzo N, Koleilat I, Prakash V, Charitable J, Garg K, Han D, Faries P, Phair J: **The Effect of COVID-19 on Training and Case Volume of Vascular Surgery Trainees.** *Vasc Endovascular Surg* 2021, **55:**429-433.

422. Imai TA, Soukiasian HJ, Truong A, Chau V, Amersi F: **The lasting footprint of COVID-19 on surgical education: A resident and attending perspective on the global pandemic.** *Am J Surg* 2021, **222:**473-480.

423. Irastorza LE, Hopson P, Ta A, Kemme S, Mallon D, Lee CK: **The Impact of COVID-19 on Job Prospects and Educational Training for Pediatric Gastroenterology Fellows.** *J Pediatr Gastroenterol Nutr* 2021, **72:**514-519.

424. Isiekwe IG, Umeizudike KA, Daramola OO, Akeredolu MO, Leo-Olagbaye AA: **The COVID-19 pandemic and dental residency training in Nigeria.** *Eur J Dent Educ* 2021, **25:**753-761.

425. Iurcov R ea: **ASSESSING THE OPINION OF SENIOR STUDENTS ABOUT DENTAL EDUCATION DURING THE PANDEMIC PERIOD.** *Medical-Surgical Journal-Revista Medico-Chirurgicala*.

426. Ivanov V ea: **ONLINE DISTANCE EDUCATION ON WORDPRESS WEB BASED PLATFORM AS AN INNOVATION IN THE LEARNING PROCESS OF SPORTS STUDENTS FROM MEDICAL UNIVERSITY - SOFIA DURING THE PANDEMIC OF COVID-19.** *Pedagogika-Pedagogy*.

427. Izreig S, Torabi SJ, Kasle DA, Rahmati RW, Manes RP: **Otolaryngology Match 2020-21: Survey of Prospective Applicants in the Setting of COVID-19.** *Ann Otol Rhinol Laryngol* 2021, **130:**450-458.

428. Jaap A, Dewar A, Duncan C, Fairhurst K, Hope D, Kluth D: **Effect of remote online exam delivery on student experience and performance in applied knowledge tests.** *BMC Med Educ* 2021, **21:**86.

429. Jensen RD, Bie M, Gundso AP, Schmid JM, Juelsgaard J, Gamborg ML, Mainz H, Rolfing JD: **Preparing an orthopedic department for COVID-19.** *Acta Orthop* 2020, **91:**644-649.

430. Jiang X, Ning Q: **The impact and evaluation of COVID-19 pandemic on the teaching model of medical molecular biology course for undergraduates major in pharmacy.** *Biochem Mol Biol Educ* 2021, **49:**346-352.

431. Jimenez-Rodriguez D, Arrogante O: **Simulated Video Consultations as a Learning Tool in Undergraduate Nursing: Students' Perceptions.** *Healthcare (Basel)* 2020, **8**.

432. Johnson AP, Wohlauer MV, Mouawad NJ, Malgor RD, Coogan SM, Sheahan MG, 3rd, Singh N, Cuff RF, Woo K, Coleman DM, Shalhub S: **The Impact of the COVID-19 Pandemic on Vascular Surgery Trainees in the United States.** *Ann Vasc Surg* 2021, **72:**182-190.

433. Johnson J, Chung MT, Stathakios J, Gonik N, Siegel B: **The impact of the COVID-19 pandemic on fellowship training: A national survey of pediatric otolaryngology fellowship directors.** *Int J Pediatr Otorhinolaryngol* 2020, **136:**110217.

434. Jones K, Hein LC, James L: **A Nursing Leadership Practicum in the Time of COVID19:: A Southeastern University Experience.** *Nurse Lead* 2021, **19:**145-149.

435. Joshi A, Wasir A, Chelluri S: **Effect of nationwide lockdown due to coronavirus disease-19 pandemic on daily activities and study pattern of the 1st MBBS students.** *National Journal of Physiology, Pharmacy and Pharmacology* 2020.

436. Joshi P, Bodkha P: **A comparative evaluation of students' insight of face to face classroom lectures and virtual online lectures.** *National Journal of Physiology, Pharmacy and Pharmacology* 2021, **11**.

437. Jum'ah AA, Elsalem L, Loch C, Schwass D, Brunton PA: **Perception of health and educational risks amongst dental students and educators in the era of COVID-19.** *Eur J Dent Educ* 2021, **25:**506-515.

438. Junod Perron N, Dominice Dao M, Rieder A, Sommer J, Audetat MC: **Online Synchronous Clinical Communication Training During the Covid-19 Pandemic.** *Adv Med Educ Pract* 2020, **11:**1029-1036.

439. Kaczmarek K, Chen E, Ohyama H: **Distance learning in the COVID-19 era: Comparison of student and faculty perceptions.** *J Dent Educ* 2020.

440. Kahwash BM, Deshpande DR, Guo C, Panganiban CM, Wangberg H, Craig TJ: **Allergy/Immunology Trainee Experiences During the COVID-19 Pandemic: AAAAI Work Group Report of the Fellows-in-Training Committee.** *J Allergy Clin Immunol Pract* 2021, **9:**1-6 e1.

441. Kaliyadan F, ElZorkany K, Al Wadani F: **An Online Dermatology Teaching Module for Undergraduate Medical Students amidst the COVID-19 Pandemic: An Experience and Suggestions for the Future.** *Indian Dermatol Online J* 2020, **11:**944-947.

442. Kalkan Ugurlu Y, Mataraci Degirmenci D, Durgun H, Gok Ugur H: **The examination of the relationship between nursing students' depression, anxiety and stress levels and restrictive, emotional, and external eating behaviors in COVID-19 social isolation process.** *Perspect Psychiatr Care* 2021, **57:**507-516.

443. Kang KA, Kim SJ, Lee MN, Kim M, Kim S: **Comparison of Learning Effects of Virtual Reality Simulation on Nursing Students Caring for Children with Asthma.** *Int J Environ Res Public Health* 2020, **17**.

444. Kanniammal C ea: **A Study to Assess the Nursing Students Experience Regarding Online Classes During Covid-19 Lock Down Periods in SRM College of Nursing, Kattankulathur, Kancheepuram District-603203, Tamil Nadu, India.** *Medico-Legal Update* 2021.

445. Kapila AK, Farid Y, Kapila V, Schettino M, Vanhoeij M, Hamdi M: **The perspective of surgical residents on current and future training in light of the COVID-19 pandemic.** *Br J Surg* 2020, **107:**e305.

446. Kapila AK, Schettino M, Farid Y, Ortiz S, Hamdi M: **The Impact of Coronavirus Disease 2019 on Plastic Surgery Training: The Resident Perspective.** *Plast Reconstr Surg Glob Open* 2020, **8:**e3054.

447. Kapila V, Corthals S, Langhendries L, Kapila AK, Everaert K: **The importance of medical student perspectives on the impact of COVID-19.** *Br J Surg* 2020, **107:**e372-e373.

448. Karampekos G ea: **Differences between fellows and fellowship program directors in their perception of the impact of the covid-19 pandemic on gastroenterology training: Results from a nationwide survey in greece.** *Annals of Gastroenterology*.

449. Kasle DA, Torabi SJ, Izreig S, Rahmati RW, Manes RP: **COVID-19's Impact on the 2020-2021 Resident Match: A Survey of Otolaryngology Program Directors.** *Ann Otol Rhinol Laryngol* 2021, **130:**666-673.

450. Kaur N, Dwivedi D, Arora J, Gandhi A: **Study of the effectiveness of e-learning to conventional teaching in medical undergraduates amid COVID-19 pandemic.** *National Journal of Physiology, Pharmacy and Pharmacology* 2020, **10**.

451. Kealey A, Alam F, McCreath G, Matava CT, Bahrey LA, Walsh CM: **Real-world impact of the COVID-19 pandemic on the assessment of anaesthesiology residents.** *Br J Anaesth* 2020, **125:**e430-e432.

452. Keihanian T, Sharma P, Goyal J, Sussman DA, Girotra M: **Telehealth Utilization in Gastroenterology Clinics Amid the COVID-19 Pandemic: Impact on Clinical Practice and Gastroenterology Training.** *Gastroenterology* 2020, **159:**1598-1601.

453. Khalaf K, El-Kishawi M, Moufti MA, Al Kawas S: **Introducing a comprehensive high-stake online exam to final-year dental students during the COVID-19 pandemic and evaluation of its effectiveness.** *Med Educ Online* 2020, **25:**1826861.

454. Khalafallah AM, Lam S, Gami A, Dornbos DL, Sivakumar W, Johnson JN, Mukherjee D: **A national survey on the impact of the COVID-19 pandemic upon burnout and career satisfaction among neurosurgery residents.** *J Clin Neurosci* 2020, **80:**137-142.

455. Khalil R, Mansour AE, Fadda WA, Almisnid K, Aldamegh M, Al-Nafeesah A, Alkhalifah A, Al-Wutayd O: **The sudden transition to synchronized online learning during the COVID-19 pandemic in Saudi Arabia: a qualitative study exploring medical students' perspectives.** *BMC Med Educ* 2020, **20:**285.

456. Khan AM, Patra S, Vaney N, Mehndiratta M, Chauhan R: **Rapid transition to online practical classes in preclinical subjects during COVID-19: Experience from a medical college in North India.** *Med J Armed Forces India* 2021, **77:**S161-S167.

457. Khan KS, Keay R, McLellan M, Mahmud S: **Impact of the COVID-19 pandemic on core surgical training.** *Scott Med J* 2020, **65:**133-137.

458. Khan MA, Sivalingam A, Haller JA: **Perceptions of Occupational Risk and Changes in Clinical Practice of United States Vitreoretinal Surgery Fellows during the COVID-19 Pandemic.** *Ophthalmol Retina* 2020, **4:**1181-1187.

459. Khare R, Mahour J, Ohary R, Kumar S: **Impact of online classes, screen time, naps on sleep, and assessment of sleep-related problems in medical college students during lockdown due to coronavirus disease-19 pandemic.** *National Journal of Physiology, Pharmacy and Pharmacology* 2021, **11**.

460. Kharma MY, Koussa B, Aldwaik A, Yaseen J, Alamari S, Alras H, Almech M: **Assessment of Anxiety and Stress among Dental Students to Return to Training in Dental College in COVID-19 Era.** *Eur J Dent* 2020, **14:**S86-S90.

461. Khoodoruth MAS, Al-Nuaimi SK, Al-Salihy Z, Ghaffar A, Khoodoruth WNC, Ouanes S: **Factors associated with mental health outcomes among medical residents exposed to COVID-19.** *BJPsych Open* 2021, **7:**e52.

462. Sameed QUA, Mumtaz T, Qamar A, Ahmad W, Razzak DA, Khurram R: **Mental Distress after 1st Wave of COVID-19 among Medical Students.** *Journal of Pharmaceutical Research International* 2021**:**7-12.

463. Khusid JA, Weinstein CS, Becerra AZ, Kashani M, Robins DJ, Fink LE, Smith MT, Jr., Weiss JP: **Well-being and education of urology residents during the COVID-19 pandemic: Results of an American National Survey.** *Int J Clin Pract* 2020, **74:**e13559.

464. Bianchi C, Kim JW, Myung SJ, Yoon HB, Moon SH, Ryu H, Yim J-J: **How medical education survives and evolves during COVID-19: Our experience and future direction.** *Plos One* 2020, **15**.

465. R. K: **Medical Students Commitment during the SARS-CoV-2 Pandemic: Preparedness, Motivation, and Impact on Students Skills.** *Mendeley Data*.

466. Kochuvilayil T, Fernandez RS, Moxham LJ, Lord H, Alomari A, Hunt L, Middleton R, Halcomb EJ: **COVID-19: Knowledge, anxiety, academic concerns and preventative behaviours among Australian and Indian undergraduate nursing students: A cross-sectional study.** *J Clin Nurs* 2021, **30:**882-891.

467. Goel S, Upadhyay K: **Short-term Impact of a Web-based COVID-19 Certificate Program on Knowledge of Global Public Health Professionals.** *Journal of Postgraduate Medicine, Education and Research* 2020, **54:**150-157.

468. Kronenfeld JP, Ryon EL, Kronenfeld DS, Hui VW, Rodgers SE, Thorson CM, Sands LR: **Medical Student Education During COVID-19: Electronic Education Does Not Decrease Examination Scores.** *Am Surg* 2021, **87:**1946-1952.

469. Kui A, Negucioiu M, Berar A, Fluerasu M, Iacob S, Manziuc M, Buduru S: **Medical Students’ Perception about Online Teaching Methods during COVID-19 Pandemic.** *Journal of Evolution of Medical and Dental Sciences* 2020, **9:**3638-3642.

470. Kumar A ea: **The Psychological Impact of the Covid-19 Lockdown on Medical Students of a College in North India.** *Indian Journal of Public Health Research & Development* 2020.

471. Dharini, Kumar S, More A, Harikar M: **The Impact of COVID-19 and Lockdown on Plastic Surgery Training and Practice in India.** *Indian J Plast Surg* 2020, **53:**273-279.

472. Laloo R, Giorga A, Williams A, Biyani CS, Yiasemidou M: **Virtual surgical education for core surgical trainees in the Yorkshire deanery during the COVID-19 pandemic.** *Scott Med J* 2020, **65:**138-143.

473. Langegard U, Kiani K, Nielsen SJ, Svensson PA: **Nursing students' experiences of a pedagogical transition from campus learning to distance learning using digital tools.** *BMC Nurs* 2021, **20:**23.

474. Lazaro T, Srinivasan VM, Rahman M, Asthagiri A, Barkhoudarian G, Chambless LB, Kan P, Rao G, Nahed BV, Patel AJ: **Virtual education in neurosurgery during the COVID-19 pandemic.** *Neurosurg Focus* 2020, **49:**E17.

475. Lenes A, Klasen M, Adelt A, Goretz U, Proch-Trodler C, Schenkat H, Sopka S: **Crisis as a chance. A digital training of social competencies with simulated persons at the Medical Faculty of RWTH Aachen, due to the lack of attendance teaching in the SARS-Cov-2 pandemic.** *GMS J Med Educ* 2020, **37:**Doc82.

476. Li DF, Shi CX, Shi FZ, Zhao L, Zhao R, Kang WQ: **Effects of simulation training on COVID-19 control ability and psychological states of nurses in a children's hospital.** *Eur Rev Med Pharmacol Sci* 2020, **24:**11381-11385.

477. Li L, Shim T, Zapanta PE: **Optimization of COVID-19 testing accuracy with nasal anatomy education.** *Am J Otolaryngol* 2021, **42:**102777.

478. Li Y, Chu C, de la Calle CM, Baussan C, Ambani SN, Borofsky MS, Breyer BN, Conti SL, Hagedorn JC, Halpern JA, et al: **Multi-Institutional Collaborative Resident Education in the Era of COVID-19.** *Urology Practice* 2020, **7:**425-433.

479. Li Y, Wang Y, Li Y, Zhong M, Liu H, Wu C, Gao X, Xia Z, Ma W: **Comparison of Repeated Video Display vs Combined Video Display and Live Demonstration as Training Methods to Healthcare Providers for Donning and Doffing Personal Protective Equipment: A Randomized Controlled Trial.** *Risk Manag Healthc Policy* 2020, **13:**2325-2335.

480. Li YM, Galimberti F, Abrouk M, Kirsner RS: **US Dermatology Resident Responses about the COVID-19 Pandemic: Results from a Nationwide Survey.** *South Med J* 2020, **113:**462-465.

481. Li Z, Cheng J, Zhou T, Wang S, Huang S, Wang H: **Evaluating a Nurse Training Program in the Emergency Surgery Department Based on the Kirkpatrick's Model and Clinical Demand During the COVID-19 Pandemic.** *Telemed J E Health* 2020, **26:**985-991.

482. Lieberman JA, Nester T, Emrich B, Staley EM, Bourassa LA, Tsang HC: **Coping With COVID-19.** *Am J Clin Pathol* 2021, **155:**79-86.

483. Lima KR, das Neves BS, Ramires CC, Dos Santos Soares M, Martini VA, Lopes LF, Mello-Carpes PB: **Student assessment of online tools to foster engagement during the COVID-19 quarantine.** *Adv Physiol Educ* 2020, **44:**679-683.

484. Lingawi HS, Afifi IK: **COVID-19 Associated Stress Among Dental Students.** *The Open Dentistry Journal* 2020, **14:**554-562.

485. Group C-SCS: **COVID-19 impact on Surgical Training and Recovery Planning (COVID-STAR) - A cross-sectional observational study.** *Int J Surg* 2021, **88:**105903.

486. Acar Sevinc S, Metin S, Balta Basi N, Cinar AS, Turkel Ozkan M, Oba S: **Anxiety and burnout in anesthetists and intensive care unit nurses during the COVID-19 pandemic: a cross-sectional study.** *Braz J Anesthesiol* 2022, **72:**169-175.

487. Acharya S, Ematty TB, Acharya S: **The Role of Online Teaching Among the Undergraduate Dental Students During the Current COVID-19 Pandemic in India: A Pilot Study.** *Pesquisa Brasileira em Odontopediatria e Clínica Integrada* 2021, **21**.

488. Adam M, Urbancic-Rak T, Crnic T: **Dental Students' Discomfort and Anxiety During the First and the Second Lockdown Due to COVID-19 Pandemic at the School of Dental Medicine, University of Zagreb.** *Acta Stomatol Croat* 2021, **55:**186-197.

489. Adhikari A, Sujakhu E, G CS, Zoowa S: **Depression among Medical Students of a Medical College in Nepal during COVID-19 Pandemic: A Descriptive Cross-sectional Study.** *JNMA J Nepal Med Assoc* 2021, **59:**645-648.

490. Adhikari G, Poudel L, Pokhrel B, Bhandari G, Shrestha KK: **Stress among Resident Doctors Working in Different Hospitals of Nepal in the Face of COVID-19 Pandemic: A Descriptive Cross-sectional Study.** *JNMA J Nepal Med Assoc* 2021, **59:**558-563.

491. Adusumilli NC, Kalen J, Hausmann K, Friedman AJ: **Dermatology applicant perspectives of a virtual visiting rotation in the era of COVID-19.** *J Am Acad Dermatol* 2021, **84:**1699-1701.

492. Aftab M, Abadi AM, Nahar S, Ahmed RA, Mahmood SE, Madaan M, Ahmad A: **COVID-19 Pandemic Affects the Medical Students' Learning Process and Assaults Their Psychological Wellbeing.** *Int J Environ Res Public Health* 2021, **18**.

493. Ahmed M ea: **Anxiety and depression in medical students of Sindh province during the covid-19 pandemic.** *Rawal Medical Journal*.

494. Al Zahrani EM, Al Naam YA, AlRabeeah SM, Aldossary DN, Al-Jamea LH, Woodman A, Shawaheen M, Altiti O, Quiambao JV, Arulanantham ZJ, Elsafi SH: **E- Learning experience of the medical profession's college students during COVID-19 pandemic in Saudi Arabia.** *BMC Med Educ* 2021, **21:**443.

495. Al-Husban N, Alkhayat A, Aljweesri M, Alharbi R, Aljazzaf Z, Al-Husban N, Elmuhtaseb MS, Al Oweidat K, Obeidat N: **Effects of COVID-19 pandemic on medical students in Jordanian universities: A multi-center cross-sectional study.** *Ann Med Surg (Lond)* 2021, **67:**102466.

496. Al-Qerem W, Hammad A, Amawi HA, Jarab AS, Ling J: **Anxiety and depression among pharmacy students before and during COVID-19 pandemic.** *Tropical Journal of Pharmaceutical Research* 2022, **20:**1039-1047.

497. Alabdulwahhab KM, Kazmi SY, Sami W, Almujel KN, Alanazi MH, Alanazi KF, Moyana AM, Ahmad MS, Alasbali TA, Al Alwadani F: **Use of online resources by undergraduate medical students at College of Medicine, Majmaah University, Kingdom of Saudi Arabia.** *PLoS One* 2021, **16:**e0255635.

498. Alam L, Alam M, Kazmi SKH, Kazmi SAH: **Impact of COVID-19 pandemic on the residency programs of the country: A multicentre study.** *Pak J Med Sci* 2021, **37:**367-372.

499. Alamer A, Alharbi F: **Synchronous distance teaching of radiology clerkship promotes medical students' learning and engagement.** *Insights Imaging* 2021, **12:**41.

500. Alavudeen SS, Easwaran V, Mir JI, Shahrani SM, Aseeri AA, Khan NA, Almodeer AM, Asiri AA: **The influence of COVID-19 related psychological and demographic variables on the effectiveness of e-learning among health care students in the southern region of Saudi Arabia.** *Saudi Pharm J* 2021, **29:**775-780.

501. Algattas H, Roy S, Agarwal N, Maroon J: **COVID-19 Impact in Neurosurgery Residency: Grit During Pandemic.** *World Neurosurg* 2021, **151:**e395-e398.

502. Ali S ea: **The impact of pandemics on surgical residency program; a survey in tertiary care hospitals of Peshawar, Pakistan.** *Journal of Medical Sciences (Peshawar)*.

503. Alkhamees AA, Assiri H, Alharbi HY, Nasser A, Alkhamees MA: **Burnout and depression among psychiatry residents during COVID-19 pandemic.** *Hum Resour Health* 2021, **19:**46.

504. Almadhi AA, Alhorishi AH, Alnamshan AZ, Alkhamis FA, Alosaimi RS, Alassaf TY, Alateeq FA: **Effect of the COVID-19 pandemic on career perceptions among medical students in (IMSIU).** *World Family Medicine Journal /Middle East Journal of Family Medicine* 2021, **19**.

505. Almhdawi KA, Alazrai A, Obeidat D, Altarifi AA, Oteir AO, Aljammal AH, Arabiat AA, Alrabbaie H, Jaber H, Almousa KM: **Healthcare students' mental and physical well-being during the COVID-19 lockdown and distance learning.** *Work* 2021, **70:**3-10.

506. Almohammed OA, Alotaibi LH, Ibn Malik SA: **Student and educator perspectives on virtual institutional introductory pharmacy practice experience (IPPE).** *BMC Med Educ* 2021, **21:**257.

507. Almomani E, Sullivan J, Hajjieh M, Leighton K: **Simulation-based education programme for upskilling non-critical care nurses for COVID-19 deployment.** *BMJ Simul Technol Enhanc Learn* 2021, **7:**319-322.

508. Almufarriji R, Elarjani T, Abdullah J, Alobaid A, Alturki AY, Aldakkan A, Ajlan A, Lary A, Al Jehani H, Algahtany M, et al: **Impact of COVID-19 on Saudi Neurosurgery Residency: Trainers' and Trainees' Perspectives.** *World Neurosurg* 2021, **154:**e547-e554.

509. AlQhtani A, AlSwedan N, Almulhim A, Aladwan R, Alessa Y, AlQhtani K, Albogami M, Altwairqi K, Alotaibi F, AlHadlaq A, Aldhafian O: **Online versus classroom teaching for medical students during COVID-19: measuring effectiveness and satisfaction.** *BMC Med Educ* 2021, **21:**452.

510. Alsairafi Z, Naser AY, Alsaleh FM, Awad A, Jalal Z: **Mental Health Status of Healthcare Professionals and Students of Health Sciences Faculties in Kuwait during the COVID-19 Pandemic.** *Int J Environ Res Public Health* 2021, **18**.

511. Alshareef R, Al Zahrani A, Alzhrani M, Suwaidi A, Alamry B: **Impact of the COVID-19 pandemic on residents' clinical training and psychosocial well-being in Saudi Arabia's Western region.** *J Family Med Prim Care* 2021, **10:**2580-2586.

512. Alshdaifat E, Sindiani A, Khasawneh W, Abu-Azzam O, Qarqash A, Abushukair H, Obeidat N: **The impact of COVID-19 pandemic on training and mental health of residents: a cross-sectional study.** *BMC Med Educ* 2021, **21:**208.

513. Alsolais A, Alquwez N, Alotaibi KA, Alqarni AS, Almalki M, Alsolami F, Almazan J, Cruz JP: **Risk perceptions, fear, depression, anxiety, stress and coping among Saudi nursing students during the COVID-19 pandemic.** *J Ment Health* 2021, **30:**194-201.

514. Alyami FA, Almuhaideb MA, Alzahrani MA, Sabr YS, Almannie RM: **Impact of COVID-19 pandemic on urology residency training.** *Urol Ann* 2021, **13:**215-219.

515. Amin D, Austin TM, Roser SM, Abramowicz S: **A cross-sectional survey of anxiety levels of oral and maxillofacial surgery residents during the early COVID-19 pandemic.** *Oral Surg Oral Med Oral Pathol Oral Radiol* 2021, **132:**137-144.

516. Rad FA, Otaki F, Baqain Z, Zary N, Al-Halabi M: **Rapid transition to distance learning due to COVID-19: Perceptions of postgraduate dental learners and instructors.** *PLoS One* 2021, **16:**e0246584.

517. Ammann AM, Cortez AR, Vaysburg DM, Winer LK, Sussman JJ, Potts JR, 3rd, Van Haren R, Quillin RC, 3rd: **Examining the impact of COVID-19 restrictions on the operative volumes of US general surgery residents.** *Surgery* 2022, **171:**354-359.

518. Amparore D, Checcucci E, Serni S, Minervini A, Gacci M, Esperto F, Fiori C, Porpiglia F, Campi R, European Society of Residents in U: **Urology Residency Training at the Time of COVID-19 in Italy: 1 Year After the Beginning.** *Eur Urol Open Sci* 2021, **31:**37-40.

519. Anteby R, Sinyard RD, Jogerst KM, McKinley SK, Coe TM, Petrusa E, Phitayakorn R, Scott DJ, Brunt LM, Gee DW: **Challenges of virtual interviewing for surgical fellowships: a qualitative analysis of applicant experiences.** *Surg Endosc* 2022, **36:**3763-3771.

520. Appelbaum NP, Misra SM, Welch J, Humphries MH, Sivam S, Ismail N: **Variations in Medical Students' Educational Preferences, Attitudes and Volunteerism during the COVID-19 Global Pandemic.** *J Community Health* 2021, **46:**1204-1212.

521. Arrogante O, Lopez-Torre EM, Carrion-Garcia L, Polo A, Jimenez-Rodriguez D: **High-Fidelity Virtual Objective Structured Clinical Examinations with Standardized Patients in Nursing Students: An Innovative Proposal during the COVID-19 Pandemic.** *Healthcare (Basel)* 2021, **9**.

522. Attarabeen OF, Gresham-Dolby C, Broedel-Zaugg K: **Pharmacy student stress with transition to online education during the COVID-19 pandemic.** *Curr Pharm Teach Learn* 2021, **13:**928-934.

523. Aulakh G, Wanis C, Wilson G, Moore R: **The impact of COVID-19 on oral surgery training.** *Oral Surg* 2021, **14:**313-320.

524. Awadallah NS, Czaja AS, Fainstad T, McNulty MC, Jaiswal KR, Jones TS, Rumack CM: **The impact of the COVID-19 pandemic on family medicine residency training.** *Fam Pract* 2021, **38:**i9-i15.

525. Awoke M, Mamo G, Abdu S, Terefe B: **Perceived Stress and Coping Strategies Among Undergraduate Health Science Students of Jimma University Amid the COVID-19 Outbreak: Online Cross-Sectional Survey.** *Front Psychol* 2021, **12:**639955.

526. Aziminia N, Khani A, Smith C, Bakhai A, Lisk C: **Hospital trainees' worries, perceived sufficiency of information and reported psychological health during the COVID-19 pandemic.** *Acute Med* 2021, **20:**25-36.

527. Aziz Ansari K, Farooqi FA, Qadir Khan S, Alhareky M, C. Trinidad MA, Abidi T, M M: **Perception on online teaching and learning among health sciences students in higher education institutions during the COVID-19 lockdown – ways to improve teaching and learning in Saudi colleges and universities.** *F1000Research* 2021, **10**.

528. Bachir B, Naji A, Tfayli A: **The educational and psychological impact of the COVID-19 pandemic on medical students: A descriptive survey at the American University of Beirut.** *Medicine (Baltimore)* 2021, **100:**e26646.

529. Badowski D, Rossler KL, Reiland N: **Exploring student perceptions of virtual simulation versus traditional clinical and manikin-based simulation.** *J Prof Nurs* 2021, **37:**683-689.

530. Bai W, Xi HT, Zhu Q, Wang Z, Han L, Chen P, Cai H, Zhao YJ, Chen L, Ge ZM, et al: **Changes in Nursing Students' Career Choices Following the COVID-19 Pandemic in China.** *Front Psychiatry* 2021, **12:**657021.

531. Baker J, Schultz M, Huecker M, Shreffler J, Mallory MN: **Smart glasses and video conferencing provide valuable medical student clinical exposure during COVID-19.** *AEM Educ Train* 2021, **5:**e10571.

532. Balmaks R, Auzina L, Gross IT: **Remote rapid cycle deliberate practice simulation training during the COVID-19 pandemic.** *BMJ Simul Technol Enhanc Learn* 2021, **7:**176-177.

533. Bandyopadhyay S, Georgiou I, Bligh E, Coyle C, Pancharatnam R, Saunders KEA, Collaborative N: **SPICE-19: a 3-Month Prospective Cohort Study of 640 Medical Students and Foundation Doctors.** *Med Sci Educ* 2021, **31:**1621-1637.

534. Banovac I, Katavic V, Blazevic A, Bicanic I, Hladnik A, Kovacic N, Petanjek Z: **The anatomy lesson of the SARS-CoV-2 pandemic: irreplaceable tradition (cadaver work) and new didactics of digital technology.** *Croat Med J* 2021, **62:**173-186.

535. Baro Vila RC, Burgos LM, Sigal A, Costabel JP, Alves de Lima A: **Burnout Syndrome in Cardiology Residents. Impact of the COVID-19 Pandemic on Burnout Syndrome in Cardiology Residents.** *Curr Probl Cardiol* 2022, **47:**100873.

536. Basheti IA, Mhaidat QN, Mhaidat HN: **Prevalence of anxiety and depression during COVID-19 pandemic among healthcare students in Jordan and its effect on their learning process: A national survey.** *PLoS One* 2021, **16:**e0249716.

537. Baticulon RE, Sy JJ, Alberto NRI, Baron MBC, Mabulay REC, Rizada LGT, Tiu CJS, Clarion CA, Reyes JCB: **Barriers to Online Learning in the Time of COVID-19: A National Survey of Medical Students in the Philippines.** *Med Sci Educ* 2021, **31:**615-626.

538. Bdair IA: **Nursing students' and faculty members' perspectives about online learning during COVID-19 pandemic: A qualitative study.** *Teaching and Learning in Nursing* 2021, **16:**220-226.

539. Bell KA, Porter C, Woods AD, Akkurt ZM, Feldman SR: **Impact of the COVID-19 pandemic on dermatology departments' support of medical students: A survey study.** *Dermatol Online J* 2021, **27**.

540. Beninato T, Laird AM, Graves CE, Drake FT, Alhefdhi A, Lee JA, Kuo JH, Grubbs EG, Wang TS, Pasieka JL, Lubitz CC: **Impact of the COVID-19 pandemic on the practice of endocrine surgery.** *Am J Surg* 2022, **223:**670-675.

541. Bernstein JD, Ball LL, Nardone ZB, Watson D: **A virtual sub-internship for otolaryngology-head and neck surgery.** *Laryngoscope Investig Otolaryngol* 2021, **6:**952-957.

542. Bhalla N, Suneja N, Kobryn A, Lew S, Dym H: **The Psychological Well-Being of Medical Versus Dental GME Residents During the COVID 19 Pandemic: A Cross-Sectional Study.** *J Oral Maxillofac Surg* 2021, **79:**1828 e1821-1828 e1828.

543. Joshi H, Bhardwaj R, Hathila S, Vaniya VH: **Pre-COVID conventional offline teaching v/s intra-COVID online teaching: A descriptive map of preference patterns among first year M.B.B.S students.** *Acta Medica International* 2021, **8**.

544. Bhattarai B, Gupta S, Dahal S, Thapa A, Bhandari P: **Perception of Online Lectures among Students of a Medical College in Kathmandu: A Descriptive Cross-sectional Study.** *JNMA J Nepal Med Assoc* 2021, **59:**234-238.

545. Bichave A ea: **Pharmacy students' perceptions on the use of online learning during the Covid-19 pandemic: Advantages, limitation and recommendations.** *Turkish Journal of Physiotherapy and Rehabilitation*.

546. Bilgi K, Aytas G, Karatoprak U, Kazancioglu R, Ozcelik S: **The Effects of Coronavirus Disease 2019 Outbreak on Medical Students.** *Front Psychiatry* 2021, **12:**637946.

547. Binshehab SM ea: **Perception for Online Learning among Undergraduate and Postgraduate Dental Students during COVID-19 Pandemic.** *Annals of Medical and Health Sciences Research*.

548. Boardman D, Wilhite JA, Adams J, Sartori D, Greene R, Hanley K, Zabar S: **Telemedicine Training in the COVID Era: Revamping a Routine OSCE to Prepare Medicine Residents for Virtual Care.** *J Med Educ Curric Dev* 2021, **8:**23821205211024076.

549. Boekhorst F, Khattak H, Topcu EG, Horala A, Goncalves Henriques M: **The influence of the COVID-19 outbreak on European trainees in obstetrics and gynaecology: A survey of the impact on training and trainee.** *Eur J Obstet Gynecol Reprod Biol* 2021, **261:**52-58.

550. Boivin-Proulx LA, Doherty A, Rousseau-Saine N, Doucet S, Ly HQ, Lavoie P, Thibodeau-Jarry N: **Use of Simulation-Based Medical Education for Advanced Resuscitation of In-Hospital Cardiac Arrest Patients With Suspected or Confirmed COVID-19.** *Can J Cardiol* 2021, **37:**1267-1270.

551. Bongomin F, Olum R, Nakiyingi L, Lalitha R, Ssinabulya I, Sekaggya-Wiltshire C, Ocama P, Byakika-Kibwika P: **Internal Medicine Clerkship Amidst COVID-19 Pandemic: A Cross-Sectional Study of the Clinical Learning Experience of Undergraduate Medical Students at Makerere University, Uganda.** *Adv Med Educ Pract* 2021, **12:**253-262.

552. Brett W, King C, Shannon B, Gosling C: **Impact of COVID-19 on paramedicine students: A mixed methods study.** *Int Emerg Nurs* 2021, **56:**100996.

553. Brouwer KR, Walmsley LA, Parrish EM, McCubbin AK, Welsh JD, Braido CEC, Okoli CTC: **Examining the associations between self-care practices and psychological distress among nursing students during the COVID-19 pandemic.** *Nurse Educ Today* 2021, **100:**104864.

554. Brown A, Kassam A, Paget M, Blades K, Mercia M, Kachra R: **Exploring the global impact of the COVID-19 pandemic on medical education: an international cross-sectional study of medical learners.** *Can Med Educ J* 2021, **12:**28-43.

555. Brueggeman DA, Via GG, Froehle AW, Krishnamurthy AB: **Virtual Interviews in the Era of COVID-19: Expectations and Perceptions of Orthopaedic Surgery Residency Candidates and Program Directors.** *JB JS Open Access* 2021, **6**.

556. Bumpstead S, Lim ZJ, Kuhn L, Flynn D, Bakos CL, Potter E, Egerton-Warburton D: **The sourcing and use of high physical resemblance personal protective equipment to train healthcare workers, improve confidence and conserve medical-grade equipment.** *Journal of Hospital Infection* 2021, **112:**104-107.

557. Burgess A, Bansal A, Clarke A, Ayton T, van Diggele C, Clark T, Matar E: **Clinical Teacher Training for health professionals: From blended to online and (maybe) back again?** *Clin Teach* 2021, **18:**630-640.

558. Bury G, Smith S, Kelly M, Bradley C, Howard W, Egan M: **COVID-19 community assessment hubs in Ireland-the experience of clinicians.** *Ir J Med Sci* 2021, **190:**475-480.

559. Byrnes YM, Luu NN, Frost AS, Chao TN, Brody RM, Cannady SB, Rajasekaran K, Shanti RM, Newman JG: **Evaluation of an interactive virtual surgical rotation during the COVID-19 pandemic.** *World J Otorhinolaryngol Head Neck Surg* 2021.

560. Calcagnile T, Sighinolfi MC, Sarchi L, Assumma S, Filippi B, Bonfante G, Cassani A, Spandri V, Turri F, Puliatti S, et al: **COVID-19 and slowdown of residents' activity: Feedback from a novel e-learning event and overview of the literature.** *Urologia* 2021, **88:**332-336.

561. Caldwell KE, Hess A, Wise PE, Awad MM: **Maintaining Effective Senior Resident-Led Intern Education through Virtual Curricular Transition.** *J Surg Educ* 2021, **78:**e112-e120.

562. Campbell S, Corbett S, Burlacu CL: **Reinstating a national simulation programme in anaesthesiology during the coronavirus pandemic.** *BMJ Simul Technol Enhanc Learn* 2021, **7:**575-580.

563. Campos J, Campos LA, Bueno JL, Martins BG: **Emotions and mood swings of pharmacy students in the context of the coronavirus disease of 2019 pandemic.** *Curr Pharm Teach Learn* 2021, **13:**635-642.

564. Canet-Velez O, Botigue T, Lavedan Santamaria A, Masot O, Cemeli T, Roca J: **The perception of training and professional development according to nursing students as health workers during COVID-19: A qualitative study.** *Nurse Educ Pract* 2021, **53:**103072.

565. Carpinito GP, Khouri RK, Jr., Kenigsberg AP, Ganesan V, Kuprasertkul A, Caldwell KM, Hudak SJ, Lemack GE: **The Virtual Urology Residency Match Process: Moving Beyond the Pandemic.** *Urology* 2021, **158:**33-38.

566. Cassidy A ea: **The pyjama sessions: Transition to online education during a pandemic.** *Irish Medical Journal*.

567. Castro MRH, Calthorpe LM, Fogh SE, McAllister S, Johnson CL, Isaacs ED, Ishizaki A, Kozas A, Lo D, Rennke S, et al: **Lessons From Learners: Adapting Medical Student Education During and Post COVID-19.** *Acad Med* 2021, **96:**1671-1679.

568. Cavalieri S, Spinetta M, Zagaria D, Franchi M, Lavazza G, Nardelli F, Serafini A, Leone R, Messina A, Arpaia F, et al: **The impact of COVID-19 pandemic on radiology residents in Northern Italy.** *Eur Radiol* 2021, **31:**7077-7087.

569. Cayo-Rojas CF, Castro-Mena MJ, Agramonte-Rosell RC, Aliaga-Marinas AS, Ladera-Castaneda MI, Cervantes-Ganoza LA, Cervantes-Linan LC: **Impact of COVID-19 Mandatory Social Isolation on the Development of Anxiety in Peruvian Dentistry Students: A Logistic Regression Analysis.** *J Int Soc Prev Community Dent* 2021, **11:**222-229.

570. Cengiz Z, Gurdap Z, Isik K: **Challenges experienced by nursing students during the COVID-19 pandemic.** *Perspect Psychiatr Care* 2022, **58:**47-53.

571. Cerqueira-Silva T, Carreiro R, Nunes V, Passos L, Canedo BF, Andrade S, Ramos PIP, Khouri R, Santos CBS, Nascimento JDS, et al: **Bridging Learning in Medicine and Citizenship During the COVID-19 Pandemic: A Telehealth-Based Case Study.** *JMIR Public Health Surveill* 2021, **7:**e24795.

572. Ch'ang JH, Ford J, Cifrese L, Woodward E, Mears J, Lowrie R, Holland C, Kaplan A, Zhang C, Guterman EL: **Preparing Neurology Residents and Advanced Practice Providers for the COVID-19 ICU-A Neurocritical Care Led Intervention.** *Neurohospitalist* 2021, **11:**342-347.

573. Chadha N, Fredrick D, Malbari A, Hojsak J: **A Virtual Clinical Reasoning Case for Medical Students Using an Ophthalmology Model: A Case of Red Eye.** *MedEdPORTAL* 2021, **17:**11117.

574. Chalfant JS, Pittman SM, Kothari PD, Chong A, Grimm LJ, Sohlich RE, Leung JWT, Downey JR, Cohen EO, Ojeda-Fournier H, et al: **Impact of the COVID-19 Pandemic on Breast Imaging Education.** *J Breast Imaging* 2021, **3:**354-362.

575. Chalise GD, Bharati M, Bajracharya J, Kc A, Pradhan S, Adhikari B, Shrestha M: **Undergraduate Medical Science Students' Positive Attitude towards Online Classes during COVID-19 Pandemic in a Medical College: A Descriptive Cross-sectional Study.** *JNMA J Nepal Med Assoc* 2021, **59:**134-140.

576. Chan JCY, Waddell TK, Yasufuku K, Keshavjee S, Donahoe LL: **Maintaining technical proficiency in senior surgical fellows during the COVID-19 pandemic through virtual teaching.** *JTCVS Open* 2021, **8:**679-687.

577. Chan SL, Lin CC, Chau PH, Takemura N, Fung JTC: **Evaluating online learning engagement of nursing students.** *Nurse Educ Today* 2021, **104:**104985.

578. Chan V, Larson ND, Moody DA, Moyer DG, Shah NL: **Impact of 360 degrees vs 2D Videos on Engagement in Anatomy Education.** *Cureus* 2021, **13:**e14260.

579. Chang CY, Hwang GJ, Gau ML: **Promoting students' learning achievement and self‐efficacy: A mobile chatbot approach for nursing training.** *British Journal of Educational Technology* 2021, **53:**171-188.

580. Chang TY, Hsu ML, Kwon JS, Kusdhany MLS, Hong G: **Effect of online learning for dental education in asia during the pandemic of COVID-19.** *J Dent Sci* 2021, **16:**1095-1101.

581. Chauhan S, Dash S, Ranjan P, Singhal M: **Examination in the Time of COVID-19-MCh Plastic Surgery Examination: How Did We Do It?** *Indian J Plast Surg* 2021, **54:**168-171.

582. Cheah WL, Francis Wing CB, Zahari AN, Idris AS, Maksul NAA, Yusman NAL, John W: **Willingness to treat COVID-19 disease: What do medical & nursing students perceive?** *Ethics Med Public Health* 2021, **17:**100651.

583. Cheng CF, Knewitz AP, Pasic TR, Glazer TA: **How We Do It: Otolaryngology Applicant Impressions of Virtual Meet and Greets at a Single Institution.** *J Surg Educ* 2022, **79:**40-45.

584. Cheng HC, Lu SL, Yen YC, Siewchaisakul P, Yen AM, Chen SL: **Dental education changed by COVID-19: Student's perceptions and attitudes.** *BMC Med Educ* 2021, **21:**364.

585. Cheng K, Grabowski C, Chong A, Yen A, Chung CB: **Initial Experience With Formal Near-Peer Mentoring in Radiology Residency.** *Curr Probl Diagn Radiol* 2022, **51:**304-307.

586. Cheng L, Guo X, Liu H, Chen Q, Cui R: **Hope, death anxiety and simplified coping style scores of nursing students during the outbreak of COVID-19: A cross-sectional study.** *Medicine (Baltimore)* 2021, **100:**e27016.

587. Cherak SJ, Brown A, Kachra R, Makuk K, Sudershan S, Paget M, Kassam A: **Exploring the impact of the COVID-19 pandemic on medical learner wellness: a needs assessment for the development of learner wellness interventions.** *Can Med Educ J* 2021, **12:**54-69.

588. Chi DL, Randall CL, Hill CM: **Dental trainees' mental health and intention to leave their programs during the COVID-19 pandemic.** *J Am Dent Assoc* 2021, **152:**526-534.

589. Cho MK, Kim MY: **Factors Affecting Learning Satisfaction in Face-to-Face and Non-Face-to-Face Flipped Learning among Nursing Students.** *Int J Environ Res Public Health* 2021, **18**.

590. Cho WC, Gill P, Aung PP, Gu J, Nagarajan P, Ivan D, Curry JL, Prieto VG, Torres-Cabala CA: **The utility of digital pathology in improving the diagnostic skills of pathology trainees in commonly encountered pigmented cutaneous lesions during the COVID-19 pandemic: A single academic institution experience.** *Ann Diagn Pathol* 2021, **54:**151807.

591. Chopra J, Rani A, Chopra S, Manik P, Singh RR: **Transition from physical to virtual classroom amidst COVID-19 crisis: Analyzing students' perspective to drive improvement in the current online teaching methodology.** *J Educ Health Promot* 2021, **10:**241.

592. Chowdhury L ea: **Effect of counseling on the mental status of undergraduate medical students during the pandemic — a prospective quasi-experimental study at a district medical college of west bengal.** *Journal of the Indian Medical Association*.

593. Christian RJ, VanSandt M: **Using Dynamic Virtual Microscopy to Train Pathology Residents During the Pandemic: Perspectives on Pathology Education in the Age of COVID-19.** *Acad Pathol* 2021, **8:**23742895211006819.

594. Chugh A, Jain G, Gaur S, Gigi PG, Kumar P, Singh S: **Transformational Effect of COVID Pandemic on Postgraduate Programme in Oral and Maxillofacial Surgery in India-A Trainee Perspective.** *J Maxillofac Oral Surg* 2022, **21:**265-270.

595. Ciavoi G ea: **TEST ANXIETY IN DENTAL MEDICAL STUDENTS DURING COVID-19 PANDEMIC.** *Romanian Journal of Oral Rehabilitation*.

596. Clements JM, Burke JR, Hope C, Nally DM, Doleman B, Giwa L, Griffiths G, Lund JN: **The quantitative impact of COVID-19 on surgical training in the United Kingdom.** *BJS Open* 2021, **5**.

597. Coe TM, McBroom TJ, Brownlee SA, Regan K, Bartels S, Saillant N, Yeh H, Petrusa E, Dageforde LA: **Medical Students and Patients Benefit from Virtual Non-Medical Interactions Due to COVID-19.** *J Med Educ Curric Dev* 2021, **8:**23821205211028343.

598. Colthorpe K, Ainscough L: **Do-it-yourself physiology labs: Can hands-on laboratory classes be effectively replicated online?** *Adv Physiol Educ* 2021, **45:**95-102.

599. Conway NB, Tempest HG, Fortun J: **Remote Learning and Its Impact on Newly Matriculated Medical Students.** *Cureus* 2021, **13:**e17223.

600. Cooke A, Hancock A, White H, Clark N, Gibb F, McNeill J, Thomas G, Lloyd C, Furber C: **Exploring the STEP-uP to practice: A survey of UK Lead Midwives for Education views of the STudent midwife Extended Practice Placement during the first wave of the COVID-19 pandemic.** *Midwifery* 2021, **101:**103048.

601. Cornes S, Gelfand JM, Calton B: **Foundational Telemedicine Workshop for First-Year Medical Students Developed During a Pandemic.** *MedEdPORTAL* 2021, **17:**11171.

602. Corte-Real A, Nunes T, Caetano C, Almiro PA: **Cone Beam Computed Tomography (CBCT) Technology and Learning Outcomes in Dental Anatomy Education: E-Learning Approach.** *Anat Sci Educ* 2021, **14:**711-720.

603. Crome M, Adam K, Flohr M, Rahman A, Staufenbiel I: **Application of the inverted classroom model in the teaching module "new classification of periodontal and peri-implant diseases and conditions" during the COVID-19 pandemic.** *GMS J Med Educ* 2021, **38:**Doc89.

604. Crossman ME, Stobart-Gallagher M, Siegel M: **Determining Goals of Care During the COVID-19 Pandemic: A Virtual Course for Emergency Medicine Residents.** *Cureus* 2021, **13:**e14558.

605. Curtin M, Downs J, Hunt A, Coleman ER, Enneking BA, McNally Keehn R: **INteractive Virtual Expert-Led Skills Training: A Multi-Modal Curriculum for Medical Trainees.** *Front Psychiatry* 2021, **12:**671442.

606. Cypro A, McGuire WC, Rolfsen M, Jones N, Shah NG, Cribbs SK, Kaul V, Bojanowski CM, Pedraza I, Lynch L, et al: **An International Virtual COVID-19 Critical Care Training Forum for Healthcare Workers.** *ATS Sch* 2021, **2:**278-286.

607. Czawlytko C, Smith E, Awan O, Resnik C, Hossain R: **The Effect of Virtual Interviews and Social Media on Applicant Decision-Making During The 2020-2021 Resident Match Cycle.** *Acad Radiol* 2022, **29:**928-934.

608. D'Angelo JD, D'Angelo AD, Mathis KL, Dozois EJ, Kelley SR: **Program Director Opinions of Virtual Interviews: Whatever Makes my Partners Happy.** *J Surg Educ* 2021, **78:**e12-e18.

609. Dale-Tam J, Thompson K, Dale L: **Creating Psychological Safety During a Virtual Simulation Session.** *Clinical Simulation in Nursing* 2021, **57:**14-17.

610. Danilovic A, Torricelli FCM, Dos Anjos G, Cordeiro MD, Machado MG, Srougi M, Nahas WC: **Impact of COVID-19 on a urology residency program.** *Int Braz J Urol* 2021, **47:**448-453.

611. Darici D, Reissner C, Brockhaus J, Missler M: **Implementation of a fully digital histology course in the anatomical teaching curriculum during COVID-19 pandemic.** *Ann Anat* 2021, **236:**151718.

612. Das A, Sil A, Chakrabarti A: **An Observational Survey to Appraise the Influence of COVID-19 Pandemic on Dermatology Training Programs in India: Residents' Standpoint.** *Indian Dermatol Online J* 2021, **12:**423-428.

613. Dave M, Dixon C, Patel N: **An educational evaluation of learner experiences in dentistry open-book examinations.** *Br Dent J* 2021, **231:**243-248.

614. Davis CE, Hayes L, Dent N, Jennings I, Arumugasamy M, Walsh TN: **Impact of COVID-19 on surgical training.** *Br J Surg* 2021, **108:**e199-e200.

615. DeAtkine AB, Chisolm PF, Singh NP, Koch CG, King TW, Greene BJ, Buczek EP: **Interviewing Otolaryngology Applicants in a Virtual Setting: A Perspective After 2020 to 2021 Match.** *Ear Nose Throat J* 2021**:**1455613211040377.

616. Dhingra S, Pasricha N, Sthapak E, Bhatnagar R: **Assessing the Role of Internal Motivation and Extrinsic Factors on Online Undergraduate Medical Teaching in a Resource-Poor Setting During Covid-19 Pandemic in North India: An Observational Study.** *Adv Med Educ Pract* 2021, **12:**817-823.

617. Doddaiah SK, Kulkarni P, Mohandas A, Murthy MRN: **Learning with lockdown: Utility of whatsApp status-based multiple-choice question discussion on COVID-19 among medical students and teaching faculty.** *J Educ Health Promot* 2021, **10:**168.

618. Domaradzki J, Walkowiak D: **Medical Students' Voluntary Service During the COVID-19 Pandemic in Poland.** *Front Public Health* 2021, **9:**618608.

619. Dorji T, Tamang ST, Tilak T: **Self-learning on COVID-19 among medical students in Bhutan: A cross-sectional study.** *Heliyon* 2021, **7:**e07533.

620. Dulohery K, Scully D, Longhurst GJ, Stone DM, Campbell T: **Emerging from emergency pandemic pedagogy: A survey of anatomical educators in the United Kingdom and Ireland.** *Clin Anat* 2021, **34:**948-960.

621. Duprez V, Vermote B, Van Hecke A, Verhaeghe R, Vansteenkiste M, Malfait S: **Are internship experiences during a pandemic related to students' commitment to nursing education? A cross-sectional study.** *Nurse Educ Today* 2021, **107:**105124.

622. Dutta AK, Goswami K, Murugayan SB, Sahoo S, Pal A, Paul C, Thallapaneni S, Biswas S: **Evaluation of e-OSPE as compared to traditional OSPE: A pilot study.** *Biochem Mol Biol Educ* 2021, **49:**457-463.

623. Edakhlon S, Mohan P, Pillai GS, Verma L, Honavar SG, Sharma N, Nadaraj A: **Impact of COVID-19 pandemic on income and opportunities of ophthalmologists in India: Ophthalmologists' Workplace Expectations and Satisfaction Survey (OWESS) Report 1.** *Indian J Ophthalmol* 2021, **69:**2189-2194.

624. Effendy E, Selian A, Siagian JM: **Factors related to anxiety among resident doctors assigned to emergency room during the COVID-19 pandemic: a multivariate study at Sumatera Utara Affiliated Teaching Hospital.** *Med Glas (Zenica)* 2021, **18:**493-498.

625. Ehilawa P, Thompson F, Ahmed R, Ariyo M, Muldoon S, Sadler P, Corne J: **Impact of COVID-19 pandemic on postgraduate medical education – a survey of UK trainees.** *Future Healthcare Journal* 2021, **8:**24-25.

626. El Homossany M, Alrowaily GS, Allugmani WA, Abo-Atwan WK, Al-Sulaiman RA, Al-Mofareh OM, Alharbi AS, Almuqrin AD: **COVID-19-Related Anxiety among Dental Students in Two Dental Schools in Saudi Arabia.** *J Pharm Bioallied Sci* 2021, **13:**S826-S830.

627. ElHawary H, Salimi A, Barone N, Alam P, Thibaudeau S: **The effect of COVID-19 on medical students' education and wellbeing: a cross-sectional survey.** *Can Med Educ J* 2021, **12:**92-99.

628. Eltaybani S, Abdelhalim GE, Abdelgawad ME: **Nursing students' and educators' experience with e-learning during a pandemic: An online survey.** *Nurs Forum* 2021, **56:**878-888.

629. Erturk Avunduk AT, Delikan E: **Satisfaction and stress levels of dentistry students relating to distance education.** *Dent Med Probl* 2021, **58:**291-298.

630. Everard KM, Schiel KZ: **Changes in Family Medicine Clerkship Teaching Due to the COVID-19 Pandemic.** *Fam Med* 2021, **53:**282-284.

631. Eycan O, Ulupinar S: **Nurse instructors' perception towards distance education during the pandemic.** *Nurse Educ Today* 2021, **107:**105102.

632. Faderani R, Monks M, Peprah D, Colori A, Allen L, Amphlett A, Edwards M: **Improving wellbeing among UK doctors redeployed during the COVID-19 pandemic.** *Future Healthc J* 2020, **7:**e71-e76.

633. Farias Bezerra HK, Passos KKM, Leonel A, Ferreti Bonan PR, Martelli-Junior H, Machado RA, Ramos-Perez FMM, Perez D: **The impact of the COVID-19 pandemic on undergraduate and graduate dental courses in Brazil.** *Work* 2021, **70:**31-39.

634. Farlow JL, Devare J, Ellsperman SE, Haring CT, Heft Neal ME, Pleasant T, Spielbauer KK, Sylvester MJ, Xie Y, Marchiano EJ: **Virtual Resident Mentorship Groups for Fourth Year Medical Students Applying into Otolaryngology-Head and Neck Surgery.** *Ann Otol Rhinol Laryngol* 2021**:**34894211015740.

635. Farsi Z, Sajadi SA, Afaghi E, Fournier A, Aliyari S, Ahmadi Y, Hazrati E: **Explaining the experiences of nursing administrators, educators, and students about education process in the COVID-19 pandemic: a qualitative study.** *BMC Nurs* 2021, **20:**151.

636. Fatima SS, Idrees R, Jabeen K, Sabzwari S, Khan S: **Online assessment in undergraduate medical education: Challenges and solutions from a LMIC university.** *Pak J Med Sci* 2021, **37:**945-951.

637. Ferguson CC, Figy SC, Manley NA: **Nursing Home Education During the COVID-19 Pandemic.** *J Med Educ Curric Dev* 2021, **8:**2382120521997096.

638. Fernandez MDS, Vieira IS, Silva N, Cardoso TA, Bielavski CH, Rakovski C, Silva AER: **Anxiety symptoms and alcohol abuse during the COVID-19 pandemic: A cross-sectional study with Brazilian dental undergraduate students.** *J Dent Educ* 2021, **85:**1739-1748.

639. Ferreira LC, Amorim RS, Melo Campos FM, Cipolotti R: **Mental health and illness of medical students and newly graduated doctors during the pandemic of SARS-Cov-2/COVID-19.** *PLoS One* 2021, **16:**e0251525.

640. Fieux M, Gavoille A, Subtil F, Bartier S, Tringali S: **Otoskills training during covid-19 pandemic: a before-after study.** *BMC Med Educ* 2021, **21:**284.

641. Foo CC, Cheung B, Chu KM: **A comparative study regarding distance learning and the conventional face-to-face approach conducted problem-based learning tutorial during the COVID-19 pandemic.** *BMC Med Educ* 2021, **21:**141.

642. Foohey S, Nagji A, Yilmaz Y, Sibbald M, Monteiro S, Chan TM: **Developing the Virtual Resus Room: Fidelity, Usability, Acceptability, and Applicability of a Virtual Simulation for Teaching and Learning.** *Acad Med* 2022, **97:**679-683.

643. Ford TR, Fix ML, Shappell E, Egan DJ, Mannix A, Bailitz J, Li X, Gottlieb M: **Beyond the emergency department: Effects of COVID-19 on emergency medicine resident education.** *AEM Educ Train* 2021, **5:**e10568.

644. Fossey S, Ather S, Davies S, Dhillon PS, Malik N, Phillips M, Harden S: **Impact of COVID-19 on radiology training: Royal College of Radiologists Junior Radiologists Forum national survey.** *Clin Radiol* 2021, **76:**549 e549-549 e515.

645. Frankl SE, Joshi A, Onorato S, Jawahir GL, Pelletier SR, Dalrymple JL, Schwartz AW: **Preparing Future Doctors for Telemedicine: An Asynchronous Curriculum for Medical Students Implemented During the COVID-19 Pandemic.** *Acad Med* 2021, **96:**1696-1701.

646. Fu L, Swete M, Selgrade D, Chan CW, Rodriguez R, Wolniak K, Blanco LZ, Jr.: **Virtual Pathology Elective Provides Uninterrupted Medical Education and Impactful Pathology Education During the COVID-19 Pandemic.** *Acad Pathol* 2021, **8:**23742895211010275.

647. Fung JTC, Zhang W, Yeung MN, Pang MTH, Lam VSF, Chan BKY, Wong JY: **Evaluation of students' perceived clinical competence and learning needs following an online virtual simulation education programme with debriefing during the COVID-19 pandemic.** *Nurs Open* 2021, **8:**3045-3054.

648. Furlan R, Gatti M, Mene R, Shiffer D, Marchiori C, Giaj Levra A, Saturnino V, Brunetta E, Dipaola F: **A Natural Language Processing-Based Virtual Patient Simulator and Intelligent Tutoring System for the Clinical Diagnostic Process: Simulator Development and Case Study.** *JMIR Med Inform* 2021, **9:**e24073.

649. Fyllos A, Kanellopoulos A, Kitixis P, Cojocari DV, Markou A, Raoulis V, Strimpakos N, Zibis A: **University Students Perception of Online Education: Is Engagement Enough?** *Acta Inform Med* 2021, **29:**4-9.

650. Gaba F, Blyuss O, Rodriguez I, Dilley J, Wan YL, Saiz A, Razumova Z, Zalewski K, Nikolova T, Selcuk I, et al: **Impact of SARS-CoV-2 on training and mental well-being of surgical gynecological oncology trainees.** *Int J Gynecol Cancer* 2021, **31:**1268-1277.

651. Ganesananthan S, Li C, Donnir A, Anthony A, Woo T, Zielinska AP, Khajuria A: **Changing Student Perception of an Online Integrated Structured Clinical Examination During the COVID-19 Pandemic.** *Adv Med Educ Pract* 2021, **12:**887-894.

652. Gao F, Jiao SX, Bi YQ, Huang ZY, Wang P, Zhang BY, Fang J, Han RL, Fan L, Wang MJ, et al: **The Impact of the SARS-COV-2 Pandemic on the Mental Health and Employment Decisions of Medical Students in North China.** *Front Psychiatry* 2021, **12:**641138.

653. Garcia-Gonzalez J, Ruqiong W, Alarcon-Rodriguez R, Requena-Mullor M, Ding C, Ventura-Miranda MI: **Analysis of Anxiety Levels of Nursing Students Because of e-Learning during the COVID-19 Pandemic.** *Healthcare (Basel)* 2021, **9**.

654. Gardas S, Nair S, Pitchai P, Panhale V: **Postgraduate Physiotherapy Training in a Quandary - Ramifications of Corona virus pandemic Lockdown: A Survey-based Study.** *J Adv Med Educ Prof* 2021, **9:**144-153.

655. Gautam S, Imteyaz SP, Alam MI: **COVID-19 Pandemic: Assessment of Stress and Perception of E-Learning amongst First Year Undergraduate Medical Students.** *Journal of Clinical and Diagnostic Research* 2021.

656. Geary AD, Wang TS, Lindeman B, Kuo JH, Lyden ML, Shen WT, Morris-Wiseman LF, Carty SE, Drake FT: **Perspectives on virtual interviews-A follow-up study of the Comprehensive Endocrine Surgery Fellowship interview process.** *Surgery* 2022, **171:**259-264.

657. Geer LA, Radigan R, Bruneli GL, Leite LS, Belian RB: **COVID-19: A Cross-Sectional Study of Healthcare Students' Perceptions of Life during the Pandemic in the United States and Brazil.** *Int J Environ Res Public Health* 2021, **18**.

658. Gelineau-Morel R, Dilts J: **Virtual Education During COVID-19 and Beyond.** *Pediatr Neurol* 2021, **119:**1-2.

659. Geng Y, Huang P-S, Huang Y-M: **Crowdsourcing in Nursing Education: A Possibility of Creating a Personalized Online Learning Environment for Student Nurses in the Post-COVID Era.** *Sustainability* 2021, **13**.

660. Ghafari R, Mirghafourvand M, Rouhi M, Osouli Tabrizi S: **Mental health and its relationship with social support in Iranian students during the COVID-19 pandemic.** *BMC Psychol* 2021, **9:**81.

661. Ghidei L, Gannon A, Schutt A: **The impact of COVID-19 on REI fellowship educational experience.** *J Assist Reprod Genet* 2021, **38:**1163-1169.

662. Ghogare A, Aloney S, Spoorthy M, Patil P, Ambad R, Bele A: **A cross-sectional online survey of relationship between the psychological impact of coronavirus disease 2019 and the resilience among postgraduate health sciences students from Maharashtra, India.** *International Journal of Academic Medicine* 2021, **7**.

663. Ghosal T, Sadhu A, Mukherjee P, Mukhopadhyay P: **Assessment of Online Learning Procedure through the Eyes of Medical Students inCOVID-19 Scenario.** *Journal of Clinical and Diagnostic Research* 2021.

664. Gieswein J, Tennill R, Austin R, Delfino K, Dynda D, Kim S: **Developing a Novel, At-Home Procedure Curriculum for Fourth-Year Medical Students in Response to the COVID-19 Pandemic.** *Cureus* 2021, **13:**e15215.

665. Gismalla MD, Mohamed MS, Ibrahim OSO, Elhassan MMA, Mohamed MN: **Medical students' perception towards E-learning during COVID 19 pandemic in a high burden developing country.** *BMC Med Educ* 2021, **21:**377.

666. Goldenson RP, Avery LL, Gill RR, Durfee SM: **The Virtual Homeroom: Utility and Benefits of Small Group Online Learning in the COVID-19 Era.** *Curr Probl Diagn Radiol* 2022, **51:**152-154.

667. Golub SA, Pham DQ, Bargeron EL, Breuner CC, Evans YN: **Evaluating the Educational Impact of Telehealth on Adolescent Medicine Trainees: a Qualitative Approach.** *Curr Pediatr Rep* 2021, **9:**72-76.

668. Gondim M, Carneiro PH, Moreno R, Lynch MI: **Impact of Coronavirus Disease on the Ophthalmology Residency Training in Brazil.** *Arq Bras Oftalmol* 2021, **84:**297-298.

669. Goodman JF, Saini P, Straughan AJ, Badger CD, Thakkar P, Zapanta PE: **The Virtual Mock Oral Examination: A Multi-institutional Study of Resident and Faculty Receptiveness.** *OTO Open* 2021, **5:**2473974X21997392.

670. Gore JL, Porten SP, Montgomery JS, Hamilton RJ, Meng MV, Sexton WJ, Psutka SP: **Applicant perceptions of virtual interviews for society of urologic oncology fellowships during the COVID-19 pandemic.** *Urol Oncol* 2021.

671. Gosak L, Fijacko N, Chabrera C, Cabrera E, Stiglic G: **Perception of the Online Learning Environment of Nursing Students in Slovenia: Validation of the DREEM Questionnaire.** *Healthcare (Basel)* 2021, **9**.

672. Gowda S, Simmons J, Gokani VJ: **International cross-sectional survey on the use of webinars in plastic surgery: a move towards a hybrid educational model.** *Eur J Plast Surg* 2022, **45:**169-176.

673. Grover P ea: **Efficacy of online pre-recorded practical demonstrations and live streamed practical demonstrations in third year physiotherapy students during the Covid-19 pandemic- A survey.** *Turkish Journal of Physiotherapy and Rehabilitation*.

674. Gupta P, B KA, Ramakrishna K: **Prevalence of Depression and Anxiety Among Medical Students and House Staff During the COVID-19 Health-Care Crisis.** *Acad Psychiatry* 2021, **45:**575-580.

675. Gurkha D, Cashen K, Patek P, Lelak K, Levasseur K: **Coronavirus-19 Multisystem Inflammatory Syndrome in Children (MIS-C): A Pediatric Simulation Case for Residents, Fellows, and Advanced Practice Providers.** *MedEdPORTAL* 2021, **17:**11180.

676. Hanafy SM, Jumaa MI, Arafa MA: **A comparative study of online learning in response to the coronavirus disease 2019 pandemic versus conventional learning.** *Saudi Med J* 2021, **42:**324-331.

677. Harmer MJ, Southgate G, Raja M, Alam S: **Paediatric trainees' training experiences during the COVID-19 pandemic: a national survey.** *Arch Dis Child Educ Pract Ed* 2022, **107:**64-70.

678. Hashmi MSS ea: **Medical student's performance and satisfaction with online theory exams during the covid-19 pandemic.** *Medical Forum Monthly*.

679. Hassell LA, Peterson J, Pantanowitz L: **Pushed Across the Digital Divide: COVID-19 Accelerated Pathology Training onto a New Digital Learning Curve.** *Acad Pathol* 2021, **8:**2374289521994240.

680. Hattar S, AlHadidi A, Sawair FA, Alraheam IA, El-Ma'aita A, Wahab FK: **Impact of COVID-19 pandemic on dental education: online experience and practice expectations among dental students at the University of Jordan.** *BMC Med Educ* 2021, **21:**151.

681. Haws BE, Mannava S, Schuster BK, DiGiovanni BF: **Implementation and Evaluation of a Formal Virtual Medical Student Away Rotation in Orthopaedic Surgery During the COVID-19 Pandemic: A Single Institution Pilot Experience.** *JB JS Open Access* 2021, **6**.

682. Hayat AA, Keshavarzi MH, Zare S, Bazrafcan L, Rezaee R, Faghihi SA, Amini M, Kojuri J: **Challenges and opportunities from the COVID-19 pandemic in medical education: a qualitative study.** *BMC Med Educ* 2021, **21:**247.

683. Hennessy O, Fowler AL, Hennessy C, Brinkman D, Hogan A, Nugent E, Joyce M: **Riding the waves: the ongoing impact of COVID-19 on a national surgical training cohort.** *Ir J Med Sci* 2022, **191:**1823-1829.

684. Herrmann-Werner A, Erschens R, Zipfel S, Loda T: **Medical education in times of COVID-19: survey on teachers' perspectives from a German medical faculty.** *GMS J Med Educ* 2021, **38:**Doc93.

685. Im JH, Kim JW, Park WB, Han I, Lee SH, Shin JS, Yoon HB: **Is it feasible and effective to provide faculty development programs online for clinical teachers?** *Korean J Med Educ* 2021, **33:**139-145.

686. Imeri H, Jadhav S, Barnard M, Rosenthal M: **Mapping the impact of the COVID-19 pandemic on pharmacy graduate students' wellness.** *Res Social Adm Pharm* 2021, **17:**1962-1967.

687. Inzunza M, Besser N, Bellolio F: **Decrease in operative volume in general surgery residents in Chile: effects of the COVID-19 pandemic.** *Br J Surg* 2021, **108:**e226-e227.

688. Jeon E, Peltonen LM, Block L, Ronquillo C, Tayaben JL, Nibber R, Pruinelli L, Perezmitre EL, Sommer J, Topaz M, et al: **Emergency Remote Learning in Nursing Education During the COVID-19 Pandemic.** *Stud Health Technol Inform* 2021, **281:**942-946.

689. Jiang Z, Zhu D, Li J, Ren L, Pu R, Yang G: **Online dental teaching practices during the COVID-19 pandemic: a cross-sectional online survey from China.** *BMC Oral Health* 2021, **21:**189.

690. Jimenez-Labaig P, Pacheco-Barcia V, Cebria A, Galvez F, Obispo B, Paez D, Quilez A, Quintanar T, Ramchandani A, Remon J, et al: **Identifying and preventing burnout in young oncologists, an overwhelming challenge in the COVID-19 era: a study of the Spanish Society of Medical Oncology (SEOM).** *ESMO Open* 2021, **6:**100215.

691. Karabulut N, Yaman Aktaş Y, Gürçayır D, Bulut G, Kara A, Yildiz B: **The effect of perceived stress and personality types of nursing college students on attitudes towards nursing profession during Covid-19 pandemic.** *Vulnerable Children and Youth Studies* 2021, **16:**345-357.

692. Keyserling K, Janetos E, Sprague C: **Teaching Telehealth During a Pandemic and Beyond: an Intern's Survival Guide for Virtual Medicine.** *J Gen Intern Med* 2021, **36:**3219-3223.

693. Kuman Tuncel O, Tasbakan SE, Gokengin D, Erdem HA, Yamazhan T, Sipahi OR, Pullukcu H, Onen Sertoz O, Isikgoz Tasbakan M: **The deep impact of the COVID-19 pandemic on medical students: An online cross-sectional study evaluating Turkish students' anxiety.** *Int J Clin Pract* 2021, **75:**e14139.

694. Kumar S, Banerjee A, Ahmad A: **A Cross-Sectional Study on Mental Health and Cardiovascular Reactivity among Fresh Resident Doctors during Covid19 Pandemic in India.** *International Journal of Current Pharmaceutical Research* 2021**:**52-54.

695. Kuravi BG, Gogineni S, Bhargav PRK, Mayilvaganan S, Nilofaur, Shanthi V, Ch S: **Utility of Virtual Platform for Conducting Practical Examination for Medical Students During Covid Times: A Prospective Study from Gynaecology Department.** *J Obstet Gynaecol India* 2021, **71:**47-51.

696. Lerendegui L, Boudou R, Percul C, Curiel A, Durante E, Moldes JM, de Badiola F, Liberto DH, Delorenzi E, Lobos PA: **Impact of the COVID-19 pandemic on surgical skills training in pediatric surgery residents.** *Pediatr Surg Int* 2021, **37:**1415-1420.

697. Lin L, Ni S, Cheng J, Zhang Z, Zeng R, Jin X, Zhao Y: **Effect of synchronous online vs. face-to-face cardiopulmonary resuscitation training on chest compression quality: A pilot randomized manikin study.** *Am J Emerg Med* 2021, **50:**80-84.

698. Luke S, Petitt E, Tombrella J, McGoff E: **Virtual Evaluation of Clinical Competence in Nurse Practitioner Students.** *Med Sci Educ* 2021, **31:**1267-1271.

699. Luton OW, James OP, Mellor K, Eley C, Hopkins L, Robinson DBT, Lebares CC, Powell A, Lewis WG, Egan RJ: **Enhanced stress-resilience training for surgical trainees.** *BJS Open* 2021, **5**.

700. Miao Q, Xie L, Xing B, Wang X, Tang S, Luo H: **Emotional states and coping methods in nursing and non-nursing students responding to COVID-19: a cross-sectional study in China.** *BMJ Open* 2021, **11:**e054007.

701. Mukherjee SS, Sarkar KD, Sengupta D, Sinhababu S: **Response of 1st Year Medical Students of West Bengal about Compulsive Online Teaching during COVID-19 Pandemic: An Observational Study.** *Journal of Clinical and Diagnostic Research* 2021.

702. Muley PP ea: **Effect of Covid-19 Lockdown on Perceived Stress Scale in Medical Students.** *Indian Journal of Forensic Medicine & Toxicology* 2020.

703. Naifeh MM, Stevenson MD, Abramson EL, Aston CE, Li ST: **Early Impact of the COVID-19 Pandemic on Pediatric Resident Workforce.** *Pediatrics* 2021, **148**.

704. Patel RR, Nel D, Coccia A, Rayamajhi S: **General Surgery Training - During the COVID 19 Pandemic and Beyond Authors.** *Surg Innov* 2021, **28:**245-246.

705. Patel ST, Shah S, Sood RP, Siddiqui Z, McKay-Davies I: **The Implementation of Virtual Clinical Skills Teaching in Improving Procedural Confidence in ENT Trainees.** *Adv Med Educ Pract* 2021, **12:**965-969.

706. Rahic O, Sirbubalo M, Tucak A, Hadziabdic J, Elezovic A, Vranic E: **COVID-19 challenges in organising teaching at a faculty of pharmacy.** *BMJ Simul Technol Enhanc Learn* 2021, **7:**646-647.

707. Rehman R, Fatima SS: **An innovation in Flipped Class Room: A teaching model to facilitate synchronous and asynchronous learning during a pandemic.** *Pak J Med Sci* 2021, **37:**131-136.

708. Rios IC, Imamura M, Garcia MLB, Battistella LR: **Virtual interviews between medical students and in-patients during COVID-19 pandemic.** *Med Educ* 2021, **55:**663.

709. Rivera-Chavarria JP, Gutierrez-Lopez C, Castro-Cordero JA, Jimenez-Ramirez G: **Impact of COVID-19 on the surgical volume of general surgery residents as main surgeons in a National Training Program in Costa Rica: A cross-sectional study.** *Medicine (Baltimore)* 2021, **100:**e27041.

710. Kasai H, Shikino K, Saito G, Tsukamoto T, Takahashi Y, Kuriyama A, Tanaka K, Onodera M, Yokoh H, Tatusmi K, et al: **Alternative approaches for clinical clerkship during the COVID-19 pandemic: online simulated clinical practice for inpatients and outpatients-A mixed method.** *BMC Med Educ* 2021, **21:**149.

711. Kaur H, Singh A, Mahajan S, Lal M, Singh G, Kaur P: **Assessment of barriers and motivators to online learning among medical undergraduates of Punjab.** *J Educ Health Promot* 2021, **10:**123.

712. Keskin G: **Self-Report Measurement of Depression, Anxiety, and Stress Caused by COVID-19 Pandemic in Senior Undergraduate Dental Students.** *Pesquisa Brasileira em Odontopediatria e Clínica Integrada* 2021, **21**.

713. Khalid AM ea: **Impact of learning portfolio in structuring routine of medical students during covid-19 lockdown period.** *Rawal Medical Journal*.

714. Khan A, Mao JZ, Soliman MAR, Rho K, Hess RM, Reynolds RM, Riley JP, Mullin JP, Siddiqui AH, Levy EI, Pollina J: **The effect of COVID-19 on trainee operative experience at a multihospital academic neurosurgical practice: A first look at case numbers.** *Surg Neurol Int* 2021, **12:**271.

715. Khan R, Tandon P, Scaffidi MA, Bishay K, Pawlak KM, Kral J, Amin S, Bilal M, Lui RN, Sandhu DS, et al: **COVID-19 and Canadian Gastroenterology Trainees.** *J Can Assoc Gastroenterol* 2021, **4:**156-162.

716. Kheirallah K, Bloukh S, Khasawneh W, Alsulaiman J, Khassawneh A, Al-Mistarehi AH, Alqudah M, Elsalem L, Al Bashir S, Awad HH, et al: **Medical students' relative immunity, or lack thereof, against COVID-19 emotional distress and psychological challenges; a descriptive study from Jordan.** *F1000Res* 2021, **10:**297.

717. Kilgore LJ, Murphy BL, Postlewait LM, Liang DH, Bedrosian I, Lucci A, Kuerer HM, Hunt KK, Teshome M: **Impact of the early COVID-19 pandemic on Breast Surgical Oncology fellow education.** *J Surg Oncol* 2021, **124:**989-994.

718. Kim SY, Kim SJ, Lee SH: **Effects of Online Learning on Nursing Students in South Korea during COVID-19.** *Int J Environ Res Public Health* 2021, **18**.

719. Kohan L, Durbhakula S, Zaidi M, Phillips CR, Rowan CC, Brenner GJ, Cohen SP: **Changes in Pain Medicine Training Programs Associated With COVID-19: Survey Results.** *Anesth Analg* 2021, **132:**605-615.

720. Laloo R, Santhosh Karri R, Wanigasooriya K, Beedham W, Darr A, Layton GR, Logan P, Tan Y, Mittapalli D, Patel T, et al: **The perceived global impact of the COVID-19 pandemic on doctors' medical and surgical training: An international survey.** *Int J Clin Pract* 2021, **75:**e14314.

721. Lazarus G, Findyartini A, Putera AM, Gamalliel N, Nugraha D, Adli I, Phowira J, Azzahra L, Ariffandi B, Widyahening IS: **Willingness to volunteer and readiness to practice of undergraduate medical students during the COVID-19 pandemic: a cross-sectional survey in Indonesia.** *BMC Med Educ* 2021, **21:**138.

722. Lou SS, Goss CW, Evanoff BA, Duncan JG, Kannampallil T: **Risk factors associated with physician trainee concern over missed educational opportunities during the COVID-19 pandemic.** *BMC Med Educ* 2021, **21:**216.

723. Mladenovic R, Matvijenko V, Subaric L, Mladenovic K: **Augmented reality as e-learning tool for intraoral examination and dental charting during COVID-19 era.** *J Dent Educ* 2022, **86 Suppl 1:**862-864.

724. Mohanty A, Caldwell DJ, Hadley CC, Gibson A, Ravanpay A, Patel AJ: **Virtual Interviews in Neurosurgery Resident Selection-A Work in Progress.** *World Neurosurg* 2021, **155:**e412-e417.

725. Nugroho SW, Pradhana I, Gunawan K: **New adaptation of neurosurgical practice and residency programs during the Covid-19 pandemic and their effects on neurosurgery resident satisfaction and welfare at the National General Hospital, Jakarta, Indonesia.** *Heliyon* 2021, **7:**e07757.

726. O'Byrne L, Gavin B, Adamis D, Lim YX, McNicholas F: **Levels of stress in medical students due to COVID-19.** *J Med Ethics* 2021.

727. Owais Nasim HM ea: **Real-time measurement of psychological impact due to E- Learning; among the undergraduate dental students during Covid-19.** *European Journal of Molecular and Clinical Medicine*.

728. Prince S, Adhiyaman V: **What was the impact of COVID-19 on the foundation training programme in north Wales?** *Future Healthc J* 2021, **8:**e5-e6.

729. Rani V, Bethi M: **Perception of E-learning among undergraduate medical and dental students during COVID-19 pandemic - A cross-sectional study.** *National Journal of Physiology, Pharmacy and Pharmacology* 2021.

730. Ray I, Agarwal V, Agarwal T, Pande A: **Medical Student's Perspective Regarding Undergraduate Surgical Education with Special Reference to Pandemic.** *Indian J Surg* 2021**:**1-5.

731. Robertson JC, Woodfin M, Bonomo L, Feng S, Shinohara MM: **The impact of the COVID-19 pandemic on US dermatology resident training and wellness.** *Int J Dermatol* 2021, **60:**e338-e340.

732. Rojek NW, Madigan LM, Seminario-Vidal L, Atwater AR, Fett NM, Milani-Nejad N, Kaffenberger BH: **A virtual faculty exchange program enhances dermatology resident education in the COVID-19 era: a survey study.** *Dermatol Online J* 2021, **27**.

733. Rose CC, Haas MRC, Yilmaz Y, Alvarez A, Mott SE, Landry AI, Gisondi MA, Ankel F, Lin M, Chan TM: **ALiEM Connect: Large-Scale, Interactive, Virtual Residency Programming in Response to COVID-19.** *Acad Med* 2021, **96:**1419-1424.

734. Rossettini G, Geri T, Turolla A, Viceconti A, Scuma C, Mirandola M, Dell'Isola A, Gianola S, Maselli F, Palese A: **Online teaching in physiotherapy education during COVID-19 pandemic in Italy: a retrospective case-control study on students' satisfaction and performance.** *BMC Med Educ* 2021, **21:**456.

735. Rufach D, Santos S, Terebiznik M: **Simulation of pediatric intubation using a low-cost videolaryngoscope in the setting of the COVID-19 pandemic.** *Arch Argent Pediatr* 2021, **119:**270-272.

736. Runge A, Wray A, Harding C: **Virtual COVID rounds: A curricular enrichment program for pre-clinical medical students.** *Med Educ* 2021, **55:**661.

737. Sandal S, Boyarsky BJ, Cantarovich M: **The higher impact of the COVID-19 pandemic on resident/fellow training in low- and middle-income countries.** *Transpl Int* 2021, **34:**988-989.

738. Santamaria E, Nahas-Combina L, Altamirano-Arcos C, Vargas-Flores E: **Seven steps to deliver a low-cost, efficient, and high-impact online plastic surgery course during COVID-19 confinement: master series microsurgery for residents' experience.** *Arch Plast Surg* 2021, **48:**462-466.

739. Saravanan K ea: **Depression and anxiety among students community during COVID-19 pandemic lockdown in Tamil nadu- A web based descriptive cross sectional study.** *European Journal of Molecular and Clinical Medicine*.

740. Sarode R ea: **Psychosocial Impact of Lockdown Due to Novel Corona Virus Covid 19 among Medical Undergraduate Students.** *Indian Journal of Forensic Medicine & Toxicology* 2021.

741. Savage A, Minshew LM, Anksorus HN, McLaughlin JE: **Remote OSCE Experience: What First Year Pharmacy Students Liked, Learned, and Suggested for Future Implementations.** *Pharmacy (Basel)* 2021, **9**.

742. Schwartz DA, Connerney MA, Davila-Molina M, Tummalapalli SL: **Resident Mental Health at the Epicenter of the COVID-19 Pandemic.** *Acad Med* 2021, **96:**e16.

743. Seehusen DA, Kost A, Barr WB, Theobald M, Harper DM, Eden AR: **Family Medicine Residents' Experience During Early Phases of the COVID-19 Pandemic.** *PRiMER* 2021, **5:**18.

744. Shappell E, Egan DJ, Eyre A, Nadel E, Wittels K: **Virtual student experiences: A case study of objectives, outcomes, and anticipated consequences.** *AEM Educ Train* 2021, **5:**e10582.

745. Singh H, Olberding L, Al Jammali Z, Steinkopf M, Bookman N, Ostrogorsky TL, Eckrich M, McCracken C, Olstad S: **Zooming forward: An advanced pharmacy practice experience utilizing virtual**

**case‐based**

**learning in response to**

**COVID**

**‐19.** *Jaccp: Journal of the American College of Clinical Pharmacy* 2021, **4:**184-194.

746. Singh N, DeMesa C, Pritzlaff S, Jung M, Green C: **Implementation of Virtual Multiple Mini-Interviews for Fellowship Recruitment.** *Pain Med* 2021, **22:**1717-1721.

747. Singh R, Baby B, Singh R, Suri A: **Role of virtual modules to supplement neurosurgery education during COVID-19.** *J Clin Neurosci* 2021, **91:**125-130.

748. Singh R, Subedi M, Pant S, Rai P, Gupta K, Thapa A, Singh K, Khan A, Adhikari K, Sharma S, et al: **Perception towards Online Teaching-learning in Medical Education among Medical Students during COVID-19 Outbreak in Nepal: A Descriptive Cross-sectional Study.** *JNMA J Nepal Med Assoc* 2021, **59:**128-133.

749. Singh V, Supehia S, Gupta PK, Narula H, Sharma M, Devi K, Bhute AR: **Effectiveness of video modules in infection control trainings during COVID-19 pandemic: A quasi-experimental study in tertiary care institute.** *J Educ Health Promot* 2021, **10:**183.

750. Smida M, Khoodoruth MAS, Al-Nuaimi SK, Al-Salihy Z, Ghaffar A, Khoodoruth WNC, Mohammed MFH, Ouanes S: **Coping strategies, optimism, and resilience factors associated with mental health outcomes among medical residents exposed to coronavirus disease 2019 in Qatar.** *Brain Behav* 2021, **11:**e2320.

751. Smood B, Spratt JR, Mehaffey JH, Luc JGY, Vinck EE, Lehtinen ML, Wallen TJ, Jenkinson CG, Kim W, Kesieme EB, et al: **COVID-19 and cardiothoracic surgery: Effects on training and workforce utilization in a global pandemic.** *J Card Surg* 2021, **36:**3296-3305.

752. Snekalatha S, Marzuk SM, Meshram SA, Maheswari KU, Sugapriya G, Sivasharan K: **Medical students' perception of the reliability, usefulness and feasibility of unproctored online formative assessment tests.** *Adv Physiol Educ* 2021, **45:**84-88.

753. Sobelman C, Richard K, McQuilkin P, Fahey N: **Adapting Helping Babies Breathe into a Virtual Curriculum: Methods, Results, and Lessons Learned.** *Glob Pediatr Health* 2021, **8:**2333794X211019698.

754. Sparkes D, Leong C, Sharrocks K, Wilson M, Moore E, Matheson NJ: **Rebooting medical education with virtual grand rounds during the COVID-19 pandemic.** *Future Healthc J* 2021, **8:**e11-e14.

755. Strangio A, Leo I, Spaccarotella CAM, Barilla F, Basso C, Calabro MP, Curcio A, Filardi PP, Mancone M, Mercuro G, et al: **Effects of the COVID-19 pandemic on the formation of fellows in training in cardiology.** *J Cardiovasc Med (Hagerstown)* 2021, **22:**711-715.

756. Sukumar S, Zakaria A, Lai CJ, Sakumoto M, Khanna R, Choi N: **Designing and Implementing a Novel Virtual Rounds Curriculum for Medical Students' Internal Medicine Clerkship During the COVID-19 Pandemic.** *MedEdPORTAL* 2021, **17:**11106.

757. Sveinsdottir H, Flygenring BG, Svavarsdottir MH, Thorsteinsson HS, Kristofersson GK, Bernharethsdottir J, Svavarsdottir EK: **Predictors of university nursing students burnout at the time of the COVID-19 pandemic: A cross-sectional study.** *Nurse Educ Today* 2021, **106:**105070.

758. Syed S, Rastogi A, Bansal A, Kumar A, Jindal A, Prakash A, Agarwal G, Varshney M: **Future of e-Learning in Medical Education—Perception, Readiness, and Challenges in a Developing Country.** *Frontiers in Education* 2021, **6**.

759. Taluja M, Khare P, Budholia P: **A study of impact of coronavirus disease-19 pandemic on behavior and teaching of medical students.** *National Journal of Physiology, Pharmacy and Pharmacology* 2021, **11**.

760. Tan SHX, Ansari A, Ali NMI, Yap AU: **Simulation design and students' satisfaction with home-based simulation learning in oral health therapy.** *J Dent Educ* 2021, **85:**847-855.

761. Tashkandi E: **E-Learning for Undergraduate Medical Students.** *Adv Med Educ Pract* 2021, **12:**665-674.

762. Tiaprapong K, Sirikul A, Krajangmek C, Duangthongkul N, Pandam N, Piya-Amornphan N: **Awareness of COVID-19 influences on the wellness of Thai health professional students: An ambulatory assessment during the early "new normal" informing policy.** *PLoS One* 2021, **16:**e0252681.

763. Tolani MA, Fidelis L, Oyelowo N, Mustapha A, Adebayo WO, Okeke CJ, Alioke, II, Abdulsalam KI, Aruna AA, Okonji NO, Okeke UA: **Impact of the COVID-19 pandemic on surgical practice, training, and research in Nigeria.** *Pan Afr Med J* 2021, **39:**59.

764. Toth D, Petrus K, Heckmann V, Simon G, Poor VS: **Application of photogrammetry in forensic pathology education of medical students in response to COVID-19.** *J Forensic Sci* 2021, **66:**1533-1537.

765. Totlis T, Tishukov M, Piagkou M, Kostares M, Natsis K: **Online educational methods vs. traditional teaching of anatomy during the COVID-19 pandemic.** *Anat Cell Biol* 2021, **54:**332-339.

766. van Bonn SM, Grajek JS, Schneider A, Oberhoffner T, Mlynski R, Weiss NM: **Interactive live-stream surgery contributes to surgical education in the context of contact restrictions.** *Eur Arch Otorhinolaryngol* 2022, **279:**2865-2871.

767. Vandenberg S, Magnuson M: **A comparison of student and faculty attitudes on the use of Zoom, a video conferencing platform: A mixed-methods study.** *Nurse Educ Pract* 2021, **54:**103138.

768. Varvara G, Bernardi S, Bianchi S, Sinjari B, Piattelli M: **Dental Education Challenges during the COVID-19 Pandemic Period in Italy: Undergraduate Student Feedback, Future Perspectives, and the Needs of Teaching Strategies for Professional Development.** *Healthcare (Basel)* 2021, **9**.

769. Vatier C, Carrie A, Renaud MC, Simon-Tillaux N, Hertig A, Jeru I: **Lessons from the impact of COVID-19 on medical educational continuity and practices.** *Adv Physiol Educ* 2021, **45:**390-398.

770. Velarde-Garcia JF, Cachon-Perez JM, Rodriguez-Garcia M, Oliva-Fernandez O, Gonzalez-Sanz P, Espejo MM, Gonzalez-Hervias R, Alvarez-Embarba B, Moro-Lopez-Menchero P, Fernandez-de-Las-Penas C, Palacios-Cena D: **The challenges of "learning on the go": A qualitative study of final-year Spanish nursing students incorporated to work during the first Covid-19 pandemic.** *Nurse Educ Today* 2021, **103:**104942.

771. Venincasa MJ, Steren B, Young BK, Parikh A, Ahmed B, Sridhar J, Kombo N: **Ophthalmology Residency Match in the Covid-19 Era: Applicant and Program Director Perceptions of the 2020-2021 Application Cycle.** *Semin Ophthalmol* 2022, **37:**36-41.

772. C.S V: **Technology Enhanced Neuroanatomy Learning during Covid-19 Pandemic - A Questionnaire Based Study from a Teaching Hospital in Mysore, Karnataka, India.** *Journal of Evolution of Medical and Dental Sciences* 2021, **10:**1130-1134.

773. Vishwanathan K, Patel GM, Patel DJ: **Medical faculty perception toward digital teaching methods during COVID-19 pandemic: Experience from India.** *J Educ Health Promot* 2021, **10:**95.

774. Vogt L, Schmidt M, Klasen M, Bickenbach J, Marx G, Sopka S: **[Medical students as helpers in the pandemic : Innovative concept for recruitment, training and assignment planning of medical students as medical personnel during the COVID-19 pandemic].** *Anaesthesist* 2022, **71:**21-29.

775. Wallace D, Sturrock A, Gishen F: **'You've got mail!': Clinical and practical skills teaching re-imagined during COVID-19.** *Future Healthc J* 2021, **8:**e50-e53.

776. Wallace S, Schuler MS, Kaulback M, Hunt K, Baker M: **Nursing student experiences of remote learning during the COVID-19 pandemic.** *Nurs Forum* 2021, **56:**612-618.

777. Wang J, Bu L, Li Y, Song J, Li N: **The mediating effect of academic engagement between psychological capital and academic burnout among nursing students during the COVID-19 pandemic: A cross-sectional study.** *Nurse Educ Today* 2021, **102:**104938.

778. Wang Y, Yu R, Liu Y, Qian W: **Students' and Teachers' Perspective on the Implementation of Online Medical Education in China: A Qualitative Study.** *Adv Med Educ Pract* 2021, **12:**895-903.

779. Wu BJ, Honan L, Tinetti ME, Marottoli RA, Brissette D, Wilkins KM: **The virtual 4Ms: A novel curriculum for first year health professional students during COVID-19.** *J Am Geriatr Soc* 2021, **69:**E13-E16.

780. Xie C, Huang C, Yang X, Luo D, Liu Z, Tu S, Jie K, Xiong X: **Innovations in education of the medical molecular biology curriculum during the COVID-19 pandemic in China.** *Biochem Mol Biol Educ* 2021, **49:**720-728.

781. Xiong P, Ming WK, Zhang C, Bai J, Luo C, Cao W, Zhang F, Tao Q: **Factors Influencing Mental Health Among Chinese Medical and Non-medical Students in the Early Stage of the COVID-19 Pandemic.** *Front Public Health* 2021, **9:**603331.

782. Yadav RK, Baral S, Khatri E, Pandey S, Pandeya P, Neupane R, Yadav DK, Marahatta SB, Kaphle HP, Poudyal JK, Adhikari C: **Anxiety and Depression Among Health Sciences Students in Home Quarantine During the COVID-19 Pandemic in Selected Provinces of Nepal.** *Front Public Health* 2021, **9:**580561.

783. Yekefallah L, Namdar P, Panahi R, Dehghankar L: **Factors related to students' satisfaction with holding e-learning during the Covid-19 pandemic based on the dimensions of e-learning.** *Heliyon* 2021, **7:**e07628.

784. Research Education IiSiC-g, Yiasemidou M, Tomlinson J, Chetter I, Biyani CS: **Impact of the SARS-CoV-2 (COVID-19) crisis on surgical training: global survey and a proposed framework for recovery.** *BJS Open* 2021, **5**.

785. Yildirim TT, Atas O: **The evaluation of psychological state of dental students during the COVID-19 pandemic.** *Braz Oral Res* 2021, **35:**e069.

786. Yilmaz Y, Sarikaya O, Senol Y, Baykan Z, Karaca O, Demiral Yilmaz N, Altintas L, Onan A, Sayek I: **RE-AIMing COVID-19 online learning for medical students: a massive open online course evaluation.** *BMC Med Educ* 2021, **21:**303.

787. Yin Y, Yang X, Gao L, Zhang S, Qi M, Zhang L, Tan Y, Chen J: **The Association Between Social Support, COVID-19 Exposure, and Medical Students' Mental Health.** *Front Psychiatry* 2021, **12:**555893.

788. Yu F, Wooster J, Yang T: **Pharmacy students and faculty perceptions of online team-based learning due to the COVID-19 pandemic.** *Pharmacy Education* 2021, **21:**121-125.

789. Yu JL, Cho DY, DeSanti RL, Kneib CJ, Friedrich JB, Colohan SM: **Resident experiences with virtual education during the COVID-19 crisis.** *J Plast Reconstr Aesthet Surg* 2021, **74:**1931-1971.

790. Yu L, Huang L, Tang HR, Li N, Rao TT, Hu D, Wen YF, Shi LX: **Analysis of factors influencing the network teaching effect of college students in a medical school during the COVID-19 epidemic.** *BMC Med Educ* 2021, **21:**397.

791. Yu Q, Liu L, Tang Q, Wu W: **Online teaching- present situation and its future: a survey of online study for medical students during the COVID-19 epidemic.** *Irish Educational Studies* 2021, **40:**207-215.

792. Yu-Fong Chang J, Wang LH, Lin TC, Cheng FC, Chiang CP: **Comparison of learning effectiveness between physical classroom and online learning for dental education during the COVID-19 pandemic.** *J Dent Sci* 2021, **16:**1281-1289.

793. Yuda Handaya A, Fauzi AR, Andrew J, Hanif AS, Tjendra KR, Aditya AFK: **Effectiveness of tutorial videos combined with online classes in surgical knotting course during COVID-19 pandemic: A cohort study.** *Ann Med Surg (Lond)* 2021, **69:**102751.

794. Yun JY, Kim JW, Myung SJ, Yoon HB, Moon SH, Ryu H, Yim JJ: **Impact of COVID-19 on Lifestyle, Personal Attitudes, and Mental Health Among Korean Medical Students: Network Analysis of Associated Patterns.** *Front Psychiatry* 2021, **12:**702092.

795. Zaidman M, Al-Shaqsi S, Yeung C, Novak CB, Dengler J: **COVID-19 Restrictions Presented Opportunities and Challenges for Plastic Surgery Residents.** *Plast Surg (Oakv)* 2021, **29:**294-302.

796. Zamberg I, Schiffer E, Stoermann-Chopard C: **Novice and Advanced Learners' Satisfaction and Perceptions of an e-Learning Renal Semiology Module During the COVID-19 Pandemic: Mixed Methods Study.** *JMIR Med Educ* 2021, **7:**e29216.

797. Zaragoza-Garcia I, Ortuno-Soriano I, Posada-Moreno P, Sanchez-Gomez R, Raurell-Torreda M: **Virtual Simulation for Last-Year Nursing Graduate Students in Times of Covid-19: A Quasi-Experimental Study.** *Clin Simul Nurs* 2021, **60:**32-41.

798. Zarzecka J, Zarzecka-Francica E, Gala A, Gebczynski K, Pihut M: **Dental environmental stress during the COVID19 pandemic at the Jagiellonian University Medical College, Krakow, Poland.** *Int J Occup Med Environ Health* 2021, **34:**211-222.

799. Zavitz J, Sarwal A, Schoeneck J, Glass C, Hays B, Shen E, Bryant C, Gupta K: **Virtual multispecialty point-of-care ultrasound rotation for fourth-year medical students during COVID-19: Innovative teaching techniques improve ultrasound knowledge and image interpretation.** *AEM Educ Train* 2021, **5:**e10632.

800. Zemela MS, Malgor RD, Smith BK, Smeds MR: **Feasibility and acceptability of virtual mock oral examinations for senior vascular surgery trainees and implications for the certifying exam.** *Ann Vasc Surg* 2021, **76:**28-37.

801. Zhang K, Peng Y, Zhang X, Li L: **Psychological Burden and Experiences Following Exposure to COVID-19: A Qualitative and Quantitative Study of Chinese Medical Student Volunteers.** *Int J Environ Res Public Health* 2021, **18**.

802. Zhang LM, Symalla T, Roggin KK, Matthews JB, Hussain M: **Creation of a COVID-19 Based Educational Curriculum: A Blueprint for Redesigning Surgical Education During Times of National Crisis.** *Annals of Surgery Open* 2021, **2**.

803. Zhang X, Tong N, Shao Q, Zhang Y: **Medical clerkships in China in a single institution since the outbreak of COVID-19.** *Med Educ* 2021, **55:**1301-1302.

804. Zhao D, Yu J, Zhang T, Du M, Yang Q, Li Z, Fouad AF: **Impact of COVID-19 on advanced dental education: Perspectives of dental residents in Wuhan.** *J Dent Educ* 2021, **85:**756-767.

805. Zhao L, Sznajder K, Cheng D, Wang S, Cui C, Yang X: **Coping Styles for Mediating the Effect of Resilience on Depression Among Medical Students in Web-Based Classes During the COVID-19 Pandemic: Cross-sectional Questionnaire Study.** *J Med Internet Res* 2021, **23:**e25259.

806. Zhou B, Calkins C, Jayaraman T, Cassells S, Rotto T, Vaughan L, Srinivasan M, Schillinger E: **Implementing Value-Added Medical Education: Lessons Learned From the Student-Initiated Stanford Frontline COVID-19 Consult Service.** *Acad Med* 2021, **96:**1690-1695.

807. Zhu M, Zhang Y: **Medical and public health instructors' perceptions of online teaching: A qualitative study using the Technology Acceptance Model 2.** *Educ Inf Technol (Dordr)* 2022, **27:**2385-2405.

808. Zhu Y, Wang H, Wang A: **An evaluation of mental health and emotion regulation experienced by undergraduate nursing students in China during the COVID-19 pandemic: A cross-sectional study.** *Int J Ment Health Nurs* 2021, **30:**1160-1169.

809. Zoorob D, Shah S, La Saevig D, Murphy C, Aouthmany S, Brickman K: **Insight into resident burnout, mental wellness, and coping mechanisms early in the COVID-19 pandemic.** *PLoS One* 2021, **16:**e0250104.

810. Zuljevic MF, Jelicic K, Vidak M, Dogas V, Buljan I: **Impact of the first COVID-19 lockdown on study satisfaction and burnout in medical students in Split, Croatia: a cross-sectional presurvey and postsurvey.** *BMJ Open* 2021, **11:**e049590.

811. AlOmar RS, AlShamlan NA, AlAmer NA, Aldulijan F, AlMuhaidib S, Almukhadhib O, Algarni SA, Alshaibani A, Darwish M, Al Shammari M: **What are the barriers and facilitators of volunteering among healthcare students during the COVID-19 pandemic? A Saudi-based cross-sectional study.** *BMJ Open* 2021, **11:**e042910.

812. Alsoufi A, Alsuyihili A, Msherghi A, Elhadi A, Atiyah H, Ashini A, Ashwieb A, Ghula M, Ben Hasan H, Abudabuos S, et al: **Impact of the COVID-19 pandemic on medical education: Medical students' knowledge, attitudes, and practices regarding electronic learning.** *PLoS One* 2020, **15:**e0242905.

813. De la Cerda-Vargas MF, Stienen MN, Soriano-Sanchez JA, Campero A, Borba LAB, Nettel-Rueda B, Castillo-Rangel C, Ley-Urzaiz L, Ramirez-Silva LH, Sandoval-Bonilla BA: **Impact of the Coronavirus Disease 2019 Pandemic on Working and Training Conditions of Neurosurgery Residents in Latin America and Spain.** *World Neurosurg* 2021, **150:**e182-e202.

814. Godoy IRB, Neto LP, Skaf A, Leao-Filho HM, Freddi TAL, Jasinowodolinski D, Yamada AF: **Audiovisual Content for a Radiology Fellowship Selection Process During the COVID-19 Pandemic: Pilot Web-Based Questionnaire Study.** *JMIR Med Educ* 2021, **7:**e28733.

815. Grant LL, Opperman MJ, Schiller B, Chastain J, Richardson JD, Eckel C, Plawecki MH: **Medical Student Engagement in a Virtual Learning Environment Positively Correlates with Course Performance and Satisfaction in Psychiatry.** *Med Sci Educ* 2021, **31:**1133-1140.

816. Guadalajara H, Palazon A, Lopez-Fernandez O, Esteban-Flores P, Garcia JM, Gutierrez-Misis A, Baca-Garcia E, Garcia-Olmo D: **Towards an Open Medical School without Checkerboards during the COVID-19 Pandemic: How to Flexibly Self-Manage General Surgery Practices in Hospitals?** *Healthcare (Basel)* 2021, **9**.

817. Gunadi, Balela N, Kalim AS, Widitjiarso W, Fahri F, Tedja AK, Purnomo E, Dwihantoro A, Agustriani N, Makhmudi A: **The COVID-19 pandemic impact on pediatric surgery residency programs.** *Heliyon* 2021, **7:**e07199.

818. Guo AA, Crum MA, Fowler LA: **Assessing the Psychological Impacts of COVID-19 in Undergraduate Medical Students.** *Int J Environ Res Public Health* 2021, **18**.

819. Gupta PC, Singh R, Khurana S, Behera RK, Thattaruthody F, Pandav SS, Ram J: **Reworking protocols of ophthalmic resident surgical training in the COVID-19 era - Experiences of a tertiary care institute in northern India.** *Indian J Ophthalmol* 2021, **69:**1928-1932.

820. Hakim: **Physiotherapy student's preference for learning during Covid- 19 pandemic.** *Turkish Journal of Physiotherapy and Rehabilitation*.

821. Haley C, Lee J, Xun H, Yesantharao P, Nolan IT, Harirah M, Crowe CS, Lopez J, Morrison SD, Drolet BC, Janis JE: **The Negative Impact of COVID-19 on Medical Education amongst Medical Students Interested in Plastic Surgery: A Cross-sectional Survey Study.** *Plast Reconstr Surg Glob Open* 2021, **9:**e3535.

822. Hamade N, Bhavsar-Burke I, Jansson-Knodell C, Wani S, Patel SG, Ehrlich AC, Paine E, Hosseini-Carroll P, Menard-Katcher P, Fayad N: **Virtual Gastroenterology Fellowship Recruitment During COVID-19 and Its Implications for the Future.** *Dig Dis Sci* 2022, **67:**2019-2028.

823. Harrell KM, McGinn MJ, Edwards CD, Warren Foster K, Meredith MA: **Crashing from cadaver to computer: Covid-driven crisis-mode pedagogy spawns active online substitute for teaching gross anatomy.** *Anat Sci Educ* 2021, **14:**536-551.

824. Hassani K, McElroy T, Coop M, Pellegrin J, Wu WL, Janke RD, Johnson LK: **Rapid Implementation and Evaluation of Virtual Health Training in a Subspecialty Hospital in British Columbia, in Response to the COVID-19 Pandemic.** *Front Pediatr* 2021, **9:**638070.

825. Hojilla C, Armstrong S, Pun C, Hickey TBM, Mete O, Han R, Hahn E, Shivji S, Done S, Lu FI: **A Holistic Approach to Pathology Education During the Coronavirus Disease 2019 (COVID-19) Pandemic.** *Arch Pathol Lab Med* 2021, **145:**923-924.

826. Holmberg MH, Dela Cruz E, Longino A, Longino N, Coruh B, Merel SE: **Development of a Single-Institution Virtual Internal Medicine Subinternship With Near-Peer Teaching in Response to the COVID-19 Pandemic.** *Acad Med* 2021, **96:**1706-1710.

827. Hosny S, Ghaly M, Hmoud AlSheikh M, Shehata MH, Salem AH, Atwa H: **Developing, Validating, and Implementing a Tool for Measuring the Readiness of Medical Teachers for Online Teaching Post-COVID-19: A Multicenter Study.** *Adv Med Educ Pract* 2021, **12:**755-768.

828. Howard V, Hartman AM, Allen DH, Reynolds SS: **Student nurse perceptions of an innovative role to support clinical practices during a pandemic: A qualitative study.** *Nurse Educ Today* 2021, **103:**104959.

829. Huarcaya-Victoria J, Elera-Fitzcarrald C, Crisol-Deza D, Villanueva-Zuniga L, Pacherres A, Torres A, Huertas G, Calderon D, Noriega-Baella C, Astonitas E, Salvador-Carrillo J: **Factors associated with mental health in Peruvian medical students during the COVID-19 pandemic: a multicentre quantitative study.** *Rev Colomb Psiquiatr (Engl Ed)* 2021.

830. Hussain A, Chau J, Bang H, Meyer L, Islam M: **Readiness, Reception, and Performance of Students in a Communications Course Delivered Amid the Pandemic.** *Am J Pharm Educ* 2021**:**8617.

831. Ienghong K, Suzuki T, Tiamkao S, Bhudhisawasdi V, Gaysonsiri D, Apiratwarakul K: **Point of Care Ultrasound Training during the Coronavirus Disease 2019 Pandemic.** *Open Access Macedonian Journal of Medical Sciences* 2021, **9:**443-446.

832. Ikram F, Rabbani MA: **Academic Integrity in Traditional Vs Online Undergraduate Medical Education Amidst COVID-19 Pandemic.** *Cureus* 2021, **13:**e13911.

833. Ion R, Craswell A, Hughes L, Johnston A, Kilbride L, Hubbard-Murdoch N, Massey D: **International nurse education leaders' experiences of responding to the COVID-19 pandemic: A qualitative study.** *J Adv Nurs* 2021, **77:**3797-3805.

834. Iosif L, Tancu AMC, Didilescu AC, Imre M, Galbinasu BM, Ilinca R: **Self-Perceived Impact of COVID-19 Pandemic by Dental Students in Bucharest.** *Int J Environ Res Public Health* 2021, **18**.

835. Isaradisaikul SK, Thansuwonnont P, Sangthongluan P: **Impact of COVID-19 pandemic on happiness and stress: comparison of preclinical and clinical medical students.** *Korean J Med Educ* 2021, **33:**75-85.

836. Ismail, II, Abdelkarim A, Al-Hashel JY: **Physicians' attitude towards webinars and online education amid COVID-19 pandemic: When less is more.** *PLoS One* 2021, **16:**e0250241.

837. Iurcov R, Pop LM, Iorga M: **Impact of COVID-19 Pandemic on Academic Activity and Health Status among Romanian Medical Dentistry Students; A Cross-Sectional Study.** *Int J Environ Res Public Health* 2021, **18**.

838. Johnston JP, Andrews LB, Adams CD, Cardinale M, Dixit D, Effendi MK, Tompkins DM, Wilczynski JA, Opsha Y: **Implementation and evaluation of a virtual learning advanced pharmacy practice experience.** *Curr Pharm Teach Learn* 2021, **13:**862-867.

839. Kalchiem-Dekel O, Schwalk AJ, Patel NM, Lin IH, Beattie JA, Husta BC, Chawla M, Sachdeva A, Akulian JA, Musani AI, et al: **COVID-19 Impact on Interventional Pulmonology Training.** *ATS Sch* 2021, **2:**236-248.

840. Kamboj AK, Raffals LE, Martin JA, Chandrasekhara V: **Virtual Interviews During the COVID-19 Pandemic: A Survey of Advanced Endoscopy Fellowship Applicants and Programs.** *Tech Innov Gastrointest Endosc* 2021, **23:**159-168.

841. Kang YJ, Kim DH: **Pre-clerkship students' perception and learning behavior of online classes during coronavirus disease 2019 pandemic.** *Korean J Med Educ* 2021, **33:**125-131.

842. Kaplan CA, Chan CC, Feingold JH, Kaye-Kauderer H, Pietrzak RH, Peccoralo L, Feder A, Southwick S, Charney D, Burka L, et al: **Psychological Consequences Among Residents and Fellows During the COVID-19 Pandemic in New York City: Implications for Targeted Interventions.** *Acad Med* 2021, **96:**1722-1731.

843. Kattan AE, Mortada H, Alzaidi S, Gelidan AG: **Perceptions of Saudi Plastic Surgery Residents and Attendings of Online Education during the COVID-19 Pandemic.** *Plast Reconstr Surg Glob Open* 2021, **9:**e3658.

844. Kawasaki H, Yamasaki S, Masuoka Y, Iwasa M, Fukita S, Matsuyama R: **Remote Teaching Due to COVID-19: An Exploration of Its Effectiveness and Issues.** *Int J Environ Res Public Health* 2021, **18**.

845. Khan AM, Patra S, Gupta P, Sharma AK, Jain AK: **Rapid transition to online teaching program during COVID-19 lockdown: Experience from a medical college of India.** *J Educ Health Promot* 2021, **10:**99.

846. Khapre M, Sinha S, Kaushal P: **Effectiveness of Integrated Google Classroom, Reciprocal Peer Teaching and Flipped Classroom on Learning Outcomes of Research Methodology: A Natural Experiment.** *Cureus* 2021, **13:**e16176.

847. Kilcoyne MF, Coyan GN, Aranda-Michel E, Kilic A, Morell VO, Sultan I: **The impact of coronavirus 2019 on general surgery residency: A national survey of program directors.** *Ann Med Surg (Lond)* 2021, **65:**102285.

848. Klaassen H, Ashida S, Comnick CL, Xie XJ, Smith BM, Tabrizi M, Arsenault K, Capin OR, Scully AC, da Mata C, et al: **COVID-19 pandemic and its impact on dental students: A multi-institutional survey.** *J Dent Educ* 2021, **85:**1280-1286.

849. Koster MA, Soffler M: **Navigate the Challenges of Simulation for Assessment: A Faculty Development Workshop.** *MedEdPORTAL* 2021, **17:**11114.

850. Kosydar S, Woodfin MW, Halasz LM, Apisarnthanarax S, Rengan R, Lo SS: **The Impact of COVID-19 on US Radiation Oncology Residents.** *J Cancer Educ* 2022, **37:**1525-1531.

851. Kovacs E, Kallai A, Frituz G, Ivanyi Z, Miko V, Valko L, Hauser B, Gal J: **The efficacy of virtual distance training of intensive therapy and anaesthesiology among fifth-year medical students during the COVID-19 pandemic: a cross-sectional study.** *BMC Med Educ* 2021, **21:**393.

852. Kumar P, Kumar A, Rahul R, Rastogi D, Singh J, Gupta A, Srivastava C: **Preferred online teaching and assessment methods among Indian medical graduates in coronavirus disease era.** *National Journal of Physiology, Pharmacy and Pharmacology* 2020, **11**.

853. Kumar PR, Hashmi Y, Beedham W, Kumar RV, Fleming S: **F1-taught orthopaedic teaching programme for students (FOTS).** *Postgrad Med J* 2022, **98:**710-717.

854. Lahon J, Shukla P, Singh A, Jain A, Brahma D, Adhaulia G, Kamal P, Abbas A: **Coronavirus disease 2019 pandemic and medical students' knowledge, attitude, and practice on online teaching-learning process in a medical institute in North India.** *National Journal of Physiology, Pharmacy and Pharmacology* 2021, **11**.

855. Larocque N, Shenoy-Bhangle A, Brook A, Eisenberg R, Chang YM, Mehta P: **Resident Experiences With Virtual Radiology Learning During the COVID-19 Pandemic.** *Acad Radiol* 2021, **28:**704-710.

856. Lee CM, Juarez M, Rae G, Jones L, Rodriguez RM, Davis JA, Boysen-Osborn M, Kashima KJ, Krane NK, Kman N, et al: **Anxiety, PTSD, and stressors in medical students during the initial peak of the COVID-19 pandemic.** *PLoS One* 2021, **16:**e0255013.

857. Lee DW, Deer T: **Pain Fellows Survey: COVID-19 Impact on the Training and Job Market.** *Pain Med* 2021, **22:**3106-3109.

858. Lei T, Yu X, Zou M, Wang P, Yuan RH: **Delivering an online course in emergency nursing education during the pandemic: What are the effects on students' learning?** *Australas Emerg Care* 2021, **24:**314-318.

859. Leite AK, Matos LL, Cernea CR, Kowalski LP: **The Impact of the COVID-19 Pandemic on Head and Neck Surgery Training: A Brazilian National Survey.** *Int Arch Otorhinolaryngol* 2021, **25:**e339-e342.

860. Leon-Manco RA, Agudelo-Suarez AA, Armas-Vega A, Figueiredo MC, Verdugo-Paiva F, Santana-Perez Y, Viteri-Garcia A: **Perceived Stress in Dentists and Dental Students of Latin America and the Caribbean during the Mandatory Social Isolation Measures for the COVID-19 Pandemic: A Cross-Sectional Study.** *Int J Environ Res Public Health* 2021, **18**.

861. Leung HTT, Ajaz A, Bruce H, Korszun A: **Teaching psychiatry to medical students in the time of COVID-19: experiences from UK medical schools.** *BJPsych Bull* 2021**:**1-10.

862. Li W, Gillies R, He M, Wu C, Liu S, Gong Z, Sun H: **Barriers and facilitators to online medical and nursing education during the COVID-19 pandemic: perspectives from international students from low- and middle-income countries and their teaching staff.** *Hum Resour Health* 2021, **19:**64.

863. Li Y, Calle C, Chu C, Baussan C, Hampson LA: **CASE-based and Guidelines-based Lectures are the Most Preferred Form of Online Webinar Education: Results from the Urology Collaborative Online Video Didactics Series (COViD).** *Urology* 2021, **158:**52-56.

864. Li Z, Yi X, Zhong M, Li Z, Xiang W, Wu S, Xiong Z: **Psychological Distress, Social Support, Coping Style, and Perceived Stress Among Medical Staff and Medical Students in the Early Stages of the COVID-19 Epidemic in China.** *Front Psychiatry* 2021, **12:**664808.

865. Liles JL, Danilkowicz R, Dugas JR, Safran M, Taylor D, Amendola AN, Herzog M, Provencher MT, Lau BC: **In Response to COVID-19: Current Trends in Orthopaedic Surgery Sports Medicine Fellowships.** *Orthop J Sports Med* 2021, **9:**2325967120987004.

866. Lois F, Hallet C, Samalea Suarez N, Ghuysen A, Brichant JF: **In situ simulation improves perceived self-efficacy of OR nurses and anaesthesiologists during COVID-19 pandemic.** *BMJ Simul Technol Enhanc Learn* 2021, **7:**555-560.

867. Luc JGY, Nguyen TC, Ad N, Group C-NACSSW: **Impact of the Coronavirus Disease 2019 Pandemic on Cardiac Surgical Education in North America.** *Innovations (Phila)* 2021, **16:**350-357.

868. Lucas D, Brient S, Eveillard BM, Gressier A, Le Grand T, Pougnet R, Dewitte JD, Lodde B: **Health Impact and Psychosocial Perceptions among French Medical Residents during the SARS-CoV-2 Outbreak: A Cross-Sectional Survey.** *Int J Environ Res Public Health* 2021, **18**.

869. Luck J, Gosling N, Saour S: **Undergraduate surgical education during COVID-19: could augmented reality provide a solution?** *Br J Surg* 2021, **108:**e129-e130.

870. Lund S, MacArthur T, Keune J, Enger T, Martinez-Jorge J, McKenzie T, Nelson M, Olson A, Que F, Stulak J, Rivera M: **Showcasing a General Surgery Residency Program During the COVID-19 Pandemic.** *J Surg Educ* 2021, **78:**e28-e34.

871. Luo Y, Geng C, Pei X, Chen X, Zou Z: **The Evaluation of the Distance Learning Combining Webinars and Virtual Simulations for Senior Nursing Students during the COVID-19 Period.** *Clin Simul Nurs* 2021, **57:**31-40.

872. M VB, Arumugam MK, Debnath DJ: **Simulated Patient Environment: A Training Tool for Healthcare Professionals in COVID-19 Era.** *Adv Med Educ Pract* 2021, **12:**579-585.

873. Mahabamunuge J, Farmer L, Pessolano J, Lakhi N: **Implementation and Assessment of a Novel Telehealth Education Curriculum for Undergraduate Medical Students.** *J Adv Med Educ Prof* 2021, **9:**127-135.

874. Makaju S, Rai CK: **Virtual Anatomy Classes among the First and Second Year Medical and Dental Students of a Medical College: A Descriptive Cross-sectional Study.** *JNMA J Nepal Med Assoc* 2021, **59:**767-770.

875. Mallick R, Odejinmi F, Sideris M, Egbase E, Kaler M: **The impact of COVID-19 on obstetrics and gynaecology trainees; how do we move on?** *Facts Views Vis Obgyn* 2021, **13:**9-14.

876. Marcen-Roman Y, Gasch-Gallen A, Vela Martin de la M, II, Calatayud E, Gomez-Soria I, Rodriguez-Roca B: **Stress Perceived by University Health Sciences Students, 1 Year after COVID-19 Pandemic.** *Int J Environ Res Public Health* 2021, **18**.

877. Martin RL, 3rd, Grant MJ, Kimani S, Midha S, May J, Patell R, Collier E, Furfaro D, Bodine C, Reap L, et al: **Forming the Hematology-Oncology Collaborative Videoconferencing (CO-VID) Learning Initiative: Experiential Lessons Learned From a Novel Trainee-Led Multidisciplinary Virtual Learning Platform.** *JCO Oncol Pract* 2022, **18:**e36-e46.

878. Martin-Delgado L, Goni-Fuste B, Alfonso-Arias C, De Juan M, Wennberg L, Rodriguez E, Fuster P, Monforte-Royo C, Martin-Ferreres ML: **Nursing students on the frontline: Impact and personal and professional gains of joining the health care workforce during the COVID-19 pandemic in Spain.** *J Prof Nurs* 2021, **37:**588-597.

879. Matthiesen MI, Hiserodt J, Naureckas Li C, Frey-Vogel AS, Johnson JH: **Going Virtual: Objective Structured Teaching Exercises as an Innovative Method for Formative Resident Education.** *Acad Pediatr* 2022, **22:**12-16.

880. Mehrzad R, Akiki R, Crozier J, Schmidt S: **Mental Health Outcomes in Plastic Surgery Residents during the COVID-19 Pandemic.** *Plast Reconstr Surg* 2021, **148:**349e-350e.

881. Mehrzad R, Akiki R, Liu PY, Schmidt S, Woo AS: **Changes in Academic Plastic Surgery Programs During the COVID-19 Pandemic.** *Ann Plast Surg* 2021, **87:**S52-S56.

882. Mendonca VS, Steil A, Gois AFT: **Mental health and the COVID-19 pandemic: a study of medical residency training over the years.** *Clinics (Sao Paulo)* 2021, **76:**e2907.

883. Menon UK, Gopalakrishnan S, CS NU, Ramachandran R, Poornima B, Sasidharan A, Ashika MS, Radhakrishnan N: **Pilot of a questionnaire study regarding perception of undergraduate medical students towards online classes: Process and perspectives.** *J Family Med Prim Care* 2021, **10:**2016-2021.

884. Michel A, Ryan N, Mattheus D, Knopf A, Abuelezam NN, Stamp K, Branson S, Hekel B, Fontenot HB: **Undergraduate nursing students' perceptions on nursing education during the 2020 COVID-19 pandemic: A national sample.** *Nurs Outlook* 2021, **69:**903-912.

885. Miguel C, Castro L, Marques Dos Santos JP, Serrao C, Duarte I: **Impact of COVID-19 on Medicine Lecturers' Mental Health and Emergency Remote Teaching Challenges.** *Int J Environ Res Public Health* 2021, **18**.

886. Mihatsch L, von der Linde M, Knolle F, Luchting B, Dimitriadis K, Heyn J: **Survey of German medical students during the COVID-19 pandemic: attitudes toward volunteering versus compulsory service and associated factors.** *J Med Ethics* 2022, **48:**630-636.

887. Miles S, Donnellan N: **Learning Fundamentals of Laparoscopic Surgery Manual Skills: An Institutional Experience With Remote Coaching and Assessment.** *Mil Med* 2021.

888. Mills S, Cioletti A, Gingell G, Ramani S: **Training Residents in Virtual Advance Care Planning: A New Twist in Telehealth.** *J Pain Symptom Manage* 2021, **62:**691-698.

889. Mishra D, Nair AG, Verma L, Grover AK, Mathur S, Srivastav T: **The perceived impact of webinars during the COVID.19 pandemic: A survey of ophthalmology trainees from India.** *Oman J Ophthalmol* 2021, **14:**78-84.

890. Mishra J, Samanta P, Panigrahi A, Dash K, Behera MR, Das R: **Mental Health Status, Coping Strategies During Covid-19 Pandemic Among Undergraduate Students of Healthcare Profession.** *Int J Ment Health Addict* 2021**:**1-13.

891. Mondal D, Barthwal M, Singh N, Pareek V: **Effect of COVID-19 Pandemic on Oncology Residency Training in India and a Novel Online Academic Solution: Results of an Online Survey.** *Adv Radiat Oncol* 2021, **6:**100688.

892. Moore WJ, Webb A, Morrisette T, Sullivan LK, Alosaimy S, Hossain S, Howe Z, Vlashyn OO, Paloucek FP, Rybak MJ, Wang SK: **Impact of COVID-19 pandemic on training of pharmacy residents and fellows: Results from a national survey of postgraduate pharmacy trainees.** *Am J Health Syst Pharm* 2021, **78:**1104-1111.

893. Morgan G, Melson E, Davitadze M, Ooi E, Zhou D, Hanania T, Chen W, Thomas L, Allison I, Ding M, et al: **Utility of Simulation via Instant Messaging - Birmingham Advance (SIMBA) in medical education during COVID-19 pandemic.** *J R Coll Physicians Edinb* 2021, **51:**168-172.

894. Morgan G, Ooi E, Hanania T, Davitadze M, Zhou D, Blaggan P, Melson E, Arlt W, Boelaert K, Cooney R, et al: **Simulation via instant messaging - Birmingham advance (SIMBA): an innovative simulation-based learning model that helped to keep medical education continue during the COVID-19 pandemic.** *Clin Med (Lond)* 2021, **21:**34-35.

895. Moschetti WE, Frye BM, Gililland JM, Braziel AJ, Shah VM: **The Emergence of Collaboration in the Education of Fellows and Residents during COVID-19.** *J Arthroplasty* 2021, **36:**2223-2226.

896. Mubeen: **Analysis of the e-learning educational atmosphere during covid 19 pandemic: Empirical evidence from medical universities of urban pakistan.** *Medical Forum Monthly*.

897. Mukhopadhyay S, Joshi D, Goel G, Singhai A, Kapoor N: **Evolution of pathology teaching for MBBS students during COVID-19 pandemic lockdown: Moving from a real to a virtual classroom.** *Indian J Pathol Microbiol* 2021, **64:**524-527.

898. Musa D, Gonzalez L, Penney H, Daher S: **Interactive Video Simulation for Remote Healthcare Learning.** *Front Surg* 2021, **8:**713119.

899. Nadir NA, Kim J, Cassara M, Hrdy M, Zaveri P, Wong AH, Ray J, Strother C, Falk M: **Simulation-based emergency medicine education in the era of physical distancing.** *AEM Educ Train* 2021, **5:**e10586.

900. Nagaraj MB, AbdelFattah KR, Scott DJ, Farr DE: **Creating a Proficiency-based Remote Laparoscopic Skills Curriculum for the COVID-19 Era.** *J Surg Educ* 2022, **79:**229-236.

901. Nagaraj MB, Weis HB, Weis JJ, Cook GS, Bailey LW, Shoultz TH, Farr DE, AbdelFattah KR, Dultz LA: **The Impact of COVID-19 on Surgical Education.** *J Surg Res* 2021, **267:**366-373.

902. Nakashima BJ, Kaur N, Wongjirad C, Inaba K, Sheikh MR: **Telemedicine Experience of General Surgery Trainees: Impact on Patient Care and Education.** *Am Surg* 2022, **88:**2017-2023.

903. Narain TA, Mittal A, Singh V, Panwar VK: **Feasibility and usefulness of online virtual training of urology residents in times of COVID-19: A single-center experience and an evidence-based strength, weakness, opportunity, and threat analysis.** *J Educ Health Promot* 2021, **10:**116.

904. Natalia D, Syakurah RA: **Mental health state in medical students during COVID-19 pandemic.** *J Educ Health Promot* 2021, **10:**208.

905. Newall N, Smith BG, Burton O, Chari A, Kolias AG, Hutchinson PJ, Alamri A, Uff C, Brainbook: **Improving Neurosurgery Education Using Social Media Case-Based Discussions: A Pilot Study.** *World Neurosurg X* 2021, **11:**100103.

906. Nijakowski K, Lehmann A, Zdrojewski J, Nowak M, Surdacka A: **The Effectiveness of the Blended Learning in Conservative Dentistry with Endodontics on the Basis of the Survey among 4th-Year Students during the COVID-19 Pandemic.** *Int J Environ Res Public Health* 2021, **18**.

907. Nijamudeen AM, Brown G, Clark S: **Trialling a novel socially-distanced teaching approach for OMFS dental core trainees: case-based discussions through WhatsApp(c).** *Br J Oral Maxillofac Surg* 2022, **60:**71-73.

908. Nikolis L, Wakim A, Adams W, Do PB: **Medical student wellness in the United States during the COVID-19 pandemic: a nationwide survey.** *BMC Med Educ* 2021, **21:**401.

909. Nodine PM, Arbet J, Jenkins PA, Rosenthal L, Carrington S, Purcell SK, Lee S, Hoon S: **Graduate nursing student stressors during the COVID-19 pandemic.** *J Prof Nurs* 2021, **37:**721-728.

910. Nolte MT, Tornetta P, 3rd, Mehta S, Ponce BA, Grabowski G, Turner NS, Spitnale MJ, Kogan M: **Resident Wellness During the COVID-19 Pandemic: A Nationwide Survey of Orthopaedic Residents.** *J Am Acad Orthop Surg* 2021, **29:**407-413.

911. Nomura O, Irie J, Park Y, Nonogi H, Hanada H: **Evaluating Effectiveness of YouTube Videos for Teaching Medical Students CPR: Solution to Optimizing Clinician Educator Workload during the COVID-19 Pandemic.** *Int J Environ Res Public Health* 2021, **18**.

912. Nurhidayati T, Rahayu DA, Alfiyanti D: **Nursing Students’ Coping for Burnout and Fatigue Online Learning during Coronavirus Disease 2019 Pandemic.** *Open Access Macedonian Journal of Medical Sciences* 2021, **9:**92-96.

913. Odayappan A, Venkatesh R, Tammineni R, Nachiappan S, Iswarya M: **Perspectives of physicians regarding the role of webinars on medical education during the COVID-19 pandemic.** *Indian J Ophthalmol* 2021, **69:**1251-1256.

914. Ojo E, Hairston D: **Recruiting Underrepresented Minority Students into Psychiatry Residency: a Virtual Diversity Initiative.** *Acad Psychiatry* 2021, **45:**440-444.

915. Olmes GL, Zimmermann JSM, Stotz L, Takacs FZ, Hamza A, Radosa MP, Findeklee S, Solomayer EF, Radosa JC: **Students' attitudes toward digital learning during the COVID-19 pandemic: a survey conducted following an online course in gynecology and obstetrics.** *Arch Gynecol Obstet* 2021, **304:**957-963.

916. Omil-Lima D, Fernstrum A, Gupta K, Jella T, Muncey W, Mishra K, Bukavina L, Scarberry K, DeLong J, Nikolavsky D, Gupta S: **Urologic Education in the Era of COVID-19: Results From a Webinar-Based Reconstructive Urology Lecture Series.** *Urology* 2021, **152:**2-8.

917. Ortadeveci A, Ermez MN, Oz S, Ozden H: **A survey study on distance anatomy education: challenges unique to anatomy.** *Surg Radiol Anat* 2022, **44:**41-47.

918. Öztürk Kaygusuz T, Ağalar C, Kurtaran B, Çağ Y, Taşbakan M: **Infectious Diseases and Clinical Microbiology Experts 'Perspectives on Online Education, Web-Based Seminars.** *Mediterranean Journal of Infection Microbes and Antimicrobials* 2021.

919. Panda DS, Gamal M, Zafar A, Parambi DGT, Senapati AK, Patro SK, Sahoo PK, Bose A: **A study on the effectiveness of online teaching in pharmacy education from teacher and student perspectives during the COVID-19 pandemic.** *Pharmacy Education* 2021, **20:**297-301.

920. Pang JH, Finlay E, Fortner S, Pickett B, Wang ML: **Teaching Effective Informed Consent Communication Skills in the Virtual Surgical Clerkship.** *J Am Coll Surg* 2021, **233:**64-72 e62.

921. Patel B, Taggar JS: **Virtual teaching of undergraduate primary care small groups during Covid-19.** *Educ Prim Care* 2021, **32:**296-302.

922. Patel M, Hui J, Ho C, Mak CK, Simpson A, Sockalingam S: **Tutors' Perceptions of the Transition to Video and Simulated Patients in Pre-clinical Psychiatry Training.** *Acad Psychiatry* 2021, **45:**593-597.

923. Patelis N, Bisdas T, Jing Z, Feng J, Trenner M, Tri Nugroho N, Ocke Reis PE, Elkouri S, Lecis A, Karam L, et al: **Vascular e-Learning During the COVID-19 Pandemic: The EL-COVID Survey.** *Ann Vasc Surg* 2021, **77:**63-70.

924. Patra V, Tekulapally K: **Second-year dental students' perception of effectiveness of formative assessment in an online learning environment during COVID-19 pandemic.** *National Journal of Physiology, Pharmacy and Pharmacology* 2021.

925. Pavan: **COVID-19 impact on the mental health of Indian pharmacy students: An online survey.** *International Journal of Pharmaceutical Research* 2021, **13**.

926. Perez-Dominguez F, Polanco-Ilabaca F, Pinto-Toledo F, Michaeli D, Achiardi J, Santana V, Urnelli C, Sawaguchi Y, Rodriguez P, Maldonado M, et al: **Lifestyle Changes Among Medical Students During COVID-19 Pandemic: A Multicenter Study Across Nine Countries.** *Health Educ Behav* 2021, **48:**446-454.

927. Perez-Villalobos C, Ventura-Ventura J, Spormann-Romeri C, Melipillan R, Jara-Reyes C, Paredes-Villarroel X, Rojas-Pino M, Baquedano-Rodriguez M, Castillo-Rabanal I, Parra-Ponce P, et al: **Satisfaction with remote teaching during the first semester of the COVID-19 crisis: Psychometric properties of a scale for health students.** *PLoS One* 2021, **16:**e0250739.

928. Perissotto T, Silva T, Miskulin FPC, Pereira MB, Neves BA, Almeida BC, Casagrande AV, Ribeiz SRI, Nunes PV: **Mental health in medical students during COVID-19 quarantine: a comprehensive analysis across year-classes.** *Clinics (Sao Paulo)* 2021, **76:**e3007.

929. Peterson WJ, Munzer BW, Tucker RV, Losman ED, Harvey C, Hatton C, Sefa N, Bassin BS, Hsu CH: **Rapid Dissemination of a COVID-19 Airway Management Simulation Using a Train-the-Trainers Curriculum.** *Acad Med* 2021, **96:**1414-1418.

930. Peyser A, Gulersen M, Nimaroff M, Mullin C, Goldman RH: **Virtual obstetrics and gynecology fellowship interviews during the coronavirus disease 2019 (COVID-19) pandemic: a survey study.** *BMC Med Educ* 2021, **21:**449.

931. Pham DQ, Golub SA, Breuner CC, Evans YN: **The Impact of Telehealth on Clinical Education in Adolescent Medicine During the COVID-19 Pandemic: Positive Preliminary Findings.** *Front Pediatr* 2021, **9:**642279.

932. Pisani A, Cremers I, Ellul P: **The Impact of the SARS-CoV-2 (COVID-19) Pandemic on Gastroenterology Training.** *GE Port J Gastroenterol* 2021, **92:**1-6.

933. Pivert KA, Boyle SM, Halbach SM, Chan L, Shah HH, Waitzman JS, Mehdi A, Norouzi S, Sozio SM: **Impact of the COVID-19 Pandemic on Nephrology Fellow Training and Well-Being in the United States: A National Survey.** *J Am Soc Nephrol* 2021, **32:**1236-1248.

934. Poelmann FB, Koeter T, Steinkamp PJ, Vriens MR, Verhoeven B, Kruijff S: **The immediate impact of the coronavirus disease 2019 (COVID-19) pandemic on burn-out, work-engagement, and surgical training in the Netherlands.** *Surgery* 2021, **170:**719-726.

935. Pokryszko-Dragan A, Marschollek K, Nowakowska-Kotas M, Aitken G: **What can we learn from the online learning experiences of medical students in Poland during the SARS-CoV-2 pandemic?** *BMC Med Educ* 2021, **21:**450.

936. Pollom EL, Sandhu N, Deig CR, Obeid JP, Miller JA, Kahn JM: **Networking and Applying to Radiation Oncology During A Pandemic: Cross-Sectional Survey of Medical Student Concerns.** *Adv Radiat Oncol* 2021, **6:**100643.

937. Potu BK, Atwa H, Nasr El-Din WA, Othman MA, Sarwani NA, Fatima A, Deifalla A, Fadel RA: **Learning anatomy before and during COVID-19 pandemic: Students' perceptions and exam performance.** *Morphologie* 2022, **106:**188-194.

938. Prezotti JA, Henriques JVT, Favorito LA, Canalini AF, Machado MG, Brandao TBV, Barbosa AMV, Moromizato JKM, Anzolch KMJ, Fernandes RC, et al: **Impact of COVID-19 on education, health and lifestyle behaviour of Brazilian urology residents.** *Int Braz J Urol* 2021, **47:**753-776.

939. Puranachaikere T, Hataiyusuk S, Anupansupsai R, In-Iw S, Saisavoey N, Techapanuwat T, Arunrodpanya F, Charonpongsuntorn C, Wiwattanaworaset P, Siripongpan A, et al: **Stress and associated factors with received and needed support in medical students during COVID-19 pandemic: a multicenter study.** *Korean J Med Educ* 2021, **33:**203-213.

940. Purdy AC, de Virgilio C, Kaji AH, Shields Frey E, Lee-Kong S, Inaba K, Gauvin JM, Neville AL, Donahue TR, Smith BR, et al: **Factors Associated With General Surgery Residents' Operative Experience During the COVID-19 Pandemic.** *JAMA Surg* 2021, **156:**767-774.

941. Qian Q, Yan Y, Xue F, Lin J, Zhang F, Zhao J: **Coronavirus Disease 2019 (COVID-19) Learning Online: A Flipped Classroom Based on Micro-Learning Combined with Case-Based Learning in Undergraduate Medical Students.** *Adv Med Educ Pract* 2021, **12:**835-842.

942. Quaranto BR, Lamb M, Traversone J, Hu J, Lukan J, Cooper C, Schwaitzberg S: **Development of an Interactive Remote Basic Surgical Skills Mini-Curriculum for Medical Students During the COVID-19 Pandemic.** *Surg Innov* 2021, **28:**220-225.

943. Rahm AK, Tollner M, Hubert MO, Klein K, Wehling C, Sauer T, Hennemann HM, Hein S, Kender Z, Gunther J, et al: **Effects of realistic e-learning cases on students' learning motivation during COVID-19.** *PLoS One* 2021, **16:**e0249425.

944. Rana R, Kumawat D, Sahay P, Gour N, Patel S, Samanta R, Singh A, Mittal S: **Perception among ophthalmologists about webinars as a method of continued medical education during COVID-19 pandemic.** *Indian J Ophthalmol* 2021, **69:**951-957.

945. Rath A, Wong MLZ, Pannuti CM: **Blackboard-based online assessment during COVID-19 era: A new leaf to an old tree.** *J Dent Educ* 2022, **86 Suppl 1:**804-807.

946. Rathod V, Agrawal T, Sahu D: **Will webinars prove to be an effective teaching medium post-pandemic?** *Postgrad Med J* 2022, **98:**e142-e143.

947. Ravat S, Barnard-Ashton P, Keller MM: **Blended teaching versus traditional teaching for undergraduate physiotherapy students at the University of the Witwatersrand.** *S Afr J Physiother* 2021, **77:**1544.

948. Redinger KE, Greene JD: **Virtual Emergency Medicine Clerkship Curriculum during the COVID-19 Pandemic: Development, Application, and Outcomes.** *West J Emerg Med* 2021, **22:**792-798.

949. Reece S, Johnson M, Simard K, Mundell A, Terpstra N, Cronin T, Dube M, Kaba A, Grant V: **Use of Virtually Facilitated Simulation to Improve COVID-19 Preparedness in Rural and Remote Canada.** *Clin Simul Nurs* 2021, **57:**3-13.

950. Ren R, Parekh K, Franzen D, Estes M, Camejo M, Olaf M, Zhang XC: **Emergency medicine clerkship director experience adapting emergency remote learning during the onset of COVID-19 pandemic.** *AEM Educ Train* 2021, **5:**e10594.

951. Repajic M, Anderson RC, Hu P, Allgood E, Reddy S: **Virtual "Radiology Rounds": Resident-run Medical Education in the COVID-19 Era.** *Acad Radiol* 2021, **28:**1477-1478.

952. Rimsha S, Moosa FA, Zaheer F, Kamal MT, Majid A: **What Does the Future Hold for a Surgical Trainee? This Lockdown Is Not a Letdown Yet: A Survey on Moodle Learning Management System as a Part of Blended Learning During COVID-19 Pandemic.** *Cureus* 2021, **13:**e16690.

953. Rivet EB, Cholyway R, Edwards C, Wishnoff M, Raza O, Haynes S, Feldman M: **Video-mediated breaking bad news simulation.** *Clin Teach* 2021, **18:**424-430.

954. Rydel TA, Bajra R, Schillinger E: **Hands Off Yet All In: A Virtual Clerkship Pilot in the Ambulatory Setting During the COVID-19 Pandemic.** *Acad Med* 2021, **96:**1702-1705.

955. Sabir: **Experience of E. learning among the students of KMSMC Sialkot conducted during COVID-19 pandemic.** *Pakistan Journal of Medical and Health Sciences*.

956. Sadid-Zadeh R, Li R, Arany H, Deluca J: **Students' perception of 3D video microscope use in preclinical fixed prosthodontics: A comparative study.** *J Dent Educ* 2021, **85:**1765-1772.

957. Saeed N, Javed N: **Lessons from the COVID-19 pandemic: Perspectives of medical students.** *Pak J Med Sci* 2021, **37:**1402-1407.

958. Sahin B, Hanalioglu S: **The Continuing Impact of Coronavirus Disease 2019 on Neurosurgical Training at the 1-Year Mark: Results of a Nationwide Survey of Neurosurgery Residents in Turkey.** *World Neurosurg* 2021, **151:**e857-e870.

959. Said: **Knowledge and practices of nurses regarding corona virus (COVID-19): An educational intervention.** *Medico-Legal Update*.

960. Saini K, Conway-Jones R, Jurdon R, Penfold R, Celi LA, Alser O: **Distance-learning collaborations for rapid knowledge sharing to the occupied Palestinian territory during the COVID-19 response: experience from the OxPal partnership.** *Med Confl Surviv* 2021, **37:**55-68.

961. Saini: **A Cross Sectional Study “Impact of Corona Virus Pandemic on Nursing Education: A Paradigm Shifting or Sustaining the Roots?** *Indian Journal of Forensic Medicine & Toxicology* 2021.

962. Saini P, Debroy K, Badger C, Powell C, Thakkar P, Chretien KC: **Virtual Shadowing Program for Preclinical Medical Students.** *Med Sci Educ* 2021, **31:**1575-1580.

963. Salehi PP, Torabi SJ, Lee YH, Azizzadeh B: **Effects of COVID-19 on Facial Plastic and Reconstructive Surgery Fellowship Training and Director Practices.** *OTO Open* 2021, **5:**2473974X211014130.

964. Sanseau E, Lavoie M, Tay KY, Good G, Tsao S, Burns R, Thomas A, Heckle T, Wilson M, Kou M, Auerbach M: **TeleSimBox: A perceived effective alternative for experiential learning for medical student education with social distancing requirements.** *AEM Educ Train* 2021, **5:**e10590.

965. Santos TM, Pedrosa R, Carvalho D, Franco MH, Silva JLG, Franci D, de Jorge B, Munhoz D, Calderan T, Grangeia TAG, Cecilio-Fernandes D: **Implementing healthcare professionals' training during COVID-19: a pre and post-test design for simulation training.** *Sao Paulo Med J* 2021, **139:**514-519.

966. Savage DJ, Gutierrez O, Montane BE, Singh AD, Yudelevich E, Mahar J, Brateanu A, Khatri L, Fleisher C, Jolly SE: **Implementing a telemedicine curriculum for internal medicine residents during a pandemic: the Cleveland Clinic experience.** *Postgrad Med J* 2022, **98:**487-491.

967. Scherg A, Ilse B, Elsner F: **[Undergraduate palliative care teaching in times of COVID-19].** *Schmerz* 2021, **35:**237-241.

968. Schindler AK, Polujanski S, Rotthoff T: **A longitudinal investigation of mental health, perceived learning environment and burdens in a cohort of first-year German medical students' before and during the COVID-19 'new normal'.** *BMC Med Educ* 2021, **21:**413.

969. Schmitz SM, Schipper S, Lemos M, Alizai PH, Kokott E, Brozat JF, Neumann UP, Ulmer TF: **Development of a tailor-made surgical online learning platform, ensuring surgical education in times of the COVID19 pandemic.** *BMC Surg* 2021, **21:**196.

970. Schuler MS, Tyo MB, Barnett K: **Nursing student perceptions of required online educational programs utilized outside the classroom.** *Nurse Educ Today* 2021, **105:**105048.

971. Scoular S, Huntsberry A, Patel T, Wettergreen S, Brunner JM: **Transitioning Competency-Based Communication Assessments to the Online Platform: Examples and Student Outcomes.** *Pharmacy (Basel)* 2021, **9**.

972. Sebbani M, Adarmouch L, Mansouri A, Mansoury O, Michaud S, ElAdib AR, Bouskraoui M, Amine M, Warnars HLHS: **Implementation of Online Teaching in Medical Education: Lessons Learned from Students’ Perspectives during the Health Crisis in Marrakesh, Morocco.** *Education Research International* 2021, **2021:**1-9.

973. Sehgal S, Shinde L, Madheswaran G, Mukherjee P, Verkicharla P, Easwaran S, Bandamwar K: **Impact of COVID-19 on Indian optometrists: A student, educator, and practitioner's perspective.** *Indian J Ophthalmol* 2021, **69:**958-963.

974. Sepp K, Volmer D: **Use of Face-to-Face Assessment Methods in E-Learning—An Example of an Objective Structured Clinical Examination (OSCE) Test.** *Pharmacy* 2021, **9**.

975. Setiawan E, Sugeng B, Luailiyah A, Makarim FR, Trisnadi S: **Evaluating knowledge and skill in surgery clerkship during covid 19 pandemics: A single-center experience in Indonesia.** *Ann Med Surg (Lond)* 2021, **68:**102685.

976. Abdel Shafi AM, Sheikh AM, Awad WI: **Comparison of cardiothoracic surgical training before and during the COVID-19 pandemic in the United Kingdom.** *JTCVS Open* 2021, **7:**394-410.

977. Shaiba LA, Alnamnakani MA, Temsah MH, Alamro N, Alsohime F, Alrabiaah A, Alanazi SN, Alhasan K, Alherbish A, Mobaireek KF, et al: **Medical Faculty's and Students' Perceptions toward Pediatric Electronic OSCE during the COVID-19 Pandemic in Saudi Arabia.** *Healthcare (Basel)* 2021, **9**.

978. Sharma N, Bhusal CK, Subedi S, Kasarla RR: **Perception towards Online Classes during COVID-19 among MBBS and BDS Students in a Medical College of Nepal: A Descriptive Cross-sectional Study.** *JNMA J Nepal Med Assoc* 2021, **59:**276-279.

979. Sharma R, King TS, Hanson ER, Fiebelkorn K: **Medical Histopathology Laboratories: Remote Teaching in Response to COVID-19 Pandemic.** *Acad Pathol* 2021, **8:**2374289521998049.

980. Sharma R, Mohanty A, Singh V, S VA, Gupta PK, Jelly P, Gupta P, Rao S: **Effectiveness of Video-Based Online Training for Health Care Workers to Prevent COVID-19 Infection: An Experience at a Tertiary Care Level Institute, Uttarakhand, India.** *Cureus* 2021, **13:**e14785.

981. Shrivastava KJ, Nahar R, Parlani S, Murthy VJ: **A cross-sectional virtual survey to evaluate the outcome of online dental education system among undergraduate dental students across India amid COVID-19 pandemic.** *Eur J Dent Educ* 2022, **26:**123-130.

982. Syofyan S, Permatasari D, Hasanah U, Armin F, Yosmar R, Sri Wahyuni F, Lailaturrahmi L: **Student and faculty perceptions related to online learning during the COVID-19 pandemic in Indonesia.** *Pharmacy Education* 2020, **20:**302-309.

983. Szigiato AA, Palakkamanil M, Aubin MJ, Ziai S: **Canadian ophthalmology resident experience during the COVID-19 pandemic.** *Can J Ophthalmol* 2021, **56:**e42-e44.

984. Nathaniel TI, Goodwin RL, Fowler L, McPhail B, Black AC, Jr.: **An Adaptive Blended Learning Model for the Implementation of an Integrated Medical Neuroscience Course During the Covid-19 Pandemic.** *Anat Sci Educ* 2021, **14:**699-710.

985. Takahashi K, Tanaka C, Numaguchi R, Kuroda Y, Nemoto H, Yoshino K, Noda M, Inoue Y, Wada K, Committee of The Japanese Society for Cardiovascular Surgery Under F: **Remote simulator training of coronary artery bypass grafting during the coronavirus disease 2019 pandemic.** *JTCVS Open* 2021, **8:**524-533.

986. Talapko J, Peric I, Vulic P, Pustijanac E, Jukic M, Bekic S, Mestrovic T, Skrlec I: **Mental Health and Physical Activity in Health-Related University Students during the COVID-19 Pandemic.** *Healthcare (Basel)* 2021, **9**.

987. Tan LT, Tanderup K, Nappa A, Petric P, Jurgenliemk-Schulz IM, Serban M, Swamidas JV, Palmu M, Duke SL, Mahantshetty U, et al: **Impact of transitioning to an online course - A report from the ESTRO gyn teaching course.** *Clin Transl Radiat Oncol* 2021, **29:**85-92.

988. Tang ACY, Kwong EW, Chen L, Cheng WLS: **Associations between demographic characteristics, perceived threat, perceived stress, coping responses and adherence to COVID-19 prevention measures among Chinese healthcare students.** *J Adv Nurs* 2021, **77:**3759-3771.

989. Taylor M, Wallen T, Mehaffey JH, Shirafkan A, Brescia AA, Freeman K, Louis C, Watson J, Okereke I: **Interviews During the Pandemic: A Thoracic Education Cooperative Group and Surgery Residents Project.** *Ann Thorac Surg* 2022, **113:**663-668.

990. Teixeira BL, Cabral J, Mendes G, Madanelo M, Rocha MA, Mesquita S, Correia J, Tavares C, Marques-Pinto A, Fraga A: **How the COVID-19 pandemic changed urology residency - a nationwide survey from the Portuguese resident's perspective.** *Cent European J Urol* 2021, **74:**121-127.

991. Tellez J, Abdelfattah K, Farr D: **In-person versus virtual suturing and knot-tying curricula: Skills training during the COVID-19 era.** *Surgery* 2021, **170:**1665-1669.

992. Tempski P, Arantes-Costa FM, Kobayasi R, Siqueira MAM, Torsani MB, Amaro B, Nascimento M, Siqueira SL, Santos IS, Martins MA: **Medical students' perceptions and motivations during the COVID-19 pandemic.** *PLoS One* 2021, **16:**e0248627.

993. Temsah MH, Alkhattabi F, Aljamaan F, Alhasan K, Alherbish A, Philby M, Alsohime F, Alobaylan M, Alabdulkarim H, Almosned B, et al: **Remote interviews for medical residency selection during the initial COVID-19 crisis: a national survey.** *BMC Med Educ* 2021, **21:**462.

994. Thapa P, Bhandari SL, Pathak S: **Nursing students' attitude on the practice of e-learning: A cross-sectional survey amid COVID-19 in Nepal.** *PLoS One* 2021, **16:**e0253651.

995. Thind AS, Singh H, Yerramsetty DL, Pandeya DR: **Impact of the COVID-19 pandemic on Caribbean Medical Students: A cross-sectional study.** *Ann Med Surg (Lond)* 2021, **67:**102515.

996. Thom ML, Kimble BA, Qua K, Wish-Baratz S: **Is remote near-peer anatomy teaching an effective teaching strategy? Lessons learned from the transition to online learning during the Covid-19 pandemic.** *Anat Sci Educ* 2021, **14:**552-561.

997. Truong A, Amersi F, Chau V, Imai T: **The Fellow's Perspective: The Impact of the COVID-19 Pandemic on Fellowship Training and Job Appointment.** *Am Surg* 2021, **87:**1678-1683.

998. Trzcionka A, Zalewska I, Tanasiewicz M: **Interpretative Phenomenological Analysis for the Comparison of Polish and Foreign Dentistry Students' Concerns during the Covid-19 Pandemic.** *Healthcare (Basel)* 2021, **9**.

999. Tsyrulnik A, Gottlieb M, Coughlin RF, Bod J, Della-Giustina D, Greenberger S, von Reinhart A, Heinrich S, Rotoli J, Goldflam K: **Socially distanced, virtually connected: Faculty and resident perceptions of virtual didactics.** *AEM Educ Train* 2021, **5:**e10617.

1000. Tuma F, Nituica C, Mansuri O, Kamel MK, McKenna J, Blebea J: **The academic experience in distance (virtual) rounding and education of emergency surgery during COVID-19 pandemic.** *Surg Open Sci* 2021, **5:**6-9.

1001. Turkles S, Bogahan M, Altundal H, Yaman Z, Yilmaz M: **Diaries of Nursing Students during the COVID-19 Pandemic: A Qualitative Descriptive Study.** *Int J Environ Res Public Health* 2021, **18**.

1002. Tzerefos C, Meling TR, Lafuente J, Fountas KN, Brotis AG, Demetriades AK: **The Impact of the Coronavirus Pandemic on European Neurosurgery Trainees.** *World Neurosurg* 2021, **154:**e283-e291.

1003. Unrue EL, White G, Cheng N, Lindsey T: **Effect of a standardized patient encounter on first year medical student confidence and satisfaction with telemedicine.** *J Osteopath Med* 2021, **121:**733-737.

1004. Upadhyay N, Wadkin JCR: **Can training in diagnostic radiology be moved online during the COVID-19 pandemic? UK trainee perceptions of the Radiology-Integrated Training Initiative (R-ITI) e-learning platform.** *Clin Radiol* 2021, **76:**854-860.

1005. Weber AM, Dua A, Chang K, Jupalli H, Rizwan F, Chouthai A, Chen C: **An outpatient telehealth elective for displaced clinical learners during the COVID-19 pandemic.** *BMC Med Educ* 2021, **21:**174.

1006. Weber C, Ntasumbumuyange D, Ngoga E, Bazzett-Matabele L, Francis J, Paley P, Graef K, Ghebre R: **Continuing medical education during COVID-19: virtual training for gynecologic oncology management in Rwanda.** *Int J Gynecol Cancer* 2021, **31:**1184-1185.

1007. Weld JK, Frank LH, Gandhi R: **Pediatric cardiology national education series: A remote education response to COVID-19.** *Progress in Pediatric Cardiology* 2021, **61**.

1008. Weygandt PL, Jordan J, Caretta-Weyer H, Osborne A, Grabow Moore K: **Impact of the COVID-19 pandemic on emergency medicine education: Insights from faculty and residents.** *AEM Educ Train* 2021, **5:**e10603.

1009. White MJ, Birkness JE, Salimian KJ, Meiss AE, Butcher M, Davis K, Ware AD, Zarella MD, Lecksell K, Rooper LM, et al: **Continuing Undergraduate Pathology Medical Education in the Coronavirus Disease 2019 (COVID-19) Global Pandemic: The Johns Hopkins Virtual Surgical Pathology Clinical Elective.** *Arch Pathol Lab Med* 2021, **145:**814-820.

1010. Whited TM, Stickley K, De Gravelles P, Steele T, English B: **Using Telehealth to Enhance Pediatric Psychiatric Clinical Simulation: Rising to Meet the COVID-19 Challenge.** *Online Learning* 2021, **25**.

1011. Wickramasinghe ND, Jayarathne SW, Pilapitiya SD: **Value-Added Roles of Medical Students During the COVID-19 Pandemic: Assessment of Medical Students' Perceptions and Willingness in Sri Lanka.** *Int J Gen Med* 2021, **14:**3187-3196.

1012. Wiedenmann C, Wacker K, Bohringer D, Maier P, Reinhard T: **[Online examination course instead of classroom teaching: adaptation of medical student teaching during the COVID-19 pandemic].** *Ophthalmologe* 2022, **119:**11-18.

1013. Wietlisbach LE, Asch DA, Eriksen W, Barg FK, Bellini LM, Desai SV, Yakubu AR, Shea JA: **Dark Clouds With Silver Linings: Resident Anxieties About COVID-19 Coupled With Program Innovations and Increased Resident Well-Being.** *J Grad Med Educ* 2021, **13:**515-525.

1014. Wilson JL, Hensley A, Culp-Roche A, Hampton D, Hardin-Fanning F, Thaxton-Wiggins A: **Transitioning to Teaching Online During the COVID-19 Pandemic.** *SAGE Open Nurs* 2021, **7:**23779608211026137.

1015. Winn AS, Weaver MD, O'Donnell KA, Sullivan JP, Robbins R, Landrigan CP, Barger LK: **Interns' perspectives on impacts of the COVID-19 pandemic on the medical school to residency transition.** *BMC Med Educ* 2021, **21:**330.

1016. Wise CE, Bereknyei Merrell S, Sasnal M, Forrester JD, Hawn MT, Lau JN, Lin DT, Schmiederer IS, Spain DA, Nassar AK, Knowlton LM: **COVID-19 Impact on Surgical Resident Education and Coping.** *J Surg Res* 2021, **264:**534-543.

1017. Wittenberg E, Goldsmith JV, Chen C, Prince-Paul M, Capper B: **COVID 19-transformed nursing education and communication competency: Testing COMFORT educational resources.** *Nurse Educ Today* 2021, **107:**105105.

1018. Liu J, Zhu Q, Fan W, Makamure J, Zheng C, Wang J: **Online Mental Health Survey in a Medical College in China During the COVID-19 Outbreak.** *Front Psychiatry* 2020, **11:**459.

1019. Liu Q, Sun W, Du C, Yang L, Yuan N, Cui H, Song W, Ge L: **Medical Morphology Training Using the Xuexi Tong Platform During the COVID-19 Pandemic: Development and Validation of a Web-Based Teaching Approach.** *JMIR Med Inform* 2021, **9:**e24497.

1020. Lo HY, Lin SC, Chaou CH, Chang YC, Ng CJ, Chen SY: **What is the impact of the COVID-19 pandemic on emergency medicine residency training: an observational study.** *BMC Med Educ* 2020, **20:**348.

1021. Loch C, Kuan IBJ, Elsalem L, Schwass D, Brunton PA, Jum'ah A: **COVID-19 and dental clinical practice: Students and clinical staff perceptions of health risks and educational impact.** *J Dent Educ* 2021, **85:**44-52.

1022. Loda T, Loffler T, Erschens R, Zipfel S, Herrmann-Werner A: **Medical education in times of COVID-19: German students' expectations - A cross-sectional study.** *PLoS One* 2020, **15:**e0241660.

1023. Longhurst GJ, Stone DM, Dulohery K, Scully D, Campbell T, Smith CF: **Strength, Weakness, Opportunity, Threat (SWOT) Analysis of the Adaptations to Anatomical Education in the United Kingdom and Republic of Ireland in Response to the Covid-19 Pandemic.** *Anat Sci Educ* 2020, **13:**301-311.

1024. LoSavio PS, Eggerstedt M, Tajudeen BA, Papagiannopoulos P, Revenaugh PC, Batra PS, Husain I: **Rapid implementation of COVID-19 tracheostomy simulation training to increase surgeon safety and confidence.** *Am J Otolaryngol* 2020, **41:**102574.

1025. Lyons Z, Wilcox H, Leung L, Dearsley O: **COVID-19 and the mental well-being of Australian medical students: impact, concerns and coping strategies used.** *Australas Psychiatry* 2020, **28:**649-652.

1026. MacDonald CW, Lonnemann E, Petersen SM, Rivett DA, Osmotherly PG, Brismee JM: **COVID 19 and manual therapy: international lessons and perspectives on current and future clinical practice and education.** *J Man Manip Ther* 2020, **28:**134-145.

1027. Maeda Y, Takeda K, Hayama M, Tsuda T, Shikina T, Nishiike S, Kawashima K, Inohara H: **Experience with online lectures about endoscopic sinus surgery using a video conferencing app.** *Auris Nasus Larynx* 2020, **47:**1083-1085.

1028. Sophia M: **Knowledge, Anxiety level and Perceptions on Prevention Protocol of COVID19 among Medical and Dental Graduates.** *Indian Journal of Public Health Research & Development* 2020.

1029. Sophia M: **Perception of Undergraduate Medical and Dental Students Towards Learning Anatomy in Google Classroom.** *Indian Journal of Public Health Research & Development* 2020.

1030. Majumder A, Eckhouse SR, Brunt LM, Awad MM, Dimou FM, Eagon JC, Holden S, Fone H, Blatnik JA: **Initial Experience with a Virtual Platform for Advanced Gastrointestinal Minimally Invasive Surgery Fellowship Interviews.** *J Am Coll Surg* 2020, **231:**670-678.

1031. Maksymenko: **Features of the advanced vocational training of medical workers under modern conditions.** *Systematic Reviews in Pharmacy*.

1032. Mallon D, Pohl JF, Phatak UP, Fernandes M, Rosen JM, Lusman SS, Nylund CM, Jump CS, Solomon AB, Srinath A, et al: **Impact of COVID-19 on Pediatric Gastroenterology Fellow Training in North America.** *J Pediatr Gastroenterol Nutr* 2020, **71:**6-11.

1033. Manalo TA, Higgins MI, Pettitt-Schieber B, Pettitt BJ, Mehta A, Hartsell LM: **A Strategy for Undergraduate Medical Education in Urology During the COVID-19 Pandemic.** *J Surg Educ* 2021, **78:**746-750.

1034. Manjareeka: **COVID-19 lockdown anxieties: Is student a vulnerable group?** *Journal of Indian Association for Child and Adolescent Mental Health*.

1035. Marasco G, Nardone OM, Maida M, Boskoski I, Pastorelli L, Scaldaferri F, Italian Association of Young G, Endoscopist: **Impact of COVID-19 outbreak on clinical practice and training of young gastroenterologists: A European survey.** *Dig Liver Dis* 2020, **52:**1396-1402.

1036. Megaloikonomos PD, Thaler M, Igoumenou VG, Bonanzinga T, Ostojic M, Couto AF, Diallo J, Khosravi I: **Impact of the COVID-19 pandemic on orthopaedic and trauma surgery training in Europe.** *Int Orthop* 2020, **44:**1611-1619.

1037. Pawlak KM, Kral J, Khan R, Amin S, Bilal M, Lui RN, Sandhu DS, Hashim A, Bollipo S, Charabaty A, et al: **Impact of COVID-19 on endoscopy trainees: an international survey.** *Gastrointest Endosc* 2020, **92:**925-935.

1038. Pertile D, Gallo G, Barra F, Pasculli A, Batistotti P, Sparavigna M, Vizzielli G, Soriero D, Graziano G, Di Saverio S, et al: **The impact of COVID-19 pandemic on surgical residency programmes in Italy: a nationwide analysis on behalf of the Italian Polyspecialistic Young Surgeons Society (SPIGC).** *Updates Surg* 2020, **72:**269-280.

1039. Pilar A, Gravel SB, Croke J, Soliman H, Chung P, Wong RKS: **Coronavirus Disease 2019's (COVID-19's) Silver Lining-Through the Eyes of Radiation Oncology Fellows.** *Adv Radiat Oncol* 2021, **6:**100527.

1040. Rajhans V, Memon U, Patil V, Goyal A: **Impact of COVID-19 on academic activities and way forward in Indian Optometry.** *J Optom* 2020, **13:**216-226.

1041. Rajwa P, Przydacz M, Zapala P, Wieckiewicz G, Ryszawy J, Choragwicki D, Drobot RB, Radziszewski P, Paradysz A, Chlosta PL: **How has the COVID-19 pandemic impacted Polish urologists? Results from a national survey.** *Cent European J Urol* 2020, **73:**252-259.

1042. Rakhmanov: **Knowledge and Anxiety Levels of African University Students Against COVID-19 During the Pandemic Outbreak by an Online Survey.** *Journal of Research in Medical and Dental Science*.

1043. Ramos-Morcillo AJ, Leal-Costa C, Moral-Garcia JE, Ruzafa-Martinez M: **Experiences of Nursing Students during the Abrupt Change from Face-to-Face to e-Learning Education during the First Month of Confinement Due to COVID-19 in Spain.** *Int J Environ Res Public Health* 2020, **17**.

1044. Rana T, Hackett C, Quezada T, Chaturvedi A, Bakalov V, Leonardo J, Rana S: **Medicine and surgery residents' perspectives on the impact of COVID-19 on graduate medical education.** *Med Educ Online* 2020, **25:**1818439.

1045. Rao P, Diamond J, Korjian S, Martin L, Varghese M, Serfas JD, Lee R, Fraiche A, Kannam J, Reza N, American College of Cardiology Fellows-in-Training Section Leadership C: **The Impact of the COVID-19 Pandemic on Cardiovascular Fellows-in-Training: A National Survey.** *J Am Coll Cardiol* 2020, **76:**871-875.

1046. Rashid A, Faisal K: **Pandemic anxiety and its correlates among young doctors working frontline in Pakistan.** *Glob Ment Health (Camb)* 2020, **7:**e27.

1047. Rasouli JJ, Shin JH, Than KD, Gibbs WN, Baum GR, Baaj AA: **Virtual Spine: A Novel, International Teleconferencing Program Developed to Increase the Accessibility of Spine Education During the COVID-19 Pandemic.** *World Neurosurg* 2020, **140:**e367-e372.

1048. Reverte-Villarroya S, Ortega L, Lavedan A, Masot O, Burjales-Marti MD, Ballester-Ferrando D, Fuentes-Pumarola C, Botigue T: **The influence of COVID-19 on the mental health of final-year nursing students: comparing the situation before and during the pandemic.** *Int J Ment Health Nurs* 2021, **30:**694-702.

1049. Robbins JB, England E, Patel MD, DeBenedectis CM, Sarkany DS, Heitkamp DE, Milburn JM, Kalia V, Ali K, Gaviola GC, et al: **COVID-19 Impact on Well-Being and Education in Radiology Residencies: A Survey of the Association of Program Directors in Radiology.** *Acad Radiol* 2020, **27:**1162-1172.

1050. Robinson K, Tang HY, Metzenberg E, Peterson J, Umoren R, Sawyer T: **Socially Distanced Neonatal Resuscitation Program (NRP): A Technical Report on How to Teach NRP Courses During the COVID-19 Pandemic.** *Cureus* 2020, **12:**e10959.

1051. Robinson KA, Shin B, Gangadharan SP: **A Comparison Between In-Person and Virtual Fellowship Interviews During the COVID-19 Pandemic.** *J Surg Educ* 2021, **78:**1175-1181.

1052. Roy H, Ray K, Bhakta A: **Faculties Perception on Anatomy Teaching and Assessment in Lockdown and Post-lockdown New Normal Phase.** *Journal of Clinical and Diagnostic Research* 2020.

1053. Roy H, Ray K, Saha S, Ghosal AK: **A Study on Students’ Perceptions for Online Zoom-app based Flipped Class Sessions on Anatomy Organised during the Lockdown Period of COVID-19 Epoch.** *Journal of Clinical and Diagnostic Research* 2020.

1054. Roy V, Arora J, Kaur N, Gandhi A: **Effect of the present situation of coronavirus disease-2019 pandemic on the academic and personal life of undergraduate medical and paramedical students.** *National Journal of Physiology, Pharmacy and Pharmacology* 2020.

1055. Singh HK, Joshi A, Malepati RN, Najeeb S, Balakrishna P, Pannerselvam NK, Singh YK, Ganne P: **A survey of E-learning methods in nursing and medical education during COVID-19 pandemic in India.** *Nurse Educ Today* 2021, **99:**104796.

1056. Singh K, Srivastav S, Bhardwaj A, Dixit A, Misra S: **Medical Education During the COVID-19 Pandemic: A Single Institution Experience.** *Indian Pediatr* 2020, **57:**678-679.

1057. Srivastava V, Pandey V, Tiwari P, Patel S, Ansari MA, Shukla VK: **Utility of Real-Time Online Teaching During COVID Era Among Surgery Postgraduates.** *Indian J Surg* 2020, **82:**762-768.

1058. Steele TN, Hemal K, Browne DT, Balumuka D, Hansen JE, David LR: **Academics in the Pandemic: Early Impact of COVID-19 on Plastic Surgery Training Programs.** *Plast Reconstr Surg Glob Open* 2020, **8:**e3320.

1059. Suarez-Cedeno G, Pantelyat A, Mills KA, Murthy M, Alshaikh JT, Rosenthal LS, Bang J, Moukheiber E: **Movement Disorders Virtual Fellowship Training in Times of Coronavirus Disease 2019: A Single-Center Experience.** *Telemed J E Health* 2021, **27:**1160-1165.

1060. Sun Y, Wang D, Han Z, Gao J, Zhu S, Zhang H: **Disease Prevention Knowledge, Anxiety, and Professional Identity during COVID-19 Pandemic in Nursing Students in Zhengzhou, China.** *J Korean Acad Nurs* 2020, **50:**533-540.

1061. Traba C, Holland B, Laboy MC, Lamba S, Chen S: **A Multi-Modal Remote Clinical Skills Mini-Course Utilizing a Teaching TeleOSCE.** *Med Sci Educ* 2021, **31:**503-509.

1062. Trembley LL, Tobias AZ, Schillo G, von Foerster N, Singer J, Pavelka SL, Phrampus P: **A Multidisciplinary Intubation Algorithm for Suspected COVID-19 Patients in the Emergency Department.** *West J Emerg Med* 2020, **21:**764-770.

1063. Ulenaers D, Grosemans J, Schrooten W, Bergs J: **Clinical placement experience of nursing students during the COVID-19 pandemic: A cross-sectional study.** *Nurse Educ Today* 2021, **99:**104746.

1064. Vallee M, Kutchukian S, Pradere B, Verdier E, Durbant E, Ramlugun D, Weizman I, Kassir R, Cayeux A, Pecheux O, et al: **Prospective and observational study of COVID-19's impact on mental health and training of young surgeons in France.** *Br J Surg* 2020, **107:**e486-e488.

1065. Veerasuri S, Vekeria M, Davies SE, Graham R, Rodrigues JCL: **Impact of COVID-19 on UK radiology training: a questionnaire study.** *Clin Radiol* 2020, **75:**877 e877-877 e814.

1066. Villanueva EW, Meissner H, Walters RW: **Medical Student Perceptions of the Learning Environment, Quality of Life, and the School of Medicine's Response to the COVID-19 Pandemic: A Single Institution Perspective.** *Med Sci Educ* 2021, **31:**589-598.

1067. Vitale E, Moretti B, Noternicola A, Covelli I: **How the Italian Nursing students deal the pandemic Covid-19 condition.** *Acta Biomed* 2020, **91:**e2020007.

1068. Wang C, Xie A, Wang W, Wu H: **Association between medical students' prior experiences and perceptions of formal online education developed in response to COVID-19: a cross-sectional study in China.** *BMJ Open* 2020, **10:**e041886.

1069. Weston J, Zauche LH: **Comparison of Virtual Simulation to Clinical Practice for Prelicensure Nursing Students in Pediatrics.** *Nurse Educ* 2021, **46:**E95-E98.

1070. Whitby T, Cleary G, Halfhide CP: **Impact of COVID-19 on training: a single-centre survey of trainees.** *BMJ Paediatr Open* 2020, **4:**e000757.

1071. Willey JM, Olvet DM, Bird JB, Brenner JM: **Pandemics Past and Present: A Guided Inquiry Approach.** *J Med Educ Curric Dev* 2020, **7:**2382120520976957.

1072. Wlodarczyk JR, Alicuben ET, Hawley L, Sullivan M, Ault GT, Inaba K: **Development and emergency implementation of an online surgical education curriculum for a General Surgery program during a global pandemic: The University of Southern California experience.** *Am J Surg* 2021, **221:**962-972.

1073. Wu S, Li Z, Li Z, Xiang W, Yuan Y, Liu Y, Xiong Z: **The mental state and risk factors of Chinese medical staff and medical students in early stages of the COVID-19 epidemic.** *Compr Psychiatry* 2020, **102:**152202.

1074. Yoo H, Kim D, Lee YM, Rhyu IJ: **Adaptations in Anatomy Education during COVID-19.** *J Korean Med Sci* 2021, **36:**e13.

1075. Boros M, Sventekova E, Cidlinova A, Bardy M, Batrlova K: **Application of VR Technology to the Training of Paramedics.** *Applied Sciences* 2022, **12**.

1076. Bosco A, Tay HW, Aleem I, Citak M, Uvaraj NR, Park JB, Matsumoto M, Marin-Penna O, Buvanesh J, Khan M, Hey HWD: **Challenges to the orthopedic resident workforce during the first wave of COVID-19 pandemic: Lessons learnt from a global cross-sectional survey.** *J Orthop* 2021, **27:**103-113.

1077. Botha RC JHL, A. Vermeulen, V. Forgan, T. R. Al-Benna, S. Chu, K. M.: **Surgical training during the COVID-19 pandemic - a single institution's trainee survey.** *S Afr J Surg*, **60:**40-43.

1078. Bouaoud J, Michon L, Saintigny P: **Teaching how to break bad news in Oncology: In-class vs. virtual peer role-plays.** *Bull Cancer* 2022, **109:**685-691.

1079. Bouiller K, Peiffer-Smadja N, Cevik M, Last K, Antunovic IA, Sterbenc A, Lopes MJ, Barac A, Schweitzer V, Delliere S: **Role and perception of clinical microbiology and infectious diseases trainees during the COVID-19 crisis.** *Future Microbiol* 2022, **17:**411-416.

1080. Boulos AN: **Evaluation of the effectiveness of online education in anatomy for medical students during the COVID-19 pandemic.** *Ann Anat* 2022, **244:**151973.

1081. Bourne T, Kyriacou C, Shah H, Ceusters J, Preisler J, Metzger U, Landolfo C, Lees C, Timmerman D: **Experiences and well-being of healthcare professionals working in the field of ultrasound in obstetrics and gynaecology as the SARS-CoV-2 pandemic were evolving: a cross-sectional survey study.** *BMJ Open* 2022, **12:**e051700.

1082. Bowers RD, Young LB, Comnick CL, Kasundra HP, Barwacz CA: **Perceived Didactic Curricular Effectiveness of In-Person vs. Virtual Formats amongst Fourth-Year Dental Students.** *Dent J (Basel)* 2022, **10**.

1083. Bowser AS, Kazakoff MA, Scott PW, Dunbar-Jacob J: **Nursing Students' Dissatisfaction With Course Organization and Student Engagement in Remote Learning 1 Year Post-COVID-19 Restrictions.** *Nurse Educ* 2022, **47:**E68-E72.

1084. Brandao CFS, Vaccarezza GF, Albanese RP, Fernandes G, Cecilio-Fernandes D: **Application of telesimulation in a medical undergraduate course during the SARS-CoV-2 pandemic: a quantitative and retrospective study.** *Sao Paulo Med J* 2022, **140:**509-513.

1085. Brazier A, Larson E, Xu Y, Judah G, Egan M, Burd H, Darzi A: **'Dear Doctor': a randomised controlled trial of a text message intervention to reduce burnout in trainee anaesthetists.** *Anaesthesia* 2022, **77:**405-415.

1086. Carpenter EL, Adams AM, Chick RC, Stull MC, Hale DF, Propper BW, Clifton GT, Vreeland TJ: **Maximizing Benefit of Virtual Learning: Lessons From the Coronavirus Disease 2019 Pandemic.** *J Surg Res* 2022, **275:**43-47.

1087. Carretero-Barrio I, Lop-Gros J, Iglesias M, Martinez C, Matias-Guiu X, Perez-Mies B, Palacios J: **Impact of the COVID-19 pandemic on pathology training: a survey among Spanish residents.** *Virchows Arch* 2022, **481:**505-509.

1088. Carter K, Podczerwinski J, Love L, Twiss M, Blanchard A, Arora VM, Martin SK: **Utilizing Telesimulation for Advanced Skills Training in Consultation and Handoff Communication: A Post-COVID-19 GME Bootcamp Experience.** *J Hosp Med* 2021, **16:**730-734.

1089. Castioni D, Galasso O, Rava A, Masse A, Gasparini G, Mercurio M, Associazione Italiana Specializzandi in Ortopedia e T: **Has the COVID-19 Pandemic Changed the Daily Practices and Psychological State of Orthopaedic Residents?** *Clin Orthop Relat Res* 2021, **479:**1947-1954.

1090. Castro C, Antunes R, Fernandes JB, Reisinho J, Rodrigues R, Sardinha J, Vaz C, Miranda L, Simoes A: **Perceptions and Representations of Senior Nursing Students about the Transition to Professional Life during the COVID-19 Pandemic.** *Int J Environ Res Public Health* 2022, **19**.

1091. Castro GM, Taala WD, Guerrero JG, Rosales NS, De Sagun RS, Cordero RP, Ciriaco RN, Javines RG: **Nursing Educators’ and Students’ Perspectives Regarding Online Learning During the Pandemic in Saudi Arabia.** *Nursing: Research and Reviews* 2022, **Volume 12:**77-84.

1092. Cernasev A, Desai M, Jonkman LJ, Connor SE, Ware N, Sekar MC, Schommer JC: **Student Pharmacists during the Pandemic: Development of a COVID-19 Knowledge, Attitudes, and Practices (COVKAP) Survey.** *Pharmacy (Basel)* 2021, **9**.

1093. Cetinkaya S, Todil T, Kara M: **Future anxiety and coping methods of nursing students during COVID-19 pandemic: A cross-sectional study.** *Medicine (Baltimore)* 2022, **101:**e28989.

1094. Cevik H, Ungan M: **The impacts of the COVID-19 pandemic on the mental health and residency training of family medicine residents: findings from a nationwide cross-sectional survey in Turkey.** *BMC Fam Pract* 2021, **22:**226.

1095. Chachula KM, Ahmad N: **Professional quality of life, stress, and trauma in nursing students: Before and during the novel coronavirus pandemic.** *Psychol Trauma* 2022, **14:**1333-1337.

1096. Chakladar J, Diomino A, Li WT, Tsai JC, Krishnan AR, Zou AE, Kharidia K, Baig FA, Householder S, Kuo SZ, et al: **Medical student's perception of the COVID-19 pandemic effect on their education and well-being: a cross-sectional survey in the United States.** *BMC Med Educ* 2022, **22:**149.

1097. Challa S, Loomer PM, Hoff GM, Hendricson WD: **Pilot test of an emerging leaders program for dental school faculty.** *J Dent Educ* 2022, **86:**918-927.

1098. Chan E, Khong ML, Torda A, Tanner JA, Velan GM, Wong GTC: **Medical teachers' experience of emergency remote teaching during the COVID-19 pandemic: a cross-institutional study.** *BMC Med Educ* 2022, **22:**303.

1099. Chandran DS, Kaur S, Deepak KK: **Student perceptions on synchronous virtual versus face-to-face teaching for leader-centered and participant-centered postgraduate activities during COVID-19.** *Adv Physiol Educ* 2021, **45:**554-562.

1100. Chandran SK, Vilambil S, Nair SS, Vijayan SM: **Logistical Aspects of Different Online Teaching- learning Methods among Medical Students during COVID-19 in a Tertiary Care Teaching Hospital, Thrissur, Southern India.** *Journal of Clinical and Diagnostic Research* 2021, **15:**JC01-JC04.

1101. Chandrasiri NR, Weerakoon BS: **Online learning during the COVID-19 pandemic: Perceptions of allied health sciences undergraduates.** *Radiography (Lond)* 2022, **28:**545-549.

1102. Chang CY, Chung MH, Yang JC: **Facilitating nursing students' skill training in distance education via online game-based learning with the watch-summarize-question approach during the COVID-19 pandemic: A quasi-experimental study.** *Nurse Educ Today* 2022, **109:**105256.

1103. Chang MF, Liao ML, Lue JH, Yeh CC: **The impact of asynchronous online anatomy teaching and smaller learning groups in the anatomy laboratory on medical students' performance during the Covid-19 pandemic.** *Anat Sci Educ* 2022, **15:**476-492.

1104. Chang WW, Shi LX, Zhang L, Jin YL, Yu JG: **The Mental Health Status and Associated Factors Among Medical Students Engaged in Online Learning at Home During the Pandemic: A Cross-Sectional Study From China.** *Front Psychiatry* 2021, **12:**755503.

1105. Chaudhuri A, Paul S, Sarkar S: **Using technology to meet the challenges of post-graduate teaching in physiology.** *National Journal of Physiology, Pharmacy and Pharmacology* 2021, **11:**981-985.

1106. Constantin VD, Socea B, Georgeanu V, Craciun R, Bogaciu C, Smaranda A, Carap A, Nica A, Ciobotaru PV: **Surgical Stasis: Anomalies Scarcities in Surgical Residents Training in the Covid-19 Period.** *Chirurgia* 2022, **117:**55-60.

1107. Coughlan J, Timus D, Crnic T, Srdoc D, Halton C, Dragan IF: **Impact of COVID-19 on dental education in Europe: The students' perspective.** *Eur J Dent Educ* 2022, **26:**599-607.

1108. Dale RK LK, L. Furnish, T. Przkora, R. Shah, S. Spektor, B. Durbhakula, S. Lombardero, M. Brancolini, S.: **Pain Medicine Fellowship Video Interviews: A COVID-19 Trend or Here to Stay?** *Pain Physician*, **25:**125-130.

1109. Darici D, Missler M, Schober A, Masthoff M, Schnittler H, Schmitz M: **"Fun slipping into the doctor's role"-The relationship between sonoanatomy teaching and professional identity formation before and during the Covid-19 pandemic.** *Anat Sci Educ* 2022, **15:**447-463.

1110. Darnton R, Khan M, Tan XS, Jenkins M: **Primary care placements in the post-COVID era: A qualitative evaluation of a final year undergraduate clerkship.** *Med Teach* 2022, **44:**319-327.

1111. Daryanto B, Rahmadiani N, Amorga R, Kautsarani I, Susilo H, Persada Isma SP: **Burnout syndrome among residents of different surgical specialties in a tertiary referral teaching hospital in Indonesia during COVID-19 pandemic.** *Clin Epidemiol Glob Health* 2022, **14:**100994.

1112. Davies KE: **Utilizing simulation to prepare nursing students to coordinate care through transitions.** *Teach Learn Nurs* 2021, **16:**410-413.

1113. Davis A, Moore L, Farmer J, Lewis S: **Development and implementation of virtual clinical skills experiences for psychiatric nurse practitioner students.** *J Am Assoc Nurse Pract* 2021.

1114. Dev P, Thyavihally BY, Waigankar SS, Agarwal V, Pednekar AP, Shah A: **The value of webinars during COVID-19 pandemic: A questionnaire-based survey.** *Indian J Urol* 2022, **38:**204-209.

1115. Diamond-Caravella M, Fox A, Clark M, Goodstone L, Glaser C: **Alternative capstone nursing experience to scale up testing and case investigation.** *Public Health Nurs* 2022, **39:**664-669.

1116. Diaz-Guio DA, Rios-Barrientos E, Santillan-Roldan PA, Mora-Martinez S, Diaz-Gomez AS, Martinez-Elizondo JA, Barrientos-Aguinaga A, Arroyo-Romero MN, Ricardo-Zapata A, Rodriguez-Morales AJ: **Online-synchronized clinical simulation: an efficient teaching-learning option for the COVID-19 pandemic time and: beyond.** *Adv Simul (Lond)* 2021, **6:**30.

1117. DiGiusto M, Lupa MC, Corridore M, Sivak EL, Lockman JL: **Impact of the COVID-19 pandemic on the 2020 pediatric anesthesiology fellowship application cycle: A survey of program directors.** *Paediatr Anaesth* 2022, **32:**471-478.

1118. Dimeo SP, Astemborksi C, Smart J, Jones EL: **A Virtual Escape Room versus Lecture on Infectious Disease Content: Effect on Resident Knowledge and Motivation.** *West J Emerg Med* 2022, **23:**9-14.

1119. Ding JJ, Has P, Hampton BS, Burrell D: **Obstetrics and gynecology resident perception of virtual fellowship interviews.** *BMC Medical Education* 2022, **22:**58.

1120. Downie A, Mashanya T, Chipwaza B, Griffiths F, Harris B, Kalolo A, Ndegese S, Sturt J, De Valliere N, Pemba S: **Remote Consulting in Primary Health Care in Low- and Middle-Income Countries: Feasibility Study of an Online Training Program to Support Care Delivery During the COVID-19 Pandemic.** *JMIR Form Res* 2022, **6:**e32964.

1121. Drueke B, Mainz V, Lemos M, Wirtz MA, Boecker M: **An Evaluation of Forced Distance Learning and Teaching Under Pandemic Conditions Using the Technology Acceptance Model.** *Front Psychol* 2021, **12:**701347.

1122. Dua AB, Bolster MB, Gensler LS, Kolfenbach J, Mamut MH, Seo P, Jonas BL: **Virtually Speaking: The Impact of a Nationwide Synchronous and Asynchronous Learning Forum.** *Arthritis Care Res (Hoboken)* 2022.

1123. Duan H, Gong M, Zhang Q, Huang X, Wan B: **Research on sleep status, body mass index, anxiety and depression of college students during the post-pandemic era in Wuhan, China.** *J Affect Disord* 2022, **301:**189-192.

1124. Duan Y, Li Z, Wang X, Gao Z, Zhang H: **Application of online case-based learning in the teaching of clinical anesthesia for residents during the COVID-19 epidemic.** *BMC Med Educ* 2021, **21:**609.

1125. Duarte I, Alves A, Coelho A, Ferreira A, Cabral B, Silva B, Peralta J, Silva J, Domingues P, Nunes P, et al: **The Mediating Role of Resilience and Life Satisfaction in the Relationship between Stress and Burnout in Medical Students during the COVID-19 Pandemic.** *Int J Environ Res Public Health* 2022, **19**.

1126. Francis YM: **Views on virtual education during the COVID-19 pandemic among medical and paramedical students in India.** *Bioinformation* 2022, **18:**518-524.

1127. Frank V, Doshi A, Demirjian NL, Fields BKK, Song C, Lei X, Reddy S, Desai B, Harvey DC, Cen S, Gholamrezanezhad A: **Educational, psychosocial, and clinical impact of SARS-CoV-2 (COVID-19) pandemic on medical students in the United States.** *World J Virol* 2022, **11:**150-169.

1128. Freeman CW, Dhanaliwala A, Moore S, Kunchala S, Scanlon MH: **Homeward Bound: A Comparison of Resident Case Volume on Home-Read Workstations and On-Site During the COVID-19 Pandemic.** *Journal of the American College of Radiology* 2022, **19:**476-479.

1129. Freeman R, Knights J, Beaton L, Araujo M, Yuan S, Clarkson J, Young L, Humphris G: **Prediction of emotional exhaustion over the course of the COVID-19 pandemic in primary care dental staff: an intensive longitudinal study.** *BMJ Open* 2021, **11**.

1130. Frenkel SMV OOS, I. V. Savytskyi, V. I. Badiuk, N. S.: **Features of students mental health indicators during the academic year under pandemic conditions of COVID-19.** **2:**776-780.

1131. Frich JC, Bratholm C, Ravnestad H, Friberg ML, Mjåset C, Kaarbøe OM: **Medical leadership development during the COVID-19 pandemic.** *BMJ Leader* 2022.

1132. Frohna JG, Waggoner-Fountain LA, Edwards J, Fussell JJ, Wueste B, Gigante J, Vinci RJ, Heitkamp NM, Neelakantan MK, Degnon LE, et al: **National Pediatric Experience With Virtual Interviews: Lessons Learned and Future Recommendations.** *Pediatrics* 2021, **148**.

1133. Frutiger M, Whillier S: **Effects of a sudden change in curriculum delivery mode in postgraduate clinical studies, following the COVID-19 pandemic.** *J Chiropr Educ* 2022, **36:**132-141.

1134. Frydman JL, Gelfman LP, Lindenberger EC, Smith CB, Berns S, Kelley AS, Dow LA: **Virtual Geritalk: Improving Serious Illness Communication of Clinicians Who Care for Older Adults.** *J Pain Symptom Manage* 2021, **62:**e206-e212.

1135. FS ESR, de Castro Oliveira L, Mardegan Goncalves DF, Rocha RS, Bresciani E, Fagundes TC: **Influence of knowledge on Brazilian student's perception of life and clinical classes during COVID-19.** *J Educ Health Promot* 2022, **11:**62.

1136. Funakoshi H, Matsumura Y, Maruhashi T, Ishida K, Funabiki T, team D-RC: **Difference in postcourse knowledge and confidence between Web-based and on-site training courses on resuscitative endovascular balloon occlusion of the aorta.** *Acute Med Surg* 2021, **8:**e707.

1137. Fung C, Maxwell N, Powell S, Benassai M, Chunga N, Corcoran J, Barbosa W, Lopez M, Hanampa B, Llaiqui-Condori M, et al: **Virtual Adaptation of an International Exchange Program in Medical Education.** *Ann Glob Health* 2022, **88:**52.

1138. Gachanja F, Mwangi N, Gicheru W: **E-learning in medical education during COVID-19 pandemic: experiences of a research course at Kenya Medical Training College.** *BMC Med Educ* 2021, **21:**612.

1139. Gadi N, Saleh S, Johnson JA, Trinidade A: **The impact of the COVID-19 pandemic on the lifestyle and behaviours, mental health and education of students studying healthcare-related courses at a British university.** *BMC Med Educ* 2022, **22:**115.

1140. Gaigbe-Togbe BHA, Menhaji K, Tran AD, Bui AH, Ascher-Walsh C, Dabney L, Hardart A: **Virtual Interviews During COVID-19 Pandemic: A Survey of Applicants to Fellowships in Female Pelvic Medicine and Reconstructive Surgery.** *Female Pelvic Med Reconstr Surg* 2021, **27:**e626-e629.

1141. Kim EG ES, G. Ohana, G. Jotkowitz, A. Taragin, B. H.: **Adapting Surgical Clerkship Curricula in Response to the COVID-19 Pandemic.** *Isr Med Assoc J*, **23:**690-692.

1142. Konopinska J, Obuchowska I, Lisowski L, Dub N, Dmuchowska DA, Rekas M: **Impact of the COVID-19 pandemic on ophthalmic specialist training in Poland.** *PLoS One* 2021, **16:**e0257876.

1143. Kor PPK, Liu JYW, Kwan RYC: **Exploring nursing students' learning experiences and attitudes toward older persons in a gerontological nursing course using self-regulated online enquiry-based learning during the COVID-19 pandemic: A mixed-methods study.** *Nurse Education Today* 2022, **111:**105301.

1144. Kositanurit W, Vivatvakin S, Kaikaew K, Varachotisate P, Burana C, Chayanupatkul M, Thanprasertsuk S, Wangsaturaka D, Kulaputana O: **Asynchronous online lecture may not be an effective method in teaching cardiovascular physiology during the COVID-19 pandemic.** *BMC Med Educ* 2022, **22:**162.

1145. Koubek R, Brady A, Gopalratnam K, Oeckler R, Kennedy CC, Kelm D: **Virtual Procedural Supervision During the COVID-19 Pandemic: A Novel Pilot for Supervising Invasive Bedside Procedures in the ICU.** *Mayo Clin Proc Innov Qual Outcomes* 2021, **5:**992-996.

1146. Kraft DO, Bowers EMR, Smith BT, Jabbour N, Schaitkin BM, O'Leary MA, Groblewski JC, Young VN, Sridharan S: **Applicant Perspectives on Virtual Otolaryngology Residency Interviews.** *Ann Otol Rhinol Laryngol* 2022, **131:**1325-1332.

1147. Krambeck A, Loth AG, Leinung M, Syed-Ali A, Filmann N, Kramer S, Baumann U, Stover T, Diensthuber M: **[Has the SARS-CoV-2 pandemic improved teaching?-Virtual education in otorhinolaryngology from the students' perspective].** *HNO* 2022, **70:**655-665.

1148. Kramer B, Plitt G, French JC, Nygaard RM, Cassaro S, Edelman DA, Lees JS, Meier AH, Joshi ART, Johnson MP, et al: **A Multicenter Analysis of the Early Impact of COVID-19 on Junior Resident Operative Case Volume.** *J Surg Res* 2022, **279:**208-217.

1149. Krauss F, Giesler M, Offergeld C: **[On the effectiveness of digital teaching of practical skills in curricular ENT education].** *HNO* 2022, **70:**287-294.

1150. Krier CR, Quinn K, Kaljo K, Farkas AH, Ellinas EH: **The Effect of COVID-19 on the Medical School Experience, Specialty Selection, and Career Choice: A Qualitative Study.** *J Surg Educ* 2022, **79:**661-667.

1151. Krifa I, Hallez Q, van Zyl LE, Braham A, Sahli J, Ben Nasr S, Shankland R: **Effectiveness of an online positive psychology intervention among Tunisian healthcare students on mental health and study engagement during the Covid-19 pandemic.** *Appl Psychol Health Well Being* 2021.

1152. Krifa I, van Zyl LE, Braham A, Ben Nasr S, Shankland R: **Mental Health during COVID-19 Pandemic: The Role of Optimism and Emotional Regulation.** *Int J Environ Res Public Health* 2022, **19**.

1153. Leibner ES, Baron EL, Shah RS, Philpotts Y, Sreeramoju D, Jawaid Y, DeVivo A, Acquah S, Hsieh J, Gidwani U, et al: **Critical Care Simulation Education Program During the COVID-19 Pandemic.** *J Patient Saf* 2022, **18:**e810-e815.

1154. Leighton K, Kardong-Edgren S, Jones A, Reedy G: **Sidelined during COVID-19: a narrative inquiry into how simulationists experienced the pandemic.** *Adv Simul (Lond)* 2022, **7:**1.

1155. Lengetti E, Cantrell MA, DellaCroce N, Diewald L, Mensinger JL, Shenkman R: **Learning environment and evidence among professionals and students satisfaction (LEAPS), experienced during the COVID-19 pandemic.** *Teach Learn Nurs* 2021, **16:**342-346.

1156. Leong Bin Abdullah MFI, Mansor NS, Mohamad MA, Teoh SH: **Quality of life and associated factors among university students during the COVID-19 pandemic: a cross-sectional study.** *BMJ Open* 2021, **11:**e048446.

1157. Lepore B, Autorino G, Coppola V, Borgogni R, Carulli R, Cerulo M, Califano G, Cardone R, Escolino M, Esposito C: **Effectiveness of a Laparoscopic Training Model for Pediatric Surgeons During COVID-19 Pandemic: A Single-Center Experience.** *J Laparoendosc Adv Surg Tech A* 2022, **32:**907-912.

1158. Lestari W, Ichwan SJA, Yaakop SZ, Sabaznur N, Ismail A, Sukotjo C: **Online Learning during the COVID-19 Pandemic: Dental Students' Perspective and Impact on Academic Performance, One Institution Experience.** *Dent J (Basel)* 2022, **10**.

1159. Li G, Yu T, Zhang L, Du H, Zhang W, Hou S: **Use of a specialty endoscopy online platform for continuing medical education for clinical endoscopists during the COVID-19 pandemic.** *BMC Med Educ* 2022, **22:**458.

1160. Li L, Hang R, Qin L, Liu H, Liu H, Hu F, Huang L: **Relationship between stress response and depression in vocational medical school students in the COVID-19 epidemic: A moderated mediation model.** *Acta Psychol (Amst)* 2022, **227:**103617.

1161. Li L, Wu H, Xie A, Ye X, Liu C, Wang W: **Students' initial perspectives on online learning experience in China during the COVID-19 outbreak: expanding online education for future doctors on a national scale.** *BMC Med Educ* 2021, **21:**584.

1162. Li S, Zhang C, Liu Q, Tong K: **E-Learning during COVID-19: perspectives and experiences of the faculty and students.** *BMC Med Educ* 2022, **22:**328.

1163. Li X, Li M, Kathirvel R: **Modified OBGYN undergraduate medical education during the COVID-19 pandemic in Singapore.** *Singapore Med J* 2021.

1164. Li Y, Sears NA, Murray IVJ, Yadav KK: **Rethinking Teaching Team-Based Learning: The Challenges and Strategies for Medical Education in a Pandemic.** *AERA Open* 2021, **7**.

1165. Liang L, Feng L, Zheng X, Wu Y, Zhang C, Li J: **Effect of dialectical behavior group therapy on the anxiety and depression of medical students under the normalization of epidemic prevention and control for the COVID-19 epidemic: a randomized study.** *Ann Palliat Med* 2021, **10:**10591-10599.

1166. Liaquat A, Atta K, Khan RA: **Challenges Faced in Teaching and Learning during Clinical Years of Dentistry amidst the Covid-19 Pandemic.** *Pakistan Journal of Medical and Health Sciences* 2021, **15:**2253-2256.

1167. Liaw SY, Rusli KDB, McKenna L, Tan JZ, Lau ST: **Effectiveness of an online program using telesimulation for academic-clinical collaboration in preparing nurse preceptors' roles.** *J Clin Nurs* 2022.

1168. Lim C, De Silva I, Moussa G, Islam T, Osman L, Malick H, Deol S, Youssef M, Farrag A, Ashraf R, et al: **Redeployment of ophthalmologists in the United Kingdom during the Coronavirus Disease Pandemic.** *Eur J Ophthalmol* 2021, **31:**2268-2274.

1169. Lim J, Ko H, Park J, Ihm J: **Effect of active learning and online discussions on the academic performances of dental students.** *BMC Med Educ* 2022, **22:**312.

1170. Lima LDG, Tomaschewski-Barlem JG, Paloski GDR, Barlem ELD, Rocha LP, Castanheira JS: **The performance of nursing students in a tele-health service during the COVID-19 pandemic.** *Rev Gaucha Enferm* 2021, **42:**e20200483.

1171. Lin E, You AX, Wardi G: **Comparison of In-Person and Telesimulation for Critical Care Training during the COVID-19 Pandemic.** *ATS Sch* 2021, **2:**581-594.

1172. Lin ZM, Hong YR, Liu CM, Luo ZY, Zhang Y, Xie XJ, Huang PT: **PBL teaching in ultrasonography resident standardization training in the COVID-19 pandemic.** *BMC Med Educ* 2022, **22:**512.

1173. Lissinna B, Rashid M, Foulds JL, Forbes KL: **Embracing uncertainty: medical student perceptions of a pediatric bootcamp developed in response to mandated changes during the pandemic.** *BMC Med Educ* 2022, **22:**390.

1174. Liu L, Caliph S, Simpson C, Khoo RZ, Neviles G, Muthumuni S, Lyons KM: **Pharmacy Student Challenges and Strategies towards Initial COVID-19 Curriculum Changes.** *Healthcare (Basel)* 2021, **9**.

1175. Liu Y, Cao Z: **The impact of social support and stress on academic burnout among medical students in online learning: The mediating role of resilience.** *Front Public Health* 2022, **10:**938132.

1176. Ludden-Schlatter A, Clithero-Eridon A, Myers O, Kelts K, Scartozzi C, Kibria Z, Wilson G, Jarrett JB: **Predictive Factors of Positive E-Learning Experience.** *Fam Med* 2022, **54:**441-451.

1177. Lugito NPH, Kurniawan A, Lorens JO, Sieto NL: **Mental health problems in Indonesian internship doctors during the COVID-19 pandemic.** *J Affect Disord Rep* 2021, **6:**100283.

1178. Ma V, Scott T, Ott M, Karimuddin A: **Adaptations to general surgery resident education in response to COVID-19.** *Can J Surg* 2021, **64:**E543-E549.

1179. Maar M, McGregor L, Desjardins D, Delaney KZ, Bessette N, Reade M: **Teaching Culturally Safe Care in Simulated Cultural Communication Scenarios During the COVID-19 Pandemic: Virtual Visits with Indigenous Animators.** *J Med Educ Curric Dev* 2022, **9:**23821205221091034.

1180. Macht L, Worlitzsch D, Braijoshri N, Bequiri P, Zudock J, Zilezinski M, Stoevesandt D, Smith J, Hofstetter S: **COVID-19: Development and implementation of a video-conference-based educational concept to improve the hygiene skills of health and nursing professionals in the Republic of Kosovo.** *GMS Hyg Infect Control* 2022, **17:**Doc09.

1181. Macluskey M, Anderson AS, Gribben M, Shepherd SD: **An Educational Evaluation of Thiel Cadavers as a Model for Teaching Suturing Skills to Dental Students during the COVID-19 Pandemic.** *Dent J (Basel)* 2022, **10**.

1182. Mady AF, Odat MA, Ramadan OA, Mahmood W, Alyousef R, Alzahrani M, Aziz Alshehri LA, Abuhamdah M, Rahman BA, Mhawish HA: **Experiences of Non-ICU Physicians Redeployment in ICU during COVID-19.** *Pakistan Journal of Medical and Health Sciences* 2022, **16:**306-309.

1183. Magdalinou A, Liaskos J, Isaakidou M, Mantas J: **The Transition to Distance Learning in the Era of Covid-19 Pandemic: The Perceptions and Experiences of Nursing Students.** *Stud Health Technol Inform* 2022, **295:**495-498.

1184. Maharjan BRD RPP, P. Byanju, J. Aryal, S.: **Perception of Students on Online Assessment: A Descriptive Study among Medical Undergraduates at Basic Sciences.** *Kathmandu University Medical Journal*, **19:**451-459.

1185. Mahdavinoor SMM, Rafiei MH, Mahdavinoor SH: **Mental health status of students during coronavirus pandemic outbreak: A cross-sectional study.** *Ann Med Surg (Lond)* 2022, **78:**103739.

1186. Maile EL, Horsley SM, Dunn T, Knight JM: **Initial impact of the COVID-19 pandemic on public health training: participatory action research to understand experiences in the East Midlands.** *J Public Health (Oxf)* 2021.

1187. Major S, Krage R, Lazarovici M: **SimUniversity at a distance: a descriptive account of a team-based remote simulation competition for health professions students.** *Adv Simul (Lond)* 2022, **7:**6.

1188. Mak DB, Russell K, Griffiths D, Vujcich DL, Strasser R: **Lessons learned from adapting a remote area health placement from physical to virtual: a COVID-19-driven innovation.** *Int J Med Educ* 2021, **12:**274-299.

1189. Mak V, Krishnan S, Chuang S: **Students' and Examiners' Experiences of Their First Virtual Pharmacy Objective Structured Clinical Examination (OSCE) in Australia during the COVID-19 Pandemic.** *Healthcare (Basel)* 2022, **10**.

1190. Makhado L, Musekwa OP, Luvhengo M, Murwira T, Lebese RT, Mulaudzi MT, Chueng MJ: **An Exploratory-Descriptive Study on the Impact of COVID-19 on Teaching and Learning: The Experiences of Student Nurses in the Rural-Based Historically Disadvantaged University of South Africa.** *Inquiry* 2022, **59:**469580221093191.

1191. Malhotra A, Kreys E, Feng X: **Impact of a Prepharmacy Program on Students' Self-Awareness of Pharmacist Professional Identity: Comparison between Virtual and In-Person Settings.** *Pharmacy (Basel)* 2022, **10**.

1192. Malik PRV, Nakhla N: **Instructor-Blinded Study of Pharmacy Student Learning When a Flipped Online Classroom Was Implemented during the COVID-19 Pandemic.** *Pharmacy (Basel)* 2022, **10**.

1193. Mamnuah M, Wantonoro W: **Online Learning Methods during COVID-19 Pandemic: On an Indonesian Nursing Student Experience.** *Open Access Macedonian Journal of Medical Sciences* 2022, **10:**105-111.

1194. Manakatt BM, Carson ZW, Penton RL, Demello AS: **Virtual learning experiences in population health nursing course during the COVID-19 pandemic.** *Int Nurs Rev* 2021, **68:**557-562.

1195. Manavalan V, Aswini Dutt R, Harishchandra B, Nirgude A: **Continuum of Surgical Skill Acquisition for the Postgraduate Residents During COVID Pandemic: Role of Advanced Simulators in a Multipronged Modular Approach.** *Indian J Surg Oncol* 2021, **12:**270-278.

1196. Mandarano P, Squatrito V, Mariotti A, Presti G: **Second Line Volunteering in Lombardy Covid-19 Emergency as a Perspective on Medical Education and Psychological Distress.** *Clin Neuropsychiatry* 2021, **18:**278-287.

1197. Manggala SK, Tantri AR, Sugiarto A, Sianipar IR, Prasetyono TOH: **In situ simulation training for a better interprofessional team performance in transferring critically ill patients with COVID-19: a prospective randomised control trial.** *Postgrad Med J* 2022, **98:**617-621.

1198. Manik M, Gultom E, Sibuea R, Pailak H: **Virtual Simulation Learning from Indonesian Nursing Students’ Perspectives.** *Open Access Macedonian Journal of Medical Sciences* 2022, **10:**112-117.

1199. Mann S, Duffy J, Muffly T, Tilva K, Gray S, Hetzler L, Kraft S, Malekzadeh S, Pletcher S, Cabrera-Muffly C: **Effect of the COVID-19 Pandemic on Otolaryngology Trainee Surgical Case Numbers: A Multi-institutional Review.** *Otolaryngol Head Neck Surg* 2022**:**1945998221083845.

1200. Manou E, Lazari EC, Lazaris AC, Agrogiannis G, Kavantzas NG, Thomopoulou GE: **Evaluating e-Learning in the Pathology Course During the COVID-19 Pandemic.** *Adv Med Educ Pract* 2022, **13:**285-300.

1201. Manou E, Lazari EC, Thomopoulou GE, Agrogiannis G, Kavantzas N, Lazaris AC: **Participation and Interactivity in Synchronous E-Learning Pathology Course During the COVID-19 Pandemic.** *Adv Med Educ Pract* 2021, **12:**1081-1091.

1202. Maravent S, Steinberg JG, Moreau C, Murzello A, Lendoiro J, Hesly J: **Students' Perceptions and Lessons Learned From Virtual APPE Rotations: A Multi-Institutional Perspective.** *J Pharm Pract* 2022**:**8971900221104256.

1203. Chootong R, Sono S, Choomalee K, Wiwattanaworaset P, Phusawat N, Wanghirankul N, Laojaroensuk P, Thongkhundum P, Saetang R, Euanontat S, Anantathaweekul S: **The association between physical activity and prevalence of anxiety and depression in medical students during COVID-19 pandemic: A cross-sectional study.** *Ann Med Surg (Lond)* 2022, **75:**103408.

1204. Choudry ZA, Ayub A, Badar SE: **Preparedness of Medical Graduates to serve in clinical settings independently: An exploratory qualitative study.** *Pak J Med Sci* 2022, **38:**785-790.

1205. Chowdhury K, Haque M, Lutfor AB, Siddiqui TH, Ahmad R, Sultana I, Sharma P, Lugova H, Etando A, Godman B: **Impact of the COVID-19 pandemic on dental and medical education in Bangladesh: a pilot study and the implications.** *Bangladesh Journal of Medical Science* 2022, **21:**444-454.

1206. Chua WL, Ooi SL, Chan GWH, Lau TC, Liaw SY: **The Effect of a Sepsis Interprofessional Education Using Virtual Patient Telesimulation on Sepsis Team Care in Clinical Practice: Mixed Methods Study.** *J Med Internet Res* 2022, **24:**e35058.

1207. Cimen ID, Alvur TM, Coskun B, Sukur NEO: **Mental health of Turkish medical students during the COVID-19 pandemic.** *Int J Soc Psychiatry* 2022, **68:**1253-1262.

1208. Constantinescu G, Gheorghe G, Ionescu VA, Plotogea O-M, Sandru V, Diaconu CC, Varlas VN, Bacalbasa N, Diaconu CC, Stan-Ilie M: **COVID-19 Pandemic and Its Impact on Training Programs of Medical Residency in Romania.** *Gastroenterology Insights* 2022, **13:**106-116.

1209. Cooper L, Din AH, Fitzgerald O'Connor E, Roblin P, Rose V, Mughal M: **Augmented Reality and Plastic Surgery Training: A Qualitative Study.** *Cureus* 2021, **13:**e19010.

1210. Corlade-Andrei M, Mairean C, Nedelea P, Grigorasi G, Cimpoesu D: **Burnout Syndrome among Staff at an Emergency Department during the COVID-19 Pandemic.** *Healthcare (Basel)* 2022, **10**.

1211. Corner EJ, Zhang X, Van Willigen Z, Tatam K, Camilleri M, Monkhouse A, Bear DE, Hemsley A, Puthucheary Z, Rosenberg A, et al: **Mixed methods evaluation of the impact of the COVID-19 ICU remote-learning rehabilitation course for frontline health professionals during the COVID-19 pandemic in the UK.** *Journal of the Intensive Care Society* 2021.

1212. Freitas DQ, Ludovichetti FS, Cascante-Sequeira D, Santaella GM, Brasil DM, Costa ED: **Digital Technology in Dental Education During COVID-19 Pandemic: Worldwide Experience of Professors and Students.** *Odovtos - International Journal of Dental Sciences* 2021, **23:**505-534.

1213. Costea CA, Popescu DM, Roman A, Stratul SI, Surlin P, Negucioiu M, Micu IC, Ciurea A, Lucaciu PO, Lazar L, et al: **The Impact of the COVID-19 Pandemic on Romanian Postgraduate Periodontal Residency Teaching: Past Experience, Present Imperatives and Future Considerations in a Multicentric Evaluation.** *Int J Environ Res Public Health* 2022, **19**.

1214. Cowan E, Altschafl B, Foertsch J, Barnes D, Lasarev M, Pelley E: **A New Normal: Assessment Outcomes and Recommendations for Virtual Versus In-Person Curricula in Post-COVID-19 Times.** *Med Sci Educ* 2022, **32:**379-387.

1215. De la Cerda-Vargas MF, Stienen MN, Campero A, Perez-Castell AF, Soriano-Sanchez JA, Nettel-Rueda B, Borba LAB, Castillo-Rangel C, Navarro-Dominguez P, Munoz-Hernandez MA, et al: **Burnout, Discrimination, Abuse, and Mistreatment in Latin America Neurosurgical Training During the Coronavirus Disease 2019 Pandemic.** *World Neurosurg* 2022, **158:**e393-e415.

1216. de Las Cuevas C, Gutierrez-Rojas L, Alvarez-Mon MA, Andreu-Bernabeu A, Capitan L, Gomez JC, Grande I, Hidalgo-Mazzei D, Mateos R, Moreno-Gea P, et al: **Evaluating the Effect of a Telepsychiatry Educational Program on the Awareness, Knowledge, Attitude, and Skills of Telepsychiatry Among Spanish Psychiatrists during COVID-19 Pandemic.** *Telemed J E Health* 2022.

1217. de Oliveira Kubrusly Sobral JB, Lima DLF, Lima Rocha HA, de Brito ES, Duarte LHG, Bento L, Kubrusly M: **Active methodologies association with online learning fatigue among medical students.** *BMC Med Educ* 2022, **22:**74.

1218. de Souza KC, Mendes TB, Gomes THS, da Silva AA, Nali L, Bachi ALL, Rossi FE, Gil S, Franca CN, Neves LM: **Medical Students Show Lower Physical Activity Levels and Higher Anxiety Than Physical Education Students: A Cross-Sectional Study During the COVID-19 Pandemic.** *Front Psychiatry* 2021, **12:**804967.

1219. de-Diego-Cordero R, Martinez-Del-Carmen C, Bonilla Sierra P, Vargas-Martinez AM: **Impact of the COVID-19 Pandemic and Psychosocial Coping Strategies in Health Sciences Students at the University of Seville: A Pilot Study.** *Healthcare (Basel)* 2021, **9**.

1220. Debnath M, Ojha S, Niraula A, Sharma D: **Perceptions of Medical and Allied Health Students Towards Online Education during the COVID-19 Pandemic Phases and Its Future Impact in India.** *J Eur CME* 2021, **10:**1993428.

1221. Debnath PR, Islam MS, Karmakar PK, Sarker R, Zhai ZW, Potenza MN: **Mental Health Concerns, Insomnia, and Loneliness Among Intern Doctors Amidst the COVID-19 Pandemic: Evidence from a Large Tertiary Care Hospital in Bangladesh.** *International Journal of Mental Health and Addiction* 2021**:**44582.

1222. Dehghanpour M, Baker J: **The Impact of COVID-19 on Medical Dosimetry Education: Students' Perception on the Effectiveness of Program's Immediate Response.** *Med Dosim* 2022, **47:**123-128.

1223. DeLay TK, Singh NP, Duong TA, Rais-Bahrami S, King TW, Chen H, Corey BL: **Virtual Interactions and the 2020-2021 Residency Application Cycle in General Surgery: A Look Ahead.** *J Surg Res* 2022, **278:**331-336.

1224. Deshmukh D, Patil AV, Deore R, Bhise MC: **Impact of COVID-19 Pandemic on Postgraduate Psychiatry Teaching- Learning and Evaluation in India: A Nationwide Survey.** *Indian J Psychol Med* 2022, **44:**479-484.

1225. Donesky D, Norton L, Fisher E, Bunker-Alberts M: **Health services and the Project RoomKey COVID-19 initiative for the unhoused: A university and community partnership.** *J Prof Nurs* 2021, **37:**1175-1179.

1226. Donn J, Scott JA, Binnie V, Mather C, Beacher N, Bell A: **Virtual objective structured clinical examination during the COVID-19 pandemic: An essential addition to dental assessment.** *Eur J Dent Educ* 2022.

1227. Donovan CM, Cooper A, Kim S: **Ready Patient One: How to Turn an In-Person Critical Care Simulation Scenario Into an Online Serious Game.** *Cureus* 2021, **13:**e17746.

1228. Duggan I, Hablase R, Beard L, Odejinmi F, Mallick R: **The impact of COVID-19 on O&G trainees; where are we now?** *Facts Views Vis Obgyn* 2022, **14:**69-75.

1229. Durani U, Major A, Velazquez AI, May J, Nelson M, Zheng Z, Hall AG, Alam ST, Reynolds R, Thompson JC, et al: **Impact of COVID-19 on Hematology-Oncology Trainees: A Quantitative and Qualitative Assessment.** *JCO Oncol Pract* 2022, **18:**e586-e599.

1230. Durns T, Gethin-Jones T, Monson E, O'Donohoe J: **Response of US psychiatric programs to the COVID-19 pandemic and the impact on trainees.** *BMC Med Educ* 2022, **22:**229.

1231. Duszenko M, Frohlich N, Kaupp A, Garaschuk O: **All-digital training course in neurophysiology: lessons learned from the COVID-19 pandemic.** *BMC Med Educ* 2022, **22:**3.

1232. Dyrek N, Wikarek A, Niemiec M, Owczarek AJ, Olszanecka-Glinianowicz M, Kocelak P: **The perception of e-learning during the SARS-CoV-2 pandemic by students of medical universities in Poland - a survey-based study.** *BMC Med Educ* 2022, **22:**529.

1233. Dzara K, Pusic M, Carlile N, Krupat E, Alexander EK: **Educational adaptation to clinical training during the COVID-19 pandemic: a process analysis.** *BMC Med Educ* 2022, **22:**200.

1234. Dziurka M, Machul M, Ozdoba P, Obuchowska A, Kotowski M, Grzegorczyk A, Pydys A, Dobrowolska B: **Clinical Training during the COVID-19 Pandemic: Experiences of Nursing Students and Implications for Education.** *Int J Environ Res Public Health* 2022, **19**.

1235. Dzurec L, Tackett M, Enslow L: **COVID-19-Mediated Strengths and Challenges for 2021 Nursing Graduates: Implications for Nurse Leaders and Educators.** *Nurse Lead* 2022, **20:**168-173.

1236. Ecker A, Berenson AB, Gonzalez SJ, Zoorob R, Hirth JM: **Depression among medical students in the United States during the COVID-19 pandemic: The role of communication between universities and their students.** *Disaster Med Public Health Prep* 2022**:**1-21.

1237. Eden E, Kuru E: **Massive online open course on pediatric dentistry for senior undergraduates during COVID-19 pandemic.** *J Dent Educ* 2022, **86 Suppl 1:**780-782.

1238. Egarter S, Mutschler A, Brass K: **Impact of COVID-19 on digital medical education: compatibility of digital teaching and examinations with integrity and ethical principles.** *International Journal for Educational Integrity* 2021, **17**.

1239. Egilsdottir HO, Heyn LG, Brembo EA, Byermoen KR, Moen A, Eide H: **The value of a redesigned clinical course during COVID-19 pandemic: an explorative convergent mixed-methods study.** *BMC Nurs* 2022, **21:**94.

1240. Eraydin C, Alpar SE: **The effect of laughter therapy on nursing students' anxiety, satisfaction with life, and psychological well-being during the COVID-19 pandemic: Randomized controlled study.** *Adv Integr Med* 2022, **9:**173-179.

1241. Erfannia L, Sharifian R, Yazdani A, Sarsarshahi A, Rahati R, Jahangiri S: **Students' Satisfaction and e-Learning Courses in Covid-19 Pandemic Era: A Case Study.** *Stud Health Technol Inform* 2022, **289:**180-183.

1242. Erklauer JC, Thomas AX, Hong SJ, Appavu BL, Carpenter JL, Chiriboga-Salazar NR, Ferrazzano PA, Goldstein Z, Griffith JL, Guilliams KP, et al: **A Virtual Community of Practice: An International Educational Series in Pediatric Neurocritical Care.** *Children (Basel)* 2022, **9**.

1243. Ernst J, Jordan K-D, Weilenmann S, Sazpinar O, Gehrke S, Paolercio F, Petry H, Pfaltz MC, Méan M, Aebischer O, et al: **Burnout, depression and anxiety among Swiss medical students – A network analysis.** *Journal of Psychiatric Research* 2021, **143:**196-201.

1244. Eroglu E, Kolcu G, Kolcu MIB: **The Effect of Distance Education Conducted during the COVID-19 Pandemic Period on the Psychomotor Skill Development of a Dental School Students.** *Biomed Res Int* 2022, **2022:**6194200.

1245. Escobio-Prieto I, Sobrino-Sánchez R, Mingorance JA, García-Marín M, Matas-Terrón A, Albornoz-Cabello M: **Analysis of the Satisfaction Degree of Students at Spain’s Physiotherapy Universities in Relation to Online Teaching during the COVID-19 Pandemic.** *Sustainability* 2021, **13**.

1246. Esmat S, Attia A, Elhabashi E: **Prevalence and Predictors for Depression among Medical Students during Coronavirus Disease-19 Pandemic: A Cross-sectional Study.** *Open Access Macedonian Journal of Medical Sciences* 2021, **9:**1454-1460.

1247. Espin S, LeGrow K, Bookey-Bassett S, Rose D, Santa Mina E, Indar A: **Exploring the Intersection Between Academic and Professional Practice During the COVID-19 Pandemic: Undergraduate and Graduate Nursing Students' Experiences.** *Can J Nurs Res* 2022, **54:**283-291.

1248. Esquivel EL, De Angelis P, Chae JK, Safdieh JE, Abramson EL, Kang Y: **Transitioning preclinical students into clerkships amidst curricular disruptions from the COVID-19 pandemic.** *Med Educ Online* 2021, **26:**1996216.

1249. Essadek A, Gressier F, Robin M, Shadili G, Bastien L, Peronnet JC, Falissard B, Rabeyron T: **Mental health of medical students during the COVID19: Impact of studies years.** *J Affect Disord Rep* 2022, **8:**100318.

1250. Etajuri EA, Mohd NR, Naimie Z, Ahmad NA: **Undergraduate dental students' perspective of online learning and their physical and mental health during COVID-19 pandemic.** *PLoS One* 2022, **17:**e0270091.

1251. Eveland AP, Prado LG, Wilhelm SR, Wong S, Barsky SH: **The virtues of the virtual medical school interview.** *Med Educ Online* 2021, **26:**1992820.

1252. Ezbercİ S, Yildirim B: **Evaluation of the Effectiveness of Distance Learning in Dental Education During COVID-19 Pandemic.** *Clinical and Experimental Health Sciences* 2021, **11:**630-636.

1253. Filipe HP, Golnik KC, Geary A, Buque A, Mack HG: **Online Faculty Development: An African Lusophone Ophthalmic Society Experience During the COVID-19 Pandemic.** *Middle East Afr J Ophthalmol* 2021, **28:**230-238.

1254. Findyartini A, Greviana N, Hanum C, Husin JM, Sudarsono NC, Krisnamurti DGB, Rahadiani P: **Supporting newly graduated medical doctors in managing COVID-19: An evaluation of a Massive Open Online Course in a limited-resource setting.** *PLoS One* 2021, **16:**e0257039.

1255. Fitzgerald N, Moylett E, Gaffney G, McCarthy G, Fapohunda O, Murphy AW, Geoghegan R, Hallahan B: **Undertaking a face-to-face objective structured clinical examination for medical students during the COVID-19 pandemic.** *Ir J Psychol Med* 2022**:**1-7.

1256. Flake NM, Chan DCN, DiMarco AC, Silverstein BD: **Use of a Knowledge-Based Governance Approach to Plan a Post-COVID-19 Predoctoral Dental.** *Dent J (Basel)* 2021, **9**.

1257. Flo J, Byermoen KR, Egilsdottir HO, Eide H, Heyn LG: **Nursing students' experiences of virtual simulation when using a video conferencing system - a mixed methods study.** *Int J Nurs Educ Scholarsh* 2021, **18**.

1258. Flolo TN, Gjeilo KH, Andersen JR, Haraldstad K, Hjelmeland IHH, Iversen MM, Loyland B, Norekval TM, Riiser K, Rohde G, et al: **The impact of educational concerns and satisfaction on baccalaureate nursing students' distress and quality of life during the Covid-19 pandemic; a cross-sectional study.** *BMC Nurs* 2022, **21:**185.

1259. Flurin L, Tekin A, Bogojevic M, Zec S, Sun Y, Li H, Finch A, Ahmad S, Kashyap R, Gajic O, et al: **International Virtual Simulation Education in Critical Care During COVID-19 Pandemic: Preliminary Description of the Virtual Checklist for Early Recognition and Treatment of Acute Illness and iNjury Program.** *Simul Healthc* 2022, **17:**205-207.

1260. Cirakoglu NY, Ozbay Y: **Evaluation of distance learning and online exam experience of Turkish undergraduate dental students during the Covid-19 pandemic.** *Niger J Clin Pract* 2022, **25:**916-922.

1261. Clanner-Engelshofen BM, Frommherz L, Mitwalli M, Stadler PC, French LE, Reinholz M: **3D printing and silicone models of primary skin lesions for dermatological education as remote learning tool.** *J Dtsch Dermatol Ges* 2022, **20:**177-183.

1262. Clemmons A, Lebovitz L, Fulford M, Greene K, Franks A, Branan T, Plake KI: **Impact of the COVID-19 pandemic on faculty at research-intensive United States schools/colleges of pharmacy.** *Curr Pharm Teach Learn* 2022, **14:**145-152.

1263. Co M, Chu KM: **A prospective case-control study on online teaching of ultrasonography skills to medical students during COVID-19 pandemic.** *Heliyon* 2022, **8:**e08744.

1264. Coakley KE, Gonzales-Pacheco D: **Dietetics Students' Perceptions of Academic and Health Impacts of the COVID-19 Pandemic.** *Top Clin Nutr* 2022, **37:**227-235.

1265. Cockburn JG, Tan CY, Poh D, Tan DJ, Foong CC, Hong WH: **Mental health and self-determination profiles of the diverse population of medical students in Malaysia during the COVID-19 pandemic.** *BMC Psychol* 2022, **10:**49.

1266. Codner K, Palla B, Miloro M: **What Are the Lessons Learned From the Virtual Interview Process for Oral and Maxillofacial Surgery Residency Programs Affected by COVID-19?** *J Oral Maxillofac Surg* 2022, **80:**386-391.

1267. Coenen L, Poel LV, Schoenmakers B, Van Renterghem A, Gielis G, Remmen R, Michels NR: **The impact of COVID-19 on the well-being, education and clinical practice of general practice trainees and trainers: a national cross-sectional study.** *BMC Med Educ* 2022, **22:**108.

1268. Cole JD, Ruble MJ: **Emergent transition to remote learning: Impact on student performance for real-time comprehensive case simulations.** *Curr Pharm Teach Learn* 2022, **14:**33-37.

1269. Collis AC, Wescott AP, Greco S, Solvang N, Lee J, Morris AE: **Airborne Isolation Cardiac Arrest: A Simulation Program for Interdisciplinary Code Blue Team Training.** *MedEdPORTAL* 2022, **18:**11213.

1270. Crowe CS, Lopez J, Morrison SD, Drolet BC, Janis JE, Resident Council W, Education Study G: **The Effects of the COVID-19 Pandemic on Resident Education and Wellness: A National Survey of Plastic Surgery Residents.** *Plast Reconstr Surg* 2021, **148:**462e-474e.

1271. Cullen SE, Tiu A, Vaghela KR, Hunter AR: **A Face-to-Face Surgical Instrumentation Course During the COVID-19 Pandemic.** *Cureus* 2021, **13:**e19266.

1272. Cullinane DP, Barry DS: **Dyad pedagogy in practical anatomy: A description of the implementation and student perceptions of an adaptive approach to cadaveric teaching.** *Anat Sci Educ* 2022.

1273. Cupido M, Gordon N, Behardien N: **Module evaluation for emergency remote teaching: An oral hygiene case study during the COVID-19 pandemic.** *African Journal of Health Professions Education* 2022, **14:**17-25.

1274. Curcio F, Gonzalez CIA, Zicchi M, Sole G, Finco G, Ez Zinabi O, Melo P, Galletta M, Martinez-Riera JR: **COVID-19 Pandemic Impact on Undergraduate Nursing Students: A Cross-Sectional Study.** *Int J Environ Res Public Health* 2022, **19**.

1275. Currat L, Suppan M, Gartner BA, Daniel E, Mayoraz M, Harbarth S, Suppan L, Stuby L: **Impact of Face-to-Face Teaching in Addition to Electronic Learning on Personal Protective Equipment Doffing Proficiency in Student Paramedics: Randomized Controlled Trial.** *Int J Environ Res Public Health* 2022, **19**.

1276. Curtolo E, Szijj JV, Camilleri L, Serracino-Inglott A, Azzopardi LM: **Development, validation and evaluation of learning activities to support the reporting of adverse drug reactions during the COVID-19 pandemic.** *Pharmacy Education* 2022, **22:**515-522.

1277. Cushen-Brewster N, Barker A, Driscoll-Evans P, Wigens L, Langton H: **The experiences of adult nursing students completing a placement during the COVID-19 pandemic.** *Br J Nurs* 2021, **30:**1250-1255.

1278. Cygan H, Bejster M, Tribbia C, Vondracek H: **Impact of COVID-19 on public health nursing student learning outcomes.** *Public Health Nurs* 2022, **39:**481-487.

1279. D'Angelo AD, D'Angelo JD, Beaty JS, Cleary RK, Hoedema RE, Mathis KL, Dozois EJ, Kelley SR: **Virtual interviews - Utilizing technological affordances as a predictor of applicant confidence.** *Am J Surg* 2021, **222:**1085-1092.

1280. D'Emilio A, Sabounchi SS, Wu Y, Kim A, Franck E, Kane D, Lieberman M: **Comparison of clinical competency self-assessments among Advanced Education in General Dentistry (AEGD) residents before and during COVID-19 pandemic.** *J Dent Educ* 2022, **86:**774-780.

1281. Dabhi N, Parmar D, Mathur A, Jana I: **Perception of the medical faculty for online teaching and assessment in coronavirus disease 2019.** *National Journal of Physiology, Pharmacy and Pharmacology* 2021, **12:**749-753.

1282. Dahiya D, Banda A, Abuji K, Sharma AK, Tandup C, Behera A: **Surgical Residents' Perspective on Online Education during COVID-19 Pandemic.** *Pol Przegl Chir* 2021, **94:**1-4.

1283. G PP: **A Comparative Study of Perception of Online Teaching Versus Traditional Teaching among MBBS Students during COVID Crisis.** *Journal of Communicable Diseases* 2022**:**3-8.

1284. Demirtas CO, Vahabov C, Mustafayev F, Sahin T, Parlak E: **Impact of COVID-19 Pandemic on Gastroenterology Fellowship Training in Turkey: A Prospective Nationwide Survey Study.** *Turk J Gastroenterol* 2021, **32:**879-887.

1285. Dempsey L, Gaffney L, Bracken S, Tully A, Corcoran O, McDonnell-Naughton M, Sweeney L, McDonnell D: **Experiences of undergraduate nursing students who worked clinically during the COVID-19 pandemic.** *Nurs Open* 2022.

1286. Deng J, Que J, Wu S, Zhang Y, Liu J, Chen S, Wu Y, Gong Y, Sun S, Yuan K, et al: **Effects of COVID-19 on career and specialty choices among Chinese medical students.** *Med Educ Online* 2021, **26:**1913785.

1287. Dennis D, Cipriano L, Mulvey G, Parkinson S, Reubenson A, Furness A: **Observational Study Exploring the Efficacy and Effectiveness of a New Model of Peer-Assisted Simulation-Based Learning Clinical Placement.** *Int J Environ Res Public Health* 2022, **19**.

1288. Dhanabal N, Govindaraju P, Kumar S, Mrthyunjeya RK, Tom KT, Pradhan D: **Perception of Dental Students of Karnataka on Effectiveness of E-Learning during Covid-19 Pandemic – A Questionnaire Based Survey.** *Journal of Young Pharmacists* 2021, **13:**429-433.

1289. Domingo A, Rdesinski RE, Stenson A, Aylor M, Sullenbarger J, Hatfield J, Walker S, Hervey S, Singer J, Cois A, Cheng A: **Virtual Residency Interviews: Applicant Perceptions Regarding Virtual Interview Effectiveness, Advantages, and Barriers.** *J Grad Med Educ* 2022, **14:**224-228.

1290. El Naggar MAA MJ: **Perception of medical students regarding e-learning during covid 19 pandemic.** *Medical Journal*, **28:**640-645.

1291. Elbeshbeshy R, Gim S, Quattrocchi E: **Impact of COVID-19 on graduating U.S. pharmacy students in the early epicenter of the pandemic in New York City.** *Explor Res Clin Soc Pharm* 2021, **4:**100085.

1292. Eleftheriou A, Rokou A, Arvaniti A, Nena E, Steiropoulos P: **Sleep Quality and Mental Health of Medical Students in Greece During the COVID-19 Pandemic.** *Front Public Health* 2021, **9:**775374.

1293. Eleftheriou A, Rokou A, Doukaki P, Kapetanios I, Diamantopoulou P, Papaioannou TG, Lytras T, Papanas N, Constantinidis TC, Nena E: **Undergraduate Medical Education During the Pandemic: How do Academic Staff Members in Greece and Cyprus Feel About it?** *Int J Low Extrem Wounds* 2021**:**15347346211045285.

1294. Elghobashy M, Wahab L, Gunavardhan A, O'Sullivan E, Provenzano E, Deb R, Pritchard S, Di Palma S, Ellis IO, Boyd C, et al: **Impact of COVID-19 on the practice of breast pathologists: a survey of breast pathologists in the UK and Ireland.** *J Clin Pathol* 2021.

1295. Elkhamisy FAA, Sharif AF: **Medical students perceptions of virtual learning stations as an innovative teaching tool: a qualitative study.** *Interactive Learning Environments* 2021**:**1-17.

1296. Elmorsi R, Asaad M, Ferry AM, Rajesh A, Maricevich RS: **How real is a virtual interview? Perspectives of orthopaedic surgery residency directors.** *Eur Rev Med Pharmacol Sci* 2021, **25:**7829-7832.

1297. Elrod J, Boettcher J, Vincent D, Schwarz D, Trautmann T, Reinshagen K, Boettcher M: **Telementoring of Open and Laparoscopic Knot Tying and Suturing Is Possible: A Randomized Controlled Study.** *Eur J Pediatr Surg* 2022, **32:**443-451.

1298. Elshaer IA, Sobaih AEE: **FLOWER: An Approach for Enhancing E-Learning Experience Amid COVID-19.** *Int J Environ Res Public Health* 2022, **19**.

1299. Elshami W, Taha MH, Abuzaid M, Saravanan C, Al Kawas S, Abdalla ME: **Satisfaction with online learning in the new normal: perspective of students and faculty at medical and health sciences colleges.** *Med Educ Online* 2021, **26:**1920090.

1300. Elver AA, Braasch MC, Byer S, Gilmer L, Sykes KJ, Tuchek C, DiPasco P: **Is a Video Worth a Thousand Words? Educating Preclinical Medical Students on Sterile Scrubbing, Gowning, and Gloving Virtually and In-Person.** *Kans J Med* 2021, **14:**265-268.

1301. Elzayat S, Elfarargy HH, Mandour M, Mahrous A, El-Deeb ME, Barbara M, Elsherief H: **The Impact of COVID-19 on the Daily Life and Medical Practice of Otolaryngology Physicians.** *Int Arch Otorhinolaryngol* 2022, **26:**e478-e486.

1302. Emory J, Kippenbrock T, Buron B: **A national survey of the impact of COVID-19 on personal, academic, and work environments of nursing students.** *Nurs Outlook* 2021, **69:**1116-1125.

1303. Engel AL, Muller A, Spat H, Kurz S, Hoffmann EM: **[German national survey of lecturers in ophthalmology on student teaching in the 2020/21 corona pandemic].** *Ophthalmologie* 2022, **119:**611-618.

1304. Enujioke SC, McBrayer K, Soe KC, Imburgia TM, Robbins C: **Impact of COVID-19 on post graduate medical education and training.** *BMC Med Educ* 2021, **21:**580.

1305. Enyama D, Balti EV, Simeni Njonnou SR, Ngongang Ouankou C, Kemta Lekpa F, Noukeu Njinkui D, Fouogue JT, Mayouego Kouam J, Njateng GSS, Kenfack B, et al: **Use of WhatsApp(R), for distance teaching during COVID-19 pandemic: Experience and perception from a sub-Saharan African setting.** *BMC Med Educ* 2021, **21:**517.

1306. Falcioni AG, Yang HC, Maricic MA, Rodriguez SP, Bailez MM: **Effectiveness of telesimulation for pediatric minimally invasive surgery essential skills training.** *J Pediatr Surg* 2022, **57:**1092-1098.

1307. Farber CM, Lemos M, Said Yekta-Michael S: **Effect of an endodontic e-learning application on students' performance during their first root canal treatment on real patients: a pilot study.** *BMC Med Educ* 2022, **22:**394.

1308. Farhan S, Al-Imam A, Motyka MA: **Evaluation of Anatomy Course Teaching and Learning Outcomes for Iraqi Pharmacy Students: Internet-based Learning versus Blended Learning During the Pandemic.** *Open Access Macedonian Journal of Medical Sciences* 2021, **9:**782-788.

1309. Farsi ZA EF, Andrew Ahmadi, Yazdan Sajadi, Seyedeh Azam Aliyari, Shahla.: **INVESTIGATING NURSING STUDENTS' SATISFACTION WITH THE QUALITY OF COURSES AND VIRTUAL LEARNING DURING THE COVID-19 PANDEMIC IN 2020-2021.** *TURKISH ONLINE JOURNAL OF DISTANCE EDUCATION*, **23:**103-117.

1310. Fashner J, Espinoza A, Mainous Iii AG: **COVID-19 disruption to family medicine residency curriculum: results from a 2020 US programme directors survey.** *Fam Med Community Health* 2021, **9**.

1311. Fayngersh A, Sudyn A, Jain N, Asri R, Traba C, Matassa D, Wong K: **Learning in the Pandemic: Medical Students' Perceived Effects of COVID-19 on Their Clinical Experiences and Career Choices During the Internal Medicine Clerkship.** *Med Sci Educ* 2022, **32:**907-915.

1312. Fedoruk K, Abir G, Carvalho B: **Virtual Compared to In-Person Obstetric Anesthesiology Trainee Education During the COVID-19 Pandemic: A Short Report.** *Cureus* 2022, **14:**e26423.

1313. Feeley A, Feeley I, Carroll A, Hehir DJ: **Student Acceptance of Virtual Bedside Surgical Tutorials During COVID-19: A Randomized Controlled Trial.** *J Surg Res* 2022, **270:**261-265.

1314. Feng X, Mi K, Shen Y, Hua H, Bian Y, Bian H: **Rain Classroom assisted by WeChat for preliminary online physiology teaching during the COVID-19 pandemic.** *Adv Physiol Educ* 2022, **46:**319-324.

1315. Ferber MF, Zubatsky M, Jacobs CK, Cronholm PF: **COVID-19 Exposure Risk, Burnout, and Shifts in Family Medicine Faculty's Efforts: A National Survey.** *Fam Med* 2022, **54:**193-199.

1316. Ferris CK, Williams DM, Shen E, Jackson JM, Bruggen JT: **The GI Simulated Clinic in the Era of COVID-19: a Comparison of Virtual to In-Person Delivery.** *Med Sci Educ* 2022, **32:**75-78.

1317. Förster CE-S JS, S. Bischoff, M. Fuchs, M. Von Luckner, A. G. Ledig, T. Barzel, A. Maun, A. Joos, S. Szecsenyi, J. Schwill, S.: **How to plan and perform E-learning in family medicine vocational training lessons learned in the KWBW-verbundweiterbildungplus.** *Zeitschrift fur Allgemeinmedizin*, **97:**318-324.

1318. Forycka J, Pawlowicz-Szlarska E, Burczynska A, Cegielska N, Harendarz K, Nowicki M: **Polish medical students facing the pandemic-Assessment of resilience, well-being and burnout in the COVID-19 era.** *PLoS One* 2022, **17:**e0261652.

1319. Gali S: **Case-based Virtual Seminars on Masticatory Muscle Disorders during the COVID-19 Pandemic.** *Med Sci Educ* 2022, **32:**953-957.

1320. Gallo R, Guarneri F, Gasparini G, Oddenino G, Carmisciano L, Rovini E, Parodi A: **Implementation of a distance learning hand eczema prevention program for healthcare workers during the COVID-19 pandemic.** *Contact Dermatitis* 2022, **87:**297-300.

1321. Z. G: **How did changes in medical education during the COVID-19 pandemic affect students’ learning and study approaches?** *Annals of Clinical and Analytical Medicine* 2021, **12:**503-507.

1322. Ganji J, Shirvani MA, Motahari-Tabari N, Tayebi T: **Design, implementation and evaluation of a virtual clinical training protocol for midwifery internship in a gynecology course during COVID-19 pandemic: A semi-experimental study.** *Nurse Educ Today* 2022, **111:**105293.

1323. Garcia RR RW, H. Chambless, L. B. Lam, S. Dahdaleh, N. S. Rosseau, G. L.: **A National Survey Evaluating the Impact of the COVID-19 Pandemic on Medical Students Pursuing Careers in Neurosurgery.** *Neurosurgery*, **67:**48.

1324. Gard J, Polley C, Cisternino A, Gray A: **The void: COVID-19 restrictions and junior doctor education opportunities.** *Med Teach* 2022**:**1-6.

1325. Garg K, Mishra S, Raheja A, Verma S, Tandon V, Agrawal S, Suri A, Chandra PS, Prada F, Servadei F, et al: **Hybrid Workshops During the COVID-19 Pandemic-Dawn of a New Era in Neurosurgical Learning Platforms.** *World Neurosurg* 2022, **157:**e198-e206.

1326. Gautam S, Shukla A, Mishra N, Kohli M, Singh GP: **Effectiveness of virtual training for medical officers and community health officers in the critical care management of COVID-19 patients in the intensive care unit.** *Indian J Anaesth* 2021, **65:**S168-S173.

1327. Gavazva E, Grekova D: **Students’ perceptions and impact of the COVID-19 pandemic on the pharmaceutical education in Bulgaria: A pilot project.** *Pharmacy Education* 2022, **22:**569-572.

1328. Gazza EA: **The Experience of Being a Full-Time Academic Nurse Educator During the COVID-19 Pandemic.** *Nurs Educ Perspect* 2022, **43:**74-79.

1329. Gebril MSB MG, M. Seth, S.: **Evaluation of the Online Learning Experience of Dalhousie Dentistry and Dental Hygiene Students during COVID-19 Pandemic Outbreak.** *J Can Dent Assoc*, **87:**l18.

1330. Gehret NL, Brooks BE, Vance TM, Wambier CG, Libby TJ: **Impact of the COVID-19 pandemic on dermatology residency education in the United States: A cross-sectional survey.** *JAAD Int* 2022, **8:**134-135.

1331. Ghoneim AH, Ghoneim SH, Bairaqi SA, Yassin MY, AlAhmed AB, Aldhamen KM, Alfaraj KA, Almayyad FT: **Virtual and Traditional Learning in Undergraduate Radiology Education: A Cross-sectional Comparative Study from Saudi Arabia.** *Saudi J Med Med Sci* 2022, **10:**151-156.

1332. Giannopoulos E, Agarwal A, Croke J, Golden DW, Hirsch AE, Jimenez RB, Malik NH, Papadakos J, Quartey NK, Samoil D, et al: **Exploring the Perceived Educational Impact of COVID-19 on Postgraduate Training in Oncology.** *J Cancer Educ* 2022**:**44774.

1333. Goel DK I: **Impact of the COVID-19 pandemic: The perceptions of educators about change in medical education.** *Asian Journal of and Research*, **14:**81-83.

1334. Goldflam K, Crichton IC, Coughlin RF, Bod J, Agrawal P, Bradby C, Tsyrulnik A: **Meeting expectations: An exploration of academic emergency medicine faculty experiences and preferences in the virtual meeting environment by age, gender and parental status.** *AEM Educ Train* 2022, **6:**e10724.

1335. Goldin S, Hood N, Pascutto A, Bennett C, Barbosa de Lima AC, Devereaux N, Caric A, Rai K, Desai S, Lindstrand A, Struminger B: **Building global capacity for COVID-19 vaccination through interactive virtual learning.** *Hum Resour Health* 2022, **20:**16.

1336. Goldin S, Kong SYJ, Tokar A, Utunen H, Ndiaye N, Bahl J, Appuhamy R, Moen A: **Learning From a Massive Open Online COVID-19 Vaccination Training Experience: Survey Study.** *JMIR Public Health Surveill* 2021, **7:**e33455.

1337. Goldman C, Pradere B, Mete M, Talso M, Bernardino R, Campi R, Marchalik D: **A Multinational Study of The Impact of Covid-19 On Urologic Surgery Residency and Wellbeing.** *Urology* 2022, **166:**87-94.

1338. Gomez-Duran EL, Fumado CM, Gasso AM, Diaz S, Miranda-Mendizabal A, Forero CG, Virumbrales M: **COVID-19 Pandemic Psychological Impact and Volunteering Experience Perceptions of Medical Students after 2 Years.** *Int J Environ Res Public Health* 2022, **19**.

1339. Gong Z, Li W, Bu H, He M, Hou H, Ma T, Hu X, Fu L, Adu-Amankwaah J, Sun H: **Impact of COVID-19 pandemic on the professional intention of medical and related students.** *BMC Med Educ* 2021, **21:**484.

1340. Grady ZJ, Gallo LK, Lin HK, Magod BL, Coulthard SL, Flink BJ, Knauer EM, Winer JH, Papandria D, Pettitt BJ: **From the Operating Room to Online: Medical Student Surgery Education in the Time of COVID-19.** *J Surg Res* 2022, **270:**145-150.

1341. Grafton-Clarke C, Uraiby H, Abraham S, Kirtley J, Xu G, McCarthy M: **Live streaming to sustain clinical learning.** *Clin Teach* 2022, **19:**282-288.

1342. Grand D, Schuster VL, Pullman JM, Golestaneh L, Raff AC: **Medical Student Experience and Outcomes, as Well as Preceptor Experience, with Rapid Conversion of a Preclinical Medical School Course to a Remote-Based Learning Format in the Setting of the COVID-19 Pandemic.** *Med Sci Educ* 2021, **31:**1895-1901.

1343. Greb AC, Altieri E, Masini I, Frisch EH, Greenberg ML: **Educational impact of an active learning session with 6-lead mobile electrocardiography on medical students' knowledge of cardiovascular physiology during the COVID-19 pandemic in the United States: a survey-based observational study.** *J Educ Eval Health Prof* 2022, **19:**12.

1344. Grewal S, Houston A, Bacon J, Balderama E, Elhassan MG: **Point-of-Care Ultrasound Curriculum for Internal Medicine Residents During the COVID-19 Era: A Pilot Study.** *Cureus* 2022, **14:**e25944.

1345. Grover S, Pandya M, Ranasinghe C, Ramji SP, Bola H, Raj S: **Assessing the utility of virtual OSCE sessions as an educational tool: a national pilot study.** *BMC Medical Education* 2022, **22:**178.

1346. Gruba G, Kasiak PS, Gebarowska J, Adamczyk N, Sikora Z, Jodczyk AM, Mamcarz A, Sliz D: **PaLS Study of Sleep Deprivation and Mental Health Consequences of the COVID-19 Pandemic among University Students: A Cross-Sectional Survey.** *Int J Environ Res Public Health* 2021, **18**.

1347. Galvis Navarrete SH, Grueso Angulo R, Torregrosa Almonacid L: **Percepción de la experiencia académica teórica de los residentes de especialidades médico-quirúrgicas durante la pandemia por COVID-19 en un hospital universitario de alta complejidad.** *Universitas Médica* 2021, **62**.

1348. Gul U, Altuntas D, Efe E: **A year and a half later: Clinical experiences of intern nursing students in the COVID-19 Pandemic: A constructivist grounded theory.** *Nurse Educ Pract* 2022, **63:**103381.

1349. Gumede L, Badriparsad N: **Online teaching and learning through the students' eyes - Uncertainty through the COVID-19 lockdown: A qualitative case study in Gauteng province, South Africa.** *Radiography (Lond)* 2022, **28:**193-198.

1350. Gummerson CE, Lo BD, Porosnicu Rodriguez KA, Cosner ZL, Hardenbergh D, Bongiorno DM, Wainger J, Hu K, Gamaldo C, Salas RME, et al: **Broadening learning communities during COVID-19: developing a curricular framework for telemedicine education in neurology.** *BMC Med Educ* 2021, **21:**549.

1351. Gunasekera A, Berg L, Sekar H, Patra-Das S, Clarke S, Yoong W: **Did the COVID-19 pandemic affect mental health, training progression, and fertility planning of obstetrics and gynecology trainees? A survey of London trainees.** *J Obstet Gynaecol Res* 2022, **48:**1026-1032.

1352. Gunner CK, Eisner E, Watson AJ, Duncan JL: **Teaching webside manner: development and initial evaluation of a video consultation skills training module for undergraduate medical students.** *Med Educ Online* 2021, **26:**1954492.

1353. Gupta B, Jain G, Pathak S, Mishra P, Kumar H, Rao S: **Airway management training program for nurses via online course in COVID-19 preparedness.** *World J Methodol* 2022, **12:**113-121.

1354. Gupta S, Grier Arthur L, Chandler N, Danielson P, Downard C, Ehrlich P, Gaines B, Gray B, Javid P, Lallier M, et al: **Is the changing landscape of fellowship recruitment during COVID-19 here to stay?** *J Pediatr Surg* 2022, **57:**445-450.

1355. Guse J, Heinen I, Mohr S, Bergelt C: **Understanding Mental Burden and Factors Associated With Study Worries Among Undergraduate Medical Students During the COVID-19 Pandemic.** *Front Psychol* 2021, **12:**734264.

1356. Guse J, Weegen AS, Heinen I, Bergelt C: **Mental burden and perception of the study situation among undergraduate students during the COVID-19 pandemic: a cross-sectional study and comparison of dental and medical students.** *BMJ Open* 2021, **11:**e054728.

1357. Hadie SNH, Tan VPS, Omar N, Nik Mohd Alwi NA, Lim HL, Ku Marsilla KI: **COVID-19 Disruptions in Health Professional Education: Use of Cognitive Load Theory on Students' Comprehension, Cognitive Load, Engagement, and Motivation.** *Front Med (Lausanne)* 2021, **8:**739238.

1358. Hafeez S, Rizwan AS, Ali T, Hashmi IB, Tahir M, Wazir ZK: **COVID-19 Pandemic: Psychological Impact on Postgraduate Gynae Residents in Pakistan.** *Pakistan Journal of Medical and Health Sciences* 2021, **15:**2210-2212.

1359. Hafner S, Zolk O, Barth H: **COVID-19 pandemic-related adaptations of medical education in clinical pharmacology - impact on students and lecturers at a German university.** *Naunyn Schmiedebergs Arch Pharmacol* 2022, **395:**681-690.

1360. Haisley KR, Renshaw SM, Needleman BJ, Narula VK, Poulose BK, Perry KA: **Virtual Interviews for Surgical Fellowship are an Acceptable Alternative to In-Person Interviews for Applicants and Faculty Alike.** *Surgical Innovation* 2022**:**1.55E+16.

1361. Ahmad A, Arif JM, Alobaidi AM, Alanazi AR, Alruwaili SH, Aldhafeeri FS, Ibrahim M, Alrashed FA, Sultan B, Alashhab M, et al: **Impact of online learning on medical and non-medical students during COVID-19 pandemic in Shaqra University Saudi Arabia: A comparative study.** *Medical Science* 2022, **26**.

1362. Hammoudi Halat D, Safwan J, Akel M, Rahal M: **Pharmacy education shift during times of pandemic and collapse: A perspective from a school of pharmacy in Lebanon.** *Pharmacy Education* 2022, **22:**33-40.

1363. Hall AJ, Walmsley P: **Technology-enhanced learning in orthopaedics: Virtual reality and multi-modality educational workshops may be effective in the training of surgeons and operating department staff.** *Surgeon* 2022.

1364. Hamad HA, Sunni ZA, AlMohsin F, AlMaimouni Y, Khabeer A, Nazir MA, Farooq I: **An Assessment of Various Challenges Perceived by Dental Students amidst the COVID-19 Pandemic: A Digital Questionnaire Study.** *Behav Sci (Basel)* 2022, **12**.

1365. Hamid M, Siddiqui Z, Aslam Joiya S: **Recovery of Surgical Training Through Extended Laparoscopic Simulation Training.** *Cureus* 2021, **13:**e18695.

1366. Hamid MH, Mazher B, Aslam S, Shirin A, Javed T: **Impact of COVID-19 pandemic on postgraduate training in Paediatrics.** *J Pak Med Assoc* 2022, **72:**912-915.

1367. Han B, Zang F, Liu J, Li S, Zhang W, Zhang Y, Li Z: **Effect Analysis of "Four-Step" Training and Assessment Tool in the Prevention and Control of COVID-19.** *Infect Drug Resist* 2022, **15:**1247-1257.

1368. Han JW, Joung J, Kang JS, Lee H: **A Study of the Educational Needs of Clinical Nurses Based on the Experiences in Training Programs for Nursing COVID-19 Patients.** *Asian Nurs Res (Korean Soc Nurs Sci)* 2022, **16:**63-72.

1369. Han R, Hahn E, Done SJ, Pun C, Shivji S, Lu FI: **Resident Depression and Burnout During the COVID-19 Pandemic.** *Arch Pathol Lab Med* 2022.

1370. Haque A, Naveed A, Ashraf MI, Ghassan A, Mirza TI: **Perceptions of faculty teaching anatomy online during COVID-19, A Qualitative study.** *Pakistan Journal of Medical and Health Sciences* 2022, **16:**209-211.

1371. Harrell Shreckengost CS, Reitz A, Ludi E, Rojas Aban R, Jauregui Paravicini L, Serrot F: **Lessons learned during the COVID-19 pandemic using virtual basic laparoscopic training in Santa Cruz de la Sierra, Bolivia: effects on confidence, knowledge, and skill.** *Surg Endosc* 2022**:**44866.

1372. Hasanpour M, Maroufizadeh S, Mousavi H, Noughani F, Afshari M: **Prevalence of generalized anxiety disorder among nursing students in Iran during the COVID-19 pandemic: A web-based cross-sectional study.** *Int J Afr Nurs Sci* 2021, **15:**100360.

1373. Haskett LA, Doster DL, Athanasiadis DI, Anton NE, Huffman EK, Wallach P, Walvoord E, Stefanidis D, Mitchell SA, Lee NK: **Resilience matters: Student perceptions of the impact of COVID-19 on medical education.** *Am J Surg* 2022, **224:**358-362.

1374. Hassan EA, Elsaman SEA: **The effect of simulation-based flipped classroom on acquisition of cardiopulmonary resuscitation skills: A simulation-based randomized trial.** *Nurs Crit Care* 2022.

1375. Hassan R, Khalifa AR, Elsewify T, Hassan MG: **Perceptions of Clinical Dental Students Toward Online Education During the COVID-19 Crisis: An Egyptian Multicenter Cross-Sectional Survey.** *Front Psychol* 2021, **12:**704179.

1376. Hassler LJ, Moscarella D, Easley L, Olaode S: **Flipped Clinical Teaching: Battling COVID-19 With Creative and Active Pedagogy.** *J Nurs Educ* 2021, **60:**534-537.

1377. Hassnain S, Ahmad A, Qayyum MS, Farrukh MG, Nawaz UA, Ahmad H: **Effects of COVID-19 Lockdown on mental health of medical students in Lahore, Pakistan.** *Bangladesh Journal of Medical Science* 2021, **20:**125-130.

1378. Hatcher TM, Schoen RR, Garmong GE, Stewart-Lynch AL: **Student Pharmacist Perspectives of a Remote Ambulatory Care and Community Pharmacy Dual-Cohort APPE.** *J Pharm Pract* 2022**:**8971900221107836.

1379. Haugh M, O'Tuathaigh C: **Adapting for sustainability: Ensuring provision of research skills development for undergraduate medical students.** *Clin Teach* 2022, **19:**86-91.

1380. Hawkins S, Fogg N, Wilson C, Browne J: **Establishing a tutoring and academic support center: Collaborating with nurse educator students.** *J Prof Nurs* 2022, **39:**19-25.

1381. Hayashi M, Nishiya K, Kaneko K: **Transition from undergraduates to residents: A SWOT analysis of the expectations and concerns of Japanese medical graduates during the COVID-19 pandemic.** *PLoS One* 2022, **17:**e0266284.

1382. Hayashi M, Saiki T, Kanter SL, Ho MJ: **Leaders' perspectives and actions to manage challenges in medical education presented by the COVID-19 pandemic: a nationwide survey of Japanese medical colleges.** *BMC Med Educ* 2022, **22:**144.

1383. Hayes C, Mears M, Rowan S, Dong F, Andrews E: **Academic performance and attitudes of dental students impacted by COVID-19.** *J Dent Educ* 2022, **86:**874-882.

1384. He M, Tang XQ, Zhang HN, Luo YY, Tang ZC, Gao SG: **Remote clinical training practice in the neurology internship during the COVID-19 pandemic.** *Med Educ Online* 2021, **26:**1899642.

1385. Head ML, Acosta S, Bickford EG, Leatherland MA: **Impact of COVID-19 on Undergraduate Nursing Education: Student Perspectives.** *Acad Med* 2022, **97:**S49-S54.

1386. Heaps BM, Dugas JR, Limpisvasti O: **The Impact of COVID-19 on Orthopedic Surgery Fellowship Training: A Survey of Fellowship Program Directors.** *HSS J* 2022, **18:**105-109.

1387. Heimbach M, Holzmann K, Stein P, Stief L, Berberat PO, Dirmeier M: **How to... train your skills goes digital! A project report on the development and implementation of practice-oriented digital student tutorials.** *GMS J Med Educ* 2022, **39:**Doc5.

1388. Heitmann H, Wagner P, Fischer E, Gartmeier M, Schmidt-Graf F: **Effectiveness of non-bedside teaching during the COVID-19 pandemic: a quasi-experimental study.** *BMC Med Educ* 2022, **22:**73.

1389. Helland HK, Tylleskar T, Kvernenes M, Reikvam H: **An Abrupt Transition to Digital Teaching-Norwegian Medical Students and Their Experiences of Learning Output during the Initial Phase of the COVID-19 Lockdown.** *Healthcare (Basel)* 2022, **10**.

1390. Helm MF, Kimball AB, Butt M, Stuckey H, Costigan H, Shinkai K, Nagler AR: **Challenges for dermatologists during the COVID-19 pandemic: A qualitative study.** *Int J Womens Dermatol* 2022, **8:**e013.

1391. Henschen BL, Jasti H, Kisielewski M, Pincavage AT, Levine D: **Teaching Telemedicine in the COVID-19 Era: a National Survey of Internal Medicine Clerkship Directors.** *J Gen Intern Med* 2021, **36:**3497-3502.

1392. Heo S, Moon S, Kim M, Park M, Cha WC, Son MH: **An Augmented Reality-Based Guide for Mechanical Ventilator Setup: Prospective Randomized Pilot Trial.** *JMIR Serious Games* 2022, **10:**e38433.

1393. Herbert A, Russell FM, Zahn G, Zakeri B, Motzkus C, Wallach PM, Ferre RM: **Point-of-Care Ultrasound Education During a Pandemic: From Webinar to Progressive Dinner-Style Bedside Learning.** *Cureus* 2022, **14:**e25141.

1394. Herbstreit S, Gestmann M, Szalai C, Diehl A: **Are medical students equipped for digital studies?Have their hopes and fears been confirmed during Covid-19? What should we consider in the future?** *J Eur CME* 2021, **10:**2014098.

1395. Hernandez T, Fallar R, Polydorides AD: **Outcomes of Remote Pathology Instruction in Student Performance and Course Evaluation.** *Acad Pathol* 2021, **8:**23742895211061822.

1396. Herr L, Jih MK, Shin J, Chae YK, Lee HS, Choi SC, Nam OH: **The perspective of undergraduate dental students on web-based learning in pediatric dentistry during the COVID-19 pandemic: a Korean multicenter cross-sectional survey.** *BMC Med Educ* 2021, **21:**505.

1397. Hertling S, Hertling D, Matziolis G, Schleussner E, Loos F, Graul I: **Digital teaching tools in sports medicine: A randomized control trial comparing the effectiveness of virtual seminar and virtual fishbowl teaching method in medical students.** *PLoS One* 2022, **17:**e0267144.

1398. Hertrampf K, Wenz HJ, Kaduszkiewicz H, Goetz K: **Suspension of face-to-face teaching and ad hoc transition to digital learning under Covid-19 conditions - a qualitative study among dental students and lecturers.** *BMC Med Educ* 2022, **22:**257.

1399. Hey HWD, Tay HW, Bosco A, Soh RCC, Oh JY: **Impact of COVID-19 on orthopaedic specialist training: a nationwide survey of orthopaedic residents in Singapore.** *Singapore Med J* 2022.

1400. Hickey K, Thorburn C, Wilson J, Pace D, Hogan M, Quigley S: **The impact of COVID-19 on surgical education: perspectives from Canadian general surgery residents.** *Can J Surg* 2021, **64:**E613-E614.

1401. Hjiej G, Idrissi FEE, Janfi T, Bouhabs M, Hnaifi H, Belakbyer H, Gabri M, Touissi Y, Hajjioui A, Bentata Y, et al: **Distant education in Moroccan medical schools following COVID-19 outbreak at the early phase of lockdown: Were the students really engaged?** *Sci Afr* 2022, **15:**e01087.

1402. Ho G, Davis J, Hindle AK, Heinz E: **Virtual Residency Interviews: A Survey of Anesthesiology Program Director Perspectives Amidst the COVID-19 Pandemic.** *J Educ Perioper Med* 2021, **23:**E674.

1403. Hoang H, Jessup B, Obamiro K, Bourke L, Hellwege B, Podubinski T, Heaney S, Sheepway L, Farthing A, Rasiah R, et al: **Impact of COVID-19 on rural and remote student placements in Australia: A national study.** *Aust J Rural Health* 2022, **30:**197-207.

1404. Hodges A, Powell S, Williams S: **An Evaluation of Audiovisual Discussion Boards in a Graduate-Level Nursing Course: Innovation During the Pandemic.** *Nurs Educ Perspect* 2022.

1405. Hofstadter-Thalmann E, Rotgans JI, Aybar Perez N, Nordquist J: **Effective Learning in Virtual Conferences: The Application of Five Principles of Learning.** *J Eur CME* 2022, **11:**2019435.

1406. Holm D, Ohr SO, Giles M: **The experiences of new graduate nurses and midwives going through a virtual interview recruitment process during the COVID-19 crisis: a cross-sectional study.** *Hum Resour Health* 2021, **19:**121.

1407. Holm P, Beckman L: **Flipped or traditional online teaching? Two different strategies to handle teaching in nursing education during the COVID-19 pandemic.** *Int J Nurs Educ Scholarsh* 2022, **19**.

1408. Holzmann-Littig C, Zerban NL, Storm C, Ulhaas L, Pfeiffer M, Kotz A, Wijnen-Meijer M, Keil S, Huber J: **One academic year under COVID-19 conditions: two multicenter cross-sectional evaluation studies among medical students in Bavarian medical schools, Germany students' needs, difficulties, and concerns about digital teaching and learning.** *BMC Med Educ* 2022, **22:**450.

1409. Hong BE, Myo Bui CC, Huang YM, Grogan T, Duval VF, Cannesson M: **Implementing COVID-19 Simulation Training for Anesthesiology Residents.** *MedEdPORTAL* 2022, **18:**11215.

1410. Hope DL, Grant GD, Rogers GD, King MA: **Virtualized Gamified Pharmacy Simulation during COVID-19.** *Pharmacy (Basel)* 2022, **10**.

1411. Masoumian Hosseini T, Ahmady S, Edelbring S: **Teaching Clinical Decision-Making Skills to Undergraduate Nursing Students via Web-based Virtual Patients during the COVID-19 Pandemic: A New Approach to The CyberPatientTM Simulator.** *Journal of Contemporary Medical Sciences* 2022, **8:**31-37.

1412. Houdmont J, Daliya P, Theophilidou E, Adiamah A, Hassard J, Lobo DN, East Midlands Surgical Academic Network Burnout Study G: **Burnout Among Surgeons in the UK During the COVID-19 Pandemic: A Cohort Study.** *World J Surg* 2022, **46:**1-9.

1413. Houle SKD, Avdiaj B, Moroz SE, Waite NM: **Experiential education supervisors as assessors of pharmacy student injection technique.** *Curr Pharm Teach Learn* 2022, **14:**372-378.

1414. House SK, Sweet S: **Adapting NCLEX-RN remediation during the COVID-19 pandemic.** *Teach Learn Nurs* 2022, **17:**237-239.

1415. Hrelic DA, Anderson JG: **Managing the unexpected: Stressors and solutions for challenges experienced by RN-BSN students during an unprecedented global pandemic.** *J Prof Nurs* 2022, **40:**48-56.

1416. Hsieh P-L, Yang S-Y, Lin W-Y, Huang T-C: **Facilitated virtual learning for advanced geriatric education among nursing students during the COVID pandemic in Taiwan.** *Library Hi Tech* 2022.

1417. Hsu JL, Bonura EM, Blyth DM, Lindholm DA, Yun HC, Barsoumian AE: **The Impact of Coronavirus Disease 2019 on Medical Trainee Career Decisions.** *Open Forum Infect Dis* 2021, **8:**ofab523.

1418. Hsu YM, Chang TS, Chu CL, Hung SW, Wu CJ, Yeh TP, Wang JY: **Effectiveness of Multimedia-Based Learning on the Improvement of Knowledge, Attitude, and Behavioral Intention toward COVID-19 Prevention among Nurse Aides in Taiwan: A Parallel-Interventional Study.** *Healthcare (Basel)* 2022, **10**.

1419. Hu AE, Fontaine R, Turcios-Ruiz R, Abedi AA, Williams S, Hilmers A, Njoh E, Bell E, Reddy C, Ijaz K, Baggett HC: **Field epidemiology training programs contribute to COVID-19 preparedness and response globally.** *BMC Public Health* 2022, **22:**63.

1420. Hu H, Lai X, Yan L: **Improving Nursing Students' COVID-19 Knowledge Using a Serious Game.** *Comput Inform Nurs* 2021, **40:**285-289.

1421. Hu Y, Ow Yong JQY, Chng M-LC, Li Z, Goh Y-S: **Exploring undergraduate nursing students’ experiences towards home-based learning as pedagogy during the COVID-19 pandemic: a descriptive qualitative exploration.** *BMC Nursing* 2022, **21:**13.

1422. Huang LY, McKenty N, Alvarez A, Gober J, Irwin R, Molinares D, Price C, Sherman A, Tiu T, Gater DR, Jr.: **Virtually Possible: Medical Student Rehabilitation Rotations During a Pandemic.** *Am J Phys Med Rehabil* 2021, **100:**831-836.

1423. Huang SF, Hsu CW, Lin CL, Ko YL, Su HC: **Cross-Sectional Analysis of Taiwanese Pharmacy Students' Experiences and Perceptions of Transitioning from In-Hospital Internships to Distance Learning Due to COVID-19.** *Healthcare (Basel)* 2022, **10**.

1424. Huang YB, Lin YR, Hung SK, Chang YC, Ng CJ, Chen SY: **Pediatric Training Crisis of Emergency Medicine Residency during the COVID-19 Pandemic.** *Children (Basel)* 2022, **9**.

1425. Huber TS, Brown KR, Lee JT, Barry CL, Ibanez B, Jones AT, Perler BA, Upchurch GR, Jr., Vascular Surgery B, Staff: **Implementation of the Vascular Surgery Board virtual certifying examination.** *J Vasc Surg* 2022, **76:**1398-1404 e1394.

1426. Huded CB, Rasquinha SL, Rao P: **Psychological impact of COVID-19 on medical interns - Findings from a nationwide survey.** *J Educ Health Promot* 2021, **10:**336.

1427. Hulke SM, Wakode SL, Thakare AE, Parashar R, Bharshnakar RN, Joshi A, Vaidya YP: **Perception of e-learning in medical students and faculty during COVID time: A study based on a questionnaire-based survey.** *J Educ Health Promot* 2022, **11:**139.

1428. Hunck S, Engelhard K, Mildenberger P, Kurz S: **[Chances and challenges of increasing digitalization of teaching in the discipline anesthesiology from the perspective of students].** *Anaesthesiologie* 2022, **71:**689-696.

1429. Huppert LA, Hsu G, Elnachef N, Flint L, Frank JA, Gensler LS, Hsiao EC, Khanna RR, Qasim A, Schwartz BS, et al: **A single center evaluation of applicant experiences in virtual interviews across eight internal medicine subspecialty fellowship programs.** *Medical Education Online* 2021, **26:**1946237.

1430. Hussain M, Khalique A, Kumar P, Hassan AS, Hashmi A, Rizvi AUH: **E-learning in postgraduate urology training: A Covid-19 pandemic experience.** *J Pak Med Assoc* 2021, **71:**2799-2801.

1431. Hutchison CE, Reminick JI, Love ER, Karan S, Gundle KR: **Orthopaedic Surgery Residency Program Adherence to Universal Interview Offer Day Guidelines: A Retrospective Analysis.** *J Am Acad Orthop Surg* 2022, **30:**e929-e938.

1432. Hwang GJ, Chang CY, Ogata H: **The effectiveness of the virtual patient-based social learning approach in undergraduate nursing education: A quasi-experimental study.** *Nurse Educ Today* 2022, **108:**105164.

1433. Hwang SM, Rice A, Toy S, Levine R, Goeddel L: **Feasibility Study of a Fully Synchronous Virtual Critical Care Elective Focused on Learner Engagement.** *Cureus* 2022, **14:**e25427.

1434. Hwang WJ, Lee J: **Effectiveness of the Infectious Disease (COVID-19) Simulation Module Program on Nursing Students: Disaster Nursing Scenarios.** *J Korean Acad Nurs* 2021, **51:**648-660.

1435. Idriss S, Aldhuhayyan A, Alanazi AA, Alasaadi W, Alharbi R, Alshahwan G, Baitalmal M, Alonazi W: **Physicians' Perceptions of Telemedicine Use During the COVID-19 Pandemic in Riyadh, Saudi Arabia: Cross-sectional Study.** *JMIR Form Res* 2022, **6:**e36029.

1436. Ienghong K, Cheung LW, Tiamkao S, Bhudhisawasdi V, Apiratwarakul K: **Development and Remodeling of Point-of-Care Ultrasound Education for Emergency Medicine Residents in Resource Limited Countries during the COVID-19 Pandemic.** *Tomography* 2021, **7:**721-733.

1437. Ifrah Naaz S, Hussein RM, Khan HB, Hussein MM, Arain SA: **Emotional responses and coping strategies of medical students during the COVID-19 pandemic.** *Saudi Med J* 2022, **43:**61-66.

1438. Iheanacho T, Stefanovics E, Okoro UG, Anyaehie UE, Njoku PO, Adimekwe AI, Ibediro K, Stefanovics GA, Haeny A, Jackson A, et al: **Assessing knowledge, attitude, practice and training related to COVID-19: a cross-sectional survey of frontline healthcare workers in Nigeria.** *BMJ Open* 2021, **11:**e050138.

1439. Iheduru-Anderson K, Foley JA: **Transitioning to Full Online Teaching During Covid-19 Crisis: The Associate Degree Nurse Faculty Experience.** *Glob Qual Nurs Res* 2021, **8:**23333936211057545.

1440. Iheduru-Anderson KC, Foley JA: **Prelicensure Nursing Students' Reflections on Institutional Response to the 2020 COVID-19 Crisis.** *Glob Qual Nurs Res* 2022, **9:**23333936221106793.

1441. Ilic J, Radovic K, Savic-Stankovic T, Popovac A, Miletic V, Milic Lemic A: **The effect of COVID-19 pandemic on final year dental students' self-confidence level in performing clinical procedures.** *PLoS One* 2021, **16:**e0257359.

1442. Inclan PM, Woiczik MR, Cummings J, Goodwin R, Hosseinzadeh P: **Virtual Pediatric Orthopaedic Fellowship Interviews During the Pandemic: What Did the Applicants and Programs Think?** *J Pediatr Orthop* 2022, **42:**e806-e810.

1443. Inoue N, Aldosari M, Park SE, Ohyama H: **The impact of COVID-19 pandemic on student performance and self-evaluation in preclinical operative dentistry.** *Eur J Dent Educ* 2022, **26:**377-383.

1444. Instrum RS, Koch RW, Rocha T, Rohani SA, Ladak H, Agrawal SK, Sowerby LJ: **Improving Nasopharyngeal Swab Technique via Simulation for Frontline Workers.** *Laryngoscope* 2022.

1445. Iqbal A, Ganji KK, Khattak O, Shrivastava D, Srivastava KC, Arjumand B, AlSharari T, Alqahtani AMA, Hamza MO, AbdelrahmanDafaalla A: **Enhancement of Skill Competencies in Operative Dentistry Using Procedure-Specific Educational Videos (E-Learning Tools) Post-COVID-19 Era-A Randomized Controlled Trial.** *Int J Environ Res Public Health* 2022, **19**.

1446. Iqbal EJ, Sutton T, Akther MS, Samhan A, MacDonald S, Coleman JR, Turner PL, Nikolian VC: **Current Surgical Trainee Perceptions and Experiences in Telehealth.** *Telemed J E Health* 2022, **28:**789-797.

1447. Iqbal S, Hanif R, Ali F, Tahir M, Minhas R, Yasmeen R, Khokhar A, Laique T: **Teachers' Perceptions of Netiquette Practices by undergraduate Dental Students During Online Classes in Covid-19 Pandemic.** *Pakistan Journal of Medical and Health Sciences* 2021, **15:**3498-3500.

1448. Iravani M, Nasab MB, Bahmaei H, Ghanbari S, Mohaghegh Z, Siahkal SF: **The level of satisfaction and quality of E-learning in medical universities of Iran during the epidemic of COVID-19.** *J Educ Health Promot* 2022, **11:**9.

1449. Islam MI, Jahan SS, Chowdhury MTH, Isha SN, Saha AK, Nath SK, Jahan MS, Kabir MH, Hoque Apu E, Kabir R, Siddika N: **Experience of Bangladeshi Dental Students towards Online Learning during the COVID-19 Pandemic: A Web-Based Cross-Sectional Study.** *Int J Environ Res Public Health* 2022, **19**.

1450. Ismail F, Yelverton C, Rademan R, Peterson C: **Perceptions and attitudes of University of Johannesburg chiropractic students toward a blended learning approach and a shift to an e-learning approach necessitated by the COVID-19 pandemic.** *J Chiropr Educ* 2022, **36:**73-81.

1451. Ismail N, Tajjudin AI, Jaafar H, Nik Jaafar NR, Baharudin A, Ibrahim N: **The Relationship between Internet Addiction, Internet Gaming and Anxiety among Medical Students in a Malaysian Public University during COVID-19 Pandemic.** *Int J Environ Res Public Health* 2021, **18**.

1452. Issa MR, Awanis Muslim N, Mat Sharif Z: **The Mediating Effect of Model-Based Learning on Attitude and Pain Management Awareness Among Nurses During COVID 19 Pandemic in Saudi Arabia.** *SAGE Open Nurs* 2021, **7:**23779608211065213.

1453. Issa N, Liddy WE, Samant S, Conley DB, Kern RC, Hungness ES, Cohen ER, Barsuk JH: **Effectiveness of a simulation-based mastery learning to train clinicians on a novel cricothyrotomy procedure at an academic medical centre during a pandemic: a quasi-experimental cohort study.** *BMJ Open* 2021, **11:**e054746.

1454. Itaya LE, Heydari A, Sands WC, Okamura MY, Creasey J, Wong A: **Virtual Interview Day: An AEGD's approach and assessment of interviewing during a pandemic.** *J Dent Educ* 2022.

1455. Iwai Y, Lenze NR, Mihalic AP, Becnel CM, Stitzenberg KB: **Effect of the COVID-19 pandemic on the residency match among surgical specialties.** *Surgery* 2022, **171:**1512-1518.

1456. Jabbour Z, Tran M: **Can students develop clinical competency in treatment planning remotely through flipped collaborative case discussion?** *Eur J Dent Educ* 2022.

1457. Jackson P, Siddharthan T, Cordoba Torres IT, Green BA, Policard CJ, Degraff J, Padalkar R, Logothetis KB, Gold JA, Fort AC: **Developing and Implementing Noninvasive Ventilator Training in Haiti during the COVID-19 Pandemic.** *ATS Sch* 2022, **3:**112-124.

1458. Jackson R, Brotherston D, Jain A, Doufle G, Piquette D, Goffi A: **Teaching Ultrasound at the Point of Care in Times of Social Distancing.** *ATS Sch* 2021, **2:**341-352.

1459. Jadhav SV, Bharambe VK, Pathak VS, Khurjekar AP, Navandar RL, K AV: **A Novel Online Dissection Course on Lower Limb Anatomy During the COVID-19 Pandemic.** *Cureus* 2022, **14:**e23081.

1460. Jafree SR, Zakar R, Rafiq N, Javed A, Durrani RR, Burhan SK, Hasnain Nadir SM, Ali F, Shahid A, Momina AU, et al: **WhatsApp-Delivered Intervention for Continued Learning for Nurses in Pakistan During the COVID-19 Pandemic: Results of a Randomized-Controlled Trial.** *Front Public Health* 2022, **10:**739761.

1461. Jain S, Kalra R, Goswami P, Mani P: **Impact of Online Learning during COVID-19 Pandemic and its Comparison with Conventional Teaching: A Cross-sectional Study.** *Journal of Clinical and Diagnostic Research* 2021, **15:**FC6-FC10.

1462. Heethal J: **Flipped Classroom for Pharmacology Teaching in a Malaysian Medical School using Online Tools during the COVID-19 Pandemic: Knowledge Gained and Student Perception.** *International Journal of Online and Biomedical Engineering (iJOE)* 2022, **18:**154-161.

1463. Jami M, Xu AL, Zhang B, Durand WM, Musharbash FN, Babu JM: **Applying to Fellowship During a Pandemic: Lessons Learned From the 2020-2021 Orthopaedic Spine Fellowship Application Cycle.** *Cureus* 2022, **14:**e22630.

1464. Jamil N, I AW, Jamludin NA, Ahmad Hisham S: **Development and Conduct of Tele-Objective Structured Clinical Examination (Tele-OSCE) to Assess Clinical Pharmacy Competencies During COVID-19 Emergency Remote Teaching.** *Med Sci Educ* 2022, **32:**321-327.

1465. Jan BL: **Knowledge and Attitude of Pharmacy Students Towards Covid-19 Pandemic and Its Impact on Their Education and Learning.** *Farmacia* 2022, **70:**372-378.

1466. Jana I, Mathur A, Parmar D, Dabhi N: **Perception of medical students for online learning and assessment during the COVID era.** *National Journal of Physiology, Pharmacy and Pharmacology* 2021, **12:**744-748.

1467. Jana S, Yadav SK, Sharma D, Agarwal P: **A Low-cost model of breast biopsy for the training of surgical residents during COVID-19 pandemic.** *Trop Doct* 2022, **52:**107-109.

1468. Jarden RJ, Jarden AJ, Weiland TJ, Taylor G, Brockenshire N, Rutherford M, Carbery C, Moroney K, Joshanloo M, Gerdtz M: **Nurses' well‐being during the coronavirus (2019) pandemic: A longitudinal mixed‐methods study.** *Nursing Open* 2022.

1469. Jardon C, Choi KR: **COVID-19 Experiences and Mental Health Among Graduate and Undergraduate Nursing Students in Los Angeles.** *J Am Psychiatr Nurses Assoc* 2022**:**10783903211072222.

1470. Javier GG, Paloma SM, Andrea SC, Octavio CPP, Miguel Angel DM: **Impact of COVID-19 pandemic in surgical training of Junior Residents in general surgery.** *Heliyon* 2022, **8:**e09740.

1471. Jawed K, Sadiq T, Rashid A, Khan RA, Yasmin R: **Exploring the Experiences of Undergraduate Medical Students with Online Open-Book Exams: An Exploratory Qualitative Study.** *Pakistan Journal of Medical and Health Sciences* 2022, **16:**131-136.

1472. Jembai JVJ, Wong YLC, Bakhtiar N, Lazim SNM, Ling HS, Kuan PX, Chua PF: **Mobile health applications: awareness, attitudes, and practices among medical students in Malaysia.** *BMC Med Educ* 2022, **22:**544.

1473. Jeong Y, Lee H, Han JW: **Development and evaluation of virtual reality simulation education based on coronavirus disease 2019 scenario for nursing students: A pilot study.** *Nurs Open* 2022, **9:**1066-1076.

1474. Jiang X, Ning Q: **Evaluation and perception of online teaching of molecular biology using DingTalk for international medical students during the COVID-19 pandemic.** *Biochem Mol Biol Educ* 2022, **50:**494-501.

1475. Jimenez-Rodriguez D, Arrogante O, Gimenez-Fernandez M, Gomez-Diaz M, Guerrero Mojica N, Morales-Moreno I: **Satisfaction and Beliefs on Gender-Based Violence: A Training Program of Mexican Nursing Students Based on Simulated Video Consultations during the COVID-19 Pandemic.** *Int J Environ Res Public Health* 2021, **18**.

1476. Jimenez-Rodriguez D, Perez-Heredia M, Molero Jurado MDM, Perez-Fuentes MDC, Arrogante O: **Improving Humanization Skills through Simulation-Based Computers Using Simulated Nursing Video Consultations.** *Healthcare (Basel)* 2021, **10**.

1477. Johns G, Waddington L, Samuel V: **Prevalence and predictors of mental health outcomes in UK doctors and final year medical students during the COVID-19 pandemic.** *J Affect Disord* 2022, **311:**267-275.

1478. Johnstone J, Gabara A, Hopman WM, Touma NJ: **Impact of the COVID-19 pandemic on the burnout rates of graduating Canadian Urology residents.** *BJU Int* 2022, **130:**389-393.

1479. Joji RM, Kumar AP, Almarabheh A, Dar FK, Deifalla AH, Tayem Y, Ismaeel AY, Bindayna K, Tabbara KS, Farid E, et al: **Perception of online and face to face microbiology laboratory sessions among medical students and faculty at Arabian Gulf University: a mixed method study.** *BMC Med Educ* 2022, **22:**411.

1480. Jones HM, Ankem A, Seroogy EA, Kalantar A, Goldsmith DC, Rizenbergs KC, Van Meter TL: **Impact of COVID-19 on Radiology Residency Selection Process: A Survey of Radiology Residency Programs in the US.** *Acad Radiol* 2022, **29:**779-785.

1481. Jones KL, Johnson MR, Lehnertz AY, Kramer RR, Drilling KE, Bungum LD, Bell SJ: **Rapid Deployment of Team Nursing During a Pandemic: Implementation Strategies and Lessons Learned.** *Crit Care Nurse* 2022, **42:**27-36.

1482. Jorge AES, Bennell KL, Kimp AJ, Campbell PK, Hinman RS: **An e-Learning Program for Physiotherapists to Manage Knee Osteoarthritis Via Telehealth During the COVID-19 Pandemic: Real-World Evaluation Study Using Registration and Survey Data.** *JMIR Med Educ* 2021, **7:**e30378.

1483. Jortberg BT, Beck Dallaghan GL, Schatte D, Christner J, Ryan MS: **Expansion of telehealth curriculum: National survey of clinical education leaders.** *J Telemed Telecare* 2022, **28:**464-468.

1484. Joshi P, Das S, Thomas M, Mawar S, Garg R, Shariff A, Gopichandran L: **Dissemination of Cardiopulmonary Resuscitation Training for Nurses Treating Coronavirus Disease-2019 Patients: A Single-arm Pre-experimental Study.** *Indian J Crit Care Med* 2022, **26:**327-330.

1485. Joshi S, Khan M, Jelen MB, Pandit AS: **Current Attitudes Toward Neuroanatomy: A Comparative Cross-Sectional Survey of Neurosurgeons from the United Kingdom and Worldwide.** *World Neurosurg* 2022, **166:**e607-e623.

1486. Joshi VR, Younger JM, Das S, Goud BKM, Pramanik K: **Factors influencing burnout in millennial medical students during the COVID-19 pandemic!** *Ir J Med Sci* 2022**:**44743.

1487. Juelsgaard J, Lofgren B, Toxvig N, Eriksen GV, Ebdrup L, Jensen RD: **Healthcare professionals' experience of using in situ simulation training in preparation for the COVID-19 pandemic: a qualitative focus group study from a Danish hospital.** *BMJ Open* 2022, **12:**e056599.

1488. Junaid Tahir M, Tariq W, Anas Tahseen Asar M, Irfan Malik M, Kamal Akhtar F, Malik M, Akhtar Q, Abbasher Hussien Mohamed Ahmed K, Talha Awan M, Ullah K, Asghar MS: **Psychological Impact of COVID-19 on Doctors and Medical Students of Punjab, Pakistan: A Logistic Regression Analysis.** *J Multidiscip Healthc* 2022, **15:**1297-1308.

1489. Jung D, De Gagne JC, Choi E, Lee K: **An Online International Collaborative Learning Program During the COVID-19 Pandemic for Nursing Students: Mixed Methods Study.** *JMIR Med Educ* 2022, **8:**e34171.

1490. Justaniah AI, Felemban BA, Garad F, Safar BO: **Interventional Radiology Board Examination Experience in Saudi Arabia during COVID-19.** *J Vasc Interv Radiol* 2022, **33:**201-202.

1491. Kabare G, Muthuuri N, Sarna K, Gwala F, Amuti T, Olabu B, Obimbo M, Ogeng’o J: **Perception and challenges of health science students toward e-learning in a Sub-Saharan African country: a multi-institutional study.** *Annals of African Surgery* 2022, **19:**16-22.

1492. Kabir H, Tonmon TT, Hasan MK, Biswas L, Chowdhury MAH, Islam MD, Rahman M, Mitra DK: **Association between preference and e-learning readiness among the Bangladeshi female nursing students in the COVID-19 pandemic: a cross-sectional study.** *Bull Natl Res Cent* 2022, **46:**8.

1493. Kadri R, Shetty PA, Kudva AA, Devika P, Shetty A, Sinhasan P: **Impact of COVID-19 on Academic Activities of Ophthalmology Postgraduates: A Cross-sectional Survey.** *Journal of Clinical and Diagnostic Research* 2021, **15:**NC07-NC12.

1494. Kai A, Park JH, Bay C: **The effectiveness of virtual interviews for postgraduate orthodontic residencies in North America.** *J World Fed Orthod* 2022, **11:**74-79.

1495. Kala PS, Thapliyal N, Pandey HS, Piyush AR, Maheshwari S, Chaudhary VS: **Medical students' perspective on online teaching during pandemic: Experience from a Government Medical College in Uttarakhand, India.** *J Educ Health Promot* 2021, **10:**473.

1496. Kam CT, Rait J, Brooke-Ball H, Ojofeitimi O: **Virtual surgical education for foundation doctors in the United Kingdom during COVID-19 pandemic: A qualitative study.** *Ann Med Surg (Lond)* 2022, **80:**104192.

1497. Kamel P, Brookmeyer C, Tang H, Solnes L, Lin CT: **Conference Attendance Tracking and Evaluation in the Era of Virtual Conferences.** *Acad Radiol* 2022, **29 Suppl 5:**S76-S81.

1498. Kamel S, Dobson JL, Patel P, Khatchikian AD, Rohren SA, Cheung JLS, Rooprai P, Gorman M, Tomasso D, Greidanus P, et al: **Teaching Radiology to Medical Students in Canada; a Virtual, Integrative, Clinical Approach.** *Can Assoc Radiol J* 2022, **73:**305-311.

1499. Kamel S, Wang MX, Ghannam S, Gopal A, Baqai F, Rohren S, Patel P, Khan Z, Aly M, Reiter AM, et al: **Acing the Fundamentals of Radiology: An Online Series for Medical Students and Interns.** *J Comput Assist Tomogr* 2022, **46:**614-620.

1500. Kamel S, Wang MX, Guccione J, Zhang X, Taher A, Sanhaji L, Hsieh P, Ferguson E, Elsayes KM: **Analyzing the Landscape of the 2021 Virtual Match: A Nationwide Survey of Radiology Programs' Stakeholders.** *Acad Radiol* 2022, **29:**1432-1446.

1501. Kamouna A, Alten F, Grabowski E, Eter N, Clemens CR: **High User Acceptance of a Retina e-Learning App in Times of Increasing Digitalization of Medical Training for Ophthalmologists.** *Ophthalmologica* 2022, **245:**368-375.

1502. Kandasamy S, John RR, Jayaraman A, Chinnakutti S, Chandrasekar M, Mallikarjunan DY: **Immediate Psychological Impact of Dental Students on COVID-19 Epidemic in India - A Cross Sectional Study.** *J Pharm Bioallied Sci* 2021, **13:**S1686-S1695.

1503. Kane C, Rintakorpi E, Wareing M, Hewson D: **The psychological effects of working in the NHS during a pandemic on final-year students: part 1.** *Br J Nurs* 2021, **30:**1303-1307.

1504. Kane C, Wareing M, Rintakorpi E: **The psychological effects of working in the NHS during a pandemic on final-year students: part 2.** *Br J Nurs* 2022, **31:**96-100.

1505. Kang KI, Lee N, Joung J: **Nursing students' experience of online peer tutoring based on the grow model: A qualitative study.** *Nurse Educ Today* 2021, **107:**105131.

1506. Kang SH, Kim TH, Son HJ, Park Y, Lee SH: **Validity of OSCE Evaluation Using the FLEX Model of Blended Learning.** *J Korean Med Sci* 2022, **37:**e163.

1507. Kappel C, Hijazi W, Singhal N: **Piloting 'Virtual Ward': a novel platform for delivering medical student education by residents.** *BMC Med Educ* 2022, **22:**392.

1508. Karabacak M, Ozkara BB, Ozcan Z: **Adjusting to the Reign of Webinars: Viewpoint.** *JMIR Med Educ* 2021, **7:**e33861.

1509. Karaca Bozdag Z, Candir BN, Gayretli O, Ari Z: **A survey-based research of medical faculty students' experiences on anatomy education during the Covid-19 pandemic process.** *Morphologie* 2022.

1510. Akyüz Karacan F, Yılmaz S, Kırpınar İ: **Psychosocial Adjustment of Healthcare Professionals During the COVID-19 Pandemic: Resident Doctors, Nurses, and Caregivers Need Extra Attention.** *Bakirkoy Tip Dergisi / Medical Journal of Bakirkoy* 2021, **17:**375-385.

1511. Karakoc K, Karabulut M, Kartal Ozcan E, Mujdeci B: **Audiology students’ opinions towards COVID-19 pandemic: occupational perspective and future expectations.** *Hearing, Balance and Communication* 2022, **20:**72-78.

1512. Kardangusheva AMD DAK, M. A. Chanaeva, A. Y. Makoeva, M. A. Khagabanova, I. S.: **Prevalence of anxiety and depression disorders among medical students one year after the start of the COVID-19 pandemic.** *Cardiometry***:**111-118.

1513. Kardangusheva AMK DZK, M. H. Budnik, A. F. Voronova, O. V. Gelyakhova, Z. A.: **Features of life activity and the incidence rate of anxiety and depressive disorders among medical students studying remotely during the epidemic of a new coronavirus infection (Covid 19).** *Cardiometry***:**130-137.

1514. Karimian Z, Farrokhi MR, Moghadami M, Zarifsanaiey N, Mehrabi M, Khojasteh L, Salehi N: **Medical education and COVID-19 pandemic: a crisis management model towards an evolutionary pathway.** *Educ Inf Technol (Dordr)* 2022, **27:**3299-3320.

1515. Karki P, Budhathoki L, Khadka M, Maharjan S, Dhakal S, Pokharel S, Poudel A, Rokaya P, Raut U, Rayamajhi S: **Willingness of Nepalese medical and nursing students to volunteer during COVID-19 pandemic: A single-centered cross-sectional study.** *Ann Med Surg (Lond)* 2021, **72:**103056.

1516. Karunaratne D, Karunaratne N, Wilmot J, Vincent T, Wright J, Mahmood N, Tang A, Sam AH, Reed M, Howlett D: **An Online Teaching Resource to Support UK Medical Student Education During the COVID-19 Pandemic: A Descriptive Account.** *Adv Med Educ Pract* 2021, **12:**1317-1327.

1517. Kasai H, Saito G, Ito S, Kuriyama A, Kawame C, Shikino K, Takeda K, Yahaba M, Taniguchi T, Igari H, et al: **COVID-19 infection control education for medical students undergoing clinical clerkship: a mixed-method approach.** *BMC Med Educ* 2022, **22:**453.

1518. Katzman JG, Thornton K, Sosa N, Tomedi L, Hayes L, Sievers M, Culbreath K, Norsworthy K, Martin C, Martinez A, et al: **Educating health professionals about COVID-19 with ECHO telementoring.** *Am J Infect Control* 2022, **50:**283-288.

1519. Kaurani P, Batra K, Rathore Hooja H, Banerjee R, Jayasinghe RM, Leuke Bandara D, Agrawal N, Singh V: **Perceptions of Dental Undergraduates Towards Online Education During COVID-19: Assessment from India, Nepal and Sri Lanka.** *Adv Med Educ Pract* 2021, **12:**1199-1210.

1520. Kaushik J, Chaitanya YVK, Kumar A, Jakhar P, Shetty R, Singhal A, Parihar JKS, Singh A: **Prevalence and effectiveness of innovative techniques in ophthalmic surgical training during COVID-19 pandemic in India.** *Indian J Ophthalmol* 2021, **69:**3704-3708.

1521. Kawasaki H, Yamasaki S, Rahman MM: **Developing a Hybrid Platform for Emergency Remote Education of Nursing Students in the Context of COVID-19.** *Int J Environ Res Public Health* 2021, **18**.

1522. Kazawa K, Shimpuku Y, Yoshinaga N: **Characteristics of early-career nurse researchers negatively impacted during the COVID-19 pandemic: a cross-sectional study.** *BMJ Open* 2022, **12:**e059331.

1523. Kazawa K, Teramoto C, Azechi A, Satake H, Moriyama M: **Undergraduate nursing students' learning experiences of a telehealth clinical practice program during the COVID-19 pandemic: A qualitative study.** *Nurse Educ Today* 2022, **111:**105297.

1524. Kearns L, Dupras D, Ortiz J, Catalanotti J: **Using Telemedicine to Preserve Internal Medicine Residency Continuity Clinic During the COVID-19 Pandemic: Results from a National Survey.** *J Gen Intern Med* 2022, **37:**2327-2329.

1525. Kehl L, Patil U, Tagorda M, Nelson-Hurwitz DC: **The Impacts of the COVID-19 Pandemic on Service-Learning Experiences Among Undergraduate Public Health Students in Hawai'i.** *Front Public Health* 2022, **10:**771844.

1526. Kells M, Jennings Mathis K: **Influence of COVID-19 on the next generation of nurses in the United States.** *J Clin Nurs* 2022.

1527. Kelly DE: **Initiation of a New Nurse Preceptor Program in the Era of COVID-19.** *J Contin Educ Nurs* 2022, **53:**221-224.

1528. Kelly EL, Casola AR, Smith K, Kelly S, de la Cruz MSD: **A qualitative analysis of third-year medical students' reflection essays regarding the impact of COVID-19 on their education.** *BMC Med Educ* 2021, **21:**481.

1529. Kelly R, Leung G, Lindstrom H, Wunder S, Yu JC: **Virtual Objective Structured Clinical Examination Experiences and Performance in Physical Medicine and Rehabilitation Residency.** *Am J Phys Med Rehabil* 2022, **101:**947-953.

1530. Kemp K, Baxa D, Cortes C: **Exploration of a Collaborative Self-Directed Learning Model in Medical Education.** *Med Sci Educ* 2022, **32:**195-207.

1531. Kennedy C, Doyle NM, Pedigo R, Toy S, Stoner A: **A novel approach to operating room readiness for airborne precautions using simulation-based clinical systems testing.** *Paediatr Anaesth* 2022, **32:**462-470.

1532. Khalafallah AM, Jimenez AE, Lam S, Gami A, Dornbos DL, 3rd, Sivakumar W, Johnson JN, Mukherjee D: **Burnout among medical students interested in neurosurgery during the COVID-19 era.** *Clin Neurol Neurosurg* 2021, **210:**106958.

1533. Khalafallah YM, Markowitz M, Levine WN, LaPorte DM, Aiyer AA: **Orthopaedic Surgery Residency Application, and Selection Criteria Adaptations, in Times of COVID-19: A Survey Study.** *JB JS Open Access* 2022, **7**.

1534. Khalid S, Aamir Rafique M, Azeem M, Jamil M, Hassan H, Abdullah HM: **Perception of E-Learning During COVID-19 Among Undergraduate Dental Students.** *Pakistan Journal of Medical and Health Sciences* 2021, **15:**3199-3203.

1535. Khamees DK CWT, S. Cranford, J. A. Carney, M. Harvey, C. Wolff, M. Haas, M. R. C. Hopson, L.: **Emergency medicine virtual conference participants' engagement with ep and competing activities.** *Western Journal of Emergency Medicine*, **23:**S18.

1536. Khan I, Afridi MZ, Wasim Z, Jawad NK, Wazir ZK, Asif S: **Frequency of Depression, Anxiety and Stress among Gynae Residents during Covid Pandemic.** *Pakistan Journal of Medical and Health Sciences* 2021, **15:**1894-1897.

1537. Khan NB, Erasmus T, Jali N, Mthiyane P, Ronne S: **Is blended learning the way forward? Students’ perceptions and attitudes at a South African university.** *African Journal of Health Professions Education* 2021, **13:**218-221.

1538. Kivlehan SM, Rybarczyk MM, Genisca AE, Lubetkin D, Kharel R, Lee JA, Michaeli N, Hynes EJC, Dixon J, Leifer N, Karim N: **Comparative Effectiveness of an In-Person and a Virtual Basic Emergency Care Instructor Course.** *Ann Glob Health* 2022, **88:**35.

1539. Kiyozumi T, Ishigami N, Tatsushima D, Araki Y, Yoshimura Y, Saitoh D: **Instructor Development Workshops for Advanced Life Support Training Courses Held in a Fully Virtual Space: Observational Study.** *JMIR Serious Games* 2022, **10:**e38952.

1540. Kızılcı E, Demiroğlu C, Duman B, Gümüş H: **Evaluation of Knowledge Levels of Dentistry Students on the COVID-19 and its Effects on Depression.** *Pesquisa Brasileira em Odontopediatria e Clínica Integrada* 2022, **22**.

1541. Koch LK, Correll-Buss A, Chang OH: **Implementation and Effectiveness of a Completely Virtual Pathology Rotation for Visiting Medical Students.** *Am J Clin Pathol* 2022, **157:**406-412.

1542. Koenig JFL, Buentzel J, Jung W, Truemper L, Wurm-Kuczera RI: **Using Instagram to Enhance a Hematology and Oncology Teaching Module During the COVID-19 Pandemic: Cross-sectional Study.** *JMIR Med Educ* 2021, **7:**e30607.

1543. Koff A, Burns R, Auerbach M, Lee B, Pearce J, Ciener D, Augenstein J, Yeung C, Thomas A: **Pediatric Emergency Medicine Didactics and Simulation (PEMDAS) Telesimulation Series: Hyperleukocytosis.** *MedEdPORTAL* 2021, **17:**11205.

1544. Koh PX, Tan K, Chan YC, Soon DT, See SJ, Tan NC: **Implementing a modified neurology OSCE during the COVID-19 pandemic: an implementation science perspective.** *Singapore Med J* 2022.

1545. Kolak V, Pavlovic M, Aleksic E, Biocanin V, Gajic M, Nikitovic A, Lalovic M, Melih I, Pesic D: **Probable Bruxism and Psychological Issues among Dental Students in Serbia during the COVID-19 Pandemic.** *Int J Environ Res Public Health* 2022, **19**.

1546. Kolcu GBK M: **Psychological Effects of COVID-19 in Medical Students.** *Psychiatr Danub*, **33:**387-391.

1547. Kolikonda MK, Blaginykh E, Brown P, Kovi S, Zhang LQ, Uchino K: **Virtual Rounding in Stroke Care and Neurology Education During the COVID-19 Pandemic - A Residency Program Survey.** *J Stroke Cerebrovasc Dis* 2022, **31:**106177.

1548. Kolodziej L, Ciechanowicz D, Rola H, Wolynski S, Wawrzyniak H, Rydzewska K, Podsiadlo K: **The impact of the COVID-19 pandemic on Polish orthopedics, in particular on the level of stress among orthopedic surgeons and the education process.** *PLoS One* 2021, **16:**e0257289.

1549. Kubiszewski K, Gulani A, Sutter K, Sarmiento B, Ghattas YS, Mathai R, Simms-Cendan JS: **Migration of an Interactive Global Health Conference to a Virtual Platform: Engaging Learners During the Pandemic.** *Cureus* 2022, **14:**e25601.

1550. Kubo A, Onoda K, Yakabi A: **Student satisfaction with initiatives for the national examination during the COVID-19 pandemic.** *J Phys Ther Sci* 2021, **33:**854-856.

1551. Kufel WD, Blaine BE, Avery LM: **Pharmacy students' knowledge and confidence of COVID-19 following an interactive didactic class.** *J Am Coll Clin Pharm* 2022, **5:**1082-1087.

1552. Kui A, Jiglau AL, Chisnoiu A, Negucioiu M, Balhuc S, Constantiniuc M, Buduru S: **A survey on dental students' perception regarding online learning during the COVID-19 pandemic.** *Med Pharm Rep* 2022, **95:**203-208.

1553. Kulshreshtha P, Bahurupi Y, Dhar M, Sharma S, Kathrotia R, Rao S, Naithani M, Gupta M: **Preparedness of Undergraduate Medical Students to Combat COVID-19: A Tertiary Care Experience on the Effectiveness and Efficiency of a Training Program and Future Prospects.** *Cureus* 2022, **14:**e22971.

1554. Kumar A, Kalal N, Rana N, Vyas H, Choudhary V, Rani R: **Online learning in nursing students: Satisfaction and barriers.** *J Educ Health Promot* 2021, **10:**411.

1555. Kumar GNP, Urs ANR, Undi M, Bakkannavar SM: **Online viva voce as a formative assessment method in forensic medicine during COVID-19 pandemic.** *J Educ Health Promot* 2022, **11:**160.

1556. Kumar R, Kumar H, Kumari R, Dars J, Qureshi S, Hamza MA, Khoso AB, Mubeen SM: **The Impact of Covid-19 on Medical Students: A Cross Sectional Survey.** *Pakistan Journal of Medical and Health Sciences* 2021, **15:**2905-2909.

1557. Kumar SEJ, Ganesan G, Anbu G, Priya T, Selvaraj V: **Perception of Learning Environment Among Anaesthesiology Residents During the Pandemic in a Tertiary Hospital in India: Comparative Cross-Sectional Study.** *Turk J Anaesthesiol Reanim* 2022, **50:**S50-S56.

1558. Kumari S, Gautam H, Nityadarshini N, Das BK, Chaudhry R: **Online classes versus traditional classes? Comparison during COVID-19.** *J Educ Health Promot* 2021, **10:**457.

1559. Kunaviktikul W, Ang E, Baridwan NS, Bernal AB, Dones LBP, Flores JL, Freedman-Doan R, Klunklin A, Lee WL, Lin CC, et al: **Nursing students' and faculty members' experiences of online education during COVID-19 across Southeast Asia: A Photovoice study.** *Nurse Educ Today* 2022, **111:**105307.

1560. Kurniawan AH, Yusmaniar, Safitri, Nur A: **Effects of health supplement self-medication learning media on health student behaviours during the COVID-19 pandemic.** *Pharmacy Education* 2022, **22:**30-35.

1561. Kuroda N, Suzuki A, Ozawa K, Nagai N, Okuyama Y, Koshiishi K, Yamada M, Raita Y, Kakisaka Y, Nakasato N, Kikukawa M: **Educational Approaches That Enhance Online Clinical Clerkship during the COVID-19 Pandemic.** *Intern Med* 2022, **61:**2431-2440.

1562. Kurtulmus-Yilmaz S, Onoral O: **Effectiveness of screen-to-screen and face-to-face learning modalities in dental anatomy module during Covid-19 pandemic.** *Anat Sci Educ* 2022, **15:**57-66.

1563. Kurz S, Buggenhagen H, Wachter N, Penzkofer L, Dietz SO, Konig TT, Heinemann MK, Neulen A, Hanke LI, Huber T: **[Testing of practical surgical teaching at a distance-Experiences with a hybrid OSCE in surgery].** *Chirurgie (Heidelb)* 2022, **93:**976-982.

1564. OA. K: **Jordanian nursing students' attitudes towards e-learning during COVID-19.** *NeuroQuantology*, **20:**1314-1321.

1565. Lai T, Liang W, Zhong M, Zhu P, Li B: **Current Status of Chinese Medical Students' Professional Identity After COVID-19 and the Factors That Influence It.** *Front Psychol* 2022, **13:**816767.

1566. Lam Shang Leen J, Wong TTC, Ku CW, Koh JC, Nguyen TAP, Shahdadpuri R, Mathur M, Chow C: **Impact of COVID-19 on paediatric and OBGYN residency training in Singapore.** *Ann Acad Med Singap* 2021, **50:**717-720.

1567. Lambdin J, Lin RP, DeAngelis EJ, Vaziri K, Lin P, Lee J, Jackson HT: **Analysis of Surgery Residency Website Content: Implications during the COVID-19 Era.** *J Surg Educ* 2022, **79:**904-908.

1568. Lannan FM, Cho S: **Developing and Measuring Effectiveness of a Distance Learning Dermatology Course: A Prospective Observational Study.** *Cutis* 2022, **109:**228-230.

1569. Lawrence K, Cho J, Torres C, Alfaro-Arias V: **Building Virtual Health Training Tools for Residents: A Design Thinking Approach.** *Front Digit Health* 2022, **4:**861579.

1570. Lechner A, Haider SP, Paul B, Escrihuela Branz PFF, Felicio-Briegel A, Widmann M, Huber J, Stadlberger U, Canis M, Schrotzlmair F, Sharaf K: **Misjudgment of Skills in Clinical Examination Increases in Medical Students Due to a Shift to Exclusively Online Studies during the COVID-19 Pandemic.** *J Pers Med* 2022, **12**.

1571. Lee GW, Kim GB, Lee IJ: **The impact of novel COVID-19 initial pandemic on orthopedic healthcare: an experience from a single institution of South Korea.** *Journal of the Korean Medical Association* 2021, **64:**778-787.

1572. Lee J, Son HK: **Effects of simulation problem-based learning based on Peplau's Interpersonal Relationship Model for cesarean section maternity nursing on communication skills, communication attitudes and team efficacy.** *Nurse Educ Today* 2022, **113:**105373.

1573. Lee J, Stevens MN, Landeen KC, Lipscomb BE, Whigham AS: **A Four-Week Otolaryngology Virtual Educational Curriculum for Prospective Applicants.** *Ear Nose Throat J* 2021**:**1455613211052337.

1574. Lee JX, Ahmad Azman AH, Ng JY, Ismail NAS: **Deciphering Learning Motivation in Open Distance Learning towards Sustainable Medical Education.** *Sustainability* 2022, **14**.

1575. Lee KE, Lim F, Silver ER, Faye AS, Hur C: **Impact of COVID-19 on residency choice: A survey of New York City medical students.** *PLoS One* 2021, **16:**e0258088.

1576. Lee T, Yoon SW, Fernando S, Willey S, Kumar A: **Blended (online and in-person) Women's Health Interprofessional Learning by Simulation (WHIPLS) for medical and midwifery students.** *Aust N Z J Obstet Gynaecol* 2022, **62:**596-604.

1577. Liu Z, Liu R, Zhang Y, Zhang R, Liang L, Wang Y, Wei Y, Zhu R, Wang F: **Latent class analysis of depression and anxiety among medical students during COVID-19 epidemic.** *BMC Psychiatry* 2021, **21:**498.

1578. Lobos K, Cobo-Rendon R, Mella-Norambuena J, Maldonado-Trapp A, Fernandez Branada C, Bruna Jofre C: **Expectations and Experiences With Online Education During the COVID-19 Pandemic in University Students.** *Front Psychol* 2021, **12:**815564.

1579. Loda T, Erschens RS, Nevins AB, Zipfel S, Herrmann-Werner A: **Perspectives, benefits and challenges of a live OSCE during the COVID-19 pandemic in a cross-sectional study.** *BMJ Open* 2022, **12**.

1580. Long X, Zhang J, Chen J, Shi L, Li X, Yi W, Chen Z, Kosasih S, Yu Z, Mei A, Wang C: **Analyzing the influence of COVID-19 epidemic on the employment intention of resident physicians in China.** *J Infect Dev Ctries* 2022, **16:**1009-1015.

1581. Looi JC, Maguire PA, Bonner D, Reay RE, Finlay AJ, Keightley P, Tedeschi M, Wardle C, Kramer D: **Final-year medical student Psychiatry and Addiction Medicine synchronous summative tele-assessments during a COVID-19 Delta-variant stay-at-home lockdown.** *Australas Psychiatry* 2022, **30:**564-569.

1582. Loset IH, Laegreid T, Rodakowska E: **Dental Students' Experiences during the COVID-19 Pandemic-A Cross-Sectional Study from Norway.** *Int J Environ Res Public Health* 2022, **19**.

1583. Low EZ, O'Sullivan NJ, Sharma V, Sebastian I, Meagher R, Alomairi D, Alhouti EH, Donohoe CL, Kelly ME: **Assessing medical students' perception and educational experience during COVID-19 pandemic.** *Ir J Med Sci* 2022.

1584. Lowe JT, Patel SR, Hao WD, Butt A, Strehlow M, Lindquist B: **Teaching From Afar: Development of a Telemedicine Curriculum for Healthcare Workers in Global Settings.** *Cureus* 2021, **13:**e20123.

1585. Lu L, Wang X, Wang X, Guo X, Pan B: **Association of Covid-19 pandemic-related stress and depressive symptoms among international medical students.** *BMC Psychiatry* 2022, **22:**20.

1586. Margolin EJ, Kurtzman JT, Gordon RJ, Anderson CB, Badalato GM: **Efficacy of an Online Blended Learning Curriculum to Improve Medical Student Urologic Education.** *Med Sci Educ* 2021, **31:**2007-2015.

1587. Margolin EJ, Mikhail D, Paniagua-Cruz A, Kavoussi LR, Badalato GM, Richstone L: **National Implementation and Evaluation of a Virtual Subinternship in Urology.** *Urology* 2022, **164:**55-62.

1588. Mariya CG, Joseph A, Marina A, Jesly MK, Veronica M, Sabeena MTP: **Study on Satisfaction of Students Regarding Online Classes in a Selected College of Nursing, Mangalore.** *Journal of Health and Allied Sciences NU* 2022.

1589. Maroof FI MK, S. A. Iqbal, M.: **Impact of COVID-19 on medical education and anxiety levels of medical students.** *Rawal Medical Journal*, **47:**434-437.

1590. Martelli AJ, Machado RA, Martelli DRB, Cruz Perez DEd, Pires FR, Martelli Júnior H: **Clinical and Research Activities of the Brazilian Productivity Fellows in Oral Medicine and Oral Pathology during the COVID-19 Era.** *Pesquisa Brasileira em Odontopediatria e Clínica Integrada* 2021, **21**.

1591. Martin A, Raber JP, Shayer D, Lai D, Goodcoff A, Kannikal J, Raja AS, He S: **Get waivered remote: Nationwide, remote DEA-x waiver course in response to COVID-19.** *Digit Health* 2021, **7:**20552076211048985.

1592. Martin EC, Hakimi AA, McIntosh C, Wong BJF: **The Transition to Online Rhinoplasty Education Amid COVID-19: Surgeon Perspectives and Areas of Improvement.** *Facial Plast Surg Aesthet Med* 2022, **24:**134-136.

1593. Martin P, Lizarondo L, Argus G, Kumar S, Kondalsamy-Chennakesavan S: **Impact of the COVID-19 Pandemic on Clinical Supervision of Healthcare Students in Rural Settings: A Qualitative Study.** *Int J Environ Res Public Health* 2022, **19**.

1594. Martin P, McGrail M, Fox J, Partanen R, Kondalsamy-Chennakesavan S: **Impact of the COVID-19 pandemic on medical student placements in rural Queensland: A survey study.** *Aust J Rural Health* 2022, **30:**478-487.

1595. Martin SK, Finn KM, Kisielewski M, Simmons R, Zaas AK: **Residency Program Responses to Early COVID-19 Surges Highlight Tension as to Whether Residents Are Learners or Essential Workers.** *Acad Med* 2022.

1596. Martinez EG, Padrón RR, Villalba PJ: **The Students' Point of View on the Teaching of Anatomy at the Universidad del Norte, Colombia, Amid the Covid-19 Pandemic.** *International Journal of Morphology* 2022, **40:**46-50.

1597. Martinez-Cuazitl A, Martinez-Salazar IN, Maza-De La Torre G, Garcia-Davila JA, Montelongo-Mercado EA, Garcia-Ruiz A, Noyola-Villalobos HF, Garcia-Araiza MG, Hernandez-Diaz S, Villegas-Tapia DL, et al: **Burnout Syndrome in a Military Tertiary Hospital Staff during the COVID-19 Contingency.** *Int J Environ Res Public Health* 2022, **19**.

1598. Martini ML, Shrivastava RK, Kellner CP, Morgenstern PF: **Evaluation of a Role for Virtual Neurosurgical Education for Medical Students Over 2 Years of a Global Pandemic.** *World Neurosurg* 2022, **166:**e253-e262.

1599. Masha'al D, Shahrour G, Aldalaykeh M: **Anxiety and coping strategies among nursing students returning to university during the COVID-19 pandemic.** *Heliyon* 2022, **8:**e08734.

1600. Mason MW, Aruma JC: **An Orthopaedic Virtual Clinical Clerkship for Visiting Medical Students: Early Successes and Future Implications.** *J Surg Educ* 2022, **79:**535-542.

1601. Mastoras G, Farooki N, Willinsky J, Dharamsi A, Somers A, Gray A, Yaphe J, Dalseg T, O'Connor E: **Rapid deployment of a virtual simulation curriculum to prepare for critical care triage during the COVID-19 pandemic.** *CJEM* 2022, **24:**382-389.

1602. Mastour HG AMN, S.: **Study of Association between Serum Hepsin Level and Lymphocyte-to-C-reactive Protein Ratio in Patients with Diabetes.** *Iranian Red Crescent Medical Journal* 2022, **24**.

1603. Matheen A, Sruthi G, Shinisha DP, Chokkalingam S, Arumugam B, Gnaneswaran P: **Impact of Lockdown due to COVID-19 Pandemic on Undergraduate Ophthalmology Teaching: Students’ and Teachers’ Perspective.** *Journal of Clinical and Diagnostic Research* 2021, **15:**NC09-NC14.

1604. Matta A, Adamson R, Hayes MM, Carmona H, Soffler MI, Benzaquen S, Gupta E: **Impact of the COVID-19 Pandemic on U.S. Pulmonary and Critical Care Medicine Fellowship Training.** *ATS Sch* 2021, **2:**556-565.

1605. May CC, Atyia SA, Hafford AJ, Smetana KS: **Clinical Advanced Pharmacy Practice Experience Rotations During COVID-19: Evaluation of a Transition to Virtual Learning.** *J Pharm Pract* 2022**:**8971900221087116.

1606. McCann A, Chang K, Gengler I, Cervenka B, Nellis JC, Hsieh TY: **Assessment of Virtual AAFPRS Fellowship Interviews During the COVID-19 Pandemic: A Pandemic Response or the Wave of the Future?** *Facial Plast Surg Aesthet Med* 2022, **24:**410-412.

1607. McDonald A, Qiabi M, Lewis D, Leeper R, Fortin D, Inculet R, Malthaner R: **Surgery crisis simulation during the COVID-19 pandemic.** *Can J Surg* 2022, **65:**E154-E158.

1608. McFadden S, Guille S, Daly-Lynn J, O'Neill B, Marley J, Hanratty C, Shepherd P, Ramsey L, Breen C, Duffy O, et al: **Academic, clinical and personal experiences of undergraduate healthcare students during the COVID-19 pandemic: A prospective cohort study.** *PLoS One* 2022, **17:**e0271873.

1609. McInerney NJ, Khan MF, Coady L, Dalli J, Stokes M, Donnelly S, Heneghan H, Cahill R: **Implementation of an on-site simulation programme during COVID-19 and the assessment of its impact on medical students' competence.** *Ir J Med Sci* 2022**:**44713.

1610. McKay MA, Pariseault CA, Whitehouse CR, Smith T, Ross JG: **The experience of baccalaureate clinical nursing faculty transitioning to emergency remote clinical teaching during the COVID-19 pandemic: Lessons for the future.** *Nurse Educ Today* 2022, **111:**105309.

1611. McLean ME, Cotarelo AA, Huls TA, Husain A, Hillman EA, Cygan LD, Archer LO, Beck-Esmay J, Burke SM, Carrick AI, et al: **UME-to-GME PandEMonium in COVID-19: Large-Scale Implementation of a Virtual ACGME Milestone-Based Curriculum for Senior Medical Students Matched Into Emergency Medicine.** *J Grad Med Educ* 2021, **13:**848-857.

1612. McMillan DG, Kalloo OR, Lara RA, Pavlova M, Kritz-Silverstein D: **Factors Affecting Dental Students' Comfort with Online Synchronous Learning.** *Dent J (Basel)* 2022, **10**.

1613. McWatt SC: **Responding to Covid-19: A thematic analysis of students' perspectives on modified learning activities during an emergency transition to remote human anatomy education.** *Anat Sci Educ* 2021, **14:**721-738.

1614. Mehmood N, Akhlaq H, Nasir M, Sajjad I, Hanif S, Sheikh H: **E-learning in the era of Covid-19 Pandemic: the Challenges and Opportunities.** *Pakistan Journal of Medical and Health Sciences* 2021, **15:**3228-3232.

1615. Mendes TB, Souza KCd, França CN, Rossi FE, Santos RPG, Duailibi K, Tuleta I, Armond JdE, Stubbs B, Neves LM: **Physical Activity and Symptoms of Anxiety and Depression among Medical Students during a Pandemic.** *Revista Brasileira de Medicina do Esporte* 2021, **27:**582-587.

1616. Messina DM, Mikhail SS, Messina MJ, Novopoltseva IA: **Assessment of learning outcomes of first year dental students using an interactive Nearpod educational platform.** *J Dent Educ* 2022, **86:**893-899.

1617. Metin Karaaslan M, Celik I, Kurt S, Yilmaz Yavuz A, Bektas M: **Undergraduate nursing students' experiences of distance education during the COVID-19 pandemic.** *J Prof Nurs* 2022, **38:**74-82.

1618. Metz CJ, Metz MJ: **The benefits of incorporating active learning into online, asynchronous coursework in dental physiology.** *Adv Physiol Educ* 2022, **46:**11-20.

1619. Meuwly JY, Mandralis K, Tenisch E, Gullo G, Frossard P, Morend L: **Use of an Online Ultrasound Simulator to Teach Basic Psychomotor Skills to Medical Students During the Initial COVID-19 Lockdown: Quality Control Study.** *JMIR Med Educ* 2021, **7:**e31132.

1620. Meyer AM, Hart AA, Keith JN: **COVID-19 Increased Residency Applications and How Virtual Interviews Impacted Applicants.** *Cureus* 2022, **14:**e26096.

1621. Meyer C, Barrett C, Joubert G, Mofolo N: **The effect of the initial months of the COVID-19 national lockdown on MMed training activities at the University of the Free State, South Africa.** *African Journal of Health Professions Education* 2022, **14:**33-42.

1622. Michaeli D, Keough G, Perez-Dominguez F, Polanco-Ilabaca F, Pinto-Toledo F, Michaeli J, Albers S, Achiardi J, Santana V, Urnelli C, et al: **Medical education and mental health during COVID-19: a survey across 9 countries.** *Int J Med Educ* 2022, **13:**35-46.

1623. Mileder LP, Bereiter M, Wegscheider T: **Telesimulation as a modality for neonatal resuscitation training.** *Med Educ Online* 2021, **26:**1892017.

1624. Min A KC S-EJ, Lee.: **Effect of Virtual Simulation Practice for Nursing Students: Focusing on Virtual Presence and Virtual Patient Learning System Evaluation (VPLSE).** *Journal of Korean Society for Simulation in Nursing*, **10:**89-102.

1625. Miracle DK, Thomas J, Keenan NF, Kuhn RJ, Schadler AD, Taylor S: **A Survey of Pandemic Impacts on Pharmacy Residency Applications, Interviews, and Selection Factors.** *Am J Pharm Educ* 2022**:**8954.

1626. Mirchia K, Khurana K: **Financial and educational impact of the COVID-19 pandemic in an academic hospital-based tertiary cytopathology practice.** *J Am Soc Cytopathol* 2022, **11:**46-55.

1627. Mirhosseini S, Grimwood S, Dadgari A, Basirinezhad MH, Montazeri R, Ebrahimi H: **One-year changes in the prevalence and positive psychological correlates of depressive symptoms during the COVID-19 pandemic among medical science students in northeast of Iran.** *Health Sci Rep* 2022, **5:**e490.

1628. Mirman B, Mukhdomi TJ, Rajaguru P, Whitten I, Fragoza K, Siddiqui A, Carayannopoulos A, Kendall MC: **Pain Education During the Coronavirus Pandemic: The Introduction of Pain Medicine During the Pre-Clinical Years.** *Pain Med* 2022, **23:**609-613.

1629. Mirsepassi Z, Karimi E, Mohammadjafari A: **Psychiatric training program during the COVID-19 pandemic: An experience in Iran.** *Asian J Psychiatr* 2022, **73:**103130.

1630. Mishra J, Panigrahi A, Samanta P, Dash K, Mahapatra P, Behera MR: **Sleep quality and associated factors among undergraduate medical students during Covid-19 confinement.** *Clin Epidemiol Glob Health* 2022, **15:**101004.

1631. Mitchnik IY, Rivkind AI: **Succeeding in Continuing Trauma Education During a Pandemic.** *World J Surg* 2022, **46:**977-981.

1632. Mladenovic R, AlQahtani S, Mladenovic K, Bukumiric Z, Zafar S: **Effectiveness of technology-enhanced teaching methods of undergraduate dental skills for local anaesthesia administration during COVID-19 era: students' perception.** *BMC Oral Health* 2022, **22:**40.

1633. Mohr S, Küfe B, Rheingans A, Guse J: **Attitude Towards and Adoption of the Novel Learning Environment Among Undergraduate Medical Students During COVID-19.** *Frontiers in Education* 2021, **6**.

1634. Mohsin SF, Shah SA, Agwan MAS, Ali S, Alsuwaydani ZA, AlSuwaydani SA: **Effect of the COVID-19 pandemic on dental interns in Saudi Arabia.** *Work* 2022, **71:**825-831.

1635. Moini A, Maajani K, Omranipour R, Zafarghandi MR, Aleyasin A, Oskoie R, Alipour S: **Residency training amid the COVID-19 pandemic: exploring the impact on mental health and training, a lesson from Iran.** *BMC Med Educ* 2021, **21:**603.

1636. Moll-Khosrawi P, Falb A, Pinnschmidt H, Zollner C, Issleib M: **Virtual reality as a teaching method for resuscitation training in undergraduate first year medical students during COVID-19 pandemic: a randomised controlled trial.** *BMC Med Educ* 2022, **22:**483.

1637. Monkman H, Palmer R, Ijams S, Kollaja L, Rodriguez KA, Liew A, Wickham A, Wen F, Miller B, Lesselroth BJ: **Using Simulations to Train Medical Students for Unanticipated Technology Failures in Telemedicine.** *Stud Health Technol Inform* 2022, **294:**775-779.

1638. Montagna E, Donohoe J, Zaia V, Duggan E, O'Leary P, Waddington J, O'Tuathaigh C: **Transition to clinical practice during the COVID-19 pandemic: a qualitative study of young doctors' experiences in Brazil and Ireland.** *BMJ Open* 2021, **11:**e053423.

1639. Moodley R, Singh S, Moodley I: **Undergraduate dental students’ perspectives on teaching and learning during the COVID-19 pandemic: Results from an online survey conducted at a South African university using a mixed-methods approach.** *African Journal of Health Professions Education* 2022, **14:**26-32.

1640. Moradi YB RF, Aram HajiAliBeigloo, Reza.: **Challenges of the sudden shift to asynchronous virtual education in nursing education during the COVID-19 pandemic: A qualitative study.** *NURSING AND MIDWIFERY STUDIES*, **11:**44-50.

1641. Moraes F, Baumont A, Dreher CB, Gauer G, Manfro GG: **Psychiatric outcomes and overall functionality in students from health care programs during the pandemic: a cross-sectional study at the first COVID-19 wave in Brazil.** *Trends Psychiatry Psychother* 2022.

1642. Moralez EA, Boren RL, Lebel DL, Drennan M, Olvera DR, Thompson B: **Teaching Strategies During the COVID-19 Pandemic: Tailoring Virtual Learning for Public Health and Cancer Health Disparities Education.** *Front Public Health* 2022, **10:**845400.

1643. Moran SK, Nguyen JK, Grimm LJ, Yee JM, Maxfield CM, Shah N, Heitkamp DE, Chapman T: **Should Radiology Residency Interviews Remain Virtual? Results of a Multi-institutional Survey Inform the Debate.** *Acad Radiol* 2022, **29:**1595-1607.

1644. Moreno-Sanchez E, Merino-Godoy MD, Pinero-Claros S, Santiago-Sanchez A, Del-Campo-Jimenez A, Mariscal-Perez L, Rodriguez-Miranda FP, Costa EI, Gago-Valiente FJ: **Nursing Education during the SARS-CoV-2 Pandemic: Assessment of Students' Satisfaction with e-Learning Environment.** *Int J Environ Res Public Health* 2022, **19**.

1645. Morris SG, Greenstone H, Chute R: **Keeping it human: Pandemic era psychiatry teaching.** *Clin Teach* 2021, **18:**641-649.

1646. Mortagy M, Abdelhameed A, Sexton P, Olken M, Hegazy MT, Gawad MA, Senna F, Mahmoud IA, Shah J, Egyptian Medical Education Collaborative G, Aiash H: **Online medical education in Egypt during the COVID-19 pandemic: a nationwide assessment of medical students' usage and perceptions.** *BMC Med Educ* 2022, **22:**218.

1647. Mortazavi F, Ghardashi F: **Medical students' psychological and behavioral responses to the COVID-19 pandemic: A descriptive phenomenological study.** *Clin Child Psychol Psychiatry* 2022, **27:**291-307.

1648. Moschovis PP, Dinesh A, Boguraev AS, Nelson BD: **Remote online global health education among U.S. medical students during COVID-19 and beyond.** *BMC Med Educ* 2022, **22:**353.

1649. Mosher C, Mukhtar F, Alnaami N, Akkielah YA, Alsharif J, Khan T, Taskiran HC, Zafar M: **Donning and Doffing of Personal Protective Equipment: Perceived Effectiveness of Virtual Simulation Training to Decrease COVID-19 Transmission and Contraction.** *Cureus* 2022, **14:**e22943.

1650. Motte-Signoret E, Labbe A, Benoist G, Linglart A, Gajdos V, Lapillonne A: **Perception of medical education by learners and teachers during the COVID-19 pandemic: a cross-sectional survey of online teaching.** *Med Educ Online* 2021, **26:**1919042.

1651. Moxham L, Fernandez R, Lord H, Halcomb E, Middleton R: **Life during lockdown: Coping strategies used by preregistration nursing students during COVID-19.** *Nurse Educ Pract* 2022, **63:**103388.

1652. Moya-Plana A, Tselikas L, Lambotte O, Temam S, De Baere T, Deutsch E, Barlesi F, Blanchard P, Levy A: **Postgraduate oncology educational shifts during the COVID-19 pandemic: results of faculty and medical student surveys.** *ESMO Open* 2022, **7:**100451.

1653. Muflih S, Abuhammad S, Al-Azzam S, Alzoubi KH, Muflih M, Karasneh R: **Online learning for undergraduate health professional education during COVID-19: Jordanian medical students' attitudes and perceptions.** *Heliyon* 2021, **7:**e08031.

1654. Mufti AHA DSB, Khulud Ayman Altalhi, Elaf Rudda Aljunaid, Baydaa Safar Abdulmuttalib, Jannat Abdullah Althebeti, Raneem Raja.: **THE ASSOCIATION BETWEEN DISTANCE LEARNING AND OCCURRENCE OF DEPRESSION AND ANXIETY: A CROSS-SECTIONAL STUDY AMONG MALE AND FEMALE MEDICAL STUDENTS AT UMM AL-QURA UNIVERSITY.** *International Journal of Biology and Biotechnology*, **19:**275-281.

1655. Mukasa J, Otim M, Monaco B, Al Marzouqi A, Breitener P, Jawahar L: **Nursing Students' Perspectives and Readiness to Transition to E-Learning During COVID-19 in the UAE: A Cross-Sectional Study.** *Adv Med Educ Pract* 2021, **12:**1505-1512.

1656. Mukharyamova L, Ziganshina A, Zhidjaevskij A, Galimova L, Kuznetsov M: **Medical students in Russia evaluate the training during the COVID-19 pandemic: a student survey.** *BMC Med Educ* 2021, **21:**560.

1657. Mulcahy CF, Terhaar SJ, Boulos S, Lee E, Zapanta PE: **Did More Otolaryngology Residency Applicants Match at Their Home Institutions in 2021? Investigating the Impact of the COVID-19 Pandemic.** *Ann Otol Rhinol Laryngol* 2022, **131:**1375-1380.

1658. Mulcahy H, Brennan C, Pardy A, McCormack B, Heslin J: **Implementing public health nursing training for Ireland's National Healthy Childhood Programme.** *Public Health Nurs* 2022, **39:**839-846.

1659. Mullen LA, Nguyen DL, Katzen JT, Brem RF, Ambinder EB: **Virtual Interviews for Breast Imaging Fellowship During the COVID-19 Pandemic: Perspectives of Program Directors and Applicants.** *Journal of Breast Imaging* 2022, **4:**309-319.

1660. Muller A, Sawicki OA, Pommee M, Blazejewski T, Schneider K, Ullmann-Moskovits J, Petersen JJ, Muller BS: **Providing an interactive undergraduate elective on safety culture online - concept and evaluation.** *BMC Med Educ* 2022, **22:**508.

1661. Mulligan KM, Pan X, Gerges C, Rabah NM, Selden NR, Wolfe SQ, Wright CH, Wright JM, 3rd: **The 2021 Neurosurgery Match: An Analysis of the Impact of Virtual Interviewing and Other COVID-19-Related Changes.** *World Neurosurg* 2022, **162:**e8-e13.

1662. Mumford S, Newton M, Benzie C, Forster D, Matthews R, Hyde R, Llewelyn F, McLachlan H: **Supporting the midwifery workforce: An evaluation of an undergraduate midwifery student employment model at a large tertiary maternity service in Victoria, Australia.** *Women and Birth* 2022.

1663. Murthy AK, Fontan CT, Filippa Trikantzopoulou M, Fitzpatrick THt, Levy JM, Alt JA, Schuman TA: **Impact of the COVID-19 pandemic on otolaryngology resident rhinology education.** *Int Forum Allergy Rhinol* 2022, **12:**1067-1070.

1664. Mutalik VS, Upadhyaya J, Le M, Schonwetter DJ: **The impact of COVID-19 on the practice of Oral and Maxillofacial Pathology in the United States and Canada.** *Med Oral Patol Oral Cir Bucal* 2022, **27:**e434-e441.

1665. N OL, Brouder N, Bessell N, Frizelle P: **An exploration of speech and language pathology student and facilitator perspectives on problem-based learning online.** *Clin Linguist Phon* 2022**:**1-19.

1666. Nabhani Y, Xie VK, Badawy M, Karim R, Abdullatif U, Negm AS, Bhosale H, Rohren S, Elhatw A, Ghannam S, et al: **Multidisciplinary approach of teaching radiology to medical students in Egypt: Is this an effective method?** *Egyptian Journal of Radiology and Nuclear Medicine* 2021, **52**.

1667. Nabolsi M, Abu-Moghli F, Khalaf I, Zumot A, Suliman W: **Nursing Faculty Experience With Online Distance Education During COVID-19 Crisis: A Qualitative Study.** *J Prof Nurs* 2021, **37:**828-835.

1668. Nadareishvili I, Syunyakov T, Smirnova D, Sinauridze A, Tskitishvili A, Tskitishvili A, Zhulina A, Patsali ME, Manafis A, Fountoulakis NK, Fountoulakis KN: **University students' mental health amidst the COVID-19 pandemic in Georgia.** *Int J Soc Psychiatry* 2022, **68:**1036-1046.

1669. Naidoo N, Azar AJ, Khamis AH, Gholami M, Lindsbro M, Alsheikh-Ali A, Banerjee Y: **Design, Implementation, and Evaluation of a Distance Learning Framework to Adapt to the Changing Landscape of Anatomy Instruction in Medical Education During COVID-19 Pandemic: A Proof-of-Concept Study.** *Front Public Health* 2021, **9:**726814.

1670. Najeeb TA MR, M. Hussain, M. Siddique, N. Asad, S.: **Online medical teaching during covid-19 pandemic: Level of satisfaction among students and faculty of a medical college in Islamabad, Pakistan.** *Rawal Medical Journal*, **46:**963-966.

1671. Nakagawa H, Sasai H: **Nursing Students' Practicums during the COVID-19 Crisis and the Effect on Infection-Prevention Behavior in Students: A Mixed-Method Approach.** *Medicina (Kaunas)* 2021, **57**.

1672. Naqvi SAA, Hasan B, Zia R, Ayaz N, Riaz S, Sajid S, Bin Rehan AH, Shahid U, Bilal A, Afzal A: **Mental Health and Its Association with Changes in Quality of Life Due to Covid-19 Pandemic among Undergraduate Students in Islamabad.** *Pakistan Journal of Public Health* 2022, **11:**220-227.

1673. Naseem SR SQ, M. A. Mangrio, S. Bareach, G. Naeem, Z.: **Conventional to virtual poster presentation in scholars' day during coronavirus disease-19 lock down: Medical students' performance and perspective.** *Int J Health Sci (Qassim)*, **15:**23-27.

1674. Naseer S, Zulfikar I, Zaheer F, Butt F, Sagheer S, Kazim E: **Perception of MBBS Students on Hybrid Teaching in Covid-19 Era: A Survey of Public Sector Medical University.** *Pakistan Journal of Medical and Health Sciences* 2021, **15:**3154-3155.

1675. Natarajan J, Joseph MA: **Impact of emergency remote teaching on nursing students' engagement, social presence, and satisfaction during the COVID-19 pandemic.** *Nurs Forum* 2022, **57:**42-48.

1676. Nath AA SN, C. Powell, S. Choi, E. P. Oyoyo, U. Christensen, H. Won, J. Kwon, S. R.: **The Outcome of a New Teledentistry Initiative in Response to the COVID-19 Pandemic: A Cross-sectional Study.** *J Contemp Dent Pract*, **23:**284-288.

1677. Natsis K, Lazaridis N, Kostares M, Anastasopoulos N, Chytas D, Totlis T, Piagkou M: **"Dissection Educational Videos" (DEVs) and their contribution in anatomy education: a students' perspective.** *Surg Radiol Anat* 2022, **44:**33-40.

1678. Nayak BS, Ria D, Narissa D, Kalere E, Gabrielle E, Richeae F, Lissette F, Amanda F, Nathanael F: **An investigation into the impact of the COVID-19 pandemic on the mental health and the study habits of medical students of the University of the West Indies.** *Psychol Health Med* 2022**:**1-10.

1679. Nazeer S, Aroosa, Munir MS, Arshad M, Ahamed F, Chaudhari MZ: **Medical Education, COVID-19 and E-Learning.** *Pakistan Journal of Medical and Health Sciences* 2022, **16:**367-370.

1680. Neelakantan M, Heitkamp NM, Blankenburg R, Frohna JG: **The #PedsMatch21 Webinar Series: Coordinated Specialty-Level Communication During the Virtual Residency Application Cycle.** *Acad Med* 2022, **97:**1012-1016.

1681. Negm AS, Elhatw A, Badawy M, Gioe ML, Khan S, Hammad MF, Shalaby N, Choucair F, Saad MA, Elfeel A, et al: **Effectiveness of virtual teaching of diagnostic and interventional imaging fundamentals to Egyptian medical students: an analytical cross-sectional study.** *Egyptian Journal of Radiology and Nuclear Medicine* 2022, **53**.

1682. Neo SHS, Zhou JX, Wong GC, Mok NK, Yee AC, Phua GL: **Teaching Communication Micro-Skills to Cardiologists Managing Seriously Ill Patients in Asia: Challenges Encountered Amidst the COVID-19 Pandemic and Future Perspectives.** *Cureus* 2021, **13:**e19957.

1683. Nguyen DD, Rourke KF, Gabara A, Matsumoto ED, Bhojani N, Domes T: **Matching to urology during the COVID-19 pandemic and with the Association of Faculties of Medicine of Canada electives diversification policy: Survey of the 2021 urology Canadian Residency Matching Service applicants.** *Can Urol Assoc J* 2022, **16:**228-230.

1684. Nguyen EV, Kim SH, Islam MA, Chang Y, Aoyagi J, Hussain A: **An entrepreneurial activity implementation and assessment among pharmacy students amid the COVID-19 pandemic lockdown.** *Pharmacy Education* 2022, **22:**16-22.

1685. Nham E, Kumar R, McAlpine K, Seabrook C, Valle M, Menard I, Watterson J, Roberts M: **Development, implementation, and uptake of a novel Canadian Resident Matching Service (CaRMS) residency recruitment committee strategy in the era of COVID-19.** *Can Urol Assoc J* 2022, **16:**206-211.

1686. Nikas IP, Lamnisos D, Meletiou-Mavrotheris M, Themistocleous SC, Pieridi C, Mytilinaios DG, Michaelides C, Johnson EO: **Shift to emergency remote preclinical medical education amidst the Covid-19 pandemic: A single-institution study.** *Anat Sci Educ* 2022, **15:**27-41.

1687. Nikendei C, Dinger-Ehrenthal U, Schumacher F, Bugaj TJ, Cranz A, Friedrich HC, Herpertz SC, Terhoeven V: **Medical students' mental burden and experiences of voluntary work in COVID-19 patient support and treatment services: a qualitative analysis.** *GMS J Med Educ* 2021, **38:**Doc120.

1688. Nix K, Liu EL, Oh L, Duanmu Y, Fong T, Ashenburg N, Liu RB: **A Distance-Learning Approach to Point-of-Care Ultrasound Training (ADAPT): A Multi-Institutional Educational Response During the COVID-19 Pandemic.** *Acad Med* 2021, **96:**1711-1716.

1689. Njambi KV, Mayoka GW: **Inequality in accessing learning during pandemic crises in developing countries: Reflections from COVID-19-induced online learning at a Kenyan pharmacy school.** *Pharmacy Education* 2021, **21:**713-722.

1690. Nodine P, Carrington S, Jenkins PA, Rosenthal L, Jones J: **Student Interviews Exploring the Influence of the Coronavirus Pandemic on Graduate Nursing Education.** *SAGE Open Nurs* 2022, **8:**23779608221090013.

1691. Nofi C, Roberts B, Demyan L, Sodhi N, DePeralta D, Zimmern A, Aronsohn J, Molmenti E, Patel V: **A Survey of the Impact of the COVID-19 Crisis on Skill Decay Among Surgery and Anesthesia Residents.** *J Surg Educ* 2022, **79:**330-341.

1692. Nonaka S, Makiishi T, Nishimura Y, Nagasaki K, Shikino K, Izumiya M, Moriya M, Sadohara M, Ohtake Y, Kuriyama A: **Prevalence of Burnout among Internal Medicine and Primary Care Physicians before and during the COVID-19 Pandemic in Japan.** *Intern Med* 2022, **61:**647-651.

1693. Noor R, Singh D, Agarwal A, Mansoori S, Ansari MI: **Perception of dental students towards the online method of dental education during the COVID-19 pandemic.** *J Oral Biol Craniofac Res* 2022, **12:**223-227.

1694. Noorani M, Manji H, Mmari E, Somji S, Walli N, Kassamali S, Adamjee S, Matillya N, Mbithe H, Nagri A, Ismail N: **Residency training on the frontlines of the COVID-19 pandemic - a qualitative study from Tanzania.** *Pan Afr Med J* 2021, **40:**28.

1695. Nour MO, Sinky TH, Natto HA: **Impact of Social Media Infodemics on Mental Health among Health Colleges’ Students at Saudi Universities during COVID-19 Pandemic.** *The Open Public Health Journal* 2022, **15**.

1696. Arekat M, Shehata MH, Deifalla A, Al-Ansari A, Kumar A, Alsenbesy M, Alshenawi H, El-Agroudy A, Husni M, Rizk D, et al: **Evaluation of the Utility of Online Objective Structured Clinical Examination Conducted During the COVID-19 Pandemic.** *Adv Med Educ Pract* 2022, **13:**407-418.

1697. Arias-Calderon M, Castro J, Gayol S: **Serious Games as a Method for Enhancing Learning Engagement: Student Perception on Online Higher Education During COVID-19.** *Front Psychol* 2022, **13:**889975.

1698. Arja SB, Fatteh S, Nandennagari S, Pemma SSK, Ponnusamy K, Arja SB: **Is Emergency Remote (Online) Teaching in the First Two Years of Medical School During the COVID-19 Pandemic Serving the Purpose?** *Adv Med Educ Pract* 2022, **13:**199-211.

1699. Arponen H, Zou-Kopsa Q, Karaharju-Suvanto T: **Examination performance of dentistry students during the COVID-19 pandemic.** *Acta Odontologica Scandinavica* 2022**:**1-7.

1700. Nozari A, Mukerji S, Lok LL, Gu Q, Buhl L, Jain S, Ortega R: **Perception of Web-Based Didactic Activities During the COVID-19 Pandemic Among Anesthesia Residents: Pilot Questionnaire Study.** *JMIR Med Educ* 2022, **8:**e31080.

1701. Nusanti S, Dearaini, Bani AP, Kartasasmita AS, Muhammad Ichsan A, Virgana R, Anggraini N, Rahayu T, Irfani I, Edwar L, et al: **Delivering a modified continuous objective structured clinical examination for ophthalmology residents through a hybrid online method.** *Korean J Med Educ* 2021, **33:**419-430.

1702. Nutting R, Ofei-Dodoo S, Rose-Borcherding K, Strella G: **Brief Mindfulness Intervention for Emotional Distress, Resilience, and Compassion in Family Physicians During COVID-19: A Pilot Study.** *PRiMER* 2022, **6:**3.

1703. Nyoni CN, Fichardt AE, Botma Y: **An innovative educational strategy for learning and teaching clinical skills during the COVID-19 pandemic.** *African Journal of Health Professions Education* 2022, **14:**3-7.

1704. O’Reilly MFM FM, K. P.: **Covid Concerns: A Radiological Perspective.** *Irish Medical Journal*, **114**.

1705. Ocak Ö, Şahin EM: **The Effects of Distance Education Applied Due to COVID-19 on Clinical Neurology Education.** *Turkish Journal Of Neurology* 2021, **27:**270-277.

1706. Odeh H, Kaddumi EG, Salameh MA, Al-Khader A: **Interactive Online Practical Histology Using the Poll Everywhere Audience Response System: An Experience During the COVID-19 Lockdown.** *International Journal of Morphology* 2022, **40:**102-106.

1707. Oducado RMF, Cleofas JV, Soriano GP: **Predicting nursing students' intention to attend face-to-face classes on school reopening: A theory of planned behavior application.** *Nurs Forum* 2022, **57:**733-738.

1708. Oh SL, Mishler O, Yang JS, Barnes C: **Effectiveness of remote simulation-based learning for periodontal instrumentation: A non-inferiority study.** *J Dent Educ* 2022, **86:**463-471.

1709. Oladele HOO JKA, Temidayo Olasinbo Afolabi, Adebukunola Olajumoke Awotorebo, Odunitan Tawakalitu.: **The perception and attitude of nursing students towards online learning during the Covid-19 lockdown in South West Nigeria.** *Knowledge Management & E-Learning: An International Journal* 2022, **14:**30-45.

1710. Olivares-Perez ME, Graglia S, Harmon DJ, Klein BA: **Virtual anatomy and point-of-care ultrasonography integration pilot for medical students.** *Anat Sci Educ* 2022, **15:**464-475.

1711. Oliver MG, Kelly K: **Student Perceptions and Use of Social Media as Residency Program Information.** *Family Medicine* 2022, **54:**380-383.

1712. Omer M, Al-Afif S, Machetanz K, Bettag C, Tatagiba M, Rohde V, Krauss JK: **Impact of COVID-19 on the Neurosurgical Resident Training Program: An Early Experience.** *J Neurol Surg A Cent Eur Neurosurg* 2022, **83:**321-329.

1713. Opsahl AG, Embree JL, Howard MS, Davis-Ajami ML, Herrington C, Wellman DS, Hodges KT: **Adapting Civility Education in an Academic-Practice Partnership.** *J Contin Educ Nurs* 2021, **52:**575-580.

1714. Oropeza-Aguilar M, Cendejas-Gomez JJ, Quiroz-Compean A, Buerba GA, Dominguez-Rosado I, Mendez-Probst CE: **Impact of COVID-19 on surgical residency training programs in Mexico City: The third victim of the pandemic. A resident's perspective.** *Cir Cir* 2022, **90:**165-171.

1715. Orsolini L, Bellagamba S, Marchetti V, Menculini G, Tempia Valenta S, Salvi V, Volpe U: **A Preliminary Italian Cross-Sectional Study on the Level of Digital Psychiatry Training, Knowledge, Beliefs and Experiences among Medical Students, Psychiatry Trainees and Professionals.** *Healthcare (Basel)* 2022, **10**.

1716. Ortadeveci A, Ermez MN, Oz S, Ozden H: **Attainments during the COVID-19: a comparative survey study on ideal anatomy education from the students' perspective.** *Surg Radiol Anat* 2022, **44:**1063-1069.

1717. Osula VO, Sanders JE, Chakare T, Mapota-Masoabi L, Ranyali-Otubanjo M, Hansoti B, McCollum ED: **COVID-19 advanced respiratory care educational training programme for healthcare workers in Lesotho: an observational study.** *BMJ Open* 2022, **12:**e058643.

1718. Overbay D, Bigand T, Springer G: **Perceptions of Live Streaming Compared With an In-Person Nursing Conference: A Quality Improvement Project.** *J Nurs Adm* 2021, **51:**645-650.

1719. Ozcan C: **Flipped Classroom in Restorative Dentistry: A First Test Influenced by the Covid-19 Pandemic.** *Oral Health Prev Dent* 2022, **20:**331-338.

1720. Ozen KE, Erdogan K, Malas MA: **Evaluation of views and perceptions of the medical faculty students about distance anatomy education during the COVID-19 pandemic.** *Surg Radiol Anat* 2022, **44:**61-71.

1721. Ozen KE, Erdogan K, Malas MA: **Assessment of the opinions and experiences of anatomy educators regarding the distance anatomy education in medical facilities under the effect of COVID-19 in Turkey.** *Surg Radiol Anat* 2022, **44:**791-802.

1722. Ozkara BB, Karabacak M, Alpaydin DD: **Student-Run Online Journal Club Initiative During a Time of Crisis: Survey Study.** *JMIR Med Educ* 2022, **8:**e33612.

1723. ÇAkir ÖZmen G, ŞİMŞEk P, Aydin R, ÇİLİNgİR D: **Nursing students’ willingness to work in COVID-19 pandemic: The role of knowledge and perceived competence: An Example FormTurkey.** *Journal of Basic and Clinical Health Sciences* 2022, **6:**20-30.

1724. Öztürk B, Akarsu R, Kayıhan H, Çelik Y, Kayhan SE: **Investigation of the factors affecting the e-learning process in occupational therapy education during the pandemic with principal component analysis.** *British Journal of Occupational Therapy* 2022, **85:**694-703.

1725. Pabst A, Goetze E, Thiem DGE, Bartella AK, Seifert L, Beiglboeck FM, Kroplin J, Hoffmann J, Zeller AN: **An update on the current training situation of German interns in oral and maxillofacial surgery at special times in 2021.** *J Craniomaxillofac Surg* 2022, **50:**380-387.

1726. Padhi KS, Balmuchu G, Acharya PS, Singh SR, Joseph T: **The Perspectives of Educators and Learners on E-Learning: A Cross-Sectional Descriptive Study in a Medical School.** *Adv Med Educ Pract* 2021, **12:**1059-1066.

1727. Palacios J, Hazkour N, Rao A, Brennan M, Oropallo A: **Virtual Events in the Era of COVID-19: Perspectives From a Virtual Interdisciplinary Wound Care Symposium.** *Wound Manag Prev* 2022, **68:**14-24.

1728. Palese A, Brugnolli A, Achil I, Mattiussi E, Fabris S, Kajander-Unkuri S, Dimonte V, Grassetti L, Danielis M: **The first COVID-19 new graduate nurses generation: findings from an Italian cross-sectional study.** *BMC Nurs* 2022, **21:**101.

1729. Paleti S, Sobani ZA, McCarty TR, Gutta A, Gremida A, Shah R, Nutalapati V, Bazerbachi F, Jesudoss R, Amin S, et al: **Impact of COVID-19 on gastroenterology fellowship training: a multicenter analysis of endoscopy volumes.** *Endosc Int Open* 2021, **9:**E1572-E1578.

1730. Palmer J, Nguyen G, Levin MR, Swamy R, Alexander J: **A novel method for strabismus surgery education using an asynchronous video module.** *J AAPOS* 2022, **26:**218-220.

1731. Palomares HRG, Pantoja PPM, Pascua KE, Pfleider ALD, Polintan ANT, Reyes RMD, Rivera APA, Ramos RP: **A case study on the perspectives of University of Santo Tomas pharmacy professors on the influence of online classes in pharmacy education.** *Pharmacy Education* 2021, **21:**759-770.

1732. Panchal V, Patel P, Chaudhari A, Goyal P: **Medical students' perception of online learning during COVID-19 pandemic in India-a survey.** *National Journal of Physiology, Pharmacy and Pharmacology* 2022, **12:**202-205.

1733. Panda R, Mishra N, Lahoti S, Prabhu RR, Mishra A, Singh K, Rai K: **Evaluation of COVID-19 ECHO training program for healthcare workers in India - A Mixed-Method Study.** *BMC Health Serv Res* 2022, **22:**883.

1734. Pandya JSN VPR, S.: **Impact of Covid-19 on Mental Health of Surgical Residents who Suffered from the Disease.** *J Assoc Physicians India*, **70:**44906.

1735. Panepucci S, Roe E, Galbraith A, Thornton T: **Learning With Laughter: Implementing Engaging Virtual Simulation During the COVID-19 Pandemic.** *Clin Simul Nurs* 2022, **62:**92-98.

1736. Papalois ZA, Aydin A, Khan A, Mazaris E, Rathnasamy Muthusamy AS, Dor F, Dasgupta P, Ahmed K: **HoloMentor: A Novel Mixed Reality Surgical Anatomy Curriculum for Robot-Assisted Radical Prostatectomy.** *Eur Surg Res* 2022, **63:**40-45.

1737. Pare G, Raymond L, Pomey MP, Gregoire G, Castonguay A, Ouimet AG: **Medical students' intention to integrate digital health into their medical practice: A pre-peri COVID-19 survey study in Canada.** *Digit Health* 2022, **8:**20552076221114195.

1738. Park J, Seo M: **Influencing Factors on Nursing Students' Learning Flow during the COVID-19 Pandemic: A Mixed Method Research.** *Asian Nurs Res (Korean Soc Nurs Sci)* 2022, **16:**35-44.

1739. Park KH, Yune SJ, Jung MK, Kim Y, Lee GH, Kim SY: **Impact of the COVID-19 pandemic on medical students of clinical clerkship in South Korea: A qualitative study exploring medical students' experiences.** *Pak J Med Sci* 2022, **38:**469-475.

1740. Parveen MK, Hannan MDJ, Ghouri MH, Anwar W, Tabassum S, Farooq Z, Abid H, Arif M: **Undergraduate Medical Education during Coronavirus Disease 19: Scope, Practices and Limitations in Developing Countries.** *Pakistan Journal of Medical and Health Sciences* 2022, **16:**251-254.

1741. Pascoe A, Johnson D, Putland M, Willis K, Smallwood N: **Differential Impacts of the COVID-19 Pandemic on Mental Health Symptoms and Working Conditions for Senior and Junior Doctors in Australian Hospitals.** *J Occup Environ Med* 2022, **64:**e291-e299.

1742. Paskal AM, Jaremkow P, Malyszczak P, Paskal W, Wojcik K, Opyrchal J, Paul MA: **Impact of COVID-19 pandemic on plastic surgery training in Europe.** *J Plast Reconstr Aesthet Surg* 2022, **75:**1696-1703.

1743. Passemard S, Faye A, Dubertret C, Peyre H, Vorms C, Boimare V, Auvin S, Flamant M, Ruszniewski P, Ricard JD: **Covid-19 crisis impact on the next generation of physicians: a survey of 800 medical students.** *BMC Med Educ* 2021, **21:**529.

1744. Passos KK, Bezerra HK, Leonel AS, Ramos-Perez FM, Martelli-Junior H, Machado RA, Bonan PR, Perez DC: **Self-regulated learning perception of undergraduate dental students during the COVID-19 pandemic: A nationwide survey in Brazil.** *J Clin Exp Dent* 2021, **13:**e987-e993.
[truncated: 119,194 more chars]
