# Supplementary figures and images for: Health worker education during the COVID-19 pandemic: global disruption, responses and lessons for the future—a systematic review and meta-analysis
Source: Hum Resour Health. 2023 Feb 24;21:13. doi: 10.1186/s12960-023-00799-4 (PMC9951171; doi:10.1186/s12960-023-00799-4)

**Additional file 3 – Supplementary Demographics and descriptive statistics**


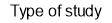


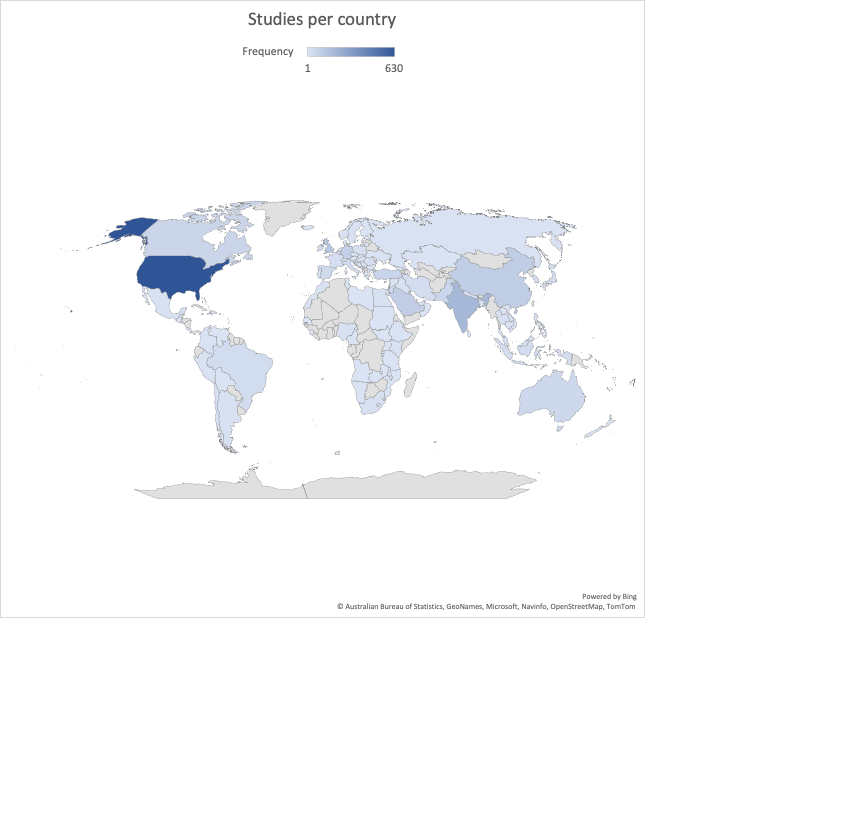

Supplement: Supplementary file 3 — Additional file 3. Descriptive Graphs and Figures for the demographics and descriptive characteristics of included individuals. [file 12960_2023_799_MOESM3_ESM.docx]
