# Supplementary material for: Health worker education during the COVID-19 pandemic: global disruption, responses and lessons for the future—a systematic review and meta-analysis
Source: Hum Resour Health. 2023 Feb 24;21:13. doi: 10.1186/s12960-023-00799-4 (PMC9951171; doi:10.1186/s12960-023-00799-4)
Supplement: Supplementary file 4 — Additional file 4. Demographics for each outcome quantitatively synthesized. [file 12960_2023_799_MOESM4_ESM.docx]

| **Additional Table** | | **Impacts of the Pandemic** | | | **Outcomes of responses** | | |
| --- | --- | --- | --- | --- | --- | --- | --- |
|  |  | **Suspension of in-person learning** | **Career disruption** | **Learner Mental Health** | **Transition to online/blended learning** | **Online assessment** | **Volunteerism initiatives** |
| **Overall** | Number of studies (k) | 314 | 193 | 287 | 1,013 | 121 | 48 |
|  | Total number of individuals (N) | 109,522 | 163,673 | 156,289 | 579,733 | 25,843 | 62,478 |
| **Learners** | N of Learners | 105,375 | 162,325 | 154,793 | 559,935 | 24,682 | 62,442 |
|  | % Women | 61.1 | 58.2 | 66.1 | 62.2 | 60.5 | 68.8 |
|  | Age (SD) in years | 28.5 (5.0) | 28.0 (4.6) | 24.3 (4.2) | 25.1 (5.7) | 25.2 (5.4) | 24.1 (3.0) |
| **Faculty** | N of Faculty | 3,317 | 969 | 1,496 | 11,994 | 983 | 36 |
|  | % Women | 89.1 | 57.3 | 59.5 | 63.8 | 36.6 | NR |
|  | Age (SD) in years | 45 (7.1) | NR | 41.3 (9.0) | 43.5 (5.8) | NR | NR |
| **Continent** | North America | 97 (34.5%) | 68 (38.4%) | 43 (15.7%) | 320 (33.0%) | 31 (26.1%) | 7 (14.9%) |
|  | South America | 14 (5.0%) | 4 (2.3%) | 11 (4.0%) | 22 (2.3%) | 2 (1.7%) | 2 (4.3%) |
|  | Europe | 83 (29.5%) | 60 (33.9%) | 63 (23.0%) | 183 (18.9%) | 15 (12.6%) | 19 (40.4%) |
|  | Asia | 69 (24.6%) | 37 (20.9%) | 144 (52.6%) | 399 (41.2%) | 64 (53.8%) | 17 (36.2%) |
|  | Africa | 11 (3.9%) | 5 (2.8%) | 11 (4.0%) | 28 (2.9%) | 4 (3.4%) | 2 (4.3%) |
|  | Oceania | 7 (2.5%) | 3 (1.7%) | 2 (0.7%) | 17 (1.8%) | 3 (2.5%) | 0 (0.0%) |
|  | **Total** | 281 (100%) | 177 (100%) | 274 (100%) | 969 (100%) | 119 (100%) | 47 (100%) |
| **WHO geographical regions** | American (AMR) | 111 (39.5%) | 72 (40.7%) | 54 (19.7%) | 342 (35.3%) | 33 (27.7%) | 9 (19.1%) |
|  | European (EUR) | 90 (32.0%) | 65 (36.7%) | 83 (30.3%) | 211 (21.8%) | 17 (14.3%) | 21 (44.7%) |
|  | African (AFR) | 7 (2.5%) | 3 (1.7%) | 4 (1.5%) | 19 (2.0%) | 1 (0.8%) | 1 (2.1%) |
|  | Eastern Mediterranean (EMR) | 30 (10.7%) | 11 (6.2%) | 66 (24.1%) | 134 (13.8%) | 28 (23.5%) | 6 (12.8%) |
|  | South East Asian (SEAR) | 29 (10.3%) | 14 (7.9%) | 32 (11.7%) | 153 (15.8%) | 31 (26.1%) | 7 (14.9%) |
|  | Western Pacific (WPR) | 14 (5.0%) | 12 (6.8%) | 35 (12.8%) | 110 (11.4%) | 9 (7.6%) | 3 (6.4%) |
|  | **Total** | 281 (100%) | 177 (100%) | 274 (100%) | 969 (100%) | 119 (100%) | 47 (100%) |
| **Setting** | University / College | 60 (20.8%) | 29 (16.6%) | 161 (62.4%) | 476 (52.3%) | 72 (62.6%) | 25 (61.0%) |
|  | WHO Health Care Provider | 229 (79.2%) | 146 (83.4%) | 97 (37.6%) | 435 (47.7%) | 43 (37.4%) | 16 (39.0%) |
|  | **Total** | 289 (100%) | 175 (100%) | 258 (100%) | 911 (100%) | 115 (100%) | 41 (100%) |
| **HCW ISCO-08 group (4-digit code)** | Medical Doctors (221) | 268 | 165 | 201 | 611 | 76 | 41 |
|  | Nursing Professionals (2221) | 10 | 5 | 31 | 109 | 9 | 5 |
|  | Midwifery Professionals (2222) | 0 | 0 | 1 | 2 | 0 | 0 |
|  | Traditional and Complementary Medicine (2230) | 0 | 0 | 1 | 0 | 0 | 0 |
|  | Paramedical Practitioners Doctors (2240) | 0 | 0 | 3 | 4 | 0 | 0 |
|  | Dentists (2261) | 25 | 13 | 28 | 87 | 18 | 2 |
|  | Pharmacists (2262) | 2 | 2 | 7 | 43 | 13 | 0 |
|  | Environmental & Occupational Health & Hygiene Professionals (2263) | 0 | 0 | 0 | 1 | 0 | 0 |
|  | Physiotherapists (2264) | 1 | 1 | 4 | 9 | 0 | 0 |
|  | Dieticians & Nutritionists (2265) | 0 | 0 | 0 | 2 | 0 | 0 |
|  | Audiologists and Speech Therapists (2266) | 0 | 0 | 0 | 3 | 0 | 0 |
|  | Optometrists & Ophthalmic Opticians (2267) | 0 | 0 | 0 | 3 | 1 | 0 |
|  | Health professionals not elsewhere classified (2269) | 1 | 1 | 1 | 8 | 0 | 1 |

**Additional File 4.** Demographics for each outcome quantitatively synthesized. Several studies are included in 2 or more columns. Unless otherwise specified, numbers refer to number of studies (k) and their category-specific proportion (%) within a given column. Numbers of learners and faculty do not always sum up to the total, since few studies report data on mixed populations. Age (SD) as well as the proportion (%) of women are derived from studies reporting such data and projected to all studies of the respective column. Total number of studies in each category do not always sum up to the column total due to overlapping and/or missing data (e.g., studies including mixed populations from 2 or more continents are omitted). Specifically, for the ISCO-08 grouping (and given that several studies provide separate data stratified by HCW group), if studies include 2 or more HCW groups they are included multiple times within the same column and thus no total is provided.

SD, standard deviation; NR, not reported.
