# Supplementary material for: Health worker education during the COVID-19 pandemic: global disruption, responses and lessons for the future—a systematic review and meta-analysis
Source: Hum Resour Health. 2023 Feb 24;21:13. doi: 10.1186/s12960-023-00799-4 (PMC9951171; doi:10.1186/s12960-023-00799-4)
Supplement: Supplementary file 5 — Additional file 5. Characteristics of eligible randomized controlled trials. PICO, findings and Risk of Bias-2 quality assessment of the 37 included randomized controlled trials. [file 12960_2023_799_MOESM5_ESM.docx]

**Additional File 5 - Randomized Clinical Trials (PICO, Findings and Risk of Bias)**

| **First author** | **Participants** | **Population** | **Intervention** | **Comparator** | **Outcome** | **Findings** | **Risk of bias** |
| --- | --- | --- | --- | --- | --- | --- | --- |
| Brazier AL, et al. | 279 | anesthesiology residents | motivational text messages on gratitude; social support; self-efficacy; and self-compassion | none | surveys on burnout well-being, meaning in work; professional value; sickness absence; and consideration of career break | no difference | low |
| Christensen L, et al. | 21 | medical students and junior doctors | video-led PPE donning and doffing session | instructor-led PPE donning and doffing session | PPE donning and doffing testing score | no difference | some concerns |
| Currat LS, et al. | 65 | first year paramedic students | face to face teaching of doffling PPE added to gemified e learning | gemified e learning only | time required, learner satisfaction, correct sequence performance, confidence knowledge, skill retention | better sequence performance, knowledge and skill retention in blended learning group | low |
| Elgzar WT, et al. | 164 | nursing students | teaching sessions on COVID-19 based on health belief model | traditional sources of information, (e.g., social media) | health belief model scale score | improved awareness with health belief model | high |
| Elrod JB, et al. | 23 | medical students | telementoring for suturing | conventional suturing | time per knot, precision, performance score, knot strength and quality score | no difference | high |
| Eraydın CAŞ, et al. | 80 | nursing students | online laughter therapy sessions | no intervention | Life Satisfaction, Psychological well being, Anxiety scales | significant positive effect | some concerns |
| Feeley AF, et al. | 21 | medical students | live-streaming virtual bedside surgical tutorials | bedside patient teaching | student perceptions and satisfaction | improved engagement, satisfaction, involvement and learning in bedside group | high |
| Hassan EAE, et al. | 163 | nursing students | CPR simulation-based flipped classroom | traditional simulation | CPR checklist, student satisfaction | increased performance and satisfaction with simulation-based flipped classroom | high |
| Heo SM, et al. | 30 | nurses | augmented reality (AR)–based self-learning platform for mechanical ventilator setuo | learning with printed manual | overall Performance, need of assistance, survey outcomes | less need for assistance, higher confidence and satisfaction in AR group | high |
| Hertling SH, et al. | 144 | medical students | virtual fishbowl for Sports Medicine Course | virtual seminar | perception questionnaire, qualitative comments, quiz scores | fishbowl group believed they were more actively involved, performed better in quiz | high |
| Hsu YMC, et al. | 127 | nurse aides | multimedia-based COVID-19 classes and skills demonstrations | traditional face-to-face approaches | knowledge, attitude, and behavioral intention | better results with novel methods | some concerns |
| Iqbal AG, et al. | 50 | dental students | hybrid e-learning educational video demonstrations and live lab demostrations for clinical skills | live in-person demonstration only | objective structured practical examination | improved competency in hybrid course | low |
| Jafree SRZ, et al. | 208 | nurses | Zoom sessions and WhatsApp-based continued learning for infection control, leadership, and communication, literacy booklet | literacy booklet | assessment of knowledge in infection prevention and control, leadership and communication, COVID-19 knowledge | improved knowledge in infection control and prevention and COVID-19 | some concerns |
| Li Y, et al. | 48 | nurses and doctors (CPD) | PPE donning and doffing video 4 times | PPE donning and doffing video twice and one live demonstration | PPE donning and doffing testing score | two video sessions and live demonstration led to better scores | low |
| Liang LF, et al. | 26 | medical students | Mindfulness skills, Emotion regulation skills, techiques of pain tolerance tutorials | no intervention | depression, anxiety scales | significant effect on symptoms | low |
| Lin L, et al. | 118 | 4th year medical students | online CPR training | face-to-face CPR training | manikin chest compression quality | no significant difference in scores | low |
| Manggala SKT, et al. | 40 | medical doctors and nurses | patient scenarios in high-fidelity simulator | patient scenarios in low-fidelity simulator | Skill in managing/communicating with the patient (Transfer skills, Communication, Team work) in pre- and post-test scores | significant improvement with high-fidelity | low |
| Moll-Khosrawi PF, et al. | 97 | first-year medical students | virtual reality and web-based Basic Life Support training | web-based Basic Life Support training | CPR preformance checklist, student satisfaction | improved performance and higher leraning gain with VR | low |
| Odeh HK, et al. | 106 | medical students | interactive histology courses with Poll-Everywhere | traditional lectures | post-course quiz scores | improved with interactive course | high |
| Pasricha ND, et al. | 10 | ophthalmology residents | remote attending feedback | no remote attending feedback | corneal suturing task in porcine eyes | objective performance did not differ | high |
| Pillong LB, et al. | 22 | medical students | video-based Head and Neck Ultrasound course | in-person course | pre- and post-training self assessment and assessment by senior physician | no difference | high |
| Pinter ZBM, et al. | 60 | medical students | online synchronous video calls and tutorials and SkillBox suturing training | face-to-face tutorial videos and SkillBox | pre- and post-training knot and suturing skills | no difference in post-scores, but significant improvement in scores with online learning | some concerns |
| Puliatti SA, et al. | 47 | medical students | educational material with pre-defined proficiency benchmarks on robotic suturing anastomosis and knot tying skills | online material without benchmarks, face-to-face lectures, peer-reviewed paper apprenticeship | objective skill assessment, theoretical knowledge | none of the trainees achieved proficiency benchmarks | high |
| Qian Q., et al. | 74 | 5th year medical students | flipped classroom with microlearning for COVID-19 diagnosis and treatment | virtual PowerPoint lecture for COVID-19 diagnosis and treatment | immediate posttest, 2-month retention test, attitude questionnaire | better retention and clinical practice attitude in intervention group | low |
| Rüllmann NH, et al. | 60 | medical students | virtual cardiac auscultation course via video chat | literature self-study | performance on auscultation simulator, satisfaction, self-assessment | improved sound description, satisfaction, self-assessment, no improvement in diagnostic accuracy | high |
| Samuels RM, et al. | 35 | pediataric interns | self-directed online curriculum on telemedicine | none | performance on Communication Checklist | improved with course | high |
| Sari Ozturk CKT, et al. | 170 | nursing students | mindfulness‐based mandala sessions | not described | Descriptive Characteristics Form, State‐trait Anxiety Inventory, Spirituality Well‐Being Scale, and Scale of Positive and Negative Experience pre- and post-test | significant reduction in anxiety and spiritual well-being | low |
| Schmitz | 58 | medical students | interactive online platform with surgical skills videos | textbook readings | online exam performance | better comprehension with online platform | high |
| Schnieders ER, et al. | 41 | medical students | asynchronousinterac tive e-learning module on chronic obstructive pulmonary disease | asynhcronous non-interactive e-learning with slides | satisfaction, usability, short-term and long-term knowledge questionnaires | no difference | high |
| Sommer GMB, et al. | 60 | medical students | 48 VR simulation tasks on surgical skills over 9 days | two VR tasks on day 1 and 9 | visual spatial ability before and after training, performance in surgical tasks | significant increase in visual spatial abilities, associated with improved performance in surgical tasks | high |
| Son HK. | 78 | nursing students | simulation problem-based learning | maternity nursing clinical practicum | learning attitude, metacognition, and critical thinking | learning attitude and critical thinking increase post-intervention higher in experimental group | high |
| Stephen TK, et al. | 24 | nursing students | simulated virtual mental health crisis (code white) scenario | standard nursing curriculum | knowledge and attitudes about mental health, workplace violence, virtual simulation | no significant difference | high |
| Suppan L, et al. | 98 | student paramedics | gamified online module on PPE | prehospital guidelines | PPE knowledge | difference in knowledge increase between groups | low |
| Suppan M, et al. | 158 | medical students | highly interactive online module on NIH Stroke Scale | didactic video on NIH Stroke Scale | NIH Stroke Scale quiz performance | better performance in interactive module group | low |
| Syamsuri DDAT, et al. | 33 | obstetrics and gynecology residnets | combination of video and simulation course for abdominal hysterectomy | video alone, simulation alone | objective structured assessment of pre- and post-intervention skills on video | improved performance in combination group | high |
| Wang XZ, et al. | 38 | obstetrics and gynecology residents | conceive-design-implement-operate (CDIO) mode | protection theory expositio | theoretical and technical score improvement | improved theoretical and technical scores, lower organizational management, workload, interpersonal relationship, and doctor-patient relationship pressure, higher job stress and professional identity evaluation scores | high |
| Zaghal AM, et al. | 118 | first, and second-year medical students | distance learning of basic suturing skills | face-to-face learning of basic suturing skills | objective performance scores,participant satisfaction | no difference | high |
