# Supplementary material for: Health worker education during the COVID-19 pandemic: global disruption, responses and lessons for the future—a systematic review and meta-analysis
Source: Hum Resour Health. 2023 Feb 24;21:13. doi: 10.1186/s12960-023-00799-4 (PMC9951171; doi:10.1186/s12960-023-00799-4)
Supplement: Supplementary file 8 — Additional file 8. Sensitivity analyses. Alternative data synthesis methods/alternative meta-analytical approaches for the main analyses. [file 12960_2023_799_MOESM8_ESM.docx]

| Analysis | Number of studies | Total individuals | Raw proportion (%) | Pooled proportion, % (DL) | 95% CI, % (DL) | Pooled proportion, % (PM) | 95% CI, % (PM) | Pooled proportion, % (HK) | 95% CI, % (HK) | I^2^ | P-value of I^2^ |
| --- | --- | --- | --- | --- | --- | --- | --- | --- | --- | --- | --- |
| Overall Learner satisfaction for online education | 599 | 425,466 | 68.5 | 75.9 | 74.2-77.7 | 76.2 | 74.0-78.2 | 75.9 | 73.8-78.0 | 99.3 | <0.001 |
| Overall Faculty satisfaction for online education | 84 | 6,525 | 58.6 | 71.8 | 66.7-76.7 | 71.9 | 66.6-77.0 | 71.8 | 66.5-76.9 | 93.9 | <0.001 |
| Overall Learner preference for online education | 215 | 94,452 | 32.4 | 32.0 | 29.3-34.8 | 32.1 | 29.1-35.2 | 32.0 | 29.0-35.1 | 98.7 | <0.001 |
| Overall Faculty preference for online education | 35 | 3,198 | 26.3 | 25.5 | 16.1-36.1 | 25.5 | 15.3-37.2 | 25.5 | 15.3-37.2 | 97.4 | <0.001 |
| Overall Learner preference for face-to-face education | 232 | 97,903 | 47.0 | 48.8 | 45.4-52.4 | 48.7 | 45.3-52.2 | 48.8 | 45.3-52.2 | 99.0 | <0.001 |
| Overall Learner preference for blended education | 65 | 14,992 | 55.9 | 56.0 | 51.2-60.7 | 56.0 | 50.0-61.9 | 56.0 | 50.0-61.8 | 96.9 | <0.001 |
| Learners wishing to keep online education post-pandemic | 126 | 59,765 | 20.7 | 34.7 | 30.7-38.8 | 34.9 | 29.8-40.1 | 34.7 | 29.7-39.9 | 99.0 | <0.001 |
| Faculty wishing to keep online education post-pandemic | 27 | 1,821 | 26.7 | 36.7 | 22.8-51.7 | 36.6 | 23.1-51.3 | 36.7 | 22.8-51.7 | 97.3 | <0.001 |
| Learners wishing to keep blended education post-pandemic | 141 | 49,585 | 60.3 | 68.1 | 64.6-71.5 | 68.2 | 64.2-72.2 | 68.1 | 64.0-72.0 | 98.4 | <0.001 |
| Learner satisfaction with online examinations | 54 | 11,072 | 54.5 | 68.8 | 60.7-76.3 | 68.8 | 60.6-76.4 | 68.8 | 60.6-76.4 | 98.6 | <0.001 |
| Learners who perceived training disruption | 220 | 66,870 | 68.1 | 71.1 | 67.9-74.2 | 71.1 | 68.1-74.1 | 71.1 | 67.9-74.2 | 98.7 | <0.001 |
| Learners wishing to prolong their training | 67 | 35,979 | 38.2 | 44.7 | 39.2-50.2 | 44.7 | 38.8-50.6 | 44.7 | 38.8-50.6 | 99.0 | <0.001 |
| Learners who were redeployed | 95 | 11,527 | 26.0 | 29.2 | 25.3-33.2 | 29.3 | 25.0-33.7 | 29.2 | 24.9-33.6 | 95.3 | <0.001 |
| Learners rethinking their career plans | 60 | 134,623 | 11.2 | 21.5 | 17.1-26.3 | 21.5 | 16.3-27.3 | 21.5 | 16.3-27.3 | 99.5 | <0.001 |
| Learners who participated in volunteering activities | 27 | 39,046 | 24.9 | 27.7 | 19.1-37.3 | 27.7 | 19.4-36.9 | 27.7 | 19.1-37.3 | 99.7 | <0.001 |
| Learners who wished to participate in volunteering activities | 26 | 28,728 | 40.5 | 62.2 | 49.2-74.4 | 62.1 | 52.2-71.6 | 62.2 | 49.2-74.4 | 99.8 | <0.001 |
| Learners who screened positive for at least moderate anxiety | 144 | 95,927 | 32.1 | 32.3 | 28.5-36.2 | 32.3 | 28.7-36.0 | 32.3 | 28.5-36.2 | 99.4 | <0.001 |
| Learners who screened positive for at least moderate anxiety based on the GAD-7 scale | 81 | 53,658 | 28.4 | 32.1 | 26.6-37.9 | 32.1 | 27.5-36.8 | 32.1 | 26.6-37.9 | 99.5 | <0.001 |
| Learners who screened positive for at least moderate depression | 122 | 84,067 | 32.1 | 32.0 | 27.9-36.2 | 32.0 | 28.1-36.0 | 32.0 | 27.9-36.2 | 99.4 | <0.001 |
| Learners who screened positive for at least moderate depression based on the PHQ-9 scale | 51 | 39,876 | 37.2 | 32.8 | 25.3-40.7 | 32.8 | 27.0-38.9 | 32.8 | 25.3-40.7 | 99.6 | <0.001 |
| Learners who screened positive for insomnia | 17 | 9,906 | 26.0 | 30.9 | 20.8-41.9 | 30.9 | 21.2-41.4 | 30.9 | 20.8-41.9 | 99.2 | <0.001 |
| Learners who screened positive for at least moderate burnout | 67 | 35,808 | 47.3 | 38.8 | 33.4-44.3 | 38.9 | 31.7-46.4 | 38.8 | 31.6-46.2 | 99.0 | <0.001 |
| Learners who screened positive for at least moderate burnout based on the MBI scale or its variants | 28 | 17,134 | 54.4 | 46.8 | 38.6-55.1 | 47.0 | 35.6-58.6 | 46.8 | 35.5-58.4 | 98.4 | <0.001 |

GAD-7, General Anxiety Disorder-7; PHQ-9, Patient Health Questionnaire; MBI, Maslach Burnout Inventory.

**Additional File 8. Sensitivity analyses.** Alternative data synthesis methods / alternative meta-analytical approaches for the main analyses.

I^2^ is calculated via the Cochran’s Q test and the P-value is obtained by comparing the statistic with a chi-square distribution with k-1 degrees of freedom (k, number of studies).

**Main approach:** DerSirmonian and Laird (DL) method for calculation of the between-study variance, estimate of the combined effect for heterogeneity derived from the inverse-variance fixed-effect model, confidence intervals (CI) calculated via the Wald-type normal distribution.

**Alternative approach 1:** Use of the Paule-Mandel (PM) estimator (identical to the empirical Bayes method) for calculation of the between-study variance.

**Alternative approach 2:** Use of the (original) Hartung-Knapp (also known as the Hartung-Knapp-Sidik-Jonkman) method (HK) for calculation of the CI. An overall effect modification factor (q) is used to multiply the overall effect variance and the final CI is given by a t-distribution.
